# RNF219/*α*‐Catenin/LGALS3 Axis Promotes Hepatocellular Carcinoma Bone Metastasis and Associated Skeletal Complications

**DOI:** 10.1002/advs.202001961

**Published:** 2020-12-31

**Authors:** Shuxia Zhang, Yingru Xu, Chan Xie, Liangliang Ren, Geyan Wu, Meisongzhu Yang, Xingui Wu, Miaoling Tang, Yameng Hu, Ziwen Li, Ruyuan Yu, Xinyi Liao, Shuang Mo, Jueheng Wu, Mengfeng Li, Erwei Song, Yanfei Qi, Libing Song, Jun Li

**Affiliations:** ^1^ Key Laboratory of Liver Disease of Guangdong Province The Third Affiliated Hospital Sun Yat‐sen University Guangzhou 510080 China; ^2^ Department of Biochemistry Zhongshan School of Medicine Sun Yat‐sen University Guangzhou 510080 China; ^3^ State Key Laboratory of Oncology in South China Collaborative Innovation Center for Cancer Medicine Sun Yat‐sen University Cancer Center Guangzhou 510080 China; ^4^ Department of Microbiology Zhongshan School of Medicine Sun Yat‐sen University Guangzhou 510080 China; ^5^ Department of Breast Oncology Sun Yat‐Sen Memorial Hospital Sun Yat‐Sen University Guangzhou 510120 China; ^6^ Centenary Institute University of Sydney Sydney 2000 Australia

**Keywords:** bone metastasis, hepatocellular carcinoma, LGALS3, RNF219, skeletal‐related events

## Abstract

The incidence of bone metastases in hepatocellular carcinoma (HCC) has increased prominently over the past decade owing to the prolonged overall survival of HCC patients. However, the mechanisms underlying HCC bone‐metastasis remain largely unknown. In the current study, HCC‐secreted lectin galactoside‐binding soluble 3 (LGALS3) is found to be significantly upregulated and correlates with shorter bone‐metastasis‐free survival of HCC patients. Overexpression of LGALS3 enhances the metastatic capability of HCC cells to bone and induces skeletal‐related events by forming a bone pre‐metastatic niche via promoting osteoclast fusion and podosome formation. Mechanically, ubiquitin ligaseRNF219‐meidated *α*‐catenin degradation prompts YAP1/*β*‐catenin complex‐dependent epigenetic modifications of LGALS3 promoter, resulting in LGALS3 upregulation and metastatic bone diseases. Importantly, treatment with verteporfin, a clinical drug for macular degeneration, decreases LGALS3 expression and effectively inhibits skeletal complications of HCC. These findings unveil a plausible role for HCC‐secreted LGALS3 in pre‐metastatic niche and can suggest a promising strategy for clinical intervention in HCC bone‐metastasis.

## Graphical abstract

Hepatocellular carcinoma (HCC)‐secreted LGALS3 induces osteolytic bone metastasis (BM) via promoting osteoclast fusion and podosome formation. Mechanistically, RNF219‐mediated *α*‐catenin degradation results in YAP1/*β*‐catenin‐dependent epigenetic modifications on LGALS3 promoter, eliciting in LGALS3 upregulation in HCC. Blocking YAP1/*β*‐catenin complex formation on LGALS3 promoter via verteporfin reduces LGALS3 expression and effectively inhibits HCC bone‐metastasis (HCC‐BM), which represents a potential strategy for HCC‐BM clinical treatment.

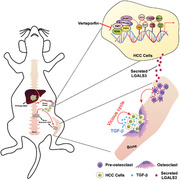

## 1 Introduction

With the improvements in diagnostic techniques, multidisciplinary treatments, and implementation of surveillance programs, the overall survival of hepatocellular carcinoma (HCC) patients has been improving markedly for the past two decades. However, the incidence of extrahepatic metastases has increased prominently. Approximately 16.1% to 38.5% of HCC patients display bone metastasis (BM) at first diagnosis^[^ ^1^ ^]^ and 11.7% HCC patients that undergo curative resections develop BM.^[^ ^2^ ^]^ The prognosis of HCC with bone metastasis (HCC‐BM) is extremely poor, with a median survival time of only 4.6 months.^[^ ^3^ ^]^ Meanwhile, most of patients with HCC‐BM are accompanied with skeletal‐related events (SREs), such as pathological fractures and spinal cord compression.^[^ ^4^ ^]^ These SREs, identified as an independent prognostic factor associated with poor overall survival,^[^ ^4^ ^]^ cause severe pain and neurologic deficits that dramatically deteriorate the quality of life of patients.^[^ ^5^, ^6^ ^]^ However, the potential therapeutic strategy for the clinical management of HCC‐BM have yet to be developed as the nature and the characteristics of HCC‐BM have not been fully explored.

Bone homeostasis is maintained by the coordinated balance between bone formation by osteoblasts and bone resorption by osteoclasts.^[^ ^7^ ^]^ However, cancer‐secreted factors could disrupt the bone homeostasis and imbalance the activities of osteoclasts/osteoblasts in bone compartments, which creates a “bone pre‐metastatic niche” to support cancer BM,^[^ ^8^ ^]^ even determining metastatic organotropism.^[^ ^9^ ^]^ Numerous studies have proved that the tumor‐produced cytokines directly or indirectly activated osteoclast, termed as osteoclastogenesis.^[^ ^10^ ^]^ Subsequently, activated osteoclasts resorb bone matrix via secretion of hydrochloric acid and matrix degrading proteases, such as tartrate‐resistant acid phosphatase (TRAP) and MMPs, which forms a “vicious cycle” involving the release of various bone matrix‐bound factors that facilitate seeding and expansion of metastatic tumor cells in the bone.^[^ ^8^, ^11^ ^]^

Expression of lectin galactoside‐binding soluble 3 (LGALS3), a member of the galectin family of carbohydrate‐binding proteins, is frequently upregulated in various solid and blood malignancies and plays broad roles in cancer progression through distinct intracellular and extracellular mechanisms.^[^ ^12^ ^]^ Recently, cancer‐secreted LGALS3 has emerged as an important regulator in modulation of tumor microenvironment and correlated with metastasis.^[^ ^13^ ^]^ In this study, we found that HCC‐secreted LGALS3 induced HCC bone‐metastasis and SREs by facilitating the formation of a pre‐metastatic niche. We further demonstrated that E3 ligase RNF219‐mediated *α*‐catenin degradation, which induced YAP1/*β*‐catenin‐dependent epigenetic modifications on LGALS3 promoter, eliciting LGALS3 upregulation. Importantly, the inhibition of YAP1/*β*‐catenin complex formation on LGALS3 promoter by verteporfin reduced LGALS3 expression and effectively inhibited HCC‐BM in nude mice. Therefore, our results represent a potential strategy for clinical treatment of skeletal complications of HCC.

## 2 Results

### 2.1 RNF219 Overexpression Correlates with HCC Bone‐Metastasis

**Figure 1:**
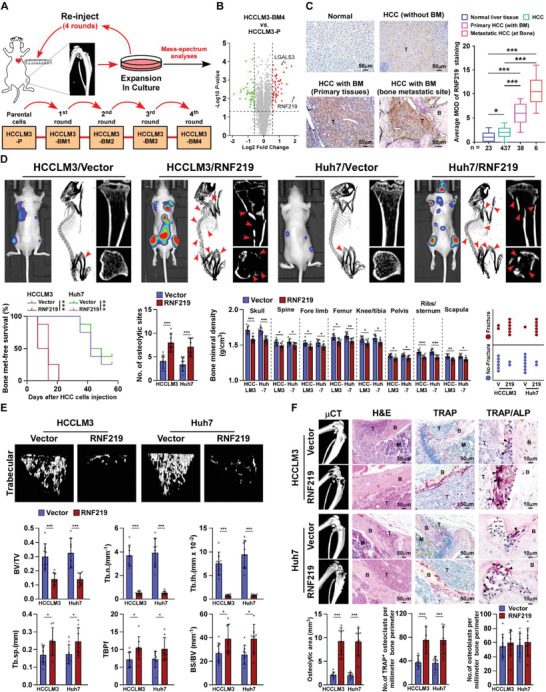
RNF219 overexpression promotes bone metastasis and SREs in HCC. A) Schematic representation of the establishment of a highly bone‐metastasis HCCLM3‐BM4 cell line. Tumor cells were isolated from bone lesions in mice injected intracardially with HCCLM3‐P/luc cells and cultured, and re‐injected intracardially into mice. This procedure was repeated for four cycles. B) Volcano plot analysis of dysregulated proteins comparing HCCLM3‐BM4 cells with HCCLM3‐P cells. C) Representative images (left) and quantification (right) of RNF219 expression in normal liver tissue (*n* = 23), HCC tissues without bone‐metastasis (*n* = 437), primary HCC tissues with bone‐metastasis (*n* = 38), and HCC bone‐metastasis tissues (*n* = 6) (left panel). Scale bar, 50 µm. D) Upper: BLI (left) and µCT images (middle and right) of bone lesions from representative mice. Arrowheads: fractured bone site. Lower: Kaplan–Meier bone metastasis‐free survival curve and quantification of the osteolytic sites, BMD and fracture frequency from representative mice (*n* = 8/group). E) µCT images of trabecular section (upper) and quantification (lower) of bone parameters from representative mice (*n* = 8/group). BV/TV, bone/tissue volume ratio; BS/TV, bone surface/ tissue volume ratio; Tb. *n*, trabecular number; Tb. sp., trabecular separation; Tb. th., trabecular thickness; TBPf, trabecular bone pattern factor. F) µCT and histological (H&E, TRAP and TRAP/ALP) images (upper) and quantification (lower) of osteolytic area and TRAP^+^‐osteoclasts/ALP^+^‐osteoblasts along the bone‐tumor interface of metastases from representative mice (*n* = 8/group). Scale bar, 50 µm. Each error bar in panels (C−F) represents the mean ± SD of three independent experiments. Significant differences were determined by one‐way ANOVA with Tukey's multiple comparison test (C–F). * *p* < 0.05, ** *p* < 0.01, *** *p* < 0.001.

To investigate the mechanism underlying HCC bone‐metastasis, highly bone‐metastatic HCCLM3‐BM4 cells were established using a bone metastatic mouse model after four rounds of intracardial injection (**Figure** **1**A), as previously reported.^[^ ^14^ ^]^ As shown in the schematic in Figure 1A, the HCCLM3‐parental cells, which stably expressed firefly luciferase reporter, were inoculated into the left cardiac ventricle of immunodeficient mice, and the formed bone metastatic tumors were monitored by bioluminescence signal (BLI). Then bone metastatic HCCLM3 cells (named as HCCLM3‐BM1) were recovered from BLI‐suspected bone sites, expanded in culture, and re‐injected intracardially into mice for the next round. In the 4th round, the mice intracardially injected with HCCLM3‐BM4 cells only displayed a strong metastatic signal in bone, but not in lung, for less than 3 weeks, whereas HCCLM3‐parental cells injected‐mice showed simultaneous metastasis at bone and other organs over 5 weeks (Table S1, Supporting Information). To identify critical factors that contribute to HCC bone‐metastasis, mass spectrometry‐based proteomics was performed in HCCLM3‐BM4 and HCCLM3‐parental cells. Protein profiling revealed that a total of 94 dysregulated proteins, including 53 upregulated proteins and 41 downregulated proteins, in HCCLM3‐BM4 cells compared with HCCLM3‐parental cells (Figure 1B and Table S2, Supporting Information). Among them, ubiquitin ligase RNF219 was found consistently elevated in high‐bone‐metastatic HCC cells and tissues compared to low‐bone‐metastatic HCC cells, non‐metastatic or other organ metastatic HCC tissues, respectively (Figure S1A,B, Supporting Information and Figure 1B,C). Furthermore, statistical analysis revealed that patients with high RNF219‐expressed HCC had significantly shorter bone‐metastasis‐free survival than those with low RNF219‐expressed HCC (*p* = 0.016 and Figure S1C and Table S3–S5, Supporting Information). These results suggest that *RNF219* overexpression is associated with progression of bone‐metastasis in HCC.

### 2.2 RNF219 Overexpression Promotes Bone Metastasis and SREs in HCC

To determine whether RNF219 overexpression induces HCC‐BM, we monitored the progression of BM after the intracardiac injection of control‐ and RNF219‐transduced HCC cells, which stably expressed firefly luciferase reporter. Prominently, the mice intracardially injected with RNF219‐transduced HCC cells displayed earlier systemic bone metastatic onsets and a larger bone metastatic tumor‐burden (Figure S1D,E, and Table S6, Supporting Information and Figure 1D). Micro‐CT (μCT) analysis showed that RNF219 also contributed to bone remodeling, as indicated by increased systematic severe osteolytic bone lesions, a reduced systemic bone mineral density (BMD), and a higher frequency of SREs, such as pathological fracture (Figure 1D). Meanwhile, the relative trabecular volume, trabecular number, and trabecular thickness were significantly increased while the trabecular separation and trabecular bone pattern factor were decreased in HCC/RNF219 cells‐injected mice compared to control mice (Figure 1E). Histological analysis revealed a larger osteolytic area and increased TRAP^+^‐osteoclasts, but no alteration of alkaline phosphatase (ALP)^+^‐osteoblasts, along the bone‐tumor interface in RNF219/mice (Figure 1F). All these results suggested that RNF219 contributes to HCC‐BM and SREs development.

Consistently, compared with control mice, the RNF219‐silenced HCC cells‐injected mice exhibited delayed bone metastases, reduced BM lesions/osteolytic areas, less BMD reduction, and fewer SREs frequency (Figure S2A–G and Table S6, Supporting Information). Histological TRAP staining showed that RNF219‐silenced HCC cells suppressed osteoclasts activation (Figure S2H, Supporting Information). Taken together, our results implicate RNF219 contributes to skeletal complications of HCC.

### 2.3 RNF219‐induced LGALS3 Promotes Osteoclastogenesis

**Figure 2:**
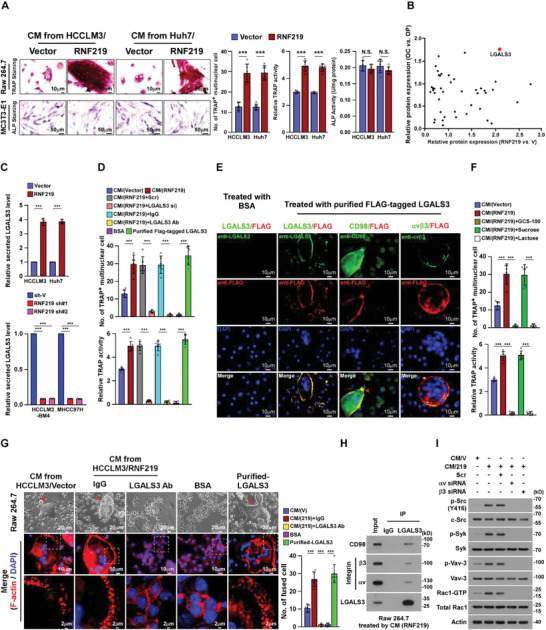
RNF219 induced‐LGALS3 promotes osteoclastogenesis in vitro. A) Left: Osteoclast differentiation assays by TRAP staining (upper) or osteoblast differentiation assay by ALP staining (lower) in the presence of CM from indicated cells. Right: Quantification of the number of TRAP^+^‐multinuclear osteoclasts, TRAP activity and ALP activity from the experiment in the left panel. B) Scatter diagram generated from dysregulated proteins in CM‐HCCLM3/vector compared with CM‐HCCLM3/RNF219 and in osteoclast (OC) compared with osteoclast precursor (OP). A full list is available in Table S7, Supporting Information. C) ELISA analysis of secreted LGALS3 protein expression in CM from indicated cells. D) Osteoclast differentiation assays in the presence of the indicated CM, or BSA, or purified LGALS3 from CM‐HCCLM3/Flag‐tagged LGALS3 cells. E) Osteoclast precursor Raw 264.7 cells were treated with BSA, or purified LGALS3 from CM‐HCCLM3/flag‐tagged LGALS3 cells, and then IF staining of LGALS3, Flag‐LGALS3, CD98 and integrin *α*v*β*3. Scale bar, 10 µm. F) Quantification of the osteoclast differentiation in the presence of the CM‐HCCLM3/Vector, or CM‐HCCLM3/RNF219, or CM‐HCCLM3 plus GCS‐100, or CM‐HCCLM3 plus sucrose, or CM‐HCCLM3 plus lactose. G) Left: Phase contrast micrograph of RAW 264.7 cells as indicated treatments (upper) and IF staining images of phalloidin (F‐actin) (middle and lower). Scale Bar, 20 µm (upper), 10 µm (middle) and 2 µm (lower). Right: Quantification of the number of fused multinuclear cells from the experiment in the left panel. H) Co‐IP assays using anti‐LGALS3 or anti‐IgG antibodies in CM‐HCCLM3/RNF219‐treated RAW264.7 cells and WB analysis of expression of CD98, integrin *α*v, integrin *β*3, and LGALS3. I) WB analysis of phosphorylation level of SRC, SYK, and VAV‐3 and expression of RAC‐GTP in Raw 264.7 cells as indicated treatments. *β*‐actin served as the loading control. Each error bar in panels A, C, D, F, and G represents the mean ± SD of three independent experiments. Significant differences were determined by one‐way ANOVA with Tukey's multiple comparison test (A, C, D, F, and G). ** *p* < 0.01, *** *p* < 0.001 and N.S.: not significant (*p* > 0.05).

Treatment with conditioned media (CM) from HCC/RNF219 cells significantly increased the number of TRAP^+^‐multinuclear osteoclasts and TRAP enzymatic activity, suggesting that RNF219 upregulation might enhance the capability of HCC cells in creating a bone tumor microenvironment (**Figure** **2**A). However, it had no effect on differentiation of pre‐osteoblast MC3T3‐E1 cells, as ALP^+^‐osteoblasts and the relative RANKL/OPG ratio were unaltered (Figure S3A, Supporting Information), suggesting that RNF219‐induced secretome promotes osteoclastogenesis.

Analysis of the protein profiling in CM collected from HCC/vector and HCC/RNF219 cells and the proteomics data of mature osteoclast (OC) and osteoclast precursor (OP), ^[^ ^15^ ^]^ indicated that LGALS3 was one of the secreted proteins with the most pronounced upregulation in HCC/RNF219 cells and OCs (Figure 2B and Table S7, Supporting Information). Consistent with elevated mRNA level of LGALS3 in HCCLM3‐BM4 and RNF219‐transduced HCC cells (Figure S3B,C, Supporting Information), the secreted LGALS3 protein levels were also significantly increased in HCCLM3‐BM4 and RNF219‐overexpressed cells but decreased in RNF219‐silenced cells (Figure 2C and Figure S3D,E, Supporting Information). Importantly, the LGALS3 protein levels in bone‐metastatic HCC tissues were also significantly higher than that in other organ metastatic HCC tissues (Figure S3F, Supporting Information), suggesting the potential role of LGALS3 in HCC‐BM.

Stimulation with CM from HCC/RNF219 cells (CM‐HCC/RNF219) or with purified secreted‐LGALS3 from HCC cells dramatically induced TRAP^+^‐multinuclear osteoclasts formation and TRAP activity (Figure 2D). In contrast, the osteoclastogenic effect of CM from HCC/RNF219 cells on osteoclastogenesis was significantly abolished by LGALS3 silencing or using LGALS3‐neutralizing antibody (Figure 2D). Therefore, our results demonstrate that LGALS3 plays an essential role in RNF219‐induced osteoclastogenesis.

### 2.4 RNF219‐Induced LGALS3 is Involved in Differentiation and Activation of Osteoclasts

Interestingly, we found that HCC‐secreted LGALS3 could still induce LGALS3‐knockout Raw 264.7 osteoclast progenitor cells to form multi‐nuclear osteoclasts (Figure 2E). Meanwhile, expression of markers regarding differentiation and activation of osteoclasts, including C‐fos, Acp5, Ctsk, Nfat‐c1, and Dc‐stamp, were significantly increased in CM‐HCC/RNF219‐treated osteoclasts but decreased in response to treatment with the LGALS3‐neutralizing antibody (Figure S3G, Supporting Information). Removal of HCC‐secreted LGALS3 from the surface of osteoclasts via lactose or LGALS3 antagonist GCS‐100 drastically impaired the CM‐HCC/RNF219‐induced osteoclastogenesis (Figure 2F), indicating that HCC secreted‐LGALS3 was required for the fusion of mono‐nuclear progenitor osteoclasts to multi‐nuclear mature osteoclasts.

Podosome formation in bone‐attached multi‐nuclear osteoclasts to develop an actin‐rich sealing zone is crucial for the bone resorption.^[^ ^16^ ^]^ IF staining showed that treatment with purified secreted‐LGALS3 or CM‐HCC/RNF219 dramatically promoted podosome formation, as indicated by increased actin ring formation, in mature osteoclasts (Figure 2G). Therefore, our results demonstrate that HCC‐secreted LGALS3 promotes differentiation and activation of osteoclasts.

Interestingly, we did not observe that HCC‐secreted LGALS3 interacted with osteoclast differentiation inhibitor myosin‐2A.^[^ ^17^ ^]^ However, we found that LGALS3 bound to and stabilized CD98 and integrin *α*v/*β*3, the key factors in osteoclastogenesis and osteoclast activation,^[^ ^18^ ^]^ in osteoclasts surface (Figure 2E,H and Figure S3H, Supporting Information). The inducing effect of CM‐HCC/RNF219 on osteoclastogenesis and activation of integrin *α*v/*β*3‐signalling, evidenced by the increased phosphorylation level of SRC, SYK, and VAV‐3 and expression of RAC‐GTP, was profoundly mitigated by CD98‐ or integrin *β*3‐silencing (Figure 2I and Figure S3I, Supporting Information). These results suggest that HCC secreted‐LGALS3‐induced osteoclastogenesis through lattices formation and activation of CD98 and integrin *α*v/*β*3 complex.

### 2.5 RNF219‐Induced LGALS3 Promotes Osteolytic Destruction and Bone Metastasis

**Figure 3:**
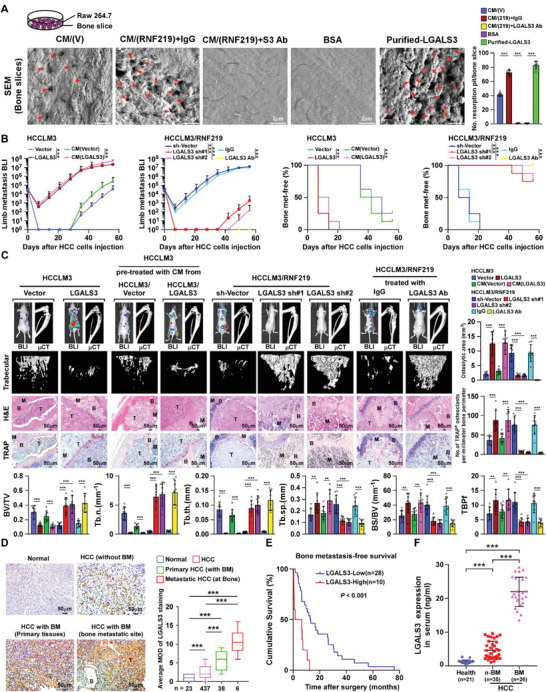
LGALS3 promotes osteolytic bone metastasis of HCC. A) Bone resorption assays of RAW 264.7 cells cultured onto the bone slices for indicated treatments. Then bone slice was fixed for scanning electron microscopy (SEM) (left) and quantification of the number of resorption pit per bone slice (right). B) Normalized BLI signals of bone metastases and Kaplan–Meier bone metastasis‐free survival curve of mice from the indicated experimental group (*n* = 8/group). C) Upper left: BLI, μCT (longitudinal and trabecular section), and histological (H&E and TRAP staining) images of bone lesions from representative mice. Scale bar, 50 µm. Upper right and lower: Quantification of the μCT osteolytic lesion area and TRAP^+^ osteoclasts along the bone‐tumor interface of metastases (upper right) and bone parameters (lower) from the experiment in the upper left panel. D) Representative images (left) and quantification (right) of LGALS3 expression in normal liver tissue (*n* = 23), HCC tissues without bone metastasis (*n* = 437), primary HCC tissues with bone metastasis (*n* = 38), and HCC tissues in bone metastatic site (*n* = 6) (left panel). Scale bar, 50 µm. E) Kaplan–Meier analysis of bone metastasis‐free survival curves in HCC‐BM with low versus high expression of LGALS3 (*n* = 38; *p* < 0.001, log‐rank test). F) ELISA analysis of serum LGALS3 expression from healthy donors (*n* = 21), HCC patients without bone metastasis (*n*‐BM, *n* = 35), HCC patients with bone metastasis (BM, *n* = 26). Each error bar in panels (A–D) and (F) represents the mean ± SD of three independent experiments. Significant differences were determined by one‐way ANOVA with Tukey's multiple comparison test (A–D, F). ** *p* < 0.01, *** *p* < 0.001.

Bone resorption assay showed that the surface of bone slice was severely eroded by Raw 264.7 cells treated with purified LGALS3 and CM‐HCC/RNF219, as indicated by increased resorption pits formation (**Figure** **3**A). However, these effects were abolished by LGALS3‐neutralizing antibody treatment (Figure 3A and Figure S9C, Supporting Information), suggesting that the HCC‐secreted LGALS3 induced a vicious cycle formation.

Furthermore, the bone‐metastasis in vivo assay showed that overexpression of LGALS3 in HCC cells or mice pretreated with CM from LGALS3‐transduced HCC cells significantly enhanced the bone metastatic capability of HCC cells, as indicated by earlier systemic BM, and also promoted HCC‐mediated osteolytic bone disease, as shown by systemic severe osteolytic bone lesions, reduced systemic BMD, and higher frequency of SREs (Figure 3B,C and Figure S3K,L, Supporting Information). Consistently, a larger osteolytic area and increased TRAP^+^‐osteoclasts were observed along the bone‐tumor interface in corresponding mice (Figure 3C). However, silencing LGALS3 in HCC cells or treated mice with LGALS3‐neutralizing antibodies dramatically decreased the bone metastatic capability and osteolytic destructive effect of HCC cells (Figure 3B,C and Figure S3J–L, Supporting Information), indicating that LGALS3 was a vital target to inhibit HCC‐BM.

Prominently, LGALS3 expression was hardly detected in normal liver tissues and low in non‐bone‐metastatic HCC tissues whereas it was markedly higher in primary HCC‐BM tissues and further elevated in HCC bone‐metastasis tissue (Figure 3D). Patients with high LGALS3‐expressed HCC exhibited significantly shorter bone‐metastasis‐free survival than those with low LGALS3‐expressed HCC (*p* < 0.001; Figure 3E). Meanwhile, the serum level of LGALS3 in HCC‐BM patients was significantly higher than that in HCC patients without bone‐metastasis (Figure 3F), suggestive of serum LGALS3 level as a potential biomarker for HCC‐BM.

### 2.6 RNF219‐Mediated *α*‐Catenin Degradation Induces LGALS3 Upregulation

**Figure 4:**
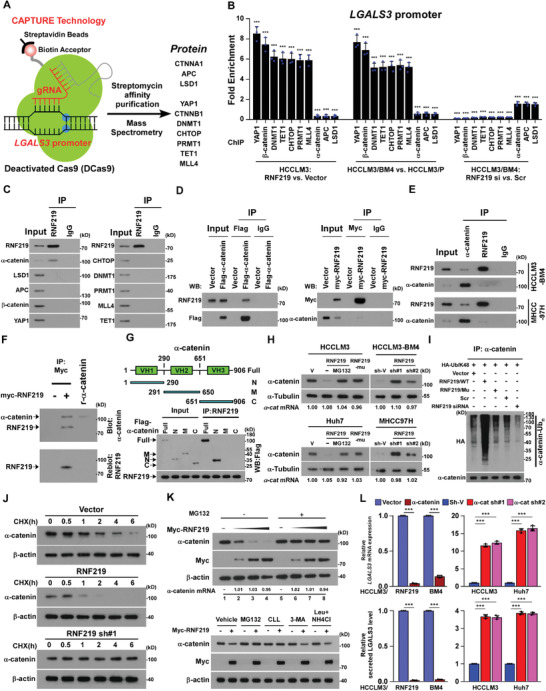
RNF219‐mediated *α*‐catenin proteasomal degradation induced LGALS3 upregulation. A) Schematic of dCas9‐mediated capture of LGALS3 promoter using five sequence‐specific sgRNAs (Left) and mass spectrometry (MS) analysis of trans‐regulatory factors targeting LGALS3 promoter (right). B) ChIP analyses of enrichment of the indicated trans‐regulatory factors on the LGALS3 promoter. C) Co‐IP analysis of interaction of RNF219 with the indicated trans‐regulatory factors in HCCLM3 cells. D) Co‐IP/IB analysis of expression of RNF219 and immunoprecipitated flag‐*α*‐catenin (left) and IB analysis of expression of *α*‐catenin and immunoprecipitated myc‐RNF219 (right) in the indicated cells. E) Co‐IP/IB analyses of expression of RNF219 and *α*‐catenin using indicated antibodies. F) Far‐western blotting analysis was performed using anti‐Myc antibody‐immunoprecipitated proteins and detected using anti‐*α*‐catenin antibody and then reblotted with anti‐RNF219 antibody. Recombinant *α*‐catenin served as the control. G) Schematic illustration of the wild‐type and truncated *α*‐catenin protein (upper) and co‐IP assays were performed using anti‐RNF219 antibody in the indicated cells (lower). H) IB analysis of *α*‐catenin expression and I) K48‐linked polyubiquitin levels of *α*‐catenin in the indicated cells. *α*‐tubulin served as a loading control. J) IB analysis of the half‐life of *α*‐catenin protein in the indicated cells treated with cycloheximide. *β*‐actin served as a loading control. K) IB analysis of *α*‐catenin and myc‐RNF219 expression in the 0, 0.5, 1.5, and 5.0 µg of myc‐RNF219‐tranfected cells treated without or with MG132 (20 µm, upper) or in the myc‐RNF219‐tranfected cells treated with the vehicle or each inhibitor (20 µm MG132, 20 µm cLL, 10 mm 3‐MA, or 100 µm leupeptin and 20 mm NH4Cl) (lower). *β*‐actin served as a loading control. L) Real‐time PCR analysis and ELISA analysis of mRNA and serum LGALS3 expression in the indicated cells. GAPDH served as a loading control. Each error bar in panels B, L represents the mean ± SD of three independent experiments. Significant differences were determined by Student's *t*‐test (B) and one‐way ANOVA with Tukey's multiple comparison test (L). *** *p* < 0.001.

To investigate the mechanism underlying RNF219‐induced LGALS3 upregulation, we employed CRISPR affinity purification in situ of regulatory elements (CAPTURE) approach following the mass spectrometry to identify the trans‐regulatory factors targeting LGALS3 promoter. As shown in **Figure** **4**A and Table S8, Supporting Information, 25 potential trans‐regulatory factors were enriched on the LGALS3 promoter in HCCLM3 cells. ChIP‐qPCR assays identified that expression of *α*‐catenin, APC and LSD1 on LGALS3 promoter was significantly decreased in HCCLM3/RNF219 and HCCLM3‐BM4 cells but increased in RNF219‐silenced cells (Figure 4B), while overexpressing RNF219 increased while silencing RNF219 reduced levels of YAP1, *β*‐catenin, DNMT1, CHTOP, PRMT1, TET1, and MLL4 on LGALS3 promoter (Figure 4B). These results suggest that multiple trans‐regulatory factors are involved in LGALS3 regulation.

co‐IP assayed showed that among abovementioned regulators, only *α*‐catenin physically interacted with RNF219 (Figure 4C). We further characterized that the first vinculin homology (VH) domain of *α*‐catenin directly bound to RNF219 (Figure 4D–G). Consistent with the ubiquitin ligase activity of RNF219,^[^ ^19^ ^]^ overexpressing RNF219 decreased while silencing RNF219 increased the expression and half‐life of *α*‐catenin protein, and conversely increased or decreased K48‐linked polyubiquitination of *α*‐catenin protein, but without effect on mRNA expression (Figure 4H–K, and Figure S4A, Supporting Information). Importantly, restoring *α*‐catenin in RNF219‐high expressed HCC cells evidently reduced, but silencing *α*‐catenin in RNF219‐low expressed HCC cells increased, both mRNA and secreted protein levels of LGALS3 (Figure S4C, Supporting Information and Figure 4L). Therefore, our results indicate that RNF219‐mediated *α*‐catenin degradation induced LGALS3 upregulation.

### 2.7 *α*‐Catenin Reduction is Vital for RNF219/LGALS3 Axis‐Induced BM and SREs

**Figure 5:**
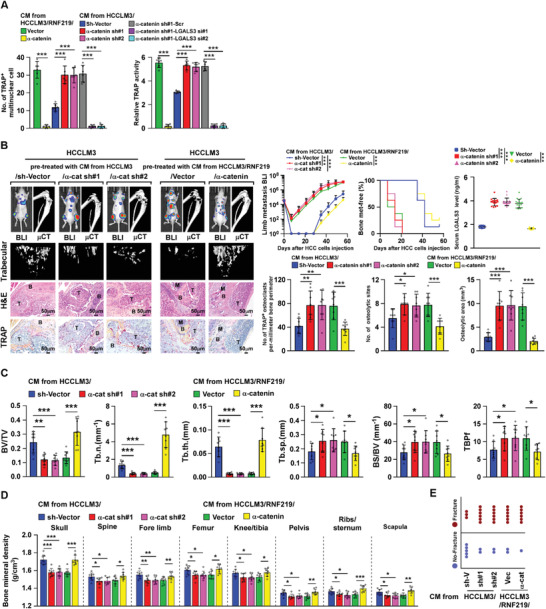
*α*‐catenin reduction is vital for RNF219/LGALS3‐induced bone metastasis and SREs. A) Quantification of the osteoclast differentiation in the presence of CM collected from the indicated cells. B) Left: BLI, μCT (longitudinal and trabecular section), and histological (H&E and TRAP staining) images of bone lesions from representative mice. Scale bar, 50 µm. Right upper: Normalized BLI signals of bone metastases, Kaplan–Meier bone metastasis‐free survival curve of mice from indicated experimental mice and ELISA analysis of serum LGALS3 expression in the indicated mice (*n* = 8/group). Right lower: quantification of the TRAP^+^ osteoclasts along the bone‐tumor interface of metastases and μCT osteolytic lesion sites and area from experiment in left panel. C) Quantification of the bone parameters analyzed by μCT assay, and D) BMD, and E) fracture frequency in the indicated mice from the experiment in Figure 5B. Each error bar in panels (A–D) represents the mean ± SD of three independent experiments. Significant differences were determined by one‐way ANOVA with Tukey's multiple comparison test (A–D). * *p* < 0.05, ** *p* < 0.01, *** *p* < 0.001.

Next, the role of *α*‐catenin reduction in RNF219/LGALS3‐induced HCC skeletal complications was examined. As shown in **Figure** **5**A, overexpressing *α*‐catenin significantly reduced the ability of HCC/RNF219 cells to induce TRAP^+^‐multinuclear osteoclasts formation and TRAP activity, whereas silencing *α*‐catenin strongly promoted the ability of HCC cells to induce osteoclastogenesis. However, the osteoclastogenic effect of *α*‐catenin silencing on HCC cells‐mediated osteoclastogenesis was abrogated by LGALS3 ablation (Figure 5A and Figure S4D, Supporting Information), strongly indicating a critical role of RNF219/*α*‐catenin/LGALS3 axis in the regulation of osteoclastogenesis in vivo.

In agreement with this, in mice pre‐treated with CM the (*α*‐catenin silenced‐HCCLM3 cells)‐induced bone microenvironment accelerated HCCLM3 cells BM; as shown by early and increased systemic bone metastatic onsets and larger bone metastatic tumor‐burden, higher serum LGALS3 level, and severe osteolytic skeletal complications; as indicated by larger osteolytic area, lower systemic BMD, and increased TRAP^+^‐osteoclasts (Figure 5B–E). However, recovering *α*‐catenin impeded the stimulatory effect of CM (HCCLM3/RNF219 cells) on bone destruction and the bone metastatic progression of HCCLM3 cells (Figure 5B–E). These results provided further evidence that *α*‐catenin reduction is vital for RNF219‐induced HCC‐BM.

### 2.8 RNF219‐Mediated *α*‐Catenin Reduction Activates Wnt/*β*‐Catenin and YAP1 Pathways

**Figure 6:**
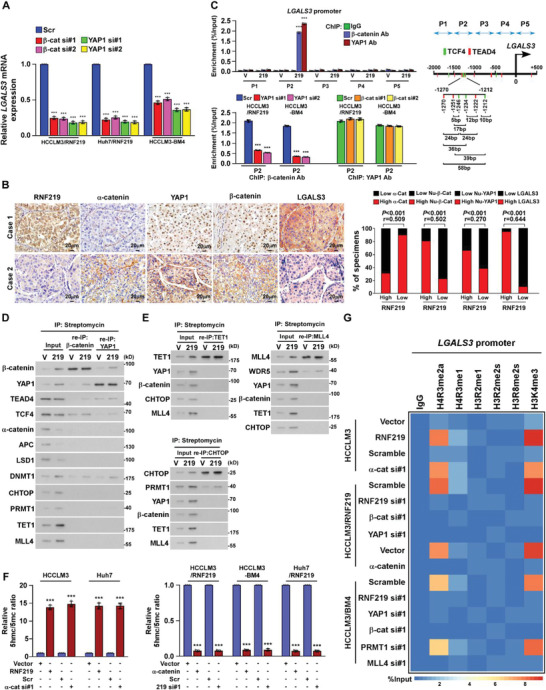
RNF219 induced spatial epigenetic modifications of the LGALS3 promoter. A) Real‐time PCR analysis of mRNA level of LGALS3 in the indicated cells. GAPDH serve as a loading control. B) RNF219 levels were negatively associated with *α*‐catenin and positively related to nuclear *β*‐catenin, YAP1, or LGALS3 expression in 475 human HCC specimens. Left: Two representative specimens are shown. Scale bars, 20 µm. Right: Percentages of specimens showing low or high RNF219 expression relative to the levels of *α*‐catenin, nuclear *β*‐catenin, nuclear YAP1, or LGALS3. C) Left: ChIP assay analyses of enrichment of *β*‐catenin and YAP1 on the LGALS3 promoter in the indicated cells. Right: Schematic illustration of TCF4 and TEAD4 binding site at LGALS3 promoter. D) Re‐co‐IP assay, using CAPTURE‐approached proteins, analyses of interaction of *β*‐catenin or YAP1 with the indicated trans‐regulatory factors, identified in experiment in Figure 4A, in vector‐ or RNF219‐transduced HCCLM3 cells. E) Re‐co‐IP assay, using CAPTURE‐approached proteins, analyses of interaction of TET1 (left upper), or CHTOP 9 (left lower), or MLL4 (right upper) with the indicated trans‐regulatory factors on the LGALS3 promoter in the vector‐ or RNF219‐transduced HCCLM3 cells. F) Relative 5hmc/5mc ratio was examined in the indicated cells. G) Heatmap represented by pseudocolors was generated using the ChIP‐qPCR values that represented the enrichment of H4R3me2a, H4R3me1, H2AR11me1, H3R2me1, H3R2me2s, H3R8me2s, and H3K4me3 on the LGALS3 promoter in the indicated cells. Each error bar in panels (A,C,F) represents the mean ± SD of three independent experiments. Significant differences were determined by one‐way ANOVA with Tukey's multiple comparison test (A,C,F). *** *p* < 0.001.

*α*‐Catenin plays vital roles in inhibition of multiple oncogenic signallings, including Wnt/*β*‐catenin signaling and YAP/TEAD signaling.^[^ ^20^ ^]^ As silencing *β*‐catenin or YAP1 significantly reduced LGALS3 expression in RNF219‐overexpressed cells (Figure S5A, Supporting Information and **Figure** **6**A), we therefore reasoned that RNF219‐mediated *α*‐catenin degradation might contribute to the activation of *β*‐catenin and YAP1 signallings. We found that the nuclear expression, phosphorylation level, and transcriptional activity of *β*‐catenin and YAP1, as well as their downstream target genes expression of both *β*‐catenin and YAP1, were significantly increased in RNF219‐transduced cells but decreased in RNF219‐silenced cells (Figure S5B–E, Supporting Information). Restoring *α*‐catenin abrogated RNF219 induced‐LGALS3 expression and transactivities of both *β*‐catenin and YAP1 (Figure S5E,F, Supporting Information). Moreover, IHC statistical analyses revealed positive correlations of RNF219 level with the expression of nuclear *β*‐catenin (*p* < 0.001, *r* = 0.502), nuclear YAP1 (*p* < 0.001, *r* = 0.270), and LGALS3 (*p* < 0.05, *r* = 0.644) but negative correlation with *α*‐catenin (*p* < 0.001, *r* = 0.509) (*n* = 475 and Figure 6B), which provided clinical evidence that RNF219‐mediated *α*‐catenin reduction activated both Wnt/*β*‐catenin and YAP1 pathways, consequently resulting in LGALS3 upregulation in HCC.

### 2.9 *α*‐Catenin Reduction Induces YAP1/*β*‐Catenin Complex Enriched on LGALS3 Promoter

ChIP assays revealed that levels of both *β*‐catenin and YAP1 were dramatically increased at P2 region (−1500 bp ≈ −1000 bp) on LGALS3 promoter in RNF219‐overexpressed or *α*‐catenin‐silenced HCC cells (Figure 6C and Figure S5G, Supporting Information). CONSITE program analysis showed that multiple TCF4‐ and TEAD4‐binding sites are pretty close together to each other at P2 region on the LGALS3 promoter (Figure 6C). Consistently, re‐co‐IP assays revealed that YAP1/TEAD4 and *β*‐catenin/TCF4 were associated together on LGALS3 promoter (Figure 6D). Interestingly, silencing YAP1 in RNF219‐upregulated cells dramatically reduced *β*‐catenin level, whereas silencing *β*‐catenin only slightly decreased YAP1 enrichment, on LGALS3 promoter (Figure 6C and Figure S5H, Supporting Information), suggesting that YAP1 stabilized *β*‐catenin on the LGALS3 promoter.

### 2.10 YAP1/*β*‐Catenin Complex Induces a Succession of Epigenetic Modifications on LGALS3 Promoter

Our CAPTURE/MS analyses revealed that DNA methyltransferase DNMT1, methylcytosine dioxygenase TET1, CHTOP/arginine methyltransferase PRMT1‐methylosome complex, and histone methyltransferase complex MLL4/WDR5 were also enriched at LGALS3 promoter in RNF219‐upregulated HCC cells (Figure 4A). Similar to the effect of YAP1 and *β*‐catenin on LGALS3 upregulation, silencing DNMT1, TET1, CHTOP, PRMT1, MLL4 or WDR5 also reduced LGALS3 mRNA expression in RNF219‐upregulated cells (Figure S6A, Supporting Information), suggesting that these trans‐regulatory factors might likewise be involved in RNF219‐induced LGALS3 expression.

Furthermore, re‐co‐IP assays indicated that RNF219 upregulation promoted the binding of DNMT1 to YAP1/*β*‐catenin complex at LGALS3 promoter (Figure 6D). Although DNA methylation is a major epigenetic mechanism for gene silencing,^[^ ^21^ ^]^ binding of DNMT1 has been reported to increase nuclear *β*‐catenin level and induced *β*‐catenin/TCF‐driven transcription and DNA methylation in colorectal cancer cells.^[^ ^22^ ^]^ DNMT1‐mediated C5 position of cytosine bases (5mC) in CpG dinucleotide might also play an important role in transcriptional regulation. For instance, methylcytosine dioxygenases, such as TET family, catalytically convert 5mC into 5‐hydroxymethylcytosine (5hmC) that serves as a recruitment signal for CHTOP/arginine methyltransferase‐methylosome complex to H4R/H3R histone modification.^[^ ^23^ ^]^ Such modifications further elicit histone methyltransferase‐mediated H3K4 methylation for transcriptional activation.^[^ ^24^ ^]^ Consistently, our reciprocal co‐IP showed that YAP1/*β*‐catenin/DNMT1, TET1, CHTOP/PRMT1, and MLL4/WDR5 complex were independently associated on LGALS3 promoter (Figure 6E). However, silencing YAP1 or *β*‐catenin reduced the expression of DNMT1, TET1, CHTOP/PRMT1, and MLL4/WDR5 at LGALS3 promoter, downregulation of DNMT1 or TET1 reduced the enrichment of CHTOP/PRMT1, and MLL4/WDR5 complex on LGALS3 promoter, with no impacts on YAP1 and *β*‐catenin levels (Figure S6B, Supporting Information).

Consistent with the biological function of each trans‐regulatory complex, the relative 5hmc/5mc ratio and levels of H4R3me2a and H3K4me3 on LGALS3 promoter were increased in RNF219‐upregulated and *α*‐catenin‐silenced HCC cells but decreased in RNF219‐silenced and *α*‐catenin‐transduced cells (Figure 6F,G). Silencing TET1, or CHTOP/PRMT1, or MLL4/WDR5 respectively reduced the relative 5hmc/5mc ratio and decreased H4R3me2a and H3K4me3 levels on LGALS3 promoter in RNF219‐upregulated cells (Figure S6C–E, Supporting Information). These results further demonstrate that YAP1/*β*‐catenin complex induces continuous epigenetic modifications‐mediated LGALS3 upregulation.

### 2.11 Disrupting YAP1/TEAD Interaction via Verteporfin Represses LGALS3 Expression

**Figure 7:**
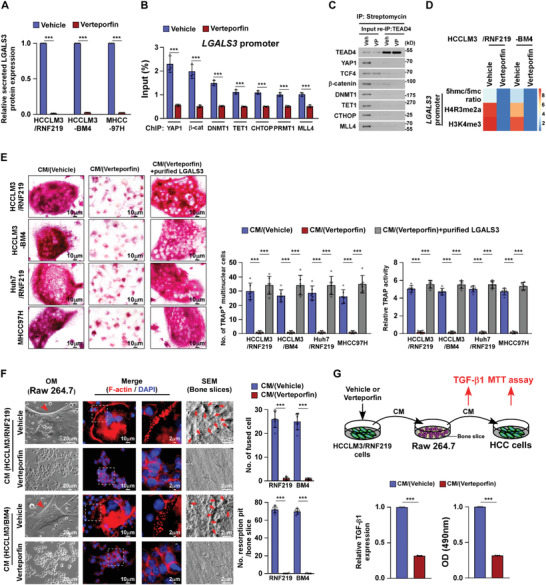
Verteporfin treatment represses LGALS3 expression and inhibits osteoclastogenesis. A) ELISA analysis of secreted LGALS3 protein expression in the indicated cells. B) ChIP assays analyses of enrichment of YAP1, *β*‐catenin, DNMT1, CHTOP, PRMT1, TET1, and MLL4 on the LGALS3 promoter in the vehicle‐ or verteporfin (10 µm)‐treated cells. C) Re‐co‐IP assay, using CAPTURE‐approached proteins, analyses of the interaction of TEAD4 with the indicated trans‐regulatory factors in the vehicle‐ or verteporfin (10 µm)‐treated cells. D) Heatmap represented by pseudocolors was generated using the ChIP‐qPCR values that represented the relative 5hmc/5mc ratio and enrichment of H4R3me2a and H3K4me3 on the LGALS3 promoter in the vehicle‐ or verteporfin (10 µm)‐treated cells. E) Left: Osteoclast differentiation assays by TRAP staining (left) in the presence of CM from the indicated cells treated with vehicle or verteporfin (10 µm). Right: Quantification of the number of TRAP^+^ multinuclear cells and TRAP activity from the experiment in the left panel. F) Left: Phase contrast and IF (staining of F‐actin) images of RAW 264.7 cells and SEM images of bone slice in the presence of CM from vehicle‐ or verteporfin (10 µm)‐treated cells. Right: Quantification of the number of fused multinuclear cells and resorption fit per bone slice TRAP activity from the experiment in the left panel. G) Upper: Schematic illustration of verteporfin inhibited “vicious cycle” between cancer cells and osteoclasts. Lower left: ELISA analysis of TGF‐*β*1 levels in CM from RAW 264.7 cells cultured onto the bone slice in the presence of CM form vehicle‐ or verteporfin (10 µm)‐treated cells. Lower right: MTT analysis of growth curves of HCC cells from the experiment in the upper panel. Each error bar in panels (A,B,E–G) represents the mean ± SD of three independent experiments. Significant differences were determined by Student's *t*‐test (A,B,E–G). *** *p* < 0.001.

Our results showed that YAP1 could stabilize *β*‐catenin on LGALS3 promoter (Figure 6C,D and Figure S5G,H, Supporting Information), indicating the key role of YAP1/TEAD interaction in LGALS3 regulation. We then examined the effect of verteporfin, a small‐molecule antagonist of the YAP‐TEAD interaction^[^ ^25^ ^]^ on LGALS3 expression. Strikingly, verteporfin‐treated RNF219‐upregulated cells not only showed significant reduction of LGALS3 at both mRNA and secreted protein levels, but also displayed decreased the formation of TEAD4/YAP1/*β*‐catenin/DNMT1 complex, reduced enrichment levels of CHTOP/PRMT1 and MLL4/WDR5 complex and less relative 5hmc/5mc ratio and H4R3me2a and H3K4me3 levels on LGALS3 promoter (**Figure** **7**A–D and Figure S7A, Supporting Information). These results demonstrate that disrupting YAP1/TEAD interaction using verteporfin represses LGALS3 expression.

### 2.12 Verteporfin Treatment Suppresses RNF219/LGALS3‐Induced Osteoclastogenesis

As shown in Figure 7E, the effect of RNF219‐induced osteoclastogenesis was profoundly inhibited by verteporfin treatment, as indicated by decreased TRAP^+^‐multinuclear osteoclasts and TRAP activity, but reversed by addition of purified‐LGALS3 from HCC cells. Meanwhile, podosome formation and bone resorption assays indicated that verteporfin treatment dramatically reduced the capability of CM/RNF219‐upregulated HCC cells‐induced fusion, actin ring formation, and resorption activity of osteoclasts (Figure 7F), indicating that verteporfin treatment inhibited osteoclastogenesis. Moreover, the HCC‐induced vicious cycle was also inhibited by verteporfin, as evidenced by reduction in bone matrix‐released TGF‐*β* and decreased growth rates of HCC cells (Figure 7G).

### 2.13 Verteporfin Treatment Blocks Initiation and Progression of HCC‐BM In Vivo

**Figure 8:**
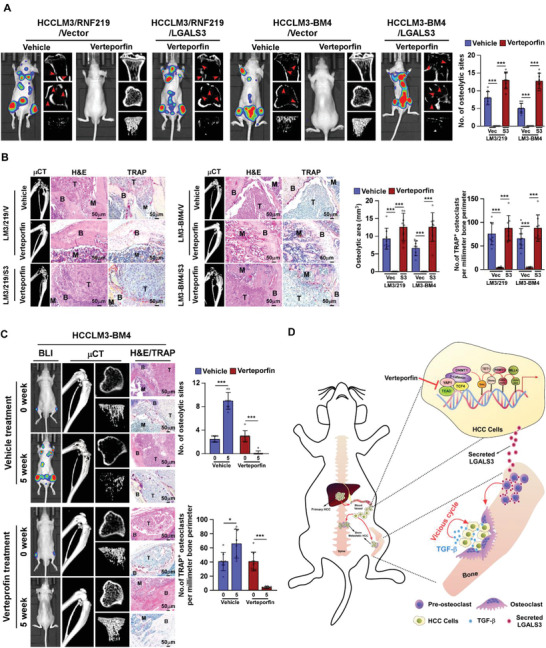
verteporfin treatment inhibits osteolytic bone metastasis of HCC. A) BLI and μCT (longitudinal, cross and trabecular section) images of bone lesions from vehicle‐ or verteporfin (20 mg kg^−1^)‐treated mice (*n* = 8/group) intracardially injected with HCCLM3/vector, or HCCLM3/RNF219, or HCCLM3/RNF219/LGALS3 cells (left) and quantification of the osteolytic sites (*n* = 8/group) (right) from the experiment in the left panel. B) Histological (H&E and TRAP) images of bone lesions (left and middle) and quantification of the osteolytic lesion area and TRAP^+^ osteoclasts along the bone‐tumor interface of metastases (right) from representative mice from experiment in (A). Scale bar, 50 µm. C) Left: BLI, μCT (longitudinal, cross, and trabecular section) and histological (H&E and TRAP) images of HCCLM3‐BM4‐injected mice (*n* = 8/group) treated with verteporfin (20 mg kg^−1^) at indicated time. Right: quantification of the osteolytic sites and TRAP^+^ osteoclasts along the bone‐tumor interface of metastases from the representative mice from the experiment in the left panel. D) Model: ubiquitin ligase RNF219‐mediated *α*‐catenin degradation prompted YAP1/*β*‐catenin‐dependent epigenetic modifications of LGALS3 promoter, resulting in LGALS3 upregulation and metastatic bone diseases, and verteporfin therapy might serve as a promising approach to inhibit HCC bone‐metastasis. Each error bar in panels (A–C) represents the mean ± SD of three independent experiments. Significant differences were determined by Student's *t*‐test (A–C). * *p* < 0.05, *** *p* < 0.001.

Finally, the in vivo effects of verteporfin on HCC‐BM and SREs were examined. To this end, the high‐bone‐metastatic HCCLM3/RNF219 and HCCLM3‐BM4 cells were intracardially injected into nude mice accompanied with verteporfin treatment. Strikingly, BL imaging and μCT analyses did not detect the BM lesions/osteolytic areas, BMD reduction, and pathologic fracture in verteporfin‐treated mice (**Figure** **8**A,B, and Figure S7B–E, Supporting Information). The number of TRAP^+^‐osteoclasts in bone surface area in verteporfin‐treated mice were drastically less than that in vehicle‐treated control mice (Figure 8B). These results suggest that verteporfin treatment prevents the initiation of HCC bone‐metastasis and SREs. However, genetically engineered to overexpress LGALS3 in HCC cells abrogated the inhibitory effect of verteporfin on skeletal complications of HCC (Figure 8A,B and Figure S7B–F, Supporting Information), which further supported the essential role of LGALS3 in HCC‐BM.

We further examined the effect of verteporfin on progression of HCC bone‐metastasis using a preclinical model of HCC bone‐metastasis, in which verteporfin treatment started after bone‐metastatic tumors formed. As shown in Figure 8C, more bone metastases and a larger bone‐metastatic tumor burden were further developed in vehicle‐treated mice, accompanied by increased osteolytic areas and higher numbers of TRAP^+^‐osteoclasts along the bone‐tumor interface. However, verteporfin‐treated mice displayed decreased bone‐metastases, reduced tumor burden, and fewer TRAP^+^‐osteoclasts along the bone‐tumor interface, consequently resulting in the longer survival of bone‐metastatic mice (Figure 8C). Therefore, these results further support our hypothesis that RNF219‐mediated *α*‐catenin degradation prompted LGALS3 upregulation via YAP1/*β*‐catenin‐dependent epigenetic modifications, which results in metastatic bone diseases, and verteporfin treatment might be a new therapeutic approach against HCC‐BM (Figure 8D).

## 3 Discussion

Before the 20th century, the survival of HCC patients was too short to regard extrahepatic metastases as a clinical challenge. The improvements in both diagnostic modalities and therapeutic procedures have markedly prolonged the survival of HCC patients over the past two decades, but also led to a concurrent worsening of the tumor progression within the extrahepatic organs and formation of metastatic foci at distant sites.^[^ ^4^, ^26^ ^]^ For instance, the incidence of bone metastases in HCC is becoming more common, occurring in 25.5% to 38.5% of patients with extrahepatic disease.^[^ ^1^, ^2^ ^]^ Meanwhile, the concomitantly developed SREs in HCC‐BM patients are morbid events that diminish the quality of life and increase the healthcare utilization costs.^[^ ^5^, ^6^ ^]^ However, no practical guideline for treatment of HCC‐BM have been developed yet, as mechanisms underlying HCC‐BM remain elusive. Herein, we reported that HCC‐secreted LGALS3 plays a vital role in bone metastases and SREs via modulation of bone remodeling. Importantly, verteporfin, the antagonist of the YAP/TEAD interaction, decreased LGALS3 expression and effectively blocked the initiation and progression of HCC metastatic bone diseases. Therefore, these findings uncover a plausible mechanism for HCC‐BM and might provide a new strategy for clinical intervention in HCC‐related bone diseases.

Our results showed that HCC‐secreted LGALS3 could directly induce osteoclastogenesis, which was consistent with the effect of breast cancer‐secreted LGALS3 on promoting osteoclast differentiation.^[^ ^17^ ^]^ Interestingly, it has been also reported that prostate cancer‐secreted LGALS3 inhibits osteoblast, but not osteoclast, differentiation.^[^ ^27^ ^]^ Hence, secreted‐LGALS3 from different cancer types may have distinct biological activities in modulation of bone microenvironment via diverse mechanisms. Indeed, breast cancer‐secreted LGALS3 nullified the suppressive effect of myosin‐2A on osteoclast differentiation and prostate cancer‐secreted cleaved LGALS3 activated Notch signaling to induce osteoblast differentiation.^[^ ^17^ ^]^ We demonstrated that HCC‐secreted LGALS3 localized on the outer membrane surface of osteoclast progenitor cells and formed lattices with and activated CD98‐ and integrin *α*v/*β*3 complex–mediated fusion and podosome formation of osteoclasts, leading to osteolytic bone remodeling. Thus, our results unveil a plausible role of HCC‐secreted LGALS3 in HCC‐related bone diseases, placing LGALS3 at the focal position for the treatment of skeletal complications of HCC. Considering the crucial role of LGALS3 in modulation of the bone tumor microenvironment and that bone metastases are a common clinical outcome of various solid cancers, the activities and mechanisms of other cancer‐derived LGALS3 underlying bone metastases are worthy of being further investigated.

In line with the elevated RNF219 expression in high‐bone‐metastatic HCC cells and tissues, we found that RNF219‐mediated LGALS3 upregulation induced HCC‐BM and SREs by facilitating pre‐metastatic niche formation. These results suggested that RNF219 might be a potential driver in promoting HCC‐BM. Although the mRNA level of RNF219 was also significantly increased in HCCLM3‐BM4 cells compared to HCCLM3‐P cells (Figure S8A, Supporting Information), genomic DNA and TCGA dataset analyses showed that the genomic locus of RNF219 was not amplified in bone‐metastatic HCC cells and tissues, which indicated that RNF219 upregulation was not associated with genomic gain. Interestingly, the MethPrimer bioinformatics tool revealed that the RNF219 promoter region contains a high frequency of CpG sites (Figure S8B, Supporting Information), suggesting that methylation of RNF219 promoter might be involved in RNF219 expression. Consistent with this hypothesis, the 5‐methylcytosine (5mc) level in RNF219 promoter in high bone‐metastatic HCCLM3‐BM4 cells and HCC‐BM tissues was significantly less than that in HCCLM3‐P cells and non‐BM HCC tissues (Figure S8C,D, Supporting Information). Importantly, treated with 5‐Aza‐2’‐deoxycytidine (5‐Aza‐dC), a DNA methyltransferase inhibitor, robustly increased the transcript level of RNF219 in HCCLM3‐P cells but has a slight effect on HCCLM3‐BM4 cells (Figure S8E, Supporting Information). Moreover, ChIP assays revealed that the enrichment of 5mc and DNMT1 and DNMT3B, but not DNMT3A, were significantly higher in HCCLM3‐P cells than those in HCCLM3‐BM4 cells (Figure S8F,G, Supporting Information). The precise mechanism underlying promoter hypomethylation‐mediated RNF219 upregulation needs to be further investigated.

HCC‐BM is thought to be a terminal stage disease, curative tumor resection/ablation is generally not recommended. The current treatment strategies, such as adjuvant radiofrequency ablation and transcatheter arterial chemoembolization, are mainly directed at the palliation of pain for relief from bone metastases.^[^ ^6^ ^]^ With respect to the controlling of the bone tumor microenvironment, bisphosphonates (BPs), a class of pyrophosphate analogues with a high affinity for bone minerals,^[^ ^28^ ^]^ and denosumab, a humanized monoclonal antibody against RANKL, have been FDA‐approved to reduce this cancer‐mediated bone destruction.^[^ ^29^ ^]^ Nevertheless, the severe side effects of these drugs have brought long‐term safety concerns. Herein, we found that targeting RNF219/*α*‐catenin/LGALS3 axis, such as LGALS3 neutralizing antibody or YAP inhibitor verteporfin, could effectively inhibit HCC‐BM in nude mice. Since currently there is no neutralizing anti‐LGALS3 antibody that has been used for clinical treatment and even tested in clinical trial. Therefore, it would take a long time to develop an anti‐LGALS3 antibody that could be used clinically for treatment. However, verteporfin has been approved as a clinical drug for treatment of macular degeneration.^[^ ^30^ ^]^ Using mouse model showed a prominent therapeutic efficacy of verteporfin against metastatic bone lesions and metastasizing osteosarcoma.^[^ ^31^ ^]^ Meanwhile, more than 10 clinical trials are currently in progress to test the therapeutic effects of verteporfin in a variety of cancers, including advanced pancreatic carcinoma, metastatic breast cancer, recurrent prostate cancer, and Stage III or Stage IV melanoma (https://www.clinicaltrials.gov). Thus, YAP inhibitor verteporfin might be the most promising medicine for future clinical testing.

Multiple signaling pathways, such as TGF‐*β*, NF‐*κ*B, and Wnt/*β*‐catenin, have been well documented to be participated in BM of multiple cancer types.^[^ ^32^ ^]^ Among these, TGF‐*β* signaling has been reported to be a crucial contributor for cancer BM via induction of local invasion and angiogenesis, promotion of pre‐metastatic niche and initiation of osteolytic vicious cycle.^[^ ^32^ ^]^ Interestingly, we found that the expression and phosphorylation level of SMAD2 and SMAD3, as well as the secreted TGF‐*β* level, were nearly the same between HCCLM3‐BM4 and ‐P cells, and HCCLM3/Vector and /RNF219 cells treated with or without TGF‐*β* (Figure S9A, Supporting Information), suggesting that TGF‐*β* signaling may not contribute to local invasion and pre‐metastatic niche formation during metastasis of HCCLM3‐BM4 and HCCLM3/RNF219 cells to bone. However, treatment with TGF‐*β* factor could significantly promote the proliferation of these two HCC cells (Figure S9B, Supporting Information). We further found that accompanied by severe erosion in the bone slice by Raw 264.7 cells, treated with CM‐HCC/RNF219 (Figure 3A), the bone matrix‐released TGF‐*β* was also significantly elevated that further promoted the proliferation of HCCLM3‐BM4 cells (Figure S9C, Supporting Information). Therefore, our results suggested that TGF‐*β* signaling was involved in HCC‐BM by inducing osteolytic vicious cycle.

In summary, our study provides substantial evidence to address that HCC‐secreted LGALS3 is clinically and functionally relevant to the HCC‐related bone diseases, including BM and SREs. The current findings not only improve the understanding of the mechanism driving the bone pre‐metastatic niche formation but also provide invaluable insights and new therapeutic strategies for the prevention and treatment of bone‐metastasis and skeletal complications of HCC.

## 4 Experimental Section

##### CAPTURE System

CAPTURE system was carried out according to a previous report.^[^ ^33^ ^]^ Briefly, the three components in the CAPTURE system, including a FB‐dCas9 and a biotin ligase BirA (purchased from Addgene, Watertown, MA, USA; 100 547 and 100 548), and target‐specific sgRNAs (targeting the promoter of LGALS3, listed in Table S9, Supporting Information), were transfected into HCC cells and the genomic locus‐associated proteins were isolated using streptavidin purification and further analyzed using mass spectrometry.

##### Patient Information

This study, which complied with all relevant ethical regulations for work with human participants, was conducted on a total of 23 tumor‐adjacent normal liver tissues and 475 paraffin‐embedded HCC samples that were histopathologically and clinically diagnosed at the Third Affiliated Hospital and Sun Yat‐sen University Cancer Center from 2005 to 2018. Among HCC samples, 279 primary HCC tissues without metastasis and 196 extrahepatic metastatic HCC samples (21 primary HCC biopsies from HCC patients with extrahepatic metastasis at diagnosis and 175 primary HCC tissues from patients that underwent hepatic surgery but developed extrahepatic metastasis within 3 years). Among the extrahepatic metastatic HCC samples, 38 primary HCC samples were with bone‐metastasis and 6 HCC biopsies were at bone site, while 158 primary HCC samples were with other organ metastasis. The detailed clinical information was summarized in Table S3–5, Supporting Information. The study protocols were approved by the Institutional Research Ethics Committee of Sun Yat‐sen University for the use of these clinical materials for research purposes. All Patients’ samples were obtained according to the Declaration of Helsinki and each patient signed a written informed consent for all the procedures.

##### Immunohistochemistry (IHC)

IHC analysis was performed to determine altered protein expression in paraffin‐embedded normal liver tissues, HCC tissues and BM tissues with anti‐RNF219 (abcam224493), anti‐LGALS3 (abcam2785), anti‐*β*‐catenin (CST#8480), anti‐*α*‐catenin (Sigma C2081), and anti‐YAP1(CST#14 074) antibodies overnight at 4 °C. The degree of immunostaining of formalin‐fixed, paraffin‐embedded sections were reviewed and scored separately by two independent pathologists blinded to the histopathological features and patient data of the samples. The scores were determined by combining the proportion of positively‐stained tumor cells and the intensity of staining. The scores given by the two independent pathologists were combined into a mean score for further comparative evaluation. Tumor cell proportions were scored as follows: 0, no positive tumor cells; 1, <10% positive tumor cells; 2, 10–35% positive tumor cells; 3, 35–75% positive tumor cells; 4, >75% positive tumor cells. The staining intensity was graded according to the following standard: 1, no staining; 2, weak staining (light yellow); 3, moderate staining (yellow brown); 4, strong staining (brown). The staining index (SI) was calculated as the product of the staining intensity score and the proportion of positive tumor cells. Using this method of assessment, we evaluated protein expression in normal liver tissues, HCC tissues and BM tissues by determining the SI, with possible scores of 0, 2, 3, 4, 6, 8, 9, 12, and 16. Samples with an SI ≥ 8 were determined as high expression and samples with an SI < 8 were determined as low expression. Cutoff values were determined on the basis of a measure of heterogeneity using the log‐rank test with respect to overall survival.

##### Statistical Analysis

All data were presented as the mean ± standard deviation (SD). *n* represents the number of independent experiments performed on different mice or different batches of cells or different clinical tissues. Statistical analysis was performed using the Student's two‐tailed *t*‐test and one‐way analysis of variance (ANOVA). Bivariate correlations between study variables were calculated by Spearman's rank correlation coefficients. Survival curves were plotted by the Kaplan–Meier method and compared by the log‐rank test. The significance of various variables for survival was analyzed by univariate and multivariate Cox regression analyses. *p*‐values of 0.05 or less were considered statistically significant. Statistical analysis was performed using the GraphPad Prism 7 and SPSS 19.0 statistical software. Representation of the *p*‐values was *p < 0.05, **p < 0.01, ***p < 0.001, and N.S.: not significant (*p* > 0.05).

## Conflict of Interest

The authors declare no conflict of interest.

## Supporting information

Supporting Information

## References

[1] a) M. Natsuizaka , T. Omura , T. Akaike , Y. Kuwata , K. Yamazaki , T. Sato , Y. Karino , J. Toyota , T. Suga , M. Asaka , J. Gastroenterol. Hepatol. 2005, 20, 1781; 16246200 10.1111/j.1440-1746.2005.03919.x

[2] Z. L. Xiang , Z. C. Zeng , Z. Y. Tang , J. Fan , J. He , H. Y. Zeng , X. D. Zhu , Oncologist 2011, 16, 1028. 21665914 10.1634/theoncologist.2010-0358 PMC3228144

[3] N. N. Liu , D. L. Shen , X. Q. Chen , Y. L. He , Chin. J. Oncol. 2010, 32, 203.

[4] J. J. Harding , G. Abu‐Zeinah , J. F. Chou , D. H. Owen , M. Ly , M. A. Lowery , M. Capanu , R. Do , N. E. Kemeny , E. M. O'Reilly , L. B. Saltz , G. K. Abou‐Alfa , J. Natl. Compr. Cancer Network 2018, 16, 50. 10.6004/jnccn.2017.7024 29295881

[5] C. Greco , L. Forte , P. Erba , G. Mariani , Q. J. Nucl. Med. Mol. Imaging 2011, 55, 337. 21738111

[6] R. Coleman , J. J. Body , M. Aapro , P. Hadji , J. Herrstedt , Ann. Oncol. 2014, 25, iii124. 24782453 10.1093/annonc/mdu103

[7] X. Chen , Z. Wang , N. Duan , G. Zhu , E. M. Schwarz , C. Xie , Connect. Tissue Res. 2018, 59, 99. 28324674 10.1080/03008207.2017.1290085 PMC5612831

[8] B. Ell , Y. Kang , Cell 2012, 151, 690. 23101634 10.1016/j.cell.2012.10.005

[9] a) Y. Liu , X. Cao , Cancer Cell 2016, 30, 668; 27846389 10.1016/j.ccell.2016.09.011

[10] a) A. Maurizi , N. Rucci , Cancers 2018, 10, 218; 10.3390/cancers10070218 PMC6071064 29954079

[11] W. J. Boyle , W. S. Simonet , D. L. Lacey , Nature 2003, 423, 337. 12748652 10.1038/nature01658

[12] a) P. P. Ruvolo , Bioch. Biophys. Acta 2016, 1863, 427; 10.1016/j.bbamcr.2015.08.008 26264495

[13] A. Fortuna‐Costa , A. l. M. Gomes , E. O. Kozlowski , M. P. Stelling , M. S. G. Pavão , Front. Oncol. 2014, 4, 138. 24982845 10.3389/fonc.2014.00138 PMC4058817

[14] R. Hou , Y. W. Wang , H. F. Liang , Z. G. Zhang , Z. M. Liu , B. H. Zhang , B. X. Zhang , X. P. Chen , J. Cancer Res. Clin. Oncol. 2015, 141, 1931. 25820528 10.1007/s00432-015-1958-6 PMC11823781

[15] E. An , M. Narayanan , N. P. Manes , A. Nita‐Lazar , Mol. Cell. Proteomics 2014, 13, 2687. 25044017 10.1074/mcp.M113.034371 PMC4188996

[16] S. L. Teitelbaum , F. P. Ross , Nat. Rev. Genet. 2003, 4, 638. 12897775 10.1038/nrg1122

[17] K. Nakajima , D. H. Kho , T. Yanagawa , Y. Harazono , V. Hogan , W. Chen , R. Ali‐Fehmi , R. Mehra , A. Raz , Cancer Res. 2016, 76, 1391. 26837763 10.1158/0008-5472.CAN-15-1793 PMC4863655

[18] a) H. Tsumura , M. Ito , M. Takami , M. Arai , X. K. Li , T. Hamatani , A. Igarashi , S. Takada , K. Miyado , A. Umezawa , Y. Ito , Biochem. Biophys. Rep. 2016, 5, 203; 28955825 10.1016/j.bbrep.2015.11.023 PMC5600448

[19] P. Coulombe , J. Nassar , I. Peiffer , S. Stanojcic , Y. Sterkers , A. Delamarre , S. Bocquet , M. Mechali , Nat. Commun. 2019, 10, 2426. 31160578 10.1038/s41467-019-10321-x PMC6547688

[20] a) S. H. Choi , C. Estaras , J. J. Moresco , J. R. Yates 3rd , K. A. Jones , Genes Dev. 2013, 27, 2473; 24240237 10.1101/gad.229062.113 PMC3841736

[21] E. Hervouet , P. Peixoto , R. Delage‐Mourroux , M. Boyer‐Guittaut , P. F. Cartron , Clin. Epigenet. 2018, 10, 17. 10.1186/s13148-018-0450-y PMC5807744 29449903

[22] J. Song , Z. Du , M. Ravasz , B. Dong , Z. Wang , R. M. Ewing , Mol. Cancer Res. 2015, 13, 969. 25753001 10.1158/1541-7786.MCR-13-0644 PMC4470773

[23] H. Takai , K. Masuda , T. Sato , Y. Sakaguchi , T. Suzuki , T. Suzuki , R. Koyama‐Nasu , Y. Nasu‐Nishimura , Y. Katou , H. Ogawa , Y. Morishita , H. Kozuka‐Hata , M. Oyama , T. Todo , Y. Ino , A. Mukasa , N. Saito , C. Toyoshima , K. Shirahige , T. Akiyama , Cell Rep. 2014, 9, 48. 25284789 10.1016/j.celrep.2014.08.071

[24] S. S. Dhar , S. H. Lee , P. Y. Kan , P. Voigt , L. Ma , X. Shi , D. Reinberg , M. G. Lee , Genes Dev. 2012, 26, 2749. 23249737 10.1101/gad.203356.112 PMC3533079

[25] a) Y. Liu‐Chittenden , B. Huang , J. S. Shim , Q. Chen , S. J. Lee , R. A. Anders , J. O. Liu , D. Pan , Genes Dev. 2012, 26, 1300; 22677547 10.1101/gad.192856.112 PMC3387657

[26] K. Uchino , R. Tateishi , S. Shiina , M. Kanda , R. Masuzaki , Y. Kondo , T. Goto , M. Omata , H. Yoshida , K. Koike , Cancer 2011, 117, 4475. 21437884 10.1002/cncr.25960

[27] K. Nakajima , D. H. Kho , T. Yanagawa , Y. Harazono , X. Gao , V. Hogan , A. Raz , Neoplasia 2014, 16, 939. 25425968 10.1016/j.neo.2014.09.005 PMC4240919

[28] A. Jung , S. Bisaz , H. Fleisch , Calcif. Tissue Res. 1973, 11, 269. 4350498 10.1007/BF02547227

[29] a) J. E. Brown , R. E. Coleman , Nat. Rev. Clin. Oncol. 2012, 9, 110; 22231759 10.1038/nrclinonc.2011.197

[30] G. K. Shah , D. N. Sang , M. S. Hughes , Retina 2009, 29, 133. 19202423 10.1097/IAE.0b013e3181960a28

[31] a) S. Burch , A. Bogaards , J. Siewerdsen , D. Moseley , A. Yee , J. Finkelstein , R. Weersink , B. C. Wilson , S. K. Bisland , J. Biomed. Opt. 2005, 10, 034011; 16229655 10.1117/1.1921887

[32] a) M. Esposito , T. Guise , Y. Kang , Cold Spring Harbor Perspect. Med. 2018, 8, a031252; 10.1101/cshperspect.a031252 PMC5980796 29101110

[33] X. Liu , Y. Zhang , Y. Chen , M. Li , F. Zhou , K. Li , H. Cao , M. Ni , Y. Liu , Z. Gu , K. E. Dickerson , S. Xie , G. C. Hon , Z. Xuan , M. Q. Zhang , Z. Shao , J. Xu , Cell 2017, 170, 1028. 28841410 10.1016/j.cell.2017.08.003 PMC6857456

