# RNF219/*α*‐Catenin/LGALS3 Axis Promotes Hepatocellular Carcinoma Bone Metastasis and Associated Skeletal Complications

**DOI:** 10.1002/advs.202102956

**Published:** 2021-08-18

**Authors:** Shuxia Zhang, Yingru Xu, Chan Xie, Liangliang Ren, Geyan Wu, Meisongzhu Yang, Xingui Wu, Miaoling Tang, Yameng Hu, Ziwen Li, Ruyuan Yu, Xinyi Liao, Shuang Mo, Jueheng Wu, Mengfeng Li, Erwei Song, Yanfei Qi, Libing Song, Jun Li

*Adv. Sci*. **2021**, *8*, 2001961

DOI: 10.1002/advs.202001961

In the originally published article, the wrong western blotting image of loading control *β*‐actin was mistakenly used in the upper right panel in HCCLM3‐BM4 cells in Figure S5A (Supporting Information). Please find the correct Figure S5a here:

**Figure S5:**
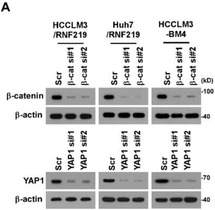
RNF219‐mediated *α*‐catenin reduction activates Wnt/*β*‐catenin and YAP1 pathways. (A) IB analysis of expression of *β*‐catenin and YAP1 in the indicated cells. *β*‐actin served as the loading control.

The authors apologize for any inconvenience this may have caused.

