# Supplementary material for: RNF219/α‐Catenin/LGALS3 Axis Promotes Hepatocellular Carcinoma Bone Metastasis and Associated Skeletal Complications
Source: Adv Sci (Weinh). 2020 Dec 31;8(4):2001961. doi: 10.1002/advs.202001961 (PMC7887580; doi:10.1002/advs.202001961)
Supplement: Supplementary file 1 — Supporting Information [file ADVS-8-2001961-s001.pdf]

WILEY-VCH

© 2020 Wiley-VCH GmbH

ADVANCED SCIENCE
Open Access

Supporting Information

for *Adv. Sci.,* DOI: 10.1002/advs.202001961

RNF219/α-catenin/LGALS3 axis Promotes Hepatocellular Carcinoma Bone Metastasis and Associated Skeletal Complications

*Shuxia Zhang, Yingru Xu, Chan Xie, Liangliang Ren, Geyan Wu, Meisongzhu Yang, Xingui Wu, Miaoling Tang, Yameng Hu, Ziwen Li, Ruyuan Yu, Xinyi Liao, Shuang Mo, Jueheng Wu, Mengfeng Li, Erwei Song, Yanfei Qi, Libing Song, Jun Li **

Supporting Information

RNF219/α-catenin/LGALS3 axis promotes hepatocellular carcinoma bone metastasis and associated skeletal complications

Shuxia Zhang, Yingru Xu, Chan Xie, Liangliang Ren, Geyan Wu, Meisongzhu Yang, Xingui Wu, Miaoling Tang, Yameng Hu, Ziwen Li, Ruyuan Yu, Xinyi Liao, Shuang Mo, Jueheng Wu, Mengfeng Li, Erwei Song, Yanfei Qi, Libing Song, Jun Li *

These authors contributed equally: Shuxia Zhang, Yingru Xu, Chan Xie

1. Supplementary Figures and Legends……………………………………………Page 2-16
2. Supplementary Materials and Methods………………………………………Page 17-26
3. Supplemental Tables…………………………………………………………..…Page 27-118
4. Abbreviations list……………..………………………………...……………..…Page 119-120

Supplementary Figures and Legends

Figure S1

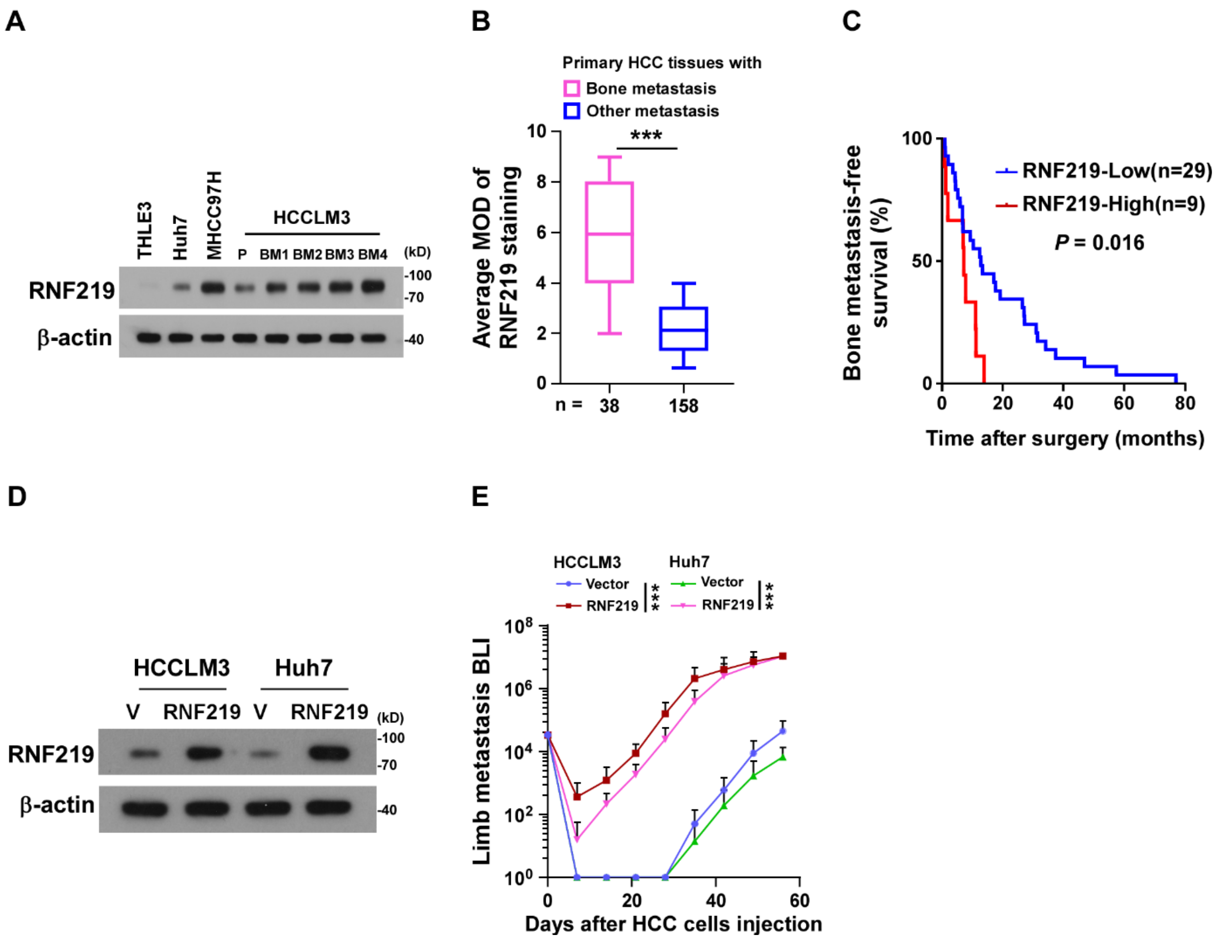

Figure S1. ***RNF219*** **overexpression promotes bone metastasis and SREs in HCC.** **(A)** IB analysis of RNF219 expression in immortalized normal liver epithelial cells THLE3, HCC cells (Huh7 and MHCC97H) and HCCLM3-derived cells (HCCLM3/BM1, HCCLM3/BM2, HCCLM3/BM3, HCCLM3/BM4). β-actin served as the loading control. **(B)** IHC statistical analysis of RNF219 expression in bone metastatic HCC tissues (n = 38) and other organ metastatic HCC tissues (n = 158). **(C)** Kaplan-Meier analysis of bone metastasis-free survival curves in HCC/BM with low vs high expression of RNF219 (n = 38; *P* = 0.016, log-rank test). **(D)** IB analysis of RNF219 expression in vector- and *RNF219*-transduced HCCLM3 and Huh7 cells. β-actin served as the loading control. **(E)** Normalized BLI signals of bone metastases in mice intracardially injected with the indicated cells from each

experimental group. Each error bar in panels **B, E** represents the mean ± SD of independent experiments. Significant differences were determined by Student's *t*-test (**B**). *** $P < 0.001$.

Figure S2

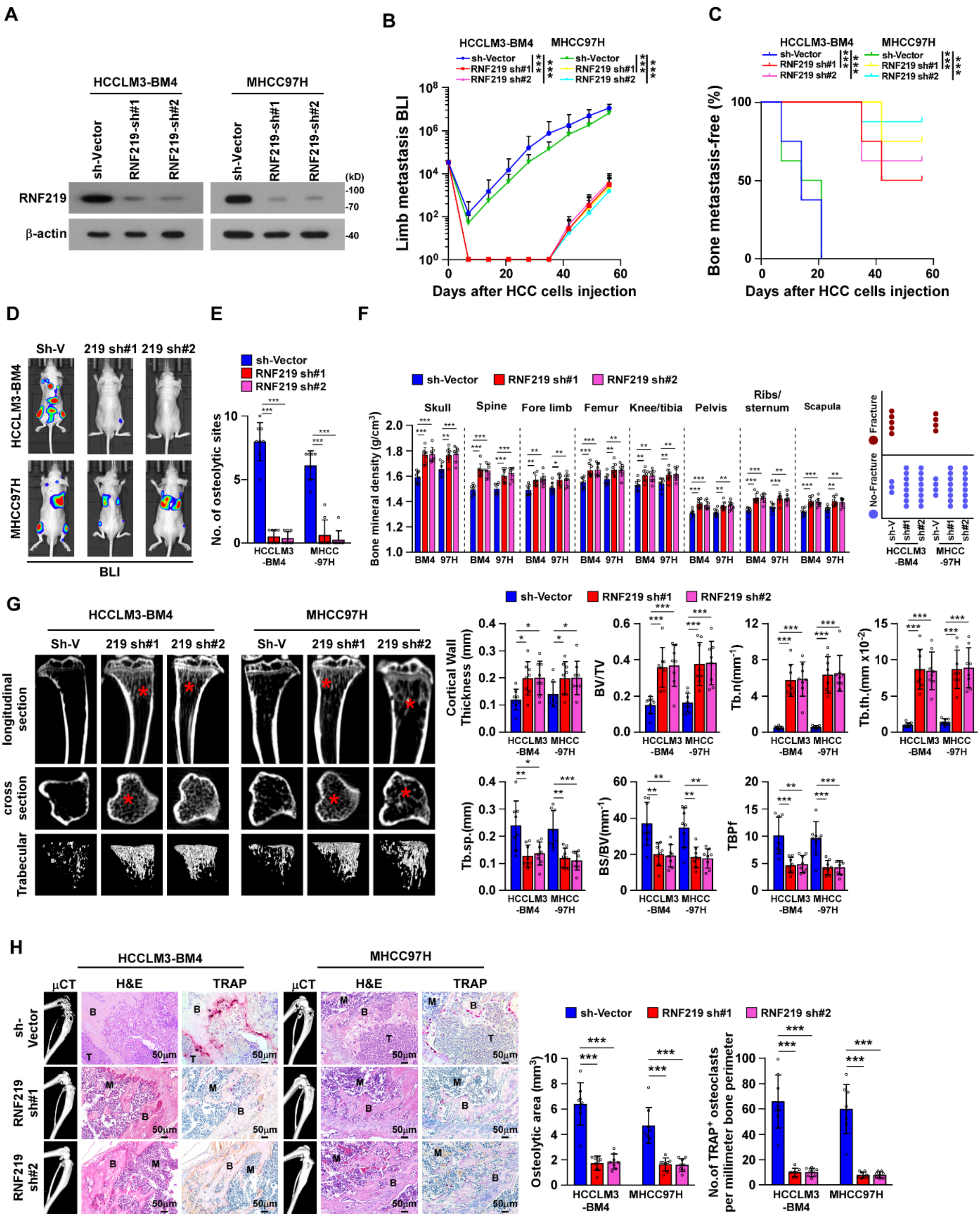

Figure S2. Downregulation of RNF219 inhibits bone metastasis and SREs in HCC.

(A) IB analysis of RNF219 expression in sh-vector- and *RNF219*-shRNA(s)-transduced HCCLM3/BM4 and MHCC97H cells. β-actin served as the loading control. **(B)**

Normalized BLI signals of bone metastases in mice intracardially injected with the indicated cells from each experimental group (n = 8/group). **(C)** Kaplan-Meier bone metastasis-free survival curve of mice from indicated experimental group (n = 8/group). **(D, E)** BLI images **(D)** and quantification **(E)** of osteolytic sites from representative mice (n = 8/group). **(F)** Quantification of BMD (left) and fracture frequency (right) of the indicated mice (n = 8/group) from experiments in **(D)**. **(G)** μCT images (left) and quantification (right) of bone parameters from representative mice. Left-upper, -middle and -lower: longitudinal, cross and trabecular section. Asterisks: trabecular structure (n = 8/group). BV/TV, bone volume/tissue volume ratio; BS/TV, bone surface/ tissue volume ratio; Tb. n, trabecular number; Tb. sp., trabecular separation; Tb. th., trabecular thickness; TBPf, trabecular bone pattern factor. **(H)** μCT and histological (H&E and TRAP) images (left) and quantification (right) of osteolytic area and $TRAP^+$-osteoclasts along the bone-tumor interface of metastases from representative mice. Scale bar, 50μm. Each error bar in panels **B** and **E-H** represents the mean ± SD of three independent experiments. Significant differences were determined by One-way ANOVA with Tukey's multiple comparison test (**B** and **E-H**). * $P < 0.05$, ** $P < 0.01$, *** $P < 0.001$.

Figure S3

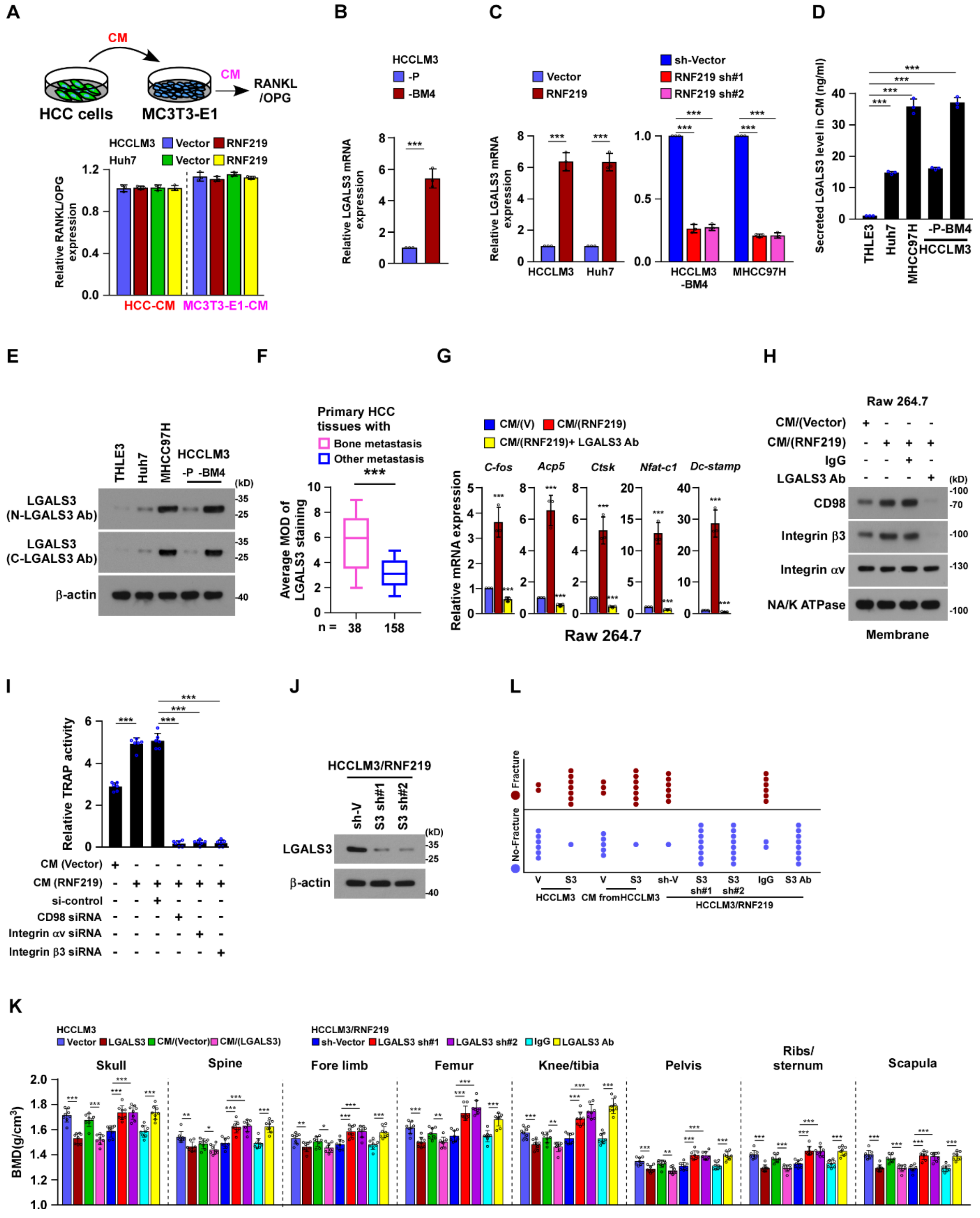

Figure S3. RNF219 induced-LGALS3 promotes osteoclastogenesis *in vitro*. (A) ELISA analysis of RANKL/OPG ratio in CM from osteoblast precursor cells MC3T3-E1 cells in the presence of CM from the indicated HCC cells. **(B, C)** Real-time PCR analysis of mRNA

level of LGALS3 in the indicated HCC cells. *GAPDH* serve as a loading control. **(D)** ELISA analysis of secreted LGALS3 protein level in CM from the indicated HCC cells. **(E)** IB analysis of secreted LGALS3 protein using N- and C- LGALS3 antibodies in CM from the indicated HCC cells. β-actin served as the loading control. **(F)** IHC statistical analysis of LGALS3 expression in bone metastatic HCC tissues (n = 38) and other organ metastatic HCC tissues (n = 158). **(G)** Real-time PCR analysis of mRNA level of osteoclast differentiation markers, including C-fos, Acp5, Ctsk, Nfat-c1 and Dc-stamp, in RAW264.7 cells cultured with CM-HCCLM3/vector, or CM-HCCLM3/RNF219, or CM-HCCLM3/RNF219 added with LGALS3 Ab. *GAPDH* serve as a loading control. **(H)** IB analysis of expression of CD98, integrin αv and integrin β3 in the cytoplasmic membrane fraction of RAW264.7 cells cultured with CM-HCCLM3/V, or CM-HCCLM3/RNF219, or CM-HCCLM3/RNF219 added with LGALS3 Ab. Na/K ATPase served as a loading control. **(I)** Analysis of TRAP activity of RAW264.7 cells as indicated treatments. **(J)** IB analysis of LGALS3 expression in sh-vector- and *LGALS3*-shRNA(s)-transduced HCCLM3/RNF219 cells. β-actin served as the loading control. **(K, L)** Quantification of BMD **(K)** and fracture frequency **(L)** of the indicated mice (n = 8/group) from experiments in Figure 3**C**. Each error bar in panels **A-D F, G. I and K** represents the mean ± SD of three independent experiments. Significant differences were determined by (**B, F**) and One-way ANOVA with Tukey's multiple comparison test (**A, D**, **G, I** and **K**). * $P < 0.05$, ** $P < 0.01$, *** $P < 0.001$.

Figure S4

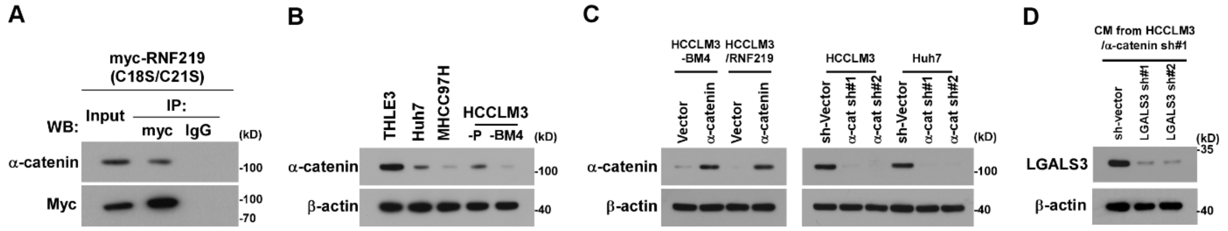

Figure S4. RNF219-mediated α-catenin proteasomal degradation induced LGALS3 expression and bone metastasis. **(A)** Co-IP using anti-myc-antibody and IB analyses of expression of α-catenin in RNF219 (C18S/C21S)-mu transfected HCCLM3 cells. **(B)** IB analysis of α-catenin expression in the indicated cells. β-actin served as a loading control. **(C)** IB analysis of α-catenin expression in the vector- or *α-catenin*-transduced cells (left) or sh-vector- or *α-catenin* shRNA(s)-transduced cells. β-actin served as a loading control. **(D)** IB analysis of LGALS3 expression in sh-vector- and *LGALS3*-shRNA(s)-transduced HCCLM3/α-catenin sh#1 cells. β-actin served as the loading control.

Figure S5

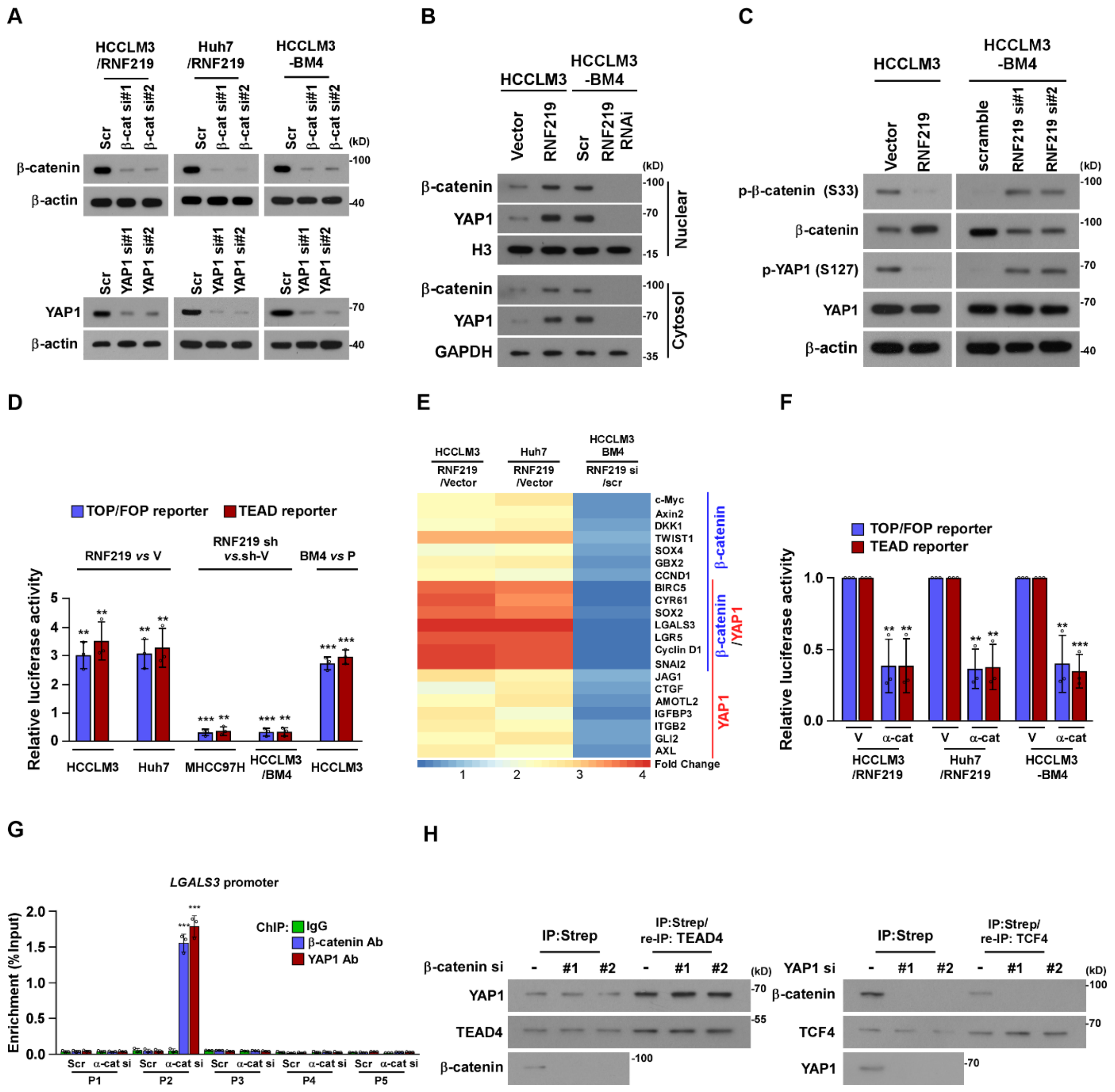

Figure S5. RNF219-mediated α-catenin reduction activates Wnt/β-catenin and YAP1 pathways. **(A)** IB analysis of expression of β-catenin and YAP1 in the indicated cells. β-actin served as the loading control. **(B)** IB analysis of expression of β-catenin and YAP1 in the nucleus (upper) and cytoplasm (lower) in the indicated cells. Histone (H3) was used as a nuclear loading control and GAPDH was used as a cytoplasmic loading control. **(C)** IB analysis of expression of phosphorylated-β-catenin (S33), total β-catenin, phosphorylated-YAP1 (S127), total YAP1 in the indicated cells. β-actin served as the

loading control. **(D)** Relative TOPflash /FOPflash (blue square) or TEAD (red square) luciferase reporter activity was analyzed in the indicated cells. **(E)** Real-time PCR analysis indicating an apparent overlap between β-catenin-regulated gene expression and YAP1-regulated gene expression in the indicated cells. The pseudocolor represents the intensity scale of RNF219 versus the vector, or RNF219 si versus scramble, generated by a log2 transformation. *GAPDH* serve as a loading control. **(F)** Relative TOPflash/FOPflash (blue square) or TEAD (red square) luciferase reporter activity was analyzed in the vector- or *α-catenin*-transduced cells. **(G)** ChIP-qPCR analysis of the enrichment of β-catenin and YAP1 on the promoter regions of *LGALS3* in the scramble- or α-catenin si-transfected cells. **(H)** Re-co-IP assay, using CAPTURE-approached proteins, analyses of YAP1/TEAD4 interaction in the scramble- or β-catenin si-transfected cell (left) or β-catenin/TCF4 interaction in the scramble- or YAP1 si-transfected cell (right). Each error bar in panels **D, F and G** represents the mean ± SD of three independent experiments. Significant differences were determined by Student's *t*-test (**D, F**) and One-way ANOVA with Tukey's multiple comparison test (**G**). ** $P < 0.01$, *** $P < 0.001$.

Figure S6

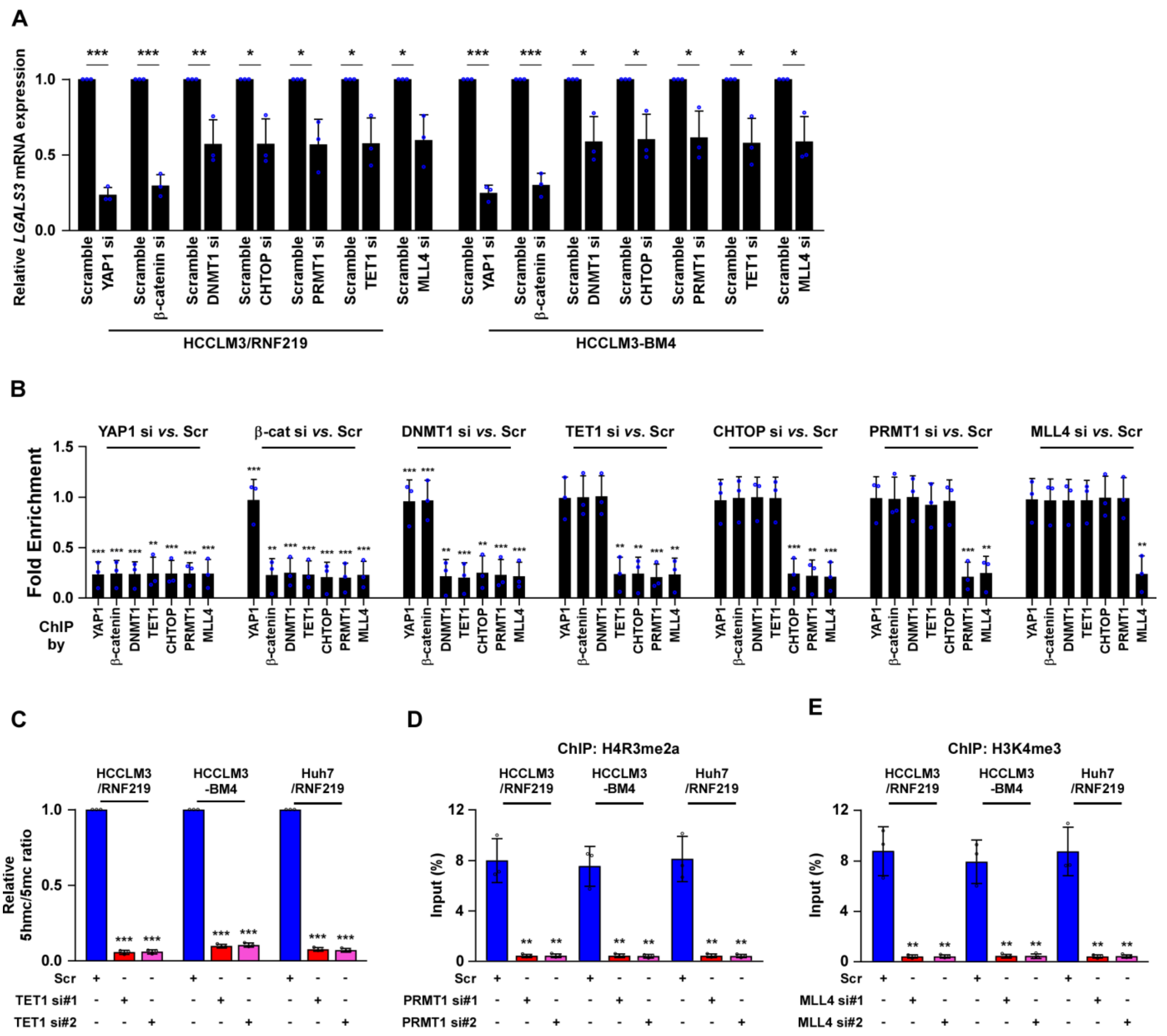

Figure S6. RNF219/α-catenin/YAP1/β-catenin axis induces a succession of epigenetic modifications at *LGALS3* promoter. **(A)** Relative mRNA expression of LGALS3, analyzed by real-time PCR assays, in YAP1 si-, or β-catenin si-, or DNMT1 si-, or CHTOP si-, or PRMT1 si-, or TET1 si-, or MLL4 si-transfected cells versus scramble-transfected cells. *GAPDH* serve as a loading control. **(B)** Relative enrichment of YAP1, or β-catenin, or DNMT1, or CHTOP, or PRMT1, or TET1, or MLL4 on the *LGALS3* promoter in the indicated gene silenced cells versus scramble-transfected cells analyzed by ChIP-qPCR assays. **(C)** Relative 5hmc/5mc ratio was examined in the scramble- and TET si(s)-transfected cells. **(D)** ChIP-qPCR analysis of the enrichment of H4R3me2a on the *LGALS3* promoter in the

scramble- or PRMT1 si-transfected cells. **(E)** ChIP-qPCR analysis of the enrichment of H3K4me3 on the *LGALS3* promoter in the scramble- or MLL4 si-transfected cells. Each error bar in panels **A-E** represents the mean ± SD of three independent experiments. Significant differences were determined by Student's *t*-test (**A, B**) and One-way ANOVA with Tukey's multiple comparison test (**C-E**). * $P < 0.05$, ** $P < 0.01$, *** $P < 0.001$.

Figure S7

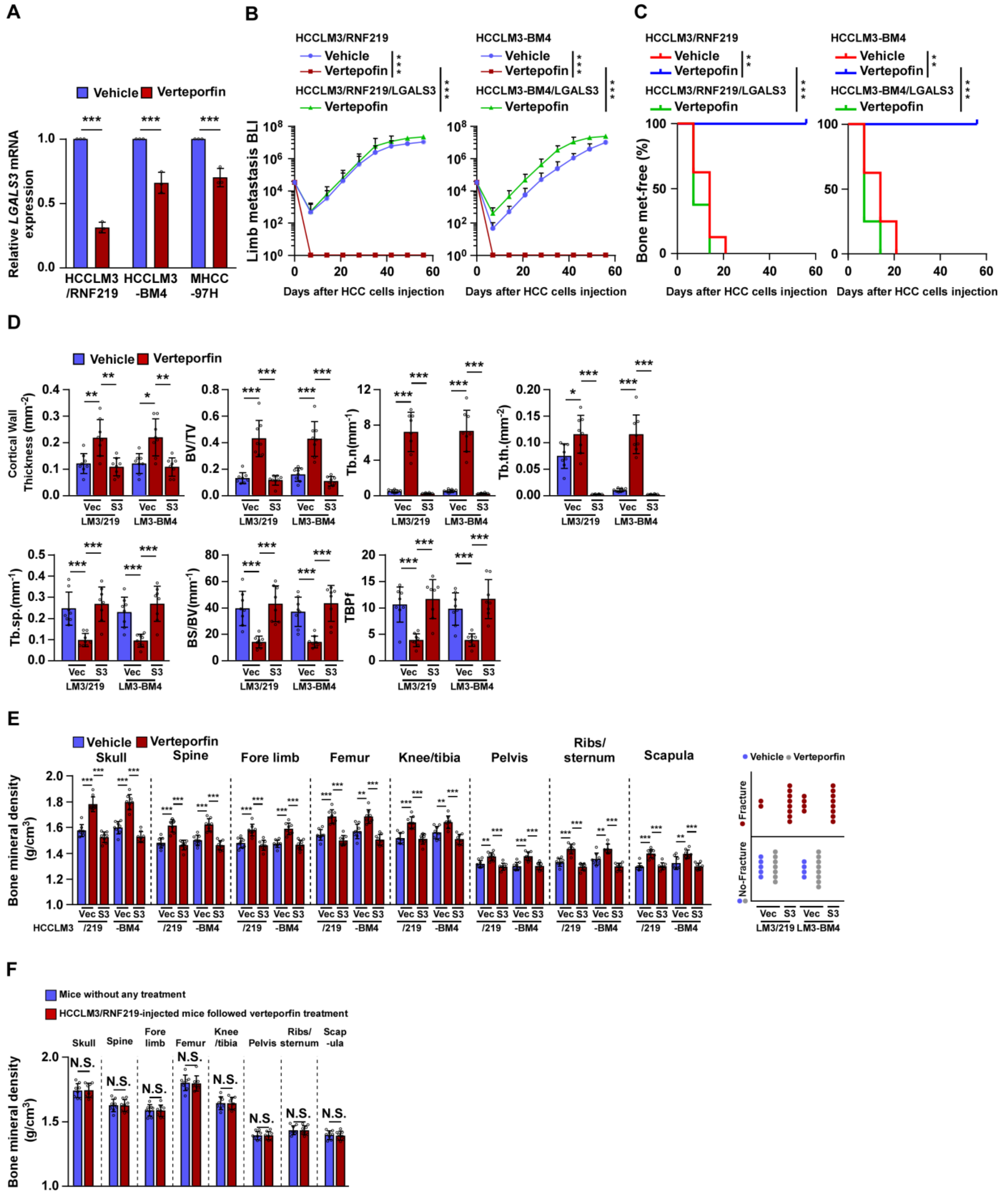

Figure S7. Verteporfin treatment reduces LGALS3 expression and suppresses HCC bone-metastasis and SREs. **(A)** Real-time PCR analysis of mRNA level of LGALS3 in the vehicle- or Verteporfin (10μM)-treated HCC cells. *GAPDH* serve as a loading control. **(B-C)** Normalized BLI signals of bone metastases (**B**) and Kaplan-Meier bone metastasis-free

survival curve (**C**) of mice from experiments in Figure 8**A** (n = 8/group). **(D-F)** Quantification of bone parameters analyzed by μCT assay **(D)** and BMD **(E-F)** and fracture frequency of mice **(E)** in the representative mice from experiment in Figure 8**A**. Each error bar in panels **A-B and D-F** represents the mean ± SD of three independent experiments. Significant differences were determined by Student's *t*-test (**A and D-F**). * $P < 0.05$, ** $P < 0.01$, *** $P < 0.001$ and N.S.: not significant ($P > 0.05$).

Figure S8

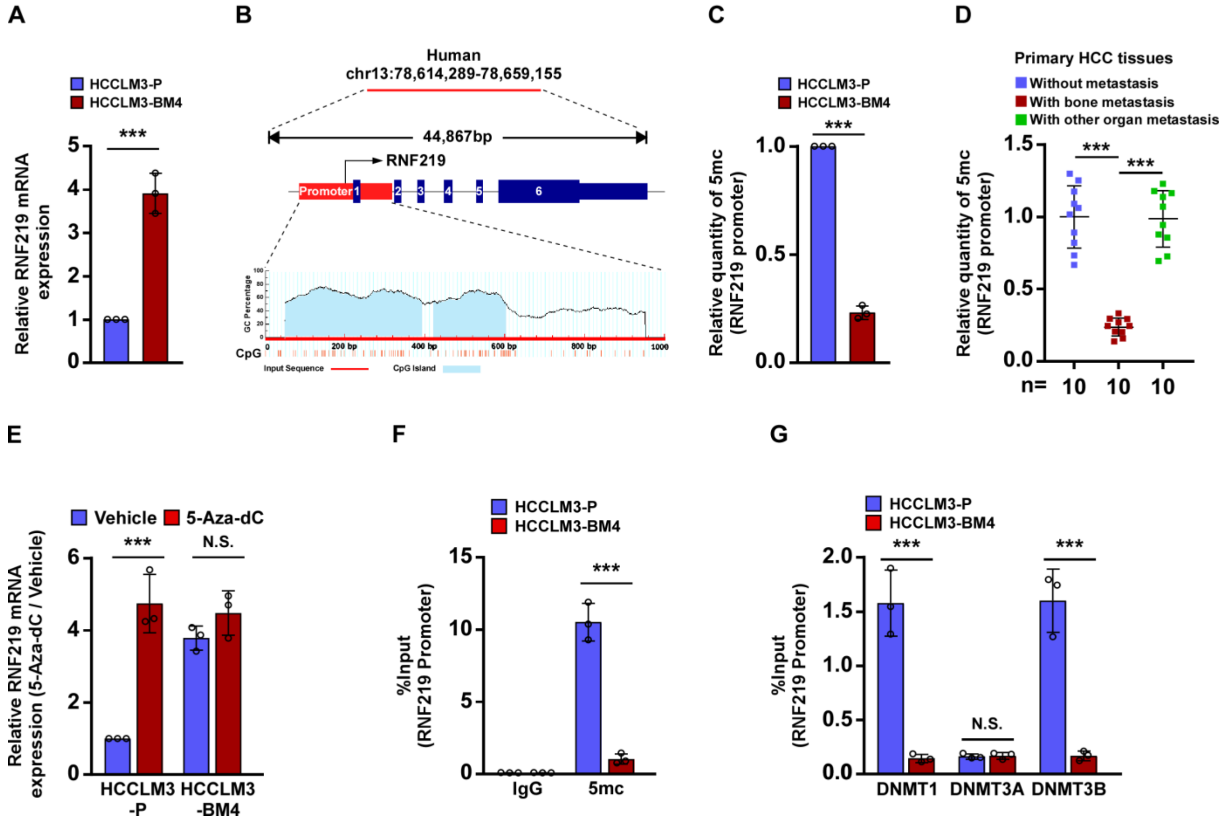

Figure S8. Promoter hypomethylation-mediated RNF219 upregulation in bone metastatic HCC cells. (A) Real-time PCR analysis of mRNA level of RNF219 in the HCCLM3-BM4 or HCCLM3-P cells. *GAPDH* serve as a loading control. **(B)** Schematic illustration of the predicted RNF219 promoter region contains high frequency of CpG sites. **(C-D)** Relative 5-methylcytosine (5mc) level was examined in RNF219 promoter in the indicated HCC cells (C) and indicated HCC tissues (n = 10/group). **(E)** Real-time PCR analysis of mRNA level of RNF219 in vehicle- or 5-Aza-dC treated HCC cells. *GAPDH* serve as a loading control. **(F, G)** ChIP-qPCR analysis of the enrichment of 5mc **(F)** and DNMTs, including DNMT1, DNMT3A and DNMT3B **(G)**, on the *RNF219* promoter in the indicated HCC cells. Each error bar in panels **A, and C-G** represents the mean ± SD of three independent experiments. Significant differences were determined by Student's *t*-test (**A, C**) and One-way ANOVA with Tukey's multiple comparison test (**D-G**). ** $P < 0.01$, *** $P < 0.001$ and N.S.: not significant ($P > 0.05$).

Figure S9

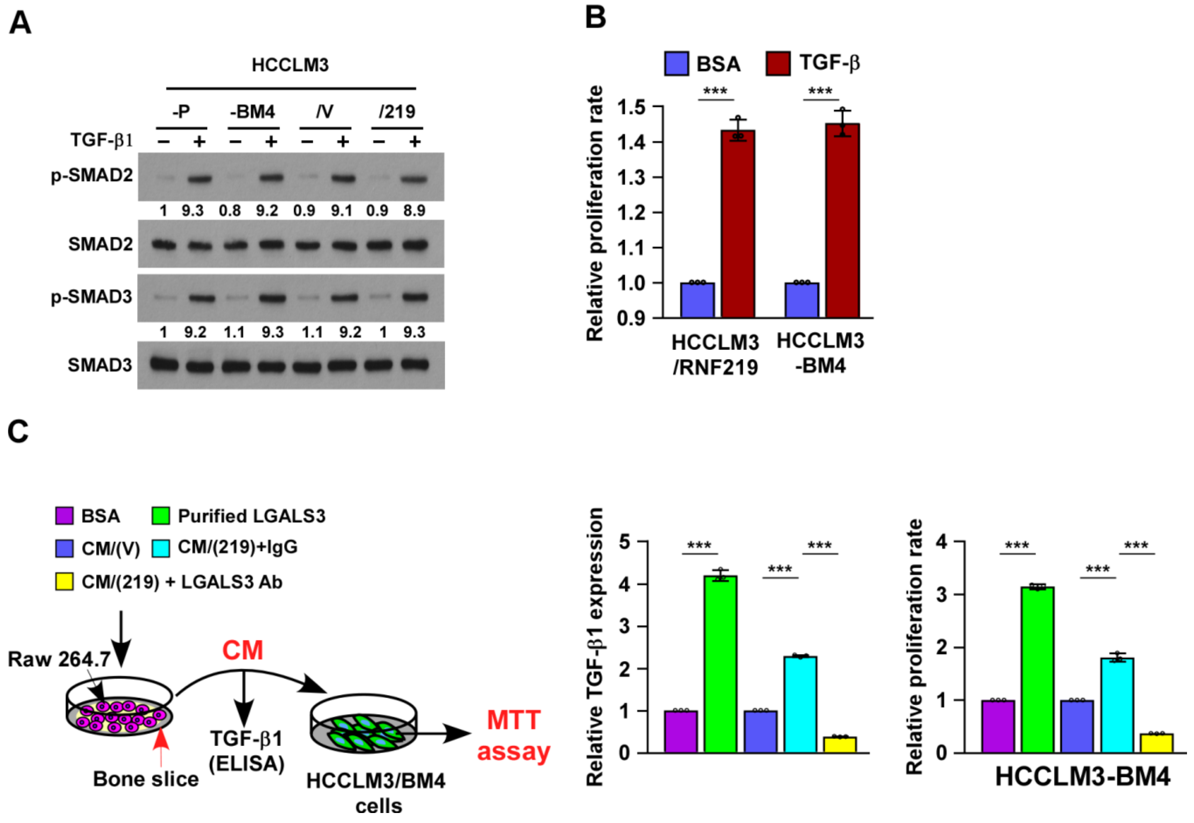

Figure S9. TGF-β signaling contributes to HCC-BM by inducing osteolytic vicious cycle. **(A)** IB analysis of the expression and phosphorylation level of SMAD2 and SMAD3 in the indicated cells treated with or without TGF-β (50 pM). **(B)** MTT assay analysis of proliferation rate of indicated cells treated with or without TGF-beta (50pM) in DMEM (1.0% FBS). **(C)** Left: Schematic illustration of LGALS3-induced “vicious cycle” between cancer cells and osteoclasts. Middle: ELISA analysis of TGF-β1 levels in CM from RAW 264.7 cells cultured onto the bone slice in the presence of indicated CM from HCC cells, or BSA, or purified LGALS3. Right：MTT assay analysis of proliferation rate of indicated cells from experiment in left panel. Each error bar in panels **B and C** represents the mean ± SD of independent experiments. Significant differences were determined by Student’s *t*-test (**B**) and One-way ANOVA with Tukey’s multiple comparison test (**C**). *** $P < 0.001$.

Supplementary Materials and Methods

Cell lines. The HCCLM3, Huh7 and MHCC97H HCC cell lines, were obtained from the Sun Yat-sen University Cancer Center mouse osteoclast. Precursors Raw 264.7 cells and Human embryonic kidney cells (HEK) 293FT cells were purchased from American Type Culture Collection (ATCC, Manassas, VA). All the cell lines were grown in Dulbecco's modified Eagle's medium (DMEM) (Gibco, Grand Island, NY) supplemented with 10% fetal bovine serum (Gibco, Grand Island, NY). All the cell lines were tested for mycoplasma contamination and were authenticated by short tandem repeat (STR) fingerprinting at Medicine Lab of Forensic Medicine Department of Sun Yat-Sen University (China).

Establishment of the HCC bone metastasis cell line. To establish HCC bone metastasis cell line, the HCCLM3-parental cells that stably expressed firefly luciferase reporter were inoculated into the left cardiac ventricle of immunodeficient mice, and the formed bone metastatic tumors were monitored by bioluminescence signal. Then bone metastatic HCCLM3 cells (named as HCCLM3-BM1) were recovered from BLI-suspected bone sites, expanded in culture, and re-injected intracardially into mice for the next round. Total 4 round was performed.

To isolate cells from bone metastatic tumors, mice were euthanized, and bones from BLI-suspected sites were resected from the body at the joints with the surrounding soft tissue removed. Excised bone was minced into tissue volumes of 1 mm^3 and incubated in RBC lysis buffer followed by collagenase/hyaluronidase (Sigma-Aldrich, MO, USA) digesting solution on a rocking plate. Cells were collected via centrifugation and seeded onto a 12-well plate. Culturing medium was replaced after 24 h to eliminate non-adherent cells. Cells were maintained in 10 % FBS DMEM.

Plasmids, retroviral infection and transfection. The human RNF219, LGALS3, α-catenin

and truncated-α-catenin fragments were cloned into pSin-EF2 vector. The RNF219-mu (C18S/C21S) was created using primers and a Stratagene mutagenesis kit according to the protocol recommended by the manufacturer (Stratagene, San Diego, CA, USA). ShRNAs targeting RNF219, α-catenin, LGALS3 and were cloned into the pSuper retro viral vector. The TCF/LEF1 reporter plasmids contain wild-type (CCTTTGATC; TOP flash) or mutated (CCTTTGGCC and FOP flash) binding site and TEAD reporter plasmid containing wild-type (ACATTCCA) TEAD binding sites). All primers and oligonucleotides used in plasmid construction are listed in Supplementary Table9. Transfection of short interfering RNAs (siRNAs) or plasmids was performed using the Lipofectamine 3000 reagent (Thermo Fisher Scientific, Waltham, MA, USA) according to the manufacturer's instructions. Stable cell lines expressing RNF219 or LGALS3 and RNF219 or LGALS3 shRNA(s) were generated via retroviral infection and selected for 10 days with 0.5 μg/mL puromycin 48 h after infection.

RNA extraction, reverse transcription and real-time PCR. Total RNA was extracted from the indicated cell using the Trizol (Life Technologies, Carlsbad, CA, USA) reagent according to the manufacturer's instruction. Real-time reverse transcription-polymerase chain reaction (PCR) primers and probes were designed with the assistance of the Primer Express v 2.0 software (Applied BioSystems, Foster City, CA, USA). Expression data were normalized to the geometric mean of housekeeping gene *GAPDH* to control the variability in expression levels and calculated as $2^{-[(C_t \text{ of gene}) - (C_t \text{ of } GAPDH)]}$, where C_t represents the cycle threshold for each transcript. All primers are listed in Supplementary Table9.

Chemical reagents. Verteporfin (VP, S1786) was purchased from Selleck Chemicals (Houston, TX, USA). LGALS3 antagonist (GCS-100, also known as MCP) was purchased from La Jolla Pharmaceutical Company (San Diego, CA, USA). Human recombinant α-catenin protein (ab51443; Cambridge, MA, USA), recombinant human TGF beta 1 protein

(Active) (ab50036) and 5-Aza-2'-deoxycytidine (Decitabine) (ab120842) were purchased from Abcam. Sucrose, Lactose, Tecfidera and MG132, 3-MA (3-methyladenine), clasto-lactacystin β-lactone(cLL), leupeptin, and NH4Cl were purchased from Sigma Aldrich (St. Louis, MO, USA).

Far-Western analysis. Far-Immunoblotting were performed by using the proteins immunoprecipitated by anti-RNF219 antibody and human recombinant α-catenin protein. Briefly, the proteins were separated by SDS-PAGE, and were transferred onto a PVDF membrane. Membranes were then preincubated in 10% skimmed milk for 1 hour at 4°C. As indicated, recombinant α-catenin protein was added at 5μg/mL and incubated at 4°C for 18 hours. After extensive washing six times with TBST, the membrane was subjected to immunoblotting analysis using indicated antibodies.

Co-immunoprecipitation (Co-IP) assay. Cells grown in 100-mm culture dishes were lysed using 500 μL of lysis buffer [25 mmol/L HEPES (pH 7.4), 150 mmol/L NaCl, 1% NP-40, 1 mmol/L EDTA, 2% glycerol, and 1 mmol/L phenylmethylsulfonyl fluoride (PMSF)]. After being maintained on ice for 30 minutes, the lysates were clarified by microcentrifugation at 12,000 rpm for 10 minutes. To preclear the supernatants, the lysates were incubated with 20 mL of agarose beads (Calbiochem, San Diego, CA, USA) for 1 hour with rotation at 4°C. After centrifugation at 2,000 rpm for 1 minutes, the supernatants were incubated with 20 mL of antibody-cross-linked protein G-agarose beads overnight at 4°C. The agarose beads were then washed six times with wash buffer [25 mmol/L HEPES (pH 7.4), 150 mmol/L NaCl, 0.5% NP-40, 1 mmol/L EDTA, 2% glycerol, 1 mmol/L PMSF]. After removing all the liquid, the pelleted beads were resuspended in 30 mL of 1 M glycine (pH 3), after which, 10 μL 4 × sample buffer was added, the samples were denatured, and the sample components were electrophoretically separated on SDS-PAGE for immunoblot analysis.

Luciferase assay. The cells (1 × 10^3) were seeded in triplicate in 48-well plates and allowed to settle for 24 h. One hundred nanograms of luciferase reporter plasmids or the control-luciferase plasmid, plus 5 ng of pRL-TK renilla plasmid (Promega, Madison, WI), were transfected into indicated cells using the Lipofectamine 3000 reagent (Invitrogen, Carlsbad, CA, USA) according to the manufacturer's recommendation. Luciferase and renilla signals were measured at 48 h after transfection using the Dual Luciferase Reporter Assay Kit (Promega, Madison, WI) according to a protocol provided by the manufacturer.

Chromatin Immunoprecipitation (ChIP) assay. The entire procedure was performed with the chromatin immunoprecipitation (ChIPs) assay kit (Cell Signaling Technology, Danvers, MA, USA) according to the manufacturer's instructions. Briefly, indicated cells were grown to 70~80% confluence on 100-mm culture dish and were fixed with 1% formaldehyde to cross-link proteins to DNA. The cell lysates were sonicated to shear DNA into small uniform fragments. Equal aliquots of chromatin supernatants were then immunoprecipitated overnight at 4°C using anti-YAP1 (Cell Signaling Technology, Danvers, MA, USA (CST) #14074), anti-β-catenin (CST#8480), anti-DNMT1 (Abcam; ab13537), anti-CHTOP (CST#2895), anti-PRMT1 (ab3768), anti-TET1 (GeneTex Inc., Irvine, CA, USA; GTX124207), anti-MLL4 (Novus Biologicals, Centennial, CO, USA; NB600-254), anti-α-catenin (ab47105), anti-APC (CST# 2504), anti-LSD1 (ab17721), anti-H3K4me3 (ab12209), anti-H4R3me1(Diagenode, Valparaiso, Chile; C15410299), anti-H4R3me2a (Active Motif, Carlsbad, CA, USA; Cat.39705), anti-H3R2me1 (ab15584), anti-H3R2me2s (ab194684), anti-DNMT1 (#ab87656), anti-DNMT3A (#ab2850), anti-DNMT3B (#ab13604), anti-5mc (#15200081; Diagenode), anti-H3R8me2s (Epigentek, Farmingdale, NY, USA; A-3706-050), and anti-IgG antibodies (Millipore, Billerica, MA) with protein G magnetic beads. The cross-linked protein/DNA complexes were collected by magnetic pull down, and then were eluted from beads by elution buffer. After reverse cross-link of protein/DNA

complexes to free DNA, PCR was performed using specific primers. All ChIP primers are listed as Supplementary Table9.

Plasma Membrane Protein Extraction. A plasma membrane protein extraction kit (ab65400; Abcam, Cambridge, MA, USA) was used to extract the plasma membrane protein according to the manufacturer's instructions.

Mean optical density (MOD) analysis. IHC staining for protein expression of RNF219 and LGALS3 in HCC samples with bone or other organ metastasis was quantitatively analyzed by using the AxioVision 4.6 computerized image analysis system assisted with an automatic measurement program (Carl Zeiss). The method of mean optical density (MOD) was used to determine the immunostaining intensity of each tested specimen. Briefly, the stained sections were evaluated at ×200 magnification, and 10 representative staining fields of each section were analyzed to verify the MOD, which represents the strength of staining signals as measured per positive pixels. The MOD data were statistically analyzed by using the t test to compare the average MOD difference between different groups of tissues, and $P < 0.05$ was considered significant.

Immunoblotting analysis (IB). IB was performed according to a standard protocol with the following antibodies: anti-integrin αv (AF1219) and anti-integrin β3 (AF2266) antibodies were purchased from BD company (Franklin lakes, NJ, USA); anti-c-SRC (ab16885), anti-p-SYK (ab58575), anti-SYK (ab40781), anti-p-VAV-3 (ab192590), anti-VAV-3 (ab203315), anti-RAC1(ab155938), anti-RNF219 (ab91464), anti-CD98 (ab2528), anti-H3 (ab1791), anti-p-YAP1(S127) (ab76252), anti-TCF4 (ab76151), anti-N-LGALS3(ab2785), anti-C-LGALS3(ab53082), anti-TEAD4 (ab58310), anti-Na^+/K^+-ATPase (ab58475), anti-APC (ab40778), anti-LSD1 (ab17721), anti-PRMT1(ab3768), anti-DNMT1(ab13537) and anti-CHTOP (ab222861) antibodies were purchased from Abcam (Cambridge, MA).

Anti-YAP1 (CST#14074), anti-β-catenin (CST#8480) and anti-β-actin (CST#3700), anti-p-SRC (CST#2101), anti-GAPDH (CST#97166), anti-α-tubulin (CST#2144) anti-Smad2 (CST#3122S) , anti-p-Smad2 (CST#18338), anti-Smad3 (CST#9523S) and anti-p-Smad3 (CST#9520S) antibodies were purchased from Cell Signaling technology (Danvers, MA). Anti-MLL4 (NB600-254) antibody was purchased from Novus Biologicals (Centennial, Colorado, USA). Anti-TET1 antibody (GTX124207) was purchased from GeneTex (San Diego, CA). Anti-α-catenin antibody (C2081), anti-β-catenin (pS33) (C2363), anti-Flag (F3165) and anti-HA (SAB1306169) antibodies purchased from Sigma-Aldrich (St. Louis, MO).

Immunofluorescence (IF) staining. The osteoclast precursors, Raw 264.7 cells were placed in 24-well clusters containing glass coverslips (Thermo Fisher Scientific) for 24 h and were treated with BSA or purified FLAG-tagged LGALS3 protein (100ng/ml) or conditioned media (CM) from HCC cells for 6 days. Cells were rinsed briefly with PBS and fixed in 4% (w/v) paraformaldehyde in PBS for 20 min at 37 °C. Aspirate fixation solution and wash cells 2-3 times in PBS. And followed by the antibody: anti-LGALS3 (ab2785, 1:200) antibody, anti-FLAG (Sigma MAB3118, 1:100) antibody, anti-CD98 (ab2528, 1:200) antibody, anti-αvβ3 (ab78289, 1: 500) antibody. The secondary antibody was goat anti-rabbit IgG (H + L) conjugated with Alexa Fluor 488 (Thermo Fisher Scientific), or added 1:1000 dilution of Phalloidin-iFluor 594 (ab176757) in 1% BSA at room temperature for 20-90 minutes. Rinse cells 2-3 times with PBS (5 min/wash). Then cells were mounted with Antifade Mountant with DAPI (Thermo Fisher Scientific). Observe the cells at Ex/Em 493/517 nm and the images were captured using the AxioVision Rel.4.6 computerized image analysis system (Carl Zeiss, Jena, Germany).

Enzyme-linked immunosorbent assay (ELISA). Serum samples were kept at room

temperature for about 1.0 h to defrost completely before assays. The levels of mouse serum LGALS3 in supernatants were measured using a mouse LGALS3 enzyme-linked immunosorbent assay kits (ab203369, Abcam, Cambridge, MA.). The LGALS3 level in human serum and the culture medium from HCC cells were measured using a human LGALS3 enzyme-linked immunosorbent assay kits (ab188394, Abcam, Cambridge, MA.) and analyzed according to the manufacturer's instructions. The levels of TGF-β, RANKL and OPG in culture medium were measured using mouse TGF-β ELISA Kit (ab119557), human RANKL ELISA Kit (ab213841), mouse RANKL ELISA Kit (ab100749), human OPG ELISA Kit (ab100617) and mouse OPG ELISA Kit (ab203365), respectively. Data were read with the SpectraMax i3x Multi-Mode Microplate Reader (Molecular Devices) at 450 nm, the concentrations of LGALS3 in the samples were determined by extrapolating from the standard curve created by plotting the absorbance of the standards versus corresponding concentrations.

Cell growth assay. Cell growth was examined by 3-(4, 5-Dimethylthiazol-2-yl)-2, 5-diphenyltetrazolium bromide (MTT) assay. Briefly, cells were seeded at the density of 8000 cells/well into 96-well plate. At the time of assay, 0.5 mg/mL MTT in basic medium was added to each well and incubated for 4 h. After removing MTT, dimethyl sulfoxide (DMSO) was added and mixed vigorously. Absorbance was measured at 490 nm using the SpectraMax i3x Multi-Mode Microplate Reader (Molecular Devices).

Osteoclastogenesis assay. The RAW264.7 cells (1×10^5) were cultured on 24-well clusters containing glass coverslips (Thermo Fisher Scientific) and grown in the conditioned media (CM) from indicated HCC cells treated with DMSO or Verteporfin (10 μM), or in medium containing purified LGALS3 protein (100 ng/ml) or BSA. Media were changed every other day. Osteoclasts were counted on day 6. The osteoclasts cultured on plastic dishes were fixed

with 4% paraformaldehyde/PBS, and TRAP expression was examined by staining with a commercial kit (387A-1KT; Sigma-Aldrich). Osteoclasts were defined as TRAP-positive multinucleated cells containing more than 3 nuclei.

Bone resorption pit assay. To study the ability of osteoclasts to form resorption pits on bone slices (IDS PLC, catalog number: DT-1BON1000-96), RAW264.7 cells (1×10^5) were seeded onto the bone slices. Then cells were cultured for 9 days in either conditioned media (CM) from HCC cells with indicated treatment or in medium containing 100 ng/ml LGALS3 protein or BSA. Media were changed every other day. After 9 days, bone slices were fixed with 2.5% glutaraldehyde, followed by the removal of cells by mechanical agitation and sonication. Resorption lacunae were visualized by scanning electron microscopy (SEM). Three fields were randomly selected for each bone slice for further analysis. Pit areas were quantified using ImageJ software.

Xenografted tumor models. All of the animal procedures were approved by the Sun Yat-sen University Animal Care Committee. Intracardiac injections of luciferase-expressing HCC cells (1×10^5) were conducted in nu/nu nude mice (5 weeks old) for bone metastasis assays. Before cancer cell injection, mice were educated for 2 weeks by intraperitoneal injection of 300 μl of CM from indicated cells. Anti-LGALS3 antibody (abcam) or anti-rabbit IgG was administered intraperitoneally twice a week at 1 mg/kg, 2 days after implantation. Verteporfin (20 mg/kg) was injected intraperitoneally twice a week. The formation of bone metastases was observed and assessed weekly in mice injected with D-luciferin (75 mg/kg) by bioluminescence imaging using the IVIS Spectrum *In Vivo* Imager. At week 5, the osteolytic lesions in mice were observed using SIEMENS micro-CT system (Inveon). The bone of mice were harvested and prepared for further analyses.

Micro-CT (μCT) analysis. The hind limbs were removed from euthanized mice and fixed in

either 4% paraformaldehyde solution or periodate-lysine-paraformaldehyde fixative. Fixed hind limbs were dissected free of tissue and scanned on a micro-CT scanner (SIEMENS, Munich, Germany). A standard trabecular volume of interest was chosen starting 0.1 mm from the growth plate and included all trabeculae in a 1 mm^3 region of bone. Trabecular volume and number were assessed in this region. Osteolytic lesions were measured through 360° view of the bone on a three-dimensional model in a 3 mm length of cortical bone, starting at the growth plate.

***In vivo* quantification of osteoclast number.** Hind limbs were fixed in paraformaldehyde solution (4%), decalcified in 14.3% EDTA for 4 days at 37 ^oC with daily changes of EDTA, then embedded in paraffin wax. Sections were used by H&E stained with Mayer's hematoxylin solution, or IHC stained using antibodies of RNF219 (ab224493), or stained with TRAP (a TRAP kit, 387A-1KT; Sigma-Aldrich) according to manufacturer's protocols. The numbers of $TRAP^+$-osteoclasts were determined on a 3 mm length of endocortical surface and viewed on an optical microscope (Olympus, DP72, Tokyo, Japan).

Alkaline phosphatase (ALP) staining. Fourteen days after osteogenic induction, a TRAP/ALP Stain Kit (Wako, Richmond, VA, USA; 294-67001) was used to perform alkaline phosphatase (ALP) staining. The cells cultured in 24-well plates were rinsed three times by PBS and fixated for 30 min using pre-cold fixative. Next, ALP substrate solution was added and cells were cultivated (room temperature, 15-45min) in a darkroom. After that, cells with ddH2O were rinsed and filmed under an optical microscope. The absorbance at 405 nm of each well was measured with a microplate reader according to the manufacturer's instruction.

Preparation of nuclear and cytosol extracts. Nuclear and cytosol fractions were isolated by using NE-PER™ Nuclear and Cytoplasmic Extraction Reagents (Thermo Fisher Scientific, Waltham, MA) according to the manufacturer's instructions. The indicated Cells were

treated with 0.25% trypsin-EDTA and subsequently harvested at centrifuge at 500 × g for 5 minutes. The cell pellet was washed 2-3 times with PBS (pH7.4) and then transferred to a 1.5mL microcentrifuge tube and centrifuged at 500 × g for 2-3 minutes. The cell pellet was resuspended in 200µl ice-cold CER I and vortexed the tube vigorously and incubated on ice for 10 min. The cells were added 11 µL ice-cold CER II to the tube, vortexed for 5 seconds and centrifuged for 5 minutes at 16,000 × g. The supernatant (cytoplasmic extract) was immediately transfer to a clean pre-chilled tube. The pelleted nuclei were resuspended in 100µl ice-cold NER, which contains nuclei and vortexed for seconds every 10 minutes, for a total of 40 minutes. The supernatant (nuclear extract) fraction was then centrifuged at ~16,000 × g for 10 minutes and then immediately transferred the to a clean pre-chilled tube. Store extracts at -80°C until use.

Purification of LGALS3 Protein. The plasmid encoding FLAG-tagged LGALS3 was transiently transfected into HCCLM3 cells (1×10^8) and cultured for 2 days. The cell cultured media were harvested and centrifuged at 2,000 x g for 5 min at 4 °C, and the supernatant was transfer into an Amicon centrifuge filter unit and spined at 4,000 × *g* for 30-40 min. The concentrated conditioned media was incubated with FLAG-M2 beads for more than 2.0 h while rotating at 4 °C. The beads were washed with cold wash buffer for 4 times by spinning at 100 × *g* for 1 min. After the last wash, syringe needles were used to remove as much buffer as possible with minimal disruption of the beads. Then the beads were incubated with elution buffer for 1 h at room temperature and collected the purified FLAG-tagged LGALS3 after centrifugation for quantification.

Supplemental Tables

Table S1. Incidence of metastasis (Number of metastasis mice/ total mice)

	HCCLM3-Parental (n=8)		HCCLM3/RNF219 (n=8)		Huh7 (n=8)		Huh7/RNF219 (n=8)	
**Days**	**Bone Metastasis**	**Other Metastasis**	**Bone Metastasis**	**Other Metastasis**	**Bone Metastasis**	**Other Metastasis**	**Bone Metastasis**	**Other Metastasis**
**7**	0/8	0/8	4/8	0/8	0/8	0/8	1/8	0/8
**14**	0/8	0/8	6/8	0/8	0/8	0/8	8/8	0/8
**21**	0/8	0/8	8/8	0/8	0/8	0/8	8/8	0/8
**28**	0/8	0/8	8/8	0/8	0/8	0/8	8/8	0/8
**35**	2/8	3/8	8/8	2/8	1/8	2/8	8/8	3/8
**42**	5/8	4/8	8/8	2/8	4/8	3/8	8/8	3/8
**49**	5/8	5/8	8/8	4/8	5/8	4/8	8/8	4/8
**56**	8/8	5/8	8/8	5/8	5/8	5/8	8/8	4/8

	HCCLM3-BM4 (n=8)		HCCLM3-BM4-RNF219 sh#1(n=8)		HCCLM3-BM4-RNF219 sh#2(n=8)	
**Days**	**Bone Metastasis**	**Other Metastasis**	**Bone Metastasis**	**Other Metastasis**	**Bone Metastasis**	**Other Metastasis**
**7**	2/8	0/8	0/8	0/8	0/8	0/8
**14**	5/8	0/8	0/8	0/8	0/8	0/8
**21**	8/8	0/8	0/8	0/8	0/8	0/8
**28**	8/8	0/8	0/8	0/8	0/8	0/8
**35**	8/8	0/8	0/8	0/8	0/8	0/8
**42**	8/8	0/8	2/8	0/8	3/8	0/8
**49**	8/8	0/8	4/8	0/8	3/8	1/8
**56**	8/8	0/8	4/8	1/8	3/8	1/8

	MHCC97H (n=8)		MHCC97H-RNF219 sh#1(n=8)		MHCC97H-RNF219 sh#2(n=8)	
**Days**	**Bone Metastasis**	**Other Metastasis**	**Bone Metastasis**	**Other Metastasis**	**Bone Metastasis**	**Other Metastasis**
**7**	3/8	2/8	0/8	2/8	0/8	1/8
**14**	4/8	4/8	0/8	3/8	0/8	4/8
**21**	8/8	5/8	0/8	5/8	0/8	6/8
**28**	8/8	8/8	0/8	8/8	0/8	8/8
**35**	8/8	8/8	0/8	8/8	0/8	8/8
**42**	8/8	8/8	2/8	8/8	1/8	8/8
**49**	8/8	8/8	2/8	8/8	1/8	8/8
**56**	8/8	8/8	2/8	8/8	1/8	8/8

Table S2: 94 proteins in Protein profiling of HCCLM3-BM4 vs. HCCLM3-parental cells

Accessions	Protein	Log2 Foldchange	P value	HCCLM3-P (113)	HCCLM3-P (114)	HCCLM3-BM4(115)	HCCLM3-BM4(116)	Gene Name
Q5W0B1	RN219	1.951231	8.57E-03	0.50519	0.46449	1.600121	1.4302052	RING finger protein 219 OS=Homo sapiens GN=RNF219 PE=1 SV=1
P00414	COX3	1.789059	1.81E-04	0.49506	0.47138	1.6044651	1.4291039	Cytochrome c oxidase subunit 3 OS=Homo sapiens GN=MT-CO3 PE=1 SV=2
Q6N075	MFSD5	1.483457	1.66E-03	0.55921	0.53796	1.5527312	1.3500966	Molybdate-anion transporter OS=Homo sapiens GN=MFSD5 PE=1 SV=2
Q9BTM1	H2AJ	1.463885	2.22E-04	0.55794	0.53829	1.5486696	1.3551051	Histone H2A.J OS=Homo sapiens GN=H2AFJ PE=1 SV=1
Q71UI9	H2AV	1.355801	1.13E-04	0.56856	0.54856	1.5390663	1.3438106	Histone H2A.V OS=Homo sapiens GN=H2AFV PE=1 SV=3
Q16864	VATF	1.284311	3.79E-04	0.60397	0.55814	1.4910116	1.3468835	V-type proton ATPase subunit F OS=Homo sapiens GN=ATP6V1F PE=1 SV=2
P17931	LEG3	1.177207	1.40E-04	0.60421	0.5895	1.4669441	1.3393405	Galectin-3 OS=Homo sapiens GN=LGALS3 PE=1 SV=2
P62807	H2B1C	1.164009	1.33E-02	0.62457	0.61643	1.3647417	1.3942573	Histone H2B type 1-C/E/F/G/I OS=Homo sapiens GN=HIST1H2BC PE=1 SV=4
O60906	NSMA	1.157004	1.51E-03	0.62161	0.58592	1.482915	1.3095514	Sphingomyelin phosphodiesterase 2 OS=Homo sapiens GN=SMPD2 PE=1 SV=2
O43653	PSCA	1.147658	5.52E-03	0.58476	0.62552	1.4790844	1.3106351	Prostate stem cell antigen OS=Homo sapiens GN=PSCA PE=1 SV=1
Q9NP84	TNR12	1.12927	3.06E-03	0.60834	0.6056	1.4858819	1.3001721	Tumor necrosis factor receptor superfamily member 12A OS=Homo sapiens GN=TNFRSF12A PE=1 SV=1
Q96F44	TRI11	1.084555	9.73E-04	0.63958	0.59205	1.46195	1.3064172	E3 ubiquitin-protein ligase TRIM11 OS=Homo sapiens GN=TRIM11 PE=1 SV=2
P47914	RL29	1.060647	1.10E-03	0.65406	0.62272	1.3880932	1.3351278	60S ribosomal protein L29 OS=Homo sapiens GN=RPL29 PE=1 SV=2
Q86T03	TM55B	0.993397	1.73E-03	0.64416	0.66219	1.4160597	1.2775839	Type 1 phosphatidylinositol 4,5-bisphosphate 4-phosphatase OS=Homo sapiens GN=TMEM55B PE=1 SV=1
P29966	MARCS	0.98084	1.18E-03	0.67987	0.6513	1.3542738	1.3145497	Myristoylated alanine-rich C-kinase substrate OS=Homo sapiens GN=MARCKS PE=1 SV=4
P62750	RL23A	0.943285	4.33E-03	0.72512	0.63504	1.329166	1.310678	60S ribosomal protein L23a OS=Homo sapiens GN=RPL23A PE=1 SV=1
Q9Y5Y0	FLVC1	0.927284	1.16E-03	0.71309	0.65784	1.3769905	1.2520803	Feline leukemia virus subgroup C receptor-related protein 1 OS=Homo sapiens GN=FLVCR1 PE=1 SV=1
P35790	CHKA	0.922426	2.47E-03	0.69958	0.68242	1.3891898	1.2288054	Choline kinase alpha OS=Homo sapiens GN=CHKA PE=1 SV=3
Q6DN03	H2B2C	0.905013	6.62E-03	0.70942	0.68849	1.2835703	1.3185155	Putative histone H2B type 2-C OS=Homo sapiens GN=HIST2H2BC PE=5 SV=3
Q6PI78	TMM65	0.836854	4.90E-04	0.72412	0.70994	1.3377645	1.2281771	Transmembrane protein 65 OS=Homo sapiens GN=TMEM65 PE=1 SV=2
Q99717	SMAD5	0.825497	5.61E-04	0.72643	0.71086	1.3239545	1.2387532	Mothers against decapentaplegic homolog 5 OS=Homo sapiens GN=SMAD5 PE=1 SV=1
Q12894	IFRD2	0.824141	7.52E-03	0.75006	0.67579	1.3607981	1.2133486	Interferon-related developmental regulator 2 OS=Homo sapiens GN=IFRD2 PE=1 SV=3
P62899	RL31	0.817736	5.52E-03	0.72054	0.72099	1.2742541	1.2842144	60S ribosomal protein L31 OS=Homo sapiens GN=RPL31 PE=1 SV=1
O14521	DHSD	0.813883	8.36E-04	0.72926	0.71038	1.3423761	1.2179861	Succinate dehydrogenase [ubiquinone] cytochrome b small subunit, mitochondrial OS=Homo sapiens GN=SDHD PE=1 SV=1
Q92820	GGH	0.80005	6.02E-03	0.7455	0.69298	1.3557817	1.2057386	Gamma-glutamyl hydrolase OS=Homo sapiens GN=GGH PE=1 SV=2
P16989	YBOX3	0.784363	6.42E-03	0.72183	0.74512	1.2914142	1.2416394	Y-box-binding protein 3 OS=Homo sapiens GN=YBX3 PE=1 SV=4
P62805	H4	0.782766	2.73E-04	0.73585	0.7178	1.3180284	1.2283179	Histone H4 OS=Homo sapiens GN=HIST1H4A PE=1 SV=2
Q9NSU2	TREX1	0.782036	3.20E-03	0.72735	0.72992	1.3254927	1.2172329	Three-prime repair exonuclease 1 OS=Homo sapiens GN=TREX1 PE=1 SV=1
Q92804	RBP56	0.779653	8.80E-03	0.78367	0.68403	1.3009145	1.2313901	TATA-binding protein-associated factor 2N OS=Homo sapiens GN=TAF15 PE=1 SV=1
Q9NV92	NFIP2	0.776018	1.91E-03	0.73795	0.72507	1.2825591	1.2544205	NEDD4 family-interacting protein 2 OS=Homo sapiens GN=NDFIP2 PE=1 SV=2
P68402	PA1B2	0.764968	1.56E-02	0.70933	0.77109	1.284836	1.2347442	Platelet-activating factor acetylhydrolase IB subunit beta OS=Homo sapiens GN=PAFAH1B2 PE=1 SV=1
P67809	YBOX1	0.764158	6.34E-04	0.74026	0.72666	1.2923635	1.2407146	Nuclease-sensitive element-binding protein 1 OS=Homo sapiens GN=YBX1 PE=1 SV=3
Q9H300	PARL	0.759599	2.69E-03	0.73437	0.73757	1.3016435	1.2264162	Presenilins-associated rhomboid-like protein, mitochondrial OS=Homo sapiens GN=PARL PE=1 SV=2
Q9Y3U8	RL36	0.751962	1.24E-03	0.75662	0.71476	1.3139966	1.2146271	60S ribosomal protein L36 OS=Homo sapiens GN=RPL36 PE=1 SV=3
Q8NBQ5	DHB11	0.748791	4.07E-03	0.75473	0.7247	1.250308	1.270257	Estradiol 17-beta-dehydrogenase 11 OS=Homo sapiens GN=HSD17B11 PE=1 SV=3
O95159	ZFPL1	0.748366	1.25E-02	0.77927	0.69716	1.3520792	1.1714848	Zinc finger protein-like 1 OS=Homo sapiens GN=ZFPL1 PE=1 SV=2
Q5T440	CAF17	0.727921	6.02E-04	0.75251	0.73094	1.3028128	1.2137441	Putative transferase CAF17, mitochondrial OS=Homo sapiens GN=IBA57 PE=1 SV=1
P0CW18	PRS56	0.723205	1.50E-03	0.76866	0.72236	1.3035537	1.2054232	Serine protease 56 OS=Homo sapiens GN=PRSS56 PE=1 SV=1
P62273	RS29	0.664116	5.50E-03	0.78236	0.76451	1.2181274	1.2350082	40S ribosomal protein S29 OS=Homo sapiens GN=RPS29 PE=1 SV=2
Q15185	TEBP	0.643312	1.20E-03	0.78796	0.76546	1.2306475	1.2159305	Prostaglandin E synthase 3 OS=Homo sapiens GN=PTGES3 PE=1 SV=1
Q9UG56	PISD	0.637388	4.51E-03	0.79698	0.7519	1.2880317	1.1630859	Phosphatidylserine decarboxylase proenzyme, mitochondrial OS=Homo sapiens GN=PISD PE=2 SV=4
P62854	RS26	0.631754	5.55E-03	0.81384	0.73468	1.2750016	1.176474	40S ribosomal protein S26 OS=Homo sapiens GN=RPS26 PE=1 SV=3
Q8NC51	PAIRB	0.62665	6.90E-03	0.82459	0.73356	1.2333271	1.2085276	Plasminogen activator inhibitor 1 RNA-binding protein OS=Homo sapiens GN=SERBP1 PE=1 SV=2
Q9UIS9	MBD1	0.622151	4.46E-02	0.77778	0.81151	1.1180738	1.2926444	Methyl-CpG-binding domain protein 1 OS=Homo sapiens GN=MBD1 PE=1 SV=2
Q5BJH7	YIF1B	0.621127	5.77E-03	0.77804	0.79911	1.2099057	1.2129388	Protein YIF1B OS=Homo sapiens GN=YIF1B PE=1 SV=1
P23528	COF1	0.61724	2.27E-03	0.8125	0.7555	1.2227411	1.2092553	Cofilin-1 OS=Homo sapiens GN=CFL1 PE=1 SV=3

Names	Sequence	Area 113	Area 114	Area 115	Area 116
RING finger protein 219	ESSVVQAGGSK	12.82	9.96	98.69	123.32
RING finger protein 219	PLTLVQGNQNEDKHLVTDNPSK	667.11	261.48	2492.45	2908.78

(Continued on next page)

Continued

P62829	RL23	0.615076	1.51E-04	0.80209	0.76467	1.2454541	1.1877863	60S ribosomal protein L23 OS=Homo sapiens GN=RPL23 PE=1 SV=1
O76095	JTB	0.607557	8.77E-03	0.77293	0.81015	1.2579218	1.1590016	Protein JTB OS=Homo sapiens GN=JTB PE=1 SV=1
Q96S97	MYADM	0.604585	6.55E-03	0.79603	0.77559	1.2788987	1.1494788	Myeloid-associated differentiation marker OS=Homo sapiens GN=MYADM PE=1 SV=2
P35637	FUS	0.604297	1.21E-02	0.83422	0.73445	1.2674598	1.1638658	RNA-binding protein FUS OS=Homo sapiens GN=FUS PE=1 SV=1
P62633	CNBP	0.601353	2.37E-02	0.78032	0.79754	1.3131105	1.1090318	Cellular nucleic acid-binding protein OS=Homo sapiens GN=CNBP PE=1 SV=1
P61927	RL37	0.593791	2.89E-04	0.80694	0.77982	1.2180567	1.1951763	60S ribosomal protein L37 OS=Homo sapiens GN=RPL37 PE=1 SV=2
O75391	SPAG7	0.59018	1.23E-02	0.79797	0.81227	1.1685565	1.2212053	Sperm-associated antigen 7 OS=Homo sapiens GN=SPAG7 PE=1 SV=2
Q6PML9	ZNT9	-0.59411	3.96E-03	1.18869	1.20312	0.8116055	0.7965892	Zinc transporter 9 OS=Homo sapiens GN=SLC30A9 PE=1 SV=1
Q15119	PDK2	-0.60113	1.85E-02	1.16385	1.22986	0.7920959	0.8141893	[Pyruvate dehydrogenase (acetyl-transferring)] kinase isozyme 2, mitochondrial OS=Homo sapiens GN=PDK2 PE=1 SV=2
Q9H1E5	TMX4	-0.60144	2.35E-02	1.17035	1.21719	0.7884671	0.8239853	Thioredoxin-related transmembrane protein 4 OS=Homo sapiens GN=TMX4 PE=1 SV=1
Q9Y3B6	EMC9	-0.60364	1.79E-02	1.16903	1.22565	0.7906697	0.8146489	ER membrane protein complex subunit 9 OS=Homo sapiens GN=EMC9 PE=1 SV=3
O00180	KCNK1	-0.6186	3.48E-02	1.32527	1.10312	0.813472	0.7581363	Potassium channel subfamily K member 1 OS=Homo sapiens GN=KCNK1 PE=1 SV=1
Q8NFX7	STXB6	-0.62489	1.48E-02	1.25332	1.16377	0.778261	0.8046533	Syntaxin-binding protein 6 OS=Homo sapiens GN=STXBP6 PE=1 SV=2
Q9H773	DCTP1	-0.62569	2.53E-02	1.18736	1.22879	0.8492898	0.7345599	dCTP pyrophosphatase 1 OS=Homo sapiens GN=DCTPP1 PE=1 SV=1
P40692	MLH1	-0.62581	5.20E-03	1.22372	1.18977	0.7897879	0.7967307	DNA mismatch repair protein Mlh1 OS=Homo sapiens GN=MLH1 PE=1 SV=1
O75351	VPS4B	-0.62647	2.53E-03	1.2063	1.20645	0.7992039	0.7880401	Vacuolar protein sorting-associated protein 4B OS=Homo sapiens GN=VPS4B PE=1 SV=2
Q9UN37	VPS4A	-0.64506	5.06E-03	1.19433	1.22821	0.7986288	0.7788326	Vacuolar protein sorting-associated protein 4A OS=Homo sapiens GN=VPS4A PE=1 SV=1
Q9UPT5	EXOC7	-0.6826	1.12E-02	1.21204	1.23061	0.7659152	0.7914357	Exocyst complex component 7 OS=Homo sapiens GN=EXOC7 PE=1 SV=3
Q5W0Z9	ZDH20	-0.68732	8.41E-03	1.30736	1.15885	0.7813449	0.7524514	Probable palmitoyltransferase ZDHHC20 OS=Homo sapiens GN=ZDHHC20 PE=1 SV=1
P52895	AK1C2	-0.69148	1.35E-03	1.23702	1.21299	0.7872556	0.7627341	Aldo-keto reductase family 1 member C2 OS=Homo sapiens GN=AKR1C2 PE=1 SV=3
Q9BV23	ABHD6	-0.69211	5.82E-03	1.20735	1.24445	0.7910231	0.7571746	Monoacylglycerol lipase ABHD6 OS=Homo sapiens GN=ABHD6 PE=1 SV=1
O95167	NDUA3	-0.72079	7.50E-05	1.27161	1.20105	0.7849357	0.7423961	NADH dehydrogenase [ubiquinone] 1 alpha subcomplex subunit 3 OS=Homo sapiens GN=NDUFA3 PE=1 SV=1
O43242	PSMD3	-0.73087	4.58E-03	1.22253	1.25248	0.7828453	0.7421517	26S proteasome non-ATPase regulatory subunit 3 OS=Homo sapiens GN=PSMD3 PE=1 SV=2
Q15006	EMC2	-0.75579	1.21E-02	1.22498	1.28478	0.7967742	0.6934717	ER membrane protein complex subunit 2 OS=Homo sapiens GN=EMC2 PE=1 SV=1
P32929	CGL	-0.76746	4.45E-03	1.28597	1.23474	0.7911612	0.6881283	Cystathionine gamma-lyase OS=Homo sapiens GN=CTH PE=1 SV=3
O43760	SNG2	-0.77741	7.58E-03	1.27294	1.25709	0.7946226	0.675352	Synaptogyrin-2 OS=Homo sapiens GN=SYNGR2 PE=1 SV=1
Q9UK23	NAGPA	-0.79099	1.04E-02	1.21552	1.3196	0.768717	0.6961603	N-acetylglucosamine-1-phosphodiester alpha-N-acetylglucosaminidase OS=Homo sapiens GN=NAGPA PE=1 SV=2
Q00059	TFAM	-0.79882	5.12E-03	1.31125	1.21618	0.7273952	0.7451779	Transcription factor A, mitochondrial OS=Homo sapiens GN=TFAM PE=1 SV=1
P15121	ALDR	-0.80789	9.57E-04	1.28994	1.24311	0.7442526	0.7227039	Aldose reductase OS=Homo sapiens GN=AKR1B1 PE=1 SV=3
Q15527	SURF2	-0.82321	1.52E-02	1.33944	1.20666	0.7943177	0.6595798	Surfeit locus protein 2 OS=Homo sapiens GN=SURF2 PE=1 SV=3
Q7KZI7	MARK2	-0.82757	2.06E-02	1.25941	1.25983	0.71611	0.7646443	Serine/threonine-protein kinase MARK2 OS=Homo sapiens GN=MARK2 PE=1 SV=2
Q9BVT8	TMUB1	-0.86246	1.15E-02	1.24554	1.30843	0.7180145	0.7280086	Transmembrane and ubiquitin-like domain-containing protein 1 OS=Homo sapiens GN=TMUB1 PE=1 SV=1
Q9NWT6	HIF1N	-0.87545	3.52E-04	1.33265	1.24946	0.7381229	0.6797711	Hypoxia-inducible factor 1-alpha inhibitor OS=Homo sapiens GN=HIF1AN PE=1 SV=2
P04004	VTNC	-0.87942	2.92E-02	1.4577	1.14497	0.7244956	0.6728264	Vitronectin OS=Homo sapiens GN=VTN PE=1 SV=1
Q6UW56	ARAID	-0.88804	5.40E-03	1.26653	1.32894	0.7442468	0.6602777	All-trans retinoic acid-induced differentiation factor OS=Homo sapiens GN=ATRAID PE=1 SV=2
Q9UK97	FBX9	-0.94648	5.07E-03	1.29673	1.31869	0.7128471	0.6717293	F-box only protein 9 OS=Homo sapiens GN=FBXO9 PE=1 SV=1
P37268	FDFT	-1.0228	3.74E-03	1.33251	1.3372	0.6703285	0.6599552	Squalene synthase OS=Homo sapiens GN=FDFT1 PE=1 SV=1
Q9NVR2	INT10	-1.03843	4.94E-03	1.29509	1.34798	0.7150775	0.6418567	Integrator complex subunit 10 OS=Homo sapiens GN=INTS10 PE=1 SV=2
Q96PY5	FMNL2	-1.04387	1.57E-03	1.34168	1.30429	0.7161243	0.637905	Formin-like protein 2 OS=Homo sapiens GN=FMNL2 PE=1 SV=3
P51151	RAB9A	-1.07663	6.01E-03	1.31473	1.38485	0.6560985	0.644318	Ras-related protein Rab-9A OS=Homo sapiens GN=RAB9A PE=1 SV=1
O75608	LYPA1	-1.11467	2.45E-03	1.34498	1.37264	0.6391517	0.6432274	Acyl-protein thioesterase 1 OS=Homo sapiens GN=LYPLA1 PE=1 SV=1
Q9Y2W2	WBP11	-1.12351	9.51E-05	1.38753	1.32482	0.6505823	0.637067	WW domain-binding protein 11 OS=Homo sapiens GN=WBP11 PE=1 SV=1
P40616	ARL1	-1.15289	1.02E-02	1.34635	1.3833	0.6116957	0.6586542	ADP-ribosylation factor-like protein 1 OS=Homo sapiens GN=ARL1 PE=1 SV=1
P27448	MARK3	-1.28438	2.89E-03	1.41379	1.39689	0.5810361	0.6082859	MAP/microtubule affinity-regulating kinase 3 OS=Homo sapiens GN=MARK3 PE=1 SV=4
Q8NCQ2	CNAS1	-1.45656	3.36E-03	1.61696	1.34192	0.5266036	0.5145202	Uncharacterized protein CSNK1G2-AS1 OS=Homo sapiens GN=CSNK1G2-AS1 PE=2 SV=2
Q14145	KEAP1	-1.48529	4.44E-03	1.56636	1.40857	0.4695892	0.5554795	Kelch-like ECH-associated protein 1 OS=Homo sapiens GN=KEAP1 PE=1 SV=2
Q99969	RARR2	-1.5285	1.01E-02	1.47351	1.49357	0.4727995	0.5601275	Retinoic acid receptor responder protein 2 OS=Homo sapiens GN=RARRES2 PE=1 SV=1
Q8IWV1	LAX1	-1.91047	5.74E-03	2.00386	1.27831	0.413466	0.3043688	Lymphocyte transmembrane adapter 1 OS=Homo sapiens GN=LAX1 PE=1 SV=1

RING finger protein 219	FAVAALQSK	1296.99	1434.54	5346.49	5340.23
RING finger protein 219	LDVTDTSMDTYLER	667.11	261.48	2492.45	2908.78
RING finger protein 219	NLSLESQIK	3314.76	3998.01	22099.16	20825.3
RING finger protein 219	SLDLDGLSK	3659.85	3414.84	18140.3	21295.9
RING finger protein 219	EIIGGTSESEPMLSH	332.65	326.7	492.06	841.9
RING finger protein 219	LKLENGGLVR	196.22	188.4	226.96	250.42
RING finger protein 219	KIQSSLSSASPSK	75.78	172.69	115.6	69.95
RING finger protein 219	DVTDTSMDTYLER	38.42	34.38	73.22	60.13
Cytochrome c oxidase subunit 3	ESTYQGHHTPPVQK	114.29	91.82	251.84	241.08
Cytochrome c oxidase subunit 3	ITIL	114.69	158.48	37.77	113.82
Cytochrome c oxidase subunit 3	LGLL	1296.99	1434.54	5346.49	5340.23
Cytochrome c oxidase subunit 3	LGLL	667.11	261.48	2492.45	2908.78
Cytochrome c oxidase subunit 3	LGLL	3314.76	3998.01	22099.16	20825.3
Cytochrome c oxidase subunit 3	LGLL	3659.85	3414.84	18140.3	21295.9
Cytochrome c oxidase subunit 3	LGLL	332.65	326.7	492.06	841.9
Molybdate-anion transporter	VPSPTEEPYAPEL	1229.65	1172.35	3403.93	4897.02
Molybdate-anion transporter	LAVL	4384.27	4183.51	14747.8	17040.8
Molybdate-anion transporter	LGLL	2105.29	1682.61	3410.78	4449.91
Molybdate-anion transporter	LGLL	1505.25	1603.39	2879.59	2988.3
Molybdate-anion transporter	LGLL	1057.77	1452.14	2799.67	2329.69
Molybdate-anion transporter	LGLL	1179.78	1206.45	2644.64	2135.2
Molybdate-anion transporter	LGLL	893.03	813.09	2037.03	1522
Molybdate-anion transporter	LLAL	3427.6	4552.18	19059.3	19150.2
Molybdate-anion transporter	LLAL	11917.0	10813.6	45916.9	50894.3
Molybdate-anion transporter	VPSPTEEPYAPEL	1558.4	1564.95	8416.24	7153.81
Histone H2A.J	NDEELNKLLGK	3095.22	2922.24	10321.92	12424.92
Histone H2A.J	AGLQFPVGR	735.36	539.74	1137.2	1255.8
Histone H2A.J	AGLQFPVGR	7391.74	7902.96	31114.53	37885.3
Histone H2A.J	AGLQFPVGR	3719.2	3082.61	12229.63	15352.61
Histone H2A.J	AGLQFPVGR	4353.16	3002.59	18013.83	16481.37
Histone H2A.J	AGLQFPVGR	3005.12	3845.47	14662.96	15630.89
Histone H2A.J	AGLQFPVGR	2346.47	1790.98	6527.64	5785.87
Histone H2A.J	AGLQFPVGR	1232.46	1639.57	5706.37	6792.3
Histone H2A.J	AGLQFPVGR	1543.28	1463.39	4984.86	3335.49
Histone H2A.J	AGLQFPVGR	1229.65	1172.35	3403.93	4897.02
Histone H2A.J	AGLQFPVGR	4384.27	4183.51	14747.87	17040.89
Histone H2A.J	AGLQFPVGR	2105.29	1682.61	3410.78	4449.91
Histone H2A.J	AGLQFPVGR	1505.25	1603.39	2879.59	2988.3
Histone H2A.J	AGLQFPVGR	1057.77	1452.14	2799.67	2329.69
Histone H2A.J	AGLQFPVGR	1179.78	1206.45	2644.64	2135.2
Histone H2A.J	AGLQFPVGR	893.03	813.09	2037.03	1522
Histone H2A.J	AGLQFPVGR	3427.6	4552.18	19059.3	19150.29
Histone H2A.J	AGLQFPVGR	11917.04	10813.63	45916.92	50894.32
Histone H2A.J	AGLQFPVGR	1558.4	1564.95	8416.24	7153.81
Histone H2A.J	AGLQFPVGR	1930.54	968.73	2663.15	3057.85
Histone H2A.J	AGLQFPVGR	3083.15	3902.41	10017.74	10807.33
Histone H2A.J	AGLQFPVGR	1605.11	1524.98	3253.99	2625.91
Histone H2A.J	AGLQFPVGR	1586.56	1230.13	6418.46	4920.93
Histone H2A.J	AGLQFPVGR	7803.66	6567.92	31816.9	34752.98
Histone H2A.J	AGLQFPVGR	1567.22	1119.55	2757.29	4205.61
Histone H2A.J	AGLQFPVGR	2736.28	2852.93	8417.13	8692.62
Histone H2A.J	AGLQFPVGR	5666.01	5635.96	19796.31	21829.52
Histone H2A.J	AGLQFPVGR	706.08	772.74	2590.48	2318.32
Histone H2A.J	AGLQFPVGR	1682.07	1368.73	6871.55	6293.89
Histone H2A.J	AGLQFPVGR	1364.82	2073.96	5169.37	6334.01
Histone H2A.J	AGLQFPVGR	7356.34	7571.09	38671.86	39202.02
Histone H2A.J	AGLQFPVGR	808.66	850.58	2566.07	1891.6
Histone H2A.J	AGLQFPVGR	1076.3	1004.63	1730.47	1973.07
Histone H2A.J	AGLQFPVGR	5221.48	4873.95	19067.57	19826.8
Histone H2A.J	AGLQFPVGR	5211.96	4750.17	19959.64	23151.88
Histone H2A.J	AGLQFPVGR	1975.77	2138.09	8959.97	8677.1
Histone H2A.J	AGLQFPVGR	1265.45	1571.74	5217.66	5867.18
Histone H2A.J	AGLQFPVGR	1474.02	1922.84	4478.49	5591.33
Histone H2A.J	AGLQFPVGR	1088.92	1353.18	2102.07	2730.95

Histone H2A.J	AGLQFPVGR	734.61	826.28	2656.6	3038.88
Histone H2A.J	AGLQFPVGR	1115.46	752.24	2104.38	2955.29
Histone H2A.J	AGLQFPVGR	844.59	705.34	1321.23	1843.46
Histone H2A.J	AGLQFPVGR	989.68	731.88	2136.19	2097.21
Histone H2A.J	AGLQFPVGR	1056.54	1042.06	4313.21	4131.46
Histone H2A.J	AGLQFPVGR	2156.06	1985.76	3015.43	3427.61
Histone H2A.J	AGLQFPVGR	2783.63	2962.21	12465.42	12932.62
Histone H2A.J	AGLQFPVGR	995.36	1022.58	2309.6	2467.86
Histone H2A.J	AGLQFPVGR	965.41	1108.98	1390.06	1732.83
Histone H2A.J	AGLQFPVGR	9080.93	9738.21	42648.28	47032.29
Histone H2A.J	AGLQFPVGR	801.65	479.34	2817.88	2014.33
Histone H2A.J	AGLQFPVGR	946.89	598.13	1303.19	1877.72
Histone H2A.J	AGLQFPVGR	12921.42	13072.49	56983.82	62024.65
Histone H2A.J	AGLQFPVGR	973.93	692.24	1016.59	1318.97
Histone H2A.J	AGLQFPVGR	906.98	1350.04	1381.44	1763.9
Histone H2A.J	AGLQFPVGR	780.34	776.35	2192.85	1573.82
Histone H2A.J	AGLQFPVGR	913.85	670.53	715.07	970.42
Histone H2A.J	AGLQFPVGR	1451.42	1283.56	6770.79	6163.01
Histone H2A.J	AGLQFPVGR	2052.99	1955.73	5889.32	5656.88
Histone H2A.J	AGLQFPVGR	973.28	969.46	1454.53	1594.17
Histone H2A.J	AGLQFPVGR	846.83	483.9	2214.39	1892.43
Histone H2A.J	AGLQFPVGR	460.6	450.89	1486.04	1116.19
Histone H2A.J	AGLQFPVGR	875.51	1161.33	3012.76	3331.16
Histone H2A.J	AGLQFPVGR	411.83	163.42	619.25	875.38
Histone H2A.J	AGLQFPVGR	1027.49	1251.77	2183.95	2801.88
Histone H2A.J	AGLQFPVGR	9789.6	8978.43	47210.65	48156.78
Histone H2A.J	AGLQFPVGR	7337.1	6812.75	30936.86	32724.84
Histone H2A.J	AGLQFPVGR	2191.89	1743.8	8310.88	6655.3
Histone H2A.J	AGLQFPVGR	381.79	578.16	532.32	470.78
Histone H2A.J	AGLQFPVGR	770.94	853.63	1649.32	2090.21
Histone H2A.J	AGLQFPVGR	2561.86	2745.08	9923.76	11767.1
Histone H2A.J	AGLQFPVGR	1072.29	1081.21	2372.08	1835.76
Histone H2A.J	AGLQFPVGR	681.56	960.87	1884.51	1837.95
Histone H2A.J	AGLQFPVGR	11781.22	10827.76	63002.79	65067.61
Histone H2A.J	AGLQFPVGR	634.06	653.99	2292.9	1868.72
Histone H2A.J	AGLQFPVGR	829.97	766.59	3823.66	4723.98
Histone H2A.J	AGLQFPVGR	1583.64	1837.12	6030.03	6618.06
Histone H2A.J	AGLQFPVGR	1463.63	1779.87	1801.83	2111.98
Histone H2A.J	AGLQFPVGR	1990.11	2530.31	7270.88	5859.88
Histone H2A.J	AGLQFPVGR	1177.54	1108.01	1724.2	1887.06
Histone H2A.J	AGLQFPVGR	807.81	934.77	1702.29	1692.95
Histone H2A.J	AGLQFPVGR	1191.31	1024.77	1802.28	1715.42
Histone H2A.J	AGLQFPVGR	15580.76	14908.89	71607.21	74831.87
Histone H2A.J	AGLQFPVGR	770.96	533.16	1331.88	1680.9
Histone H2A.J	AGLQFPVGR	800.46	384.67	1016.38	1345.14
Histone H2A.J	AGLQFPVGR	728.49	1217.84	1820.1	2499.85
Histone H2A.J	AGLQFPVGR	5500.73	6113.81	22880.82	28990.17
Histone H2A.J	AGLQFPVGR	13342.21	14477.4	69294.52	77085.73
Histone H2A.J	AGLQFPVGR	963.56	866.55	1421.16	1210.32
Histone H2A.J	AGLQFPVGR	659.15	515.27	924.45	1211.15
Histone H2A.J	AGLQFPVGR	1169.19	1720.32	4390.78	5177.26
Histone H2A.J	AGLQFPVGR	1835.38	1965.98	5807.36	7331.35
Histone H2A.J	AGLQFPVGR	750.79	717.55	2390.72	1899.98
Histone H2A.J	AGLQFPVGR	4341.52	3939.4	16395.2	19115.57
Histone H2A.J	AGLQFPVGR	3840.33	2711.39	13979.03	14060.98
Histone H2A.J	AGLQFPVGR	13267.3	12410.55	64418.72	68188.72
Histone H2A.J	AGLQFPVGR	1669.13	2646.7	5934.27	9711.38
Histone H2A.J	AGLQFPVGR	1256.66	845.09	3589.58	3030.78
Histone H2A.J	AGLQFPVGR	22173.4	23357.7	113914.5	117779.3
Histone H2A.J	AGLQFPVGR	615.78	1290.12	2115.03	2124.18
Histone H2A.J	AGLQFPVGR	4396.96	3355.39	8858.52	9554.16
Histone H2A.J	AGLQFPVGR	2323.51	2144.22	7595.02	5547.48
Histone H2A.J	AGLQFPVGR	771.65	783.48	1818.86	1461.33
Histone H2A.J	AGLQFPVGR	755.07	613.21	1845.28	1443.02

Histone H2A.J	AGLQFPVGR	1337.63	1462.02	1498.03	2430.35
Histone H2A.J	AGLQFPVGR	1787.99	1648.17	2500.27	2807.22
Histone H2A.J	AGLQFPVGR	1206.66	891.01	1926.46	1862.94
Histone H2A.J	AGLQFPVGR	15965.26	17347.55	82594.47	85498.15
Histone H2A.J	AGLQFPVGR	2950.12	2896.52	7923.98	8552.25
Histone H2A.J	AGLQFPVGR	1914.91	1768.74	3092.14	3497.98
Histone H2A.J	AGLQFPVGR	25003.83	27547.2	133263	132321.3
Histone H2A.J	AGLQFPVGR	776.19	335.35	1311.11	830.06
Histone H2A.J	AGLQFPVGR	1078.53	890.24	1530.31	1802.05
Histone H2A.J	AGLQFPVGR	621.31	530.86	664.59	649.84
Histone H2A.J	AGLQFPVGR	974.47	1596.7	3559.94	2425.87
Histone H2A.J	AGLQFPVGR	1512.85	1810.52	8615.78	8315.48
Histone H2A.J	AGLQFPVGR	18520.47	21280.89	94612.43	100476.7
Histone H2A.J	AGLQFPVGR	688.78	673.05	1096.26	1126.67
Histone H2A.J	AGLQFPVGR	461.72	340.39	618.4	920.2
Histone H2A.J	AGLQFPVGR	1259.02	1143.55	2220.91	2744.3
Histone H2A.J	AGLQFPVGR	2654.47	3382.38	4919.8	5595.68
Histone H2A.J	AGLQFPVGR	357.04	469.41	1002.77	1192.24
Histone H2A.J	AGLQFPVGR	1270.08	901.76	1428.42	1014.2
Histone H2A.J	AGLQFPVGR	21202.08	18045.75	97054.73	97989.67
Histone H2A.J	AGLQFPVGR	1022	624.66	1789.91	1699.53
Histone H2A.J	AGLQFPVGR	6274.58	6892.98	33468.67	34576.11
Histone H2A.J	AGLQFPVGR	1949.98	1814.34	5335.88	4130.09
Histone H2A.J	AGLQFPVGR	408.49	493.33	854.2	937.65
Histone H2A.J	AGLQFPVGR	953.99	748.01	1948.09	1932.83
Histone H2A.J	AGLQFPVGR	7506.25	6802.85	15275.03	16186.78
Histone H2A.J	AGLQFPVGR	25508.24	26861.2	137211.1	150031.6
Histone H2A.J	AGLQFPVGR	839.08	1142.89	2037.25	2143.55
Histone H2A.J	AGLQFPVGR	204.16	254.95	1958.89	898.18
Histone H2A.J	AGLQFPVGR	9390.52	9364.29	40276.81	43372.96
Histone H2A.J	AGLQFPVGR	781.62	755.91	1178.04	1225.45
Histone H2A.J	AGLQFPVGR	359.77	199.84	285.26	343.68
Histone H2A.J	AGLQFPVGR	1064.99	845.5	2463.35	2116.21
Histone H2A.J	AGLQFPVGR	12898.72	12549.22	60824.75	66481.3
Histone H2A.J	AGLQFPVGR	717.32	938.89	1776.93	1638.44
Histone H2A.J	AGLQFPVGR	1512.12	1244.2	2112.28	2589.62
Histone H2A.J	AGLQFPVGR	3754.24	4727.03	5169.87	7731.69
Histone H2A.J	AGLQFPVGR	730.17	946.19	2379.41	2757.84
Histone H2A.J	AGLQFPVGR	1010.57	1328.08	1981.97	1578.95
Histone H2A.J	AGLQFPVGR	594.64	931.85	1298.1	1376.65
Histone H2A.J	AGLQFPVGR	5684.85	6008.46	10502.22	14303.33
Histone H2A.J	AGLQFPVGR	1499.12	2103.35	6733.66	4358.06
Histone H2A.J	AGLQFPVGR	10952.99	11591.67	47877.18	49740.2
Histone H2A.J	AGLQFPVGR	1930.09	2821.77	2753.96	2318.96
Histone H2A.J	AGLQFPVGR	32037.25	35319.58	180394.8	177293.2
Histone H2A.J	AGLQFPVGR	6193.2	8550.31	11781.76	14807.87
Histone H2A.J	AGLQFPVGR	1314.71	1279.9	2718.63	2826.48
Histone H2A.J	AGLQFPVGR	1116.67	1696.97	685.15	644.61
Histone H2A.J	AGLQFPVGR	8329.98	7760.7	12479.6	16636.78
Histone H2A.J	AGLQFPVGR	881.92	1484.84	1720.03	2290.7
Histone H2A.J	AGLQFPVGR	778.46	536.83	1495.14	1886.07
Histone H2A.J	AGLQFPVGR	459.93	198.27	1272.39	1253.57
Histone H2A.J	AGLQFPVGR	17775.7	16564.34	95128.73	90015.13
Histone H2A.J	AGLQFPVGR	538.35	429.01	1059.91	969.96
Histone H2A.J	AGLQFPVGR	959.21	1828.02	2811.16	3161.15
Histone H2A.J	AGLQFPVGR	307.29	278.98	392.5	897.91
Histone H2A.J	AGLQFPVGR	509.98	785.59	1929.15	706.56
Histone H2A.J	AGLQFPVGR	278.74	546.54	1197.66	1129.15
Histone H2A.J	AGLQFPVGR	1255.47	2664.7	10602.54	2080.34
Histone H2A.J	AGLQFPVGR	883.09	837.78	692.62	1080.91
Histone H2A.J	AGLQFPVGR	8340.85	9878.16	12958.73	17024.35
Histone H2A.J	AGLQFPVGR	7429.04	9324.5	12678.39	16348.85
Histone H2A.J	AGLQFPVGR	436.23	305.23	1174.46	967.27
Histone H2A.J	AGLQFPVGR	1440.41	1413.68	3246.5	2125.68

Histone H2A.J	AGLQFPVGR	769.75	758.75	2889.53	693.74
Histone H2A.J	AGLQFPVGR	765.55	1173.51	2653.85	1286.41
Histone H2A.J	AGLQFPVGR	799.88	1505.24	2341.31	1378.18
Histone H2A.J	AGLQFPVGR	9134.02	11195.56	41371.96	53640.86
Histone H2A.J	AGLQFPVGR	358.62	313.44	1052.6	581.51
Histone H2A.J	AGLQFPVGR	658.77	1191.15	3183.2	1092.5
Histone H2A.J	AGLQFPVGR	425.61	290.65	658.22	350.91
Histone H2A.J	AGLQFPVGR	438.12	118.68	183.07	477.05
Histone H2A.J	AGLQFPVGR	535.37	830.33	1005.83	960.25
Histone H2A.J	AGLQFPVGR	397.55	510.46	1270.11	1211.33
Histone H2A.J	AGLQFPVGR	24910.02	28091.79	141081.8	137110
Histone H2A.J	AGLQFPVGR	7062.89	9223.16	15097.36	16468.32
Histone H2A.J	AGLQFPVGR	968.2	620.37	709.89	709.61
Histone H2A.J	AGLQFPVGR	2521.6	3743.75	13966.48	16544.49
Histone H2A.J	AGLQFPVGR	2521.6	3743.75	13966.48	16544.49
Histone H2A.J	AGLQFPVGR	406.9	543.67	1082.3	818.21
Histone H2A.J	AGLQFPVGR	840.21	1031.86	3057.41	3192.68
Histone H2A.J	AGLQFPVGR	668.36	350.57	421.08	623.23
Histone H2A.J	AGLQFPVGR	1418.3	1683.46	1098.86	958.1
Histone H2A.J	AGLQFPVGR	2912.07	3038.75	1133.96	1269.9
Histone H2A.J	AGLQFPVGR	178.05	281.42	745.2	320.37
Histone H2A.J	AGLQFPVGR	852.94	1156.96	5918.07	700.19
Histone H2A.J	AGLQFPVGR	16367.95	19583.16	72920.64	82336.19
Histone H2A.J	AGLQFPVGR	488.76	541.34	685.19	1306.41
Histone H2A.J	AGLQFPVGR	23470.75	25820.33	120423.2	119929.1
Histone H2A.J	AGLQFPVGR	509.98	785.59	1929.15	706.56
Histone H2A.J	AGLQFPVGR	888.88	691.45	2165.07	2089.24
Histone H2A.J	AGLQFPVGR	769.75	758.75	2889.53	693.74
Histone H2A.J	AGLQFPVGR	28765.71	27264.85	157592.2	146114.7
Histone H2A.J	AGLQFPVGR	1207.97	1080.54	5737.75	6853.03
Histone H2A.J	AGLQFPVGR	4045.77	4648.2	2469.06	2154.7
Histone H2A.J	AGLQFPVGR	31596.32	30159.57	175405.2	170862.1
Histone H2A.J	AGLQFPVGR	31384.23	39943.32	198050.1	194084.3
Histone H2A.J	AGLQFPVGR	333.11	281.33	181.56	196.3
Histone H2A.J	AGLQFPVGR	658.77	1191.15	3183.2	1092.5
Histone H2A.J	AGLQFPVGR	26360.24	26302.61	145444.1	146949.2
Histone H2A.J	AGLQFPVGR	28568.85	31075.36	159389.5	154730
Histone H2A.J	AGLQFPVGR	30331.6	30643.17	171915	166589.2
Histone H2A.J	AGLQFPVGR	769.12	501.57	1093.09	1370.01
Histone H2A.J	AGLQFPVGR	33743.25	33579.08	190507.8	186818.8
Histone H2A.J	AGLQFPVGR	30236.94	30207.49	160197.4	157297.6
Histone H2A.J	AGLQFPVGR	52526.38	52207.16	281929.4	304593.4
Histone H2A.J	AGLQFPVGR	1354.4	1932.75	3841.77	2597.42
Histone H2A.J	AGLQFPVGR	58565.43	53434.29	298827.4	324923.5
Histone H2A.J	AGLQFPVGR	958.12	552.94	1210.06	859.72
Histone H2A.J	AGLQFPVGR	486.55	334.21	460.71	960.71
Histone H2A.J	AGLQFPVGR	1193.06	2128.14	5086.7	2653.11
Histone H2A.J	AGLQFPVGR	1195.6	1983.61	4354.74	3935.23
Histone H2A.J	AGLQFPVGR	179.5	138.09	170.7	16.57
Histone H2A.J	AGLQFPVGR	571.42	566.22	3034.2	2546.55
Histone H2A.J	AGLQFPVGR	31542.75	30133.07	179465.5	172668.1
Histone H2A.J	AGLQFPVGR	5621.18	6022.58	28229.04	26278.96
Histone H2A.J	AGLQFPVGR	231.27	129.64	398.74	159.37
Histone H2A.J	AGLQFPVGR	408.3	512.34	1191.24	1045.61
Histone H2A.J	AGLQFPVGR	59862.48	57948.53	352305.3	381336.6
Histone H2A.J	AGLQFPVGR	561.55	584.14	1333.07	1520.52
Histone H2A.J	AGLQFPVGR	256.34	206.44	337.55	314.07
Histone H2A.J	AGLQFPVGR	789.17	967.8	2796.5	2654.17
Histone H2A.J	AGLQFPVGR	5621.18	6022.58	28229.04	26278.96
Histone H2A.J	AGLQFPVGR	305.85	421.07	1028.26	706.26
Histone H2A.J	AGLQFPVGR	33825.63	35865.82	195702.3	183745.3
Histone H2A.J	AGLQFPVGR	356.25	547.45	868.27	1357.04
Histone H2A.J	AGLQFPVGR	958.12	552.94	1210.06	859.72
Histone H2A.J	AGLQFPVGR	30350.76	33111.27	186082.3	169240.5

Histone H2A.J	AGLQFPVGR	956.41	934.7	2065.4	2277.84
Histone H2A.J	AGLQFPVGR	872.12	1708.26	4692.79	5115.06
Histone H2A.J	AGLQFPVGR	614.01	613.72	688.21	1293.43
Histone H2A.J	AGLQFPVGR	381.98	558.93	685.14	924.91
Histone H2A.J	AGLQFPVGR	204.06	265.09	181.36	247.95
Histone H2A.J	AGLQFPVGR	799.88	1505.24	2341.31	1378.18
Histone H2A.J	AGLQFPVGR	408.02	433.59	1311.1	1082.41
Histone H2A.J	AGLQFPVGR	1207.97	1080.54	5737.75	6853.03
Histone H2A.J	AGLQFPVGR	506.73	362.05	871.03	1321.75
Histone H2A.J	AGLQFPVGR	756.81	866.76	1424.1	987.71
Histone H2A.J	AGLQFPVGR	496.18	340.55	484.39	1051.85
Histone H2A.J	AGLQFPVGR	669.57	337.37	1175.99	1326.11
Histone H2A.J	AGLQFPVGR	305.24	480.89	571.52	414.08
Histone H2A.J	AGLQFPVGR	505.84	1088.23	909.13	1098.16
Histone H2A.J	AGLQFPVGR	1001.85	487.22	1056.17	1783.61
Histone H2A.J	AGLQFPVGR	1416.92	1075.45	1659.77	2608.41
Histone H2A.J	AGLQFPVGR	1932.74	2879.5	1164.89	1161.08
Histone H2A.J	AGLQFPVGR	3013.27	3785.06	12302.32	13726.99
Histone H2A.J	AGLQFPVGR	953.46	1332.51	2184.47	2191.61
Histone H2A.J	AGLQFPVGR	153.13	190.41	232.33	427.19
Histone H2A.J	AGLQFPVGR	304.28	575.71	937.6	823.44
Histone H2A.J	AGLQFPVGR	1255.47	2664.7	10602.54	2080.34
Histone H2A.J	AGLQFPVGR	4884.28	5422.52	2794.05	2499.44
Histone H2A.J	AGLQFPVGR	3013.27	3785.06	12302.32	13726.99
Histone H2A.J	AGLQFPVGR	1833.78	1698.45	3409.3	3955.73
Histone H2A.J	AGLQFPVGR	535.03	438.15	726.65	1141.12
Histone H2A.J	AGLQFPVGR	409.55	388.09	1003.16	813.93
Histone H2A.J	AGLQFPVGR	651.37	645.75	1306.89	1501.44
Histone H2A.J	AGLQFPVGR	506.73	362.05	871.03	1321.75
Histone H2A.J	AGLQFPVGR	496.18	340.55	484.39	1051.85
Histone H2A.J	AGLQFPVGR	230.21	234.19	337.37	264.51
Histone H2A.J	AGLQFPVGR	1663.44	2571.04	6027.59	9178.14
Histone H2A.J	AGLQFPVGR	523.28	535.42	1170.39	1428.15
Histone H2A.J	AGLQFPVGR	334.77	116.6	664.71	792.52
Histone H2A.J	AGLQFPVGR	383.11	447.31	723.55	706.98
Histone H2A.J	AGLQFPVGR	12694.93	12683.01	52399.88	61492.76
Histone H2A.J	GLQFPVGR	5465.72	3615.26	4160.52	5161
Histone H2A.J	GLQFPVGR	3623.58	3806.48	8633.53	11317.45
Histone H2A.J	GLQFPVGR	5458.29	4774.93	13576.62	15270.13
Histone H2A.J	HLQLAIR	3406.51	2805.59	9845.33	9587
Histone H2A.J	HLQLAIR	4891.35	6532.1	17241.07	18515.23
Histone H2A.J	HLQLAIR	2459.32	2024.44	4086.24	6509.23
Histone H2A.J	HLQLAIR	7413.79	6682.28	19822.83	25349.53
Histone H2A.J	HLQLAIR	495.22	434.72	935.64	881.85
Histone H2A.J	HLQLAIR	282	224.26	638.52	477.65
Histone H2A.J	HLQLAIR	1530.69	1537.48	3653.3	3427
Histone H2A.J	HLQLAIR	3931.58	4653.8	12025.64	13178.84
Histone H2A.J	HLQLAIR	1002.23	982.03	3598.64	4772.14
Histone H2A.J	HLQLAIR	1935.65	1740.56	4303.7	3262.49
Histone H2A.J	HLQLAIR	15328.33	16445.01	49841.69	56921.78
Histone H2A.J	HLQLAIR	563.83	465.08	405.85	895.7
Histone H2A.J	HLQLAIR	1388.91	1608.16	3389.16	3857.72
Histone H2A.J	HLQLAIR	10528.26	9191.79	30012.6	33431.36
Histone H2A.J	HLQLAIR	669.97	723.28	808.02	1712.34
Histone H2A.J	HLQLAIR	559.96	634.81	853.33	941.75
Histone H2A.J	HLQLAIR	384.9	270.38	533.4	793.71
Histone H2A.J	HLQLAIR	1740.16	1160.57	2860.4	3819.71
Histone H2A.J	HLQLAIR	781.09	489.86	936.67	1419
Histone H2A.J	HLQLAIR	887.97	781.1	765.9	1651.08
Histone H2A.J	HLQLAIR	2482.4	1978.24	3467.88	4010.84
Histone H2A.J	HLQLAIR	610.06	791.49	1430.07	2167.54
Histone H2A.J	HLQLAIR	14199.58	13601.52	47619.1	56553.43
Histone H2A.J	HLQLAIR	963.2	1540.38	2557.8	2904.39
Histone H2A.J	HLQLAIR	690.08	758.18	1082.08	919.83

Histone H2A.J	HLQLAIR	631.41	916.33	1080.23	1045.42
Histone H2A.J	HLQLAIR	308.12	196.69	630.41	589.88
Histone H2A.J	HLQLAIR	513.5	330.95	895.5	1183.96
Histone H2A.J	HLQLAIR	573.38	585.5	743.35	562.86
Histone H2A.J	HLQLAIR	537.34	528.6	658.33	1329.39
Histone H2A.J	IIPR	640.2	366.14	1243.74	910.95
Histone H2A.J	IQAVLLPK	1305.23	1258.8	2254.8	2691.81
Histone H2A.J	IQAVLLPK	559.15	821.1	1109.19	1145.08
Histone H2A.J	KGNYAER	254.49	282.79	1142.13	1352.36
Histone H2A.J	KGNYAER	206.92	195	871.88	1031.79
Histone H2A.J	LLGK	3497.2	4271.32	5463.79	5805.31
Histone H2A.J	LLGK	6358.35	8191.63	19930.68	27872.12
Histone H2A.J	LLGK	5627.84	5364.58	18307.75	21015.58
Histone H2A.J	LLGK	4093.22	3045.08	7609.02	8332.05
Histone H2A.J	LLGK	2955.88	3816.51	8510.03	11071
Histone H2A.J	LLGK	1741.15	1701.46	2482.79	3844.17
Histone H2A.J	LLPK	4819.23	6635.9	9232.09	9789.93
Histone H2A.J	LLPK	1534.23	1613.26	2604.07	4192.49
Histone H2A.J	LLPK	2978.85	3887.58	5122.53	5474.69
Histone H2A.J	NDEELNKLLGK	902.6	1143.99	3824.53	7125.37
Histone H2A.J	NDEELNKLLGK	2901.36	2500.87	8951.51	11805.43
Histone H2A.J	NDEELNKLLGK	5623.4	5164.91	26073.44	32967.19
Histone H2A.J	NDEELNKLLGK	5021.62	6321.13	25444.44	37838.48
Histone H2A.J	NDEELNKLLGK	2420.32	1940.94	5912.96	8101.84
Histone H2A.J	NDEELNKLLGK	5596.52	5054.19	21755.51	30486.4
Histone H2A.J	NDEELNKLLGK	4912.7	5593.56	26197.29	35994.03
Histone H2A.J	NDEELNKLLGK	792.81	820.98	2492.88	2953
Histone H2A.J	NDEELNKLLGK	1539.82	1911.76	3615.23	3935.66
Histone H2A.J	NDEELNKLLGK	1737.71	2147.31	3950.68	4334.55
Histone H2A.J	NDEELNKLLGK	2453.13	3274.67	10666.68	15847.08
Histone H2A.J	NDEELNKLLGK	1345.14	1252.22	2486.98	2554.85
Histone H2A.J	RAGLQFPVGR	842.15	2329.3	10836.33	1053.15
Histone H2A.J	RAGLQFPVGR	571.41	779.52	993.43	409.63
Histone H2A.J	TRIIPRHLQLAIR	2335.79	2101.65	3804.97	3910.23
Histone H2A.J	TRIIPRHLQLAIR	2184.26	1965.37	2697.14	3301.59
Histone H2A.J	VLLPK	1406.89	1640.91	2820.39	2781.79
Histone H2A.J	VLLPK	1770.34	1689.95	2306.96	3430.99
Histone H2A.J	VLLPK	3943.06	3199.82	5823.9	7006.84
Histone H2A.J	VLLPK	1595.46	1839.56	2887.4	3401.13
Histone H2A.J	VTIAQGGVLP	1190.82	115.51	134.33	328.03
Histone H2A.J	VTIAQGGVLPN	511.18	454.14	1139.99	865.66
Histone H2A.J	VTIAQGGVLPN	253.1	526.63	680.64	369.69
Histone H2A.J	VTIAQGGVLPN	356.55	517.88	681.32	784.42
Histone H2A.J	VTIAQGGVLPN	231.79	78.64	366.84	467.48
Histone H2A.J	VTIAQGGVLPN	436.65	264.08	655.15	612.14
Histone H2A.J	VTIAQGGVLPNIQ	76.07	144.29	242.94	179.99
Histone H2A.J	VTIAQGGVLPNIQA	25.76	7.85	49.11	61.88
Histone H2A.J	VTIAQGGVLPNIQA	102.94	42.08	94.08	200.44
Histone H2A.J	VTIAQGGVLPNIQA	38.54	22.48	23.51	24.08
Histone H2A.J	VTIAQGGVLPNIQA	76.95	57.39	70.48	99.39
Histone H2A.J	VTIAQGGVLPNIQAV	25.52	31.73	38.86	66.68
Histone H2A.V	ATIAGGGVIPHIHK	1502.26	1686.15	3956.14	4213.9
Histone H2A.V	GDEELDSLIK	357.64	410.52	1360.14	1677.5
Histone H2A.V	ATIAGGGVIPH	1559.96	1411.51	1692.45	2161.21
Histone H2A.V	AGGK	112.85	127.66	292.73	305.06
Histone H2A.V	AGLQFPVGR	735.36	539.74	1137.2	1255.8
Histone H2A.V	AGLQFPVGR	7391.74	7902.96	31114.53	37885.3
Histone H2A.V	AGLQFPVGR	3719.2	3082.61	12229.63	15352.61
Histone H2A.V	AGLQFPVGR	4353.16	3002.59	18013.83	16481.37
Histone H2A.V	AGLQFPVGR	3005.12	3845.47	14662.96	15630.89
Histone H2A.V	AGLQFPVGR	2346.47	1790.98	6527.64	5785.87
Histone H2A.V	AGLQFPVGR	1232.46	1639.57	5706.37	6792.3
Histone H2A.V	AGLQFPVGR	1543.28	1463.39	4984.86	3335.49
Histone H2A.V	AGLQFPVGR	1229.65	1172.35	3403.93	4897.02

Histone H2A.V	AGLQFPVGR	4384.27	4183.51	14747.87	17040.89
Histone H2A.V	AGLQFPVGR	2105.29	1682.61	3410.78	4449.91
Histone H2A.V	AGLQFPVGR	1505.25	1603.39	2879.59	2988.3
Histone H2A.V	AGLQFPVGR	1057.77	1452.14	2799.67	2329.69
Histone H2A.V	AGLQFPVGR	1179.78	1206.45	2644.64	2135.2
Histone H2A.V	AGLQFPVGR	893.03	813.09	2037.03	1522
Histone H2A.V	AGLQFPVGR	3427.6	4552.18	19059.3	19150.29
Histone H2A.V	AGLQFPVGR	11917.04	10813.63	45916.92	50894.32
Histone H2A.V	AGLQFPVGR	1558.4	1564.95	8416.24	7153.81
Histone H2A.V	AGLQFPVGR	1930.54	968.73	2663.15	3057.85
Histone H2A.V	AGLQFPVGR	3083.15	3902.41	10017.74	10807.33
Histone H2A.V	AGLQFPVGR	1605.11	1524.98	3253.99	2625.91
Histone H2A.V	AGLQFPVGR	1586.56	1230.13	6418.46	4920.93
Histone H2A.V	AGLQFPVGR	7803.66	6567.92	31816.9	34752.98
Histone H2A.V	AGLQFPVGR	1567.22	1119.55	2757.29	4205.61
Histone H2A.V	AGLQFPVGR	2736.28	2852.93	8417.13	8692.62
Histone H2A.V	AGLQFPVGR	5666.01	5635.96	19796.31	21829.52
Histone H2A.V	AGLQFPVGR	706.08	772.74	2590.48	2318.32
Histone H2A.V	AGLQFPVGR	1682.07	1368.73	6871.55	6293.89
Histone H2A.V	AGLQFPVGR	1364.82	2073.96	5169.37	6334.01
Histone H2A.V	AGLQFPVGR	7356.34	7571.09	38671.86	39202.02
Histone H2A.V	AGLQFPVGR	808.66	850.58	2566.07	1891.6
Histone H2A.V	AGLQFPVGR	1076.3	1004.63	1730.47	1973.07
Histone H2A.V	AGLQFPVGR	5221.48	4873.95	19067.57	19826.8
Histone H2A.V	AGLQFPVGR	5211.96	4750.17	19959.64	23151.88
Histone H2A.V	AGLQFPVGR	1975.77	2138.09	8959.97	8677.1
Histone H2A.V	AGLQFPVGR	1265.45	1571.74	5217.66	5867.18
Histone H2A.V	AGLQFPVGR	1474.02	1922.84	4478.49	5591.33
Histone H2A.V	AGLQFPVGR	1088.92	1353.18	2102.07	2730.95
Histone H2A.V	AGLQFPVGR	734.61	826.28	2656.6	3038.88
Histone H2A.V	AGLQFPVGR	1115.46	752.24	2104.38	2955.29
Histone H2A.V	AGLQFPVGR	844.59	705.34	1321.23	1843.46
Histone H2A.V	AGLQFPVGR	989.68	731.88	2136.19	2097.21
Histone H2A.V	AGLQFPVGR	1056.54	1042.06	4313.21	4131.46
Histone H2A.V	AGLQFPVGR	2156.06	1985.76	3015.43	3427.61
Histone H2A.V	AGLQFPVGR	2783.63	2962.21	12465.42	12932.62
Histone H2A.V	AGLQFPVGR	995.36	1022.58	2309.6	2467.86
Histone H2A.V	AGLQFPVGR	965.41	1108.98	1390.06	1732.83
Histone H2A.V	AGLQFPVGR	9080.93	9738.21	42648.28	47032.29
Histone H2A.V	AGLQFPVGR	801.65	479.34	2817.88	2014.33
Histone H2A.V	AGLQFPVGR	946.89	598.13	1303.19	1877.72
Histone H2A.V	AGLQFPVGR	12921.42	13072.49	56983.82	62024.65
Histone H2A.V	AGLQFPVGR	973.93	692.24	1016.59	1318.97
Histone H2A.V	AGLQFPVGR	906.98	1350.04	1381.44	1763.9
Histone H2A.V	AGLQFPVGR	780.34	776.35	2192.85	1573.82
Histone H2A.V	AGLQFPVGR	913.85	670.53	715.07	970.42
Histone H2A.V	AGLQFPVGR	1451.42	1283.56	6770.79	6163.01
Histone H2A.V	AGLQFPVGR	2052.99	1955.73	5889.32	5656.88
Histone H2A.V	AGLQFPVGR	973.28	969.46	1454.53	1594.17
Histone H2A.V	AGLQFPVGR	846.83	483.9	2214.39	1892.43
Histone H2A.V	AGLQFPVGR	460.6	450.89	1486.04	1116.19
Histone H2A.V	AGLQFPVGR	875.51	1161.33	3012.76	3331.16
Histone H2A.V	AGLQFPVGR	411.83	163.42	619.25	875.38
Histone H2A.V	AGLQFPVGR	1027.49	1251.77	2183.95	2801.88
Histone H2A.V	AGLQFPVGR	9789.6	8978.43	47210.65	48156.78
Histone H2A.V	AGLQFPVGR	7337.1	6812.75	30936.86	32724.84
Histone H2A.V	AGLQFPVGR	2191.89	1743.8	8310.88	6655.3
Histone H2A.V	AGLQFPVGR	381.79	578.16	532.32	470.78
Histone H2A.V	AGLQFPVGR	770.94	853.63	1649.32	2090.21
Histone H2A.V	AGLQFPVGR	2561.86	2745.08	9923.76	11767.1
Histone H2A.V	AGLQFPVGR	1072.29	1081.21	2372.08	1835.76
Histone H2A.V	AGLQFPVGR	681.56	960.87	1884.51	1837.95
Histone H2A.V	AGLQFPVGR	11781.22	10827.76	63002.79	65067.61
Histone H2A.V	AGLQFPVGR	634.06	653.99	2292.9	1868.72

Histone H2A.V	AGLQFPVGR	829.97	766.59	3823.66	4723.98
Histone H2A.V	AGLQFPVGR	1583.64	1837.12	6030.03	6618.06
Histone H2A.V	AGLQFPVGR	1463.63	1779.87	1801.83	2111.98
Histone H2A.V	AGLQFPVGR	1990.11	2530.31	7270.88	5859.88
Histone H2A.V	AGLQFPVGR	1177.54	1108.01	1724.2	1887.06
Histone H2A.V	AGLQFPVGR	807.81	934.77	1702.29	1692.95
Histone H2A.V	AGLQFPVGR	1191.31	1024.77	1802.28	1715.42
Histone H2A.V	AGLQFPVGR	15580.76	14908.89	71607.21	74831.87
Histone H2A.V	AGLQFPVGR	770.96	533.16	1331.88	1680.9
Histone H2A.V	AGLQFPVGR	800.46	384.67	1016.38	1345.14
Histone H2A.V	AGLQFPVGR	728.49	1217.84	1820.1	2499.85
Histone H2A.V	AGLQFPVGR	5500.73	6113.81	22880.82	28990.17
Histone H2A.V	AGLQFPVGR	13342.21	14477.4	69294.52	77085.73
Histone H2A.V	AGLQFPVGR	963.56	866.55	1421.16	1210.32
Histone H2A.V	AGLQFPVGR	659.15	515.27	924.45	1211.15
Histone H2A.V	AGLQFPVGR	1169.19	1720.32	4390.78	5177.26
Histone H2A.V	AGLQFPVGR	1835.38	1965.98	5807.36	7331.35
Histone H2A.V	AGLQFPVGR	750.79	717.55	2390.72	1899.98
Histone H2A.V	AGLQFPVGR	4341.52	3939.4	16395.2	19115.57
Histone H2A.V	AGLQFPVGR	3840.33	2711.39	13979.03	14060.98
Histone H2A.V	AGLQFPVGR	13267.3	12410.55	64418.72	68188.72
Histone H2A.V	AGLQFPVGR	1669.13	2646.7	5934.27	9711.38
Histone H2A.V	AGLQFPVGR	1256.66	845.09	3589.58	3030.78
Histone H2A.V	AGLQFPVGR	22173.4	23357.7	113914.5	117779.3
Histone H2A.V	AGLQFPVGR	615.78	1290.12	2115.03	2124.18
Histone H2A.V	AGLQFPVGR	4396.96	3355.39	8858.52	9554.16
Histone H2A.V	AGLQFPVGR	2323.51	2144.22	7595.02	5547.48
Histone H2A.V	AGLQFPVGR	771.65	783.48	1818.86	1461.33
Histone H2A.V	AGLQFPVGR	755.07	613.21	1845.28	1443.02
Histone H2A.V	AGLQFPVGR	1337.63	1462.02	1498.03	2430.35
Histone H2A.V	AGLQFPVGR	1787.99	1648.17	2500.27	2807.22
Histone H2A.V	AGLQFPVGR	1206.66	891.01	1926.46	1862.94
Histone H2A.V	AGLQFPVGR	15965.26	17347.55	82594.47	85498.15
Histone H2A.V	AGLQFPVGR	2950.12	2896.52	7923.98	8552.25
Histone H2A.V	AGLQFPVGR	1914.91	1768.74	3092.14	3497.98
Histone H2A.V	AGLQFPVGR	25003.83	27547.2	133263	132321.3
Histone H2A.V	AGLQFPVGR	776.19	335.35	1311.11	830.06
Histone H2A.V	AGLQFPVGR	1078.53	890.24	1530.31	1802.05
Histone H2A.V	AGLQFPVGR	621.31	530.86	664.59	649.84
Histone H2A.V	AGLQFPVGR	974.47	1596.7	3559.94	2425.87
Histone H2A.V	AGLQFPVGR	1512.85	1810.52	8615.78	8315.48
Histone H2A.V	AGLQFPVGR	18520.47	21280.89	94612.43	100476.7
Histone H2A.V	AGLQFPVGR	688.78	673.05	1096.26	1126.67
Histone H2A.V	AGLQFPVGR	461.72	340.39	618.4	920.2
Histone H2A.V	AGLQFPVGR	1259.02	1143.55	2220.91	2744.3
Histone H2A.V	AGLQFPVGR	2654.47	3382.38	4919.8	5595.68
Histone H2A.V	AGLQFPVGR	357.04	469.41	1002.77	1192.24
Histone H2A.V	AGLQFPVGR	1270.08	901.76	1428.42	1014.2
Histone H2A.V	AGLQFPVGR	21202.08	18045.75	97054.73	97989.67
Histone H2A.V	AGLQFPVGR	1022	624.66	1789.91	1699.53
Histone H2A.V	AGLQFPVGR	6274.58	6892.98	33468.67	34576.11
Histone H2A.V	AGLQFPVGR	1949.98	1814.34	5335.88	4130.09
Histone H2A.V	AGLQFPVGR	408.49	493.33	854.2	937.65
Histone H2A.V	AGLQFPVGR	953.99	748.01	1948.09	1932.83
Histone H2A.V	AGLQFPVGR	7506.25	6802.85	15275.03	16186.78
Histone H2A.V	AGLQFPVGR	25508.24	26861.2	137211.1	150031.6
Histone H2A.V	AGLQFPVGR	839.08	1142.89	2037.25	2143.55
Histone H2A.V	AGLQFPVGR	204.16	254.95	1958.89	898.18
Histone H2A.V	AGLQFPVGR	9390.52	9364.29	40276.81	43372.96
Histone H2A.V	AGLQFPVGR	781.62	755.91	1178.04	1225.45
Histone H2A.V	AGLQFPVGR	359.77	199.84	285.26	343.68
Histone H2A.V	AGLQFPVGR	1064.99	845.5	2463.35	2116.21
Histone H2A.V	AGLQFPVGR	12898.72	12549.22	60824.75	66481.3
Histone H2A.V	AGLQFPVGR	717.32	938.89	1776.93	1638.44

Histone H2A.V	AGLQFPVGR	1512.12	1244.2	2112.28	2589.62
Histone H2A.V	AGLQFPVGR	3754.24	4727.03	5169.87	7731.69
Histone H2A.V	AGLQFPVGR	730.17	946.19	2379.41	2757.84
Histone H2A.V	AGLQFPVGR	1010.57	1328.08	1981.97	1578.95
Histone H2A.V	AGLQFPVGR	594.64	931.85	1298.1	1376.65
Histone H2A.V	AGLQFPVGR	5684.85	6008.46	10502.22	14303.33
Histone H2A.V	AGLQFPVGR	1499.12	2103.35	6733.66	4358.06
Histone H2A.V	AGLQFPVGR	10952.99	11591.67	47877.18	49740.2
Histone H2A.V	AGLQFPVGR	1930.09	2821.77	2753.96	2318.96
Histone H2A.V	AGLQFPVGR	32037.25	35319.58	180394.8	177293.2
Histone H2A.V	AGLQFPVGR	6193.2	8550.31	11781.76	14807.87
Histone H2A.V	AGLQFPVGR	1314.71	1279.9	2718.63	2826.48
Histone H2A.V	AGLQFPVGR	1116.67	1696.97	685.15	644.61
Histone H2A.V	AGLQFPVGR	8329.98	7760.7	12479.6	16636.78
Histone H2A.V	AGLQFPVGR	881.92	1484.84	1720.03	2290.7
Histone H2A.V	AGLQFPVGR	778.46	536.83	1495.14	1886.07
Histone H2A.V	AGLQFPVGR	459.93	198.27	1272.39	1253.57
Histone H2A.V	AGLQFPVGR	17775.7	16564.34	95128.73	90015.13
Histone H2A.V	AGLQFPVGR	538.35	429.01	1059.91	969.96
Histone H2A.V	AGLQFPVGR	959.21	1828.02	2811.16	3161.15
Histone H2A.V	AGLQFPVGR	307.29	278.98	392.5	897.91
Histone H2A.V	AGLQFPVGR	509.98	785.59	1929.15	706.56
Histone H2A.V	AGLQFPVGR	278.74	546.54	1197.66	1129.15
Histone H2A.V	AGLQFPVGR	1255.47	2664.7	10602.54	2080.34
Histone H2A.V	AGLQFPVGR	883.09	837.78	692.62	1080.91
Histone H2A.V	AGLQFPVGR	8340.85	9878.16	12958.73	17024.35
Histone H2A.V	AGLQFPVGR	7429.04	9324.5	12678.39	16348.85
Histone H2A.V	AGLQFPVGR	436.23	305.23	1174.46	967.27
Histone H2A.V	AGLQFPVGR	1440.41	1413.68	3246.5	2125.68
Histone H2A.V	AGLQFPVGR	769.75	758.75	2889.53	693.74
Histone H2A.V	AGLQFPVGR	765.55	1173.51	2653.85	1286.41
Histone H2A.V	AGLQFPVGR	799.88	1505.24	2341.31	1378.18
Histone H2A.V	AGLQFPVGR	9134.02	11195.56	41371.96	53640.86
Histone H2A.V	AGLQFPVGR	358.62	313.44	1052.6	581.51
Histone H2A.V	AGLQFPVGR	658.77	1191.15	3183.2	1092.5
Histone H2A.V	AGLQFPVGR	425.61	290.65	658.22	350.91
Histone H2A.V	AGLQFPVGR	438.12	118.68	183.07	477.05
Histone H2A.V	AGLQFPVGR	535.37	830.33	1005.83	960.25
Histone H2A.V	AGLQFPVGR	397.55	510.46	1270.11	1211.33
Histone H2A.V	AGLQFPVGR	24910.02	28091.79	141081.8	137110
Histone H2A.V	AGLQFPVGR	7062.89	9223.16	15097.36	16468.32
Histone H2A.V	AGLQFPVGR	968.2	620.37	709.89	709.61
Histone H2A.V	AGLQFPVGR	2521.6	3743.75	13966.48	16544.49
Histone H2A.V	AGLQFPVGR	2521.6	3743.75	13966.48	16544.49
Histone H2A.V	AGLQFPVGR	406.9	543.67	1082.3	818.21
Histone H2A.V	AGLQFPVGR	840.21	1031.86	3057.41	3192.68
Histone H2A.V	AGLQFPVGR	668.36	350.57	421.08	623.23
Histone H2A.V	AGLQFPVGR	1418.3	1683.46	1098.86	958.1
Histone H2A.V	AGLQFPVGR	2912.07	3038.75	1133.96	1269.9
Histone H2A.V	AGLQFPVGR	178.05	281.42	745.2	320.37
Histone H2A.V	AGLQFPVGR	852.94	1156.96	5918.07	700.19
Histone H2A.V	AGLQFPVGR	16367.95	19583.16	72920.64	82336.19
Histone H2A.V	AGLQFPVGR	488.76	541.34	685.19	1306.41
Histone H2A.V	AGLQFPVGR	23470.75	25820.33	120423.2	119929.1
Histone H2A.V	AGLQFPVGR	509.98	785.59	1929.15	706.56
Histone H2A.V	AGLQFPVGR	888.88	691.45	2165.07	2089.24
Histone H2A.V	AGLQFPVGR	769.75	758.75	2889.53	693.74
Histone H2A.V	AGLQFPVGR	28765.71	27264.85	157592.2	146114.7
Histone H2A.V	AGLQFPVGR	1207.97	1080.54	5737.75	6853.03
Histone H2A.V	AGLQFPVGR	4045.77	4648.2	2469.06	2154.7
Histone H2A.V	AGLQFPVGR	31596.32	30159.57	175405.2	170862.1
Histone H2A.V	AGLQFPVGR	31384.23	39943.32	198050.1	194084.3
Histone H2A.V	AGLQFPVGR	333.11	281.33	181.56	196.3
Histone H2A.V	AGLQFPVGR	658.77	1191.15	3183.2	1092.5

Histone H2A.V	AGLQFPVGR	26360.24	26302.61	145444.1	146949.2
Histone H2A.V	AGLQFPVGR	28568.85	31075.36	159389.5	154730
Histone H2A.V	AGLQFPVGR	30331.6	30643.17	171915	166589.2
Histone H2A.V	AGLQFPVGR	769.12	501.57	1093.09	1370.01
Histone H2A.V	AGLQFPVGR	33743.25	33579.08	190507.8	186818.8
Histone H2A.V	AGLQFPVGR	30236.94	30207.49	160197.4	157297.6
Histone H2A.V	AGLQFPVGR	52526.38	52207.16	281929.4	304593.4
Histone H2A.V	AGLQFPVGR	1354.4	1932.75	3841.77	2597.42
Histone H2A.V	AGLQFPVGR	58565.43	53434.29	298827.4	324923.5
Histone H2A.V	AGLQFPVGR	958.12	552.94	1210.06	859.72
Histone H2A.V	AGLQFPVGR	486.55	334.21	460.71	960.71
Histone H2A.V	AGLQFPVGR	1193.06	2128.14	5086.7	2653.11
Histone H2A.V	AGLQFPVGR	1195.6	1983.61	4354.74	3935.23
Histone H2A.V	AGLQFPVGR	179.5	138.09	170.7	16.57
Histone H2A.V	AGLQFPVGR	571.42	566.22	3034.2	2546.55
Histone H2A.V	AGLQFPVGR	31542.75	30133.07	179465.5	172668.1
Histone H2A.V	AGLQFPVGR	5621.18	6022.58	28229.04	26278.96
Histone H2A.V	AGLQFPVGR	231.27	129.64	398.74	159.37
Histone H2A.V	AGLQFPVGR	408.3	512.34	1191.24	1045.61
Histone H2A.V	AGLQFPVGR	59862.48	57948.53	352305.3	381336.6
Histone H2A.V	AGLQFPVGR	561.55	584.14	1333.07	1520.52
Histone H2A.V	AGLQFPVGR	256.34	206.44	337.55	314.07
Histone H2A.V	AGLQFPVGR	789.17	967.8	2796.5	2654.17
Histone H2A.V	AGLQFPVGR	5621.18	6022.58	28229.04	26278.96
Histone H2A.V	AGLQFPVGR	305.85	421.07	1028.26	706.26
Histone H2A.V	AGLQFPVGR	33825.63	35865.82	195702.3	183745.3
Histone H2A.V	AGLQFPVGR	356.25	547.45	868.27	1357.04
Histone H2A.V	AGLQFPVGR	958.12	552.94	1210.06	859.72
Histone H2A.V	AGLQFPVGR	30350.76	33111.27	186082.3	169240.5
Histone H2A.V	AGLQFPVGR	956.41	934.7	2065.4	2277.84
Histone H2A.V	AGLQFPVGR	872.12	1708.26	4692.79	5115.06
Histone H2A.V	AGLQFPVGR	614.01	613.72	688.21	1293.43
Histone H2A.V	AGLQFPVGR	381.98	558.93	685.14	924.91
Histone H2A.V	AGLQFPVGR	204.06	265.09	181.36	247.95
Histone H2A.V	AGLQFPVGR	799.88	1505.24	2341.31	1378.18
Histone H2A.V	AGLQFPVGR	408.02	433.59	1311.1	1082.41
Histone H2A.V	AGLQFPVGR	1207.97	1080.54	5737.75	6853.03
Histone H2A.V	AGLQFPVGR	506.73	362.05	871.03	1321.75
Histone H2A.V	AGLQFPVGR	756.81	866.76	1424.1	987.71
Histone H2A.V	AGLQFPVGR	496.18	340.55	484.39	1051.85
Histone H2A.V	AGLQFPVGR	669.57	337.37	1175.99	1326.11
Histone H2A.V	AGLQFPVGR	305.24	480.89	571.52	414.08
Histone H2A.V	AGLQFPVGR	505.84	1088.23	909.13	1098.16
Histone H2A.V	AGLQFPVGR	1001.85	487.22	1056.17	1783.61
Histone H2A.V	AGLQFPVGR	1416.92	1075.45	1659.77	2608.41
Histone H2A.V	AGLQFPVGR	1932.74	2879.5	1164.89	1161.08
Histone H2A.V	AGLQFPVGR	3013.27	3785.06	12302.32	13726.99
Histone H2A.V	AGLQFPVGR	953.46	1332.51	2184.47	2191.61
Histone H2A.V	AGLQFPVGR	153.13	190.41	232.33	427.19
Histone H2A.V	AGLQFPVGR	304.28	575.71	937.6	823.44
Histone H2A.V	AGLQFPVGR	1255.47	2664.7	10602.54	2080.34
Histone H2A.V	AGLQFPVGR	4884.28	5422.52	2794.05	2499.44
Histone H2A.V	AGLQFPVGR	3013.27	3785.06	12302.32	13726.99
Histone H2A.V	AGLQFPVGR	1833.78	1698.45	3409.3	3955.73
Histone H2A.V	AGLQFPVGR	535.03	438.15	726.65	1141.12
Histone H2A.V	AGLQFPVGR	409.55	388.09	1003.16	813.93
Histone H2A.V	AGLQFPVGR	651.37	645.75	1306.89	1501.44
Histone H2A.V	AGLQFPVGR	506.73	362.05	871.03	1321.75
Histone H2A.V	AGLQFPVGR	496.18	340.55	484.39	1051.85
Histone H2A.V	AGLQFPVGR	230.21	234.19	337.37	264.51
Histone H2A.V	AGLQFPVGR	1663.44	2571.04	6027.59	9178.14
Histone H2A.V	AGLQFPVGR	523.28	535.42	1170.39	1428.15
Histone H2A.V	AGLQFPVGR	334.77	116.6	664.71	792.52
Histone H2A.V	AGLQFPVGR	383.11	447.31	723.55	706.98

Histone H2A.V	AGLQFPVGR	12694.93	12683.01	52399.88	61492.76
Histone H2A.V	ATIAGGGVIPHIHK	625.74	1156.62	2671.66	2176.83
Histone H2A.V	ATIAGGGVIPHIHK	3192.06	2716.31	9037.32	10270.15
Histone H2A.V	ATIAGGGVIPHIHK	1600.38	1885.77	3721.58	3521.86
Histone H2A.V	ATIAGGGVIPHIHK	2144.65	1516.99	3799.51	4263.05
Histone H2A.V	ATIAGGGVIPHIHK	5047.6	5350.32	8745.87	10039.22
Histone H2A.V	ATIAGGGVIPHIHK	2037.63	1665.66	3911.89	3687.03
Histone H2A.V	ATIAGGGVIPHIHK	2690.86	2446.24	3329.36	3115
Histone H2A.V	ATIAGGGVIPHIHK	1945.45	2261.97	3900.53	3785.59
Histone H2A.V	ATIAGGGVIPHIHK	6336.7	4692.18	11471.79	9511.1
Histone H2A.V	ATIAGGGVIPHIHK	1991.83	1616.08	2915.23	3374.47
Histone H2A.V	ATIAGGGVIPHIHK	3064.41	2774.8	5920.01	6741.72
Histone H2A.V	DSLIK	1116.61	1064.15	1511.15	2352
Histone H2A.V	GDEELDSLIK	601.43	474.07	1099.92	1500.78
Histone H2A.V	GDEELDSLIK	2412.05	3184.35	7306.71	7992.74
Histone H2A.V	GDEELDSLIK	5741.47	5520.86	17660.19	22177.41
Histone H2A.V	GDEELDSLIK	5569.36	5290.75	14567.5	17206.21
Histone H2A.V	GDEELDSLIK	2784.9	2624.22	4446.49	5985.54
Histone H2A.V	GDEELDSLIK	1469.74	1601.77	2398.24	3466.03
Histone H2A.V	GDEELDSLIK	6912.77	7778.19	30318.64	33961.05
Histone H2A.V	GDEELDSLIK	1706.38	2051.76	3632.49	3055.51
Histone H2A.V	GLQFPVGR	5465.72	3615.26	4160.52	5161
Histone H2A.V	GLQFPVGR	3623.58	3806.48	8633.53	11317.45
Histone H2A.V	GLQFPVGR	5458.29	4774.93	13576.62	15270.13
Histone H2A.V	HLQLAIR	3406.51	2805.59	9845.33	9587
Histone H2A.V	HLQLAIR	4891.35	6532.1	17241.07	18515.23
Histone H2A.V	HLQLAIR	2459.32	2024.44	4086.24	6509.23
Histone H2A.V	HLQLAIR	7413.79	6682.28	19822.83	25349.53
Histone H2A.V	HLQLAIR	495.22	434.72	935.64	881.85
Histone H2A.V	HLQLAIR	282	224.26	638.52	477.65
Histone H2A.V	HLQLAIR	1530.69	1537.48	3653.3	3427
Histone H2A.V	HLQLAIR	3931.58	4653.8	12025.64	13178.84
Histone H2A.V	HLQLAIR	1002.23	982.03	3598.64	4772.14
Histone H2A.V	HLQLAIR	1935.65	1740.56	4303.7	3262.49
Histone H2A.V	HLQLAIR	15328.33	16445.01	49841.69	56921.78
Histone H2A.V	HLQLAIR	563.83	465.08	405.85	895.7
Histone H2A.V	HLQLAIR	1388.91	1608.16	3389.16	3857.72
Histone H2A.V	HLQLAIR	10528.26	9191.79	30012.6	33431.36
Histone H2A.V	HLQLAIR	669.97	723.28	808.02	1712.34
Histone H2A.V	HLQLAIR	559.96	634.81	853.33	941.75
Histone H2A.V	HLQLAIR	384.9	270.38	533.4	793.71
Histone H2A.V	HLQLAIR	1740.16	1160.57	2860.4	3819.71
Histone H2A.V	HLQLAIR	781.09	489.86	936.67	1419
Histone H2A.V	HLQLAIR	887.97	781.1	765.9	1651.08
Histone H2A.V	HLQLAIR	2482.4	1978.24	3467.88	4010.84
Histone H2A.V	HLQLAIR	610.06	791.49	1430.07	2167.54
Histone H2A.V	HLQLAIR	14199.58	13601.52	47619.1	56553.43
Histone H2A.V	HLQLAIR	963.2	1540.38	2557.8	2904.39
Histone H2A.V	HLQLAIR	690.08	758.18	1082.08	919.83
Histone H2A.V	HLQLAIR	631.41	916.33	1080.23	1045.42
Histone H2A.V	HLQLAIR	308.12	196.69	630.41	589.88
Histone H2A.V	HLQLAIR	513.5	330.95	895.5	1183.96
Histone H2A.V	HLQLAIR	573.38	585.5	743.35	562.86
Histone H2A.V	HLQLAIR	537.34	528.6	658.33	1329.39
Histone H2A.V	LDSL	357.3	444.42	376.14	235.28
Histone H2A.V	LDSL	382.05	658.05	515.6	643.4
Histone H2A.V	LIGK	3497.2	4271.32	5463.79	5805.31
Histone H2A.V	LIGK	6358.35	8191.63	19930.68	27872.12
Histone H2A.V	LIGK	5627.84	5364.58	18307.75	21015.58
Histone H2A.V	LIGK	4093.22	3045.08	7609.02	8332.05
Histone H2A.V	LIGK	2955.88	3816.51	8510.03	11071
Histone H2A.V	LIGK	1741.15	1701.46	2482.79	3844.17
Histone H2A.V	LIKA	1307.15	1601.51	2368.98	4199.33
Histone H2A.V	LIKA	2139.52	2555.78	5516.39	5965.56

Histone H2A.V	LIKA	2452.19	2516.46	6687.89	7159.26
Histone H2A.V	LIKA	2478.27	3662.86	5467.4	7682.72
Histone H2A.V	RAGLQFPVGR	842.15	2329.3	10836.33	1053.15
Histone H2A.V	RAGLQFPVGR	571.41	779.52	993.43	409.63
Histone H2A.V	RITPRHLQLAIR	661.13	851.58	1834.17	962.91
Histone H2A.V	RITPRHLQLAIR	661.13	851.58	1834.17	962.91
Histone H2A.V	SLIGK	4603.28	5738	8909.5	12586.64
Histone H2A.V	SLIGK	3379.39	3676.75	10520.34	15298.75
Histone H2A.V	SLIGK	2271.88	2883.21	5749	8369.79
Histone H2A.V	SLIGK	2753.12	2998.01	8093.61	9673.51
Histone H2A.V	SLIGK	2456.19	2227.74	4360.39	4192.61
Histone H2A.V	SLIGK	9655.84	9109.04	17994.91	21970.2
Histone H2A.V	SLIGK	676.71	801.94	1519.87	1799.44
Histone H2A.V	SLIGK	5495.46	5251.42	8433.31	10103.3
Histone H2A.V	SLIGK	6026.24	7598.65	18193.98	25070.11
Histone H2A.V	SLIGK	4421.56	4329.99	4652.49	5512.69
Histone H2A.V	SLIGK	6330.68	5924.92	12161.75	14789.7
Histone H2A.V	SLIGK	280.05	416.73	1284.15	1378.46
Histone H2A.V	SLIGK	1788.66	2220.59	4075.22	4606.11
V-type proton ATPase subunit F	GMFTAEDLR	1030.94	911.18	1998.24	1595.68
V-type proton ATPase subunit F	AEDLR	691.52	508.75	383.74	673.37
V-type proton ATPase subunit F	EIPSK	437.91	139.9	410.94	566.14
V-type proton ATPase subunit F	GMFTAEDLR	2324.43	2797.9	3978.49	5194.1
V-type proton ATPase subunit F	GMFTAEDLR	685.19	496.38	1231.77	1313.25
V-type proton ATPase subunit F	IGII	1296.99	1434.54	5346.49	5340.23
V-type proton ATPase subunit F	IGII	667.11	261.48	2492.45	2908.78
V-type proton ATPase subunit F	IGII	3314.76	3998.01	22099.16	20825.3
V-type proton ATPase subunit F	IGII	3659.85	3414.84	18140.3	21295.9
V-type proton ATPase subunit F	IGII	332.65	326.7	492.06	841.9
Galectin-3	GNDVAFHFNPR	1707.37	1953.65	3269.34	2840.6
Galectin-3	IALDFQR	1938.24	1697.66	3225.27	4037.03
Galectin-3	VAVNDAHLLQYNHR	636.23	865.42	2357.7	3225.41
Galectin-3	VIVCNTK	732.96	1202.26	925.53	1114.38
Galectin-3	LDNNWGR	2340.04	2851.25	3409.78	4893.78
Galectin-3	LDNNWGREER	1188.41	1310.91	2764.69	2458.5
Galectin-3	LNEISK	2852.15	2043.94	3809.84	3855.56
Galectin-3	GNDVAFHFNPR	2371.69	2490.81	4209.46	5156.65
Galectin-3	GNDVAFHFNPR	1989.42	1853.54	2879.42	3102
Galectin-3	GNDVAFHFNPR	811.51	887.89	1508.12	1677.37
Galectin-3	GNDVAFHFNPR	6064.74	6561.63	12543.78	16612.59
Galectin-3	GNDVAFHFNPR	663.75	699.31	2335.51	1465.46
Galectin-3	GNDVAFHFNPR	4372.89	4776.73	8529.38	9403.49
Galectin-3	GNDVAFHFNPR	1543.45	1234.26	2329.38	2636.31
Galectin-3	GNDVAFHFNPR	3884.62	3867.07	6594.42	7510.22
Galectin-3	GNDVAFHFNPR	986.58	1464.19	2145.31	2254.33
Galectin-3	GNDVAFHFNPR	534.85	1200.24	1272.8	873.63
Galectin-3	GNDVAFHFNPR	1106.53	2166.97	2897.74	3196.91
Galectin-3	GNDVAFHFNPR	9031.67	9392.73	11780.77	12799.15
Galectin-3	GNDVAFHFNPR	1109.83	1308.15	1593.17	1933.53
Galectin-3	GNDVAFHFNPR	788.34	943.42	1133.03	1396.13
Galectin-3	IALDFQR	809.74	1276.11	2097.21	2900.03
Galectin-3	IALDFQR	12119.15	13717.63	29925.46	35107.63
Galectin-3	IALDFQR	330.7	518.84	834.67	970.75
Galectin-3	IALDFQR	1306.64	1651.25	4271.6	4668.96
Galectin-3	IALDFQR	1024.44	915.56	2603.35	3342.54
Galectin-3	IALDFQR	1788.76	1572.19	2586.24	2785.72
Galectin-3	IALDFQR	934	807.55	2001.12	2861.66
Galectin-3	IALDFQR	1533.36	1485.61	2272.88	2736.62
Galectin-3	IALDFQR	410.84	686.76	1170.37	2363.95
Galectin-3	IALDFQR	356.77	496.3	949.94	683.25
Galectin-3	IALDFQR	3180.76	2875.63	5931.5	7776.64
Galectin-3	IALDFQR	406.63	677.37	905.93	1292.5
Galectin-3	IALDFQR	848.17	1415.81	2013.62	1818.77
Galectin-3	IALDFQR	6994.54	4911.56	10496.01	12038.38

Galectin-3	IALDFQR	2022.17	2448.42	4076.71	5581.75
Galectin-3	IALDFQR	4032.78	4335.85	8287.53	8728.16
Galectin-3	IALDFQR	301.12	249.77	583.48	433.13
Galectin-3	ITIL	114.69	158.48	37.77	113.82
Galectin-3	LDNNWGR	1303.11	1467.84	2932.35	4309.26
Galectin-3	LDNNWGREER	1414.05	614.08	2046.89	2067.62
Galectin-3	LNEISK	1090.18	803.57	2529.94	2521.15
Galectin-3	LNEISK	2426.77	2687.23	3918.57	4949.16
Galectin-3	LNEISK	2504.78	2533.09	5300.13	8087.77
Galectin-3	LNEISK	3733.46	4120.62	5722.84	6837.65
Galectin-3	VAVNDAHLLQYNHR	2616.18	3335.3	5899.82	5991.57
Galectin-3	VIVCNTK	1704.6	2333.86	3088.54	3478.04
Histone H2B type 1-C/E/F/G/I	AMGIMNSFVNDIFER	26.57	34.56	54.85	29.13
Histone H2B type 1-C/E/F/G/I	ESYSVYVYK	931.14	1409.25	4427.87	4055
Histone H2B type 1-C/E/F/G/I	ESYSVYVYKVLK	410.41	302.95	1381.96	1108.93
Histone H2B type 1-C/E/F/G/I	HAVSEGTK	310.93	344.41	1503.84	1827.56
Histone H2B type 1-C/E/F/G/I	IAGEASR	165.44	144.04	353.21	503.41
Histone H2B type 1-C/E/F/G/I	KESYSVYVYK	660.98	1079.41	2479.03	3840.86
Histone H2B type 1-C/E/F/G/I	LLLPGELAK	489.85	326.94	1180.36	1360.34
Histone H2B type 1-C/E/F/G/I	QVHPDTGISSK	4048	3789.63	14453.91	15354.33
Histone H2B type 1-C/E/F/G/I	STITSR	420.06	413.54	1088.58	1343.96
Histone H2B type 1-C/E/F/G/I	SAPAPK	223.19	289.49	978.76	1133.44
Histone H2B type 1-C/E/F/G/I	EIQTAVR	938.21	498.52	783.88	1574.28
Histone H2B type 1-C/E/F/G/I	PEPAKSAPAPK	665.2	875.41	2395.74	3114.4
Histone H2B type 1-C/E/F/G/I	LAHYNK	471.05	695.36	1375.76	2331.67
Histone H2B type 1-C/E/F/G/I	SFVNDIFER	277.81	637.9	724.07	654.02
Histone H2B type 1-C/E/F/G/I	AVTKYTSSK	402.55	441.78	926.07	516.03
Histone H2B type 1-C/E/F/G/I	SRKESYSVYVYK	277.97	622.59	1001.96	694.44
Histone H2B type 1-C/E/F/G/I	AMGIMNSF	195.49	47.46	127.29	113.32
Histone H2B type 1-C/E/F/G/I	ESYSVYVY	552.51	626.35	873.58	821.48
Histone H2B type 1-C/E/F/G/I	IAGEASRL	846.05	666.97	1033.18	1010.71
Histone H2B type 1-C/E/F/G/I	LLLPGEL	49.28	24.75	84.59	58.53
Histone H2B type 1-C/E/F/G/I	PGELAK	359.32	244.38	636.56	848.48
Histone H2B type 1-C/E/F/G/I	SVYVYK	3918.57	5619.97	7470.13	8961.18
Histone H2B type 1-C/E/F/G/I	AMGIMNSF	214.49	85.76	184.4	273.87
Histone H2B type 1-C/E/F/G/I	AMGIMNSF	188.54	96.31	289.39	332.57
Histone H2B type 1-C/E/F/G/I	AMGIMNSF	150.04	69.84	144.38	251.1
Histone H2B type 1-C/E/F/G/I	AMGIMNSFVNDIFER	60.95	42.44	70.82	49.81
Histone H2B type 1-C/E/F/G/I	AMGIMNSFVNDIFER	473.02	181.24	790.04	831.92
Histone H2B type 1-C/E/F/G/I	AMGIMNSFVNDIFER	231.03	153.67	487.6	436.28
Histone H2B type 1-C/E/F/G/I	AMGIMNSFVNDIFER	230.24	231.61	465.05	309.39
Histone H2B type 1-C/E/F/G/I	AMGIMNSFVNDIFER	384.36	323.61	455.5	809.47
Histone H2B type 1-C/E/F/G/I	AMGIMNSFVNDIFER	358.47	328.24	320.97	599.4
Histone H2B type 1-C/E/F/G/I	AMGIMNSFVNDIFER	203.85	285.58	445.2	855.16
Histone H2B type 1-C/E/F/G/I	AMGIMNSFVNDIFER	282.97	127.9	295.49	162.69
Histone H2B type 1-C/E/F/G/I	AMGIMNSFVNDIFER	589.82	344.54	1326.65	950.75
Histone H2B type 1-C/E/F/G/I	AMGIMNSFVNDIFER	333.41	250.87	410.41	361.69
Histone H2B type 1-C/E/F/G/I	AMGIMNSFVNDIFER	306.45	361.49	143.72	429.24
Histone H2B type 1-C/E/F/G/I	AMGIMNSFVNDIFER	86.6	60.72	29.96	81.93
Histone H2B type 1-C/E/F/G/I	AMGIMNSFVNDIFER	90.01	43.6	66.43	47.8
Histone H2B type 1-C/E/F/G/I	AMGIMNSFVNDIFER	461.87	431.99	341.72	537
Histone H2B type 1-C/E/F/G/I	AMGIMNSFVNDIFER	102.44	91.98	180.87	209.04
Histone H2B type 1-C/E/F/G/I	AMGIMNSFVNDIFER	77.12	39.8	301.59	163.74
Histone H2B type 1-C/E/F/G/I	AMGIMNSFVNDIFER	178.47	239.63	257.83	331.22
Histone H2B type 1-C/E/F/G/I	AMGIMNSFVNDIFER	152.87	215.67	260.51	295.43
Histone H2B type 1-C/E/F/G/I	AMGIMNSFVNDIFER	230.99	157.56	295.28	113.15
Histone H2B type 1-C/E/F/G/I	AMGIMNSFVNDIFER	256.83	157.88	184.93	349.02
Histone H2B type 1-C/E/F/G/I	AMGIMNSFVNDIFER	788.19	639.04	1350.44	1342.32
Histone H2B type 1-C/E/F/G/I	AMGIMNSFVNDIFER	127.02	216.62	308.25	420.83
Histone H2B type 1-C/E/F/G/I	AMGIMNSFVNDIFER	25.17	66.2	37.2	49.52
Histone H2B type 1-C/E/F/G/I	AMGIMNSFVNDIFER	101.76	159.5	35.01	203.7
Histone H2B type 1-C/E/F/G/I	AMGIMNSFVNDIFER	115.63	65.35	173.52	81.04
Histone H2B type 1-C/E/F/G/I	AMGIMNSFVNDIFER	179.78	110.08	278.62	162.06
Histone H2B type 1-C/E/F/G/I	AMGIMNSFVNDIFER	788.19	639.04	1350.44	1342.32

Histone H2B type 1-C/E/F/G/I	AMGIMNSFVNDIFER	754.6	660.11	1441.77	1510.51
Histone H2B type 1-C/E/F/G/I	AMGIMNSFVNDIFER	153.41	162.44	338.24	391.47
Histone H2B type 1-C/E/F/G/I	AMGIMNSFVNDIFER	754.6	660.11	1441.77	1510.51
Histone H2B type 1-C/E/F/G/I	AVTK	148.31	134.21	477.05	549.01
Histone H2B type 1-C/E/F/G/I	AVTKYTSSK	654.17	582.49	968.61	960.45
Histone H2B type 1-C/E/F/G/I	AVTKYTSSK	843.45	817.51	1202.25	1390.58
Histone H2B type 1-C/E/F/G/I	AVTKYTSSK	331.18	472.06	912.86	822.99
Histone H2B type 1-C/E/F/G/I	AVTKYTSSK	473.37	359.48	907.7	1077.01
Histone H2B type 1-C/E/F/G/I	DIFER	1593.21	2487.58	2488.22	3345.97
Histone H2B type 1-C/E/F/G/I	EIQTAVR	604.17	628.08	1449.76	2002.3
Histone H2B type 1-C/E/F/G/I	EIQTAVR	560.64	461.38	653.58	630.16
Histone H2B type 1-C/E/F/G/I	EIQTAVR	411.04	241.48	590.79	801.46
Histone H2B type 1-C/E/F/G/I	ESYSVYVY	775.93	680.09	960.49	835.08
Histone H2B type 1-C/E/F/G/I	ESYSVYVYK	1076.52	663.49	2062.75	2565.5
Histone H2B type 1-C/E/F/G/I	ESYSVYVYK	382.29	528.07	1837.97	1716.51
Histone H2B type 1-C/E/F/G/I	ESYSVYVYK	733.98	463.23	1256.51	1199.2
Histone H2B type 1-C/E/F/G/I	ESYSVYVYK	7323.28	8177.34	19069.2	22569.74
Histone H2B type 1-C/E/F/G/I	ESYSVYVYK	3424.66	3672.25	7545.23	9604.92
Histone H2B type 1-C/E/F/G/I	ESYSVYVYK	2352.45	2689.5	5036.46	6547.51
Histone H2B type 1-C/E/F/G/I	ESYSVYVYK	177.28	357.57	811.03	965.94
Histone H2B type 1-C/E/F/G/I	ESYSVYVYK	4040.11	5739	11296.05	14499.39
Histone H2B type 1-C/E/F/G/I	ESYSVYVYK	5149.31	4131.55	11376.26	14479.84
Histone H2B type 1-C/E/F/G/I	ESYSVYVYK	3890.58	3917.3	10417.68	10759.36
Histone H2B type 1-C/E/F/G/I	ESYSVYVYK	9540.3	11374.57	32570.53	38824.03
Histone H2B type 1-C/E/F/G/I	ESYSVYVYK	1689.37	1817.03	3551.49	3672.69
Histone H2B type 1-C/E/F/G/I	ESYSVYVYK	2546.99	1660.43	3899.39	3464.57
Histone H2B type 1-C/E/F/G/I	ESYSVYVYK	500.03	491.69	1823.41	1979.59
Histone H2B type 1-C/E/F/G/I	ESYSVYVYK	3560.21	3471.82	6218.5	9255.4
Histone H2B type 1-C/E/F/G/I	ESYSVYVYK	306.26	380.87	1472.71	1682.19
Histone H2B type 1-C/E/F/G/I	ESYSVYVYK	3524.13	4483.05	9306.06	11674.98
Histone H2B type 1-C/E/F/G/I	ESYSVYVYK	381.36	620.34	894.12	561.38
Histone H2B type 1-C/E/F/G/I	ESYSVYVYK	2094.24	2029.32	4970.16	5638.48
Histone H2B type 1-C/E/F/G/I	ESYSVYVYK	1164.41	1448.5	3386.67	4044.16
Histone H2B type 1-C/E/F/G/I	ESYSVYVYK	487.37	359.78	841.96	726.99
Histone H2B type 1-C/E/F/G/I	ESYSVYVYK	2780.75	1757.51	6113.09	5254.13
Histone H2B type 1-C/E/F/G/I	ESYSVYVYK	3277.69	3405.68	7494.11	7046.67
Histone H2B type 1-C/E/F/G/I	ESYSVYVYK	1574.9	1318.05	4026.75	5168.07
Histone H2B type 1-C/E/F/G/I	ESYSVYVYK	1007	829.58	2611.98	3024.25
Histone H2B type 1-C/E/F/G/I	ESYSVYVYK	48785.91	53034.17	184887	227413.7
Histone H2B type 1-C/E/F/G/I	ESYSVYVYK	4839.98	7139.82	14122.17	18183.23
Histone H2B type 1-C/E/F/G/I	ESYSVYVYK	1695.36	1331.49	2335.42	4239.5
Histone H2B type 1-C/E/F/G/I	ESYSVYVYK	2355.47	1753.02	3159.73	4062.06
Histone H2B type 1-C/E/F/G/I	ESYSVYVYK	1303.11	936.45	2889.18	2878.31
Histone H2B type 1-C/E/F/G/I	ESYSVYVYK	5778.92	4054.68	12073.42	13224.38
Histone H2B type 1-C/E/F/G/I	ESYSVYVYK	1366.56	1156.68	3411.37	2759.1
Histone H2B type 1-C/E/F/G/I	ESYSVYVYK	1171.57	1591.35	2623.64	3334.11
Histone H2B type 1-C/E/F/G/I	ESYSVYVYK	4705.24	3643.23	7638.75	11296.71
Histone H2B type 1-C/E/F/G/I	ESYSVYVYK	1849.69	1509.84	3106.62	3992.1
Histone H2B type 1-C/E/F/G/I	ESYSVYVYK	1007	829.58	2611.98	3024.25
Histone H2B type 1-C/E/F/G/I	ESYSVYVYK	5001.6	3831.47	8889.42	9568.49
Histone H2B type 1-C/E/F/G/I	ESYSVYVYK	41229.34	43012.36	144883.2	183573.4
Histone H2B type 1-C/E/F/G/I	ESYSVYVYK	8098.13	8964.59	17551.38	22549.53
Histone H2B type 1-C/E/F/G/I	ESYSVYVYK	56713.13	61118.64	230964.2	294623
Histone H2B type 1-C/E/F/G/I	ESYSVYVYK	1867.16	2017.82	3924.43	3966.87
Histone H2B type 1-C/E/F/G/I	ESYSVYVYK	2018.1	2318.57	3227.77	5624.49
Histone H2B type 1-C/E/F/G/I	ESYSVYVYK	1218.36	1437.11	1996.9	2262.72
Histone H2B type 1-C/E/F/G/I	ESYSVYVYK	5902.44	6957.63	13283.21	16096.88
Histone H2B type 1-C/E/F/G/I	ESYSVYVYK	487.13	383.27	957.38	1157.11
Histone H2B type 1-C/E/F/G/I	ESYSVYVYK	501.85	630.92	1365.24	1212.6
Histone H2B type 1-C/E/F/G/I	ESYSVYVYK	1012.29	945.65	2382.69	3580.91
Histone H2B type 1-C/E/F/G/I	ESYSVYVYK	3004.17	2131.04	5113.95	7018.25
Histone H2B type 1-C/E/F/G/I	ESYSVYVYK	898.49	1124.39	1990.05	2069.79
Histone H2B type 1-C/E/F/G/I	ESYSVYVYK	2780.75	1757.51	6113.09	5254.13
Histone H2B type 1-C/E/F/G/I	ESYSVYVYK	500.03	491.69	1823.41	1979.59

Histone H2B type 1-C/E/F/G/I	ESYSVYVYK	3311.48	3471.36	5716.58	6666.04
Histone H2B type 1-C/E/F/G/I	ESYSVYVYK	1448.13	1608.55	3259.34	3066.17
Histone H2B type 1-C/E/F/G/I	ESYSVYVYK	306.26	380.87	1472.71	1682.19
Histone H2B type 1-C/E/F/G/I	ESYSVYVYK	1985.16	2062.1	4812.21	5328.35
Histone H2B type 1-C/E/F/G/I	ESYSVYVYK	1076.35	574.21	2543.68	2194.12
Histone H2B type 1-C/E/F/G/I	ESYSVYVYK	1218.36	1437.11	1996.9	2262.72
Histone H2B type 1-C/E/F/G/I	ESYSVYVYK	2018.1	2318.57	3227.77	5624.49
Histone H2B type 1-C/E/F/G/I	ESYSVYVYK	1861.82	1694.81	2635.39	3914.55
Histone H2B type 1-C/E/F/G/I	ESYSVYVYK	1012.29	945.65	2382.69	3580.91
Histone H2B type 1-C/E/F/G/I	ESYSVYVYK	1012.29	945.65	2382.69	3580.91
Histone H2B type 1-C/E/F/G/I	ESYSVYVYK	3004.17	2131.04	5113.95	7018.25
Histone H2B type 1-C/E/F/G/I	ESYSVYVYK	1277.18	1264.06	2256.91	1761.06
Histone H2B type 1-C/E/F/G/I	ESYSVYVYK	1007	829.58	2611.98	3024.25
Histone H2B type 1-C/E/F/G/I	ESYSVYVYK	739.76	637.12	1888.77	1977.07
Histone H2B type 1-C/E/F/G/I	ESYSVYVYK	5408.01	4636.25	7323.39	7229.91
Histone H2B type 1-C/E/F/G/I	ESYSVYVYK	1076.35	574.21	2543.68	2194.12
Histone H2B type 1-C/E/F/G/I	ESYSVYVYK	5857.07	6547.1	12640.76	14807.32
Histone H2B type 1-C/E/F/G/I	ESYSVYVYK	153.94	110.7	342.81	350.38
Histone H2B type 1-C/E/F/G/I	ESYSVYVYK	2780.75	1757.51	6113.09	5254.13
Histone H2B type 1-C/E/F/G/I	ESYSVYVYK	3697.5	3524.72	6609.07	8509.17
Histone H2B type 1-C/E/F/G/I	ESYSVYVYK	530.06	78.47	558.49	532.59
Histone H2B type 1-C/E/F/G/I	ESYSVYVYK	3004.17	2131.04	5113.95	7018.25
Histone H2B type 1-C/E/F/G/I	ESYSVYVYKVLK	485.46	548.44	1515.95	1803.44
Histone H2B type 1-C/E/F/G/I	ESYSVYVYKVLK	334.2	172.88	435.9	534.6
Histone H2B type 1-C/E/F/G/I	GELAK	1457.89	1389.09	2058.59	2290.22
Histone H2B type 1-C/E/F/G/I	GELAK	1348.31	1470.66	1669.66	3162.87
Histone H2B type 1-C/E/F/G/I	GELAK	773.77	893.62	1469.23	1073.26
Histone H2B type 1-C/E/F/G/I	GELAK	1903.96	1679.84	2583.27	3006.86
Histone H2B type 1-C/E/F/G/I	GELAK	824.81	851.06	1366.22	1404.52
Histone H2B type 1-C/E/F/G/I	GELAK	1256.84	933	1952.88	2320.18
Histone H2B type 1-C/E/F/G/I	GELAK	840.61	991.82	1153.9	1309.03
Histone H2B type 1-C/E/F/G/I	GELAK	1529.98	1394.42	1583.48	1682.39
Histone H2B type 1-C/E/F/G/I	GELAK	1387.51	1108.72	1664.47	2564.02
Histone H2B type 1-C/E/F/G/I	GELAK	1744.6	1572.91	2978.2	2970.08
Histone H2B type 1-C/E/F/G/I	GELAK	596.94	1130.41	1467.6	1142.85
Histone H2B type 1-C/E/F/G/I	GELAK	1511.1	1450.81	1629.16	2542.72
Histone H2B type 1-C/E/F/G/I	HAVSEGTK	277.5	243.61	974.78	1249.78
Histone H2B type 1-C/E/F/G/I	KESYSVYVYK	3554.9	4315.54	11468.69	15257.32
Histone H2B type 1-C/E/F/G/I	KESYSVYVYK	1738.93	2133.46	6227.21	8229.1
Histone H2B type 1-C/E/F/G/I	KESYSVYVYK	358.57	318.32	811.28	1130.39
Histone H2B type 1-C/E/F/G/I	KESYSVYVYK	937.13	1137.3	2752.4	3071.97
Histone H2B type 1-C/E/F/G/I	KESYSVYVYK	959.89	696.93	2325.44	3125.34
Histone H2B type 1-C/E/F/G/I	KESYSVYVYK	7109.95	6701.05	16762.98	23363.76
Histone H2B type 1-C/E/F/G/I	KESYSVYVYK	1023.16	722.51	2051.75	2386.53
Histone H2B type 1-C/E/F/G/I	KESYSVYVYK	2804.55	3235.95	7996.56	11950.12
Histone H2B type 1-C/E/F/G/I	KESYSVYVYK	5963.9	6417.74	21946.35	25978.26
Histone H2B type 1-C/E/F/G/I	KESYSVYVYK	887.79	1224.71	2332.73	3201.64
Histone H2B type 1-C/E/F/G/I	KESYSVYVYK	648.17	429.85	1945.56	2991.1
Histone H2B type 1-C/E/F/G/I	LAHYNK	2471.12	2985.83	10846.92	12348.12
Histone H2B type 1-C/E/F/G/I	LAHYNK	617.8	345.73	1058.58	1359.2
Histone H2B type 1-C/E/F/G/I	LAHYNK	539.66	299.56	1075.88	1173.62
Histone H2B type 1-C/E/F/G/I	LAHYNK	1469.56	2150.93	5392.58	5677.07
Histone H2B type 1-C/E/F/G/I	LAHYNK	4115.62	2853.34	9352.38	12624.02
Histone H2B type 1-C/E/F/G/I	LAHYNK	128.09	110.61	441.69	491.54
Histone H2B type 1-C/E/F/G/I	LAHYNK	253.54	375.86	743.47	774.99
Histone H2B type 1-C/E/F/G/I	LAHYNK	366.53	595.67	838.74	1024.38
Histone H2B type 1-C/E/F/G/I	LAHYNK	252.83	553.31	638.49	712.57
Histone H2B type 1-C/E/F/G/I	LLLPGEL	174.61	88.91	68.23	99.09
Histone H2B type 1-C/E/F/G/I	LLLPGEL	77.27	25.39	56.51	130.88
Histone H2B type 1-C/E/F/G/I	LLLPGEL	245.2	30.46	147.64	146.41
Histone H2B type 1-C/E/F/G/I	LLLPGELA	59.67	62.31	103.08	148.53
Histone H2B type 1-C/E/F/G/I	LLLPGELAK	3118.23	3734.01	11091.76	15233.83
Histone H2B type 1-C/E/F/G/I	LLLPGELAK	1039.37	1142.45	1857.06	2403.43
Histone H2B type 1-C/E/F/G/I	LLLPGELAK	1618.24	2355.68	6421.5	6540.6

Histone H2B type 1-C/E/F/G/I	LLLPGELAK	794.73	631.66	2595.77	2258.86
Histone H2B type 1-C/E/F/G/I	LLLPGELAK	666.66	624.4	2413.44	2912.42
Histone H2B type 1-C/E/F/G/I	LLLPGELAK	2401.33	3391.82	8997.65	14025.22
Histone H2B type 1-C/E/F/G/I	LLLPGELAK	593.91	578.67	1219.16	1493.28
Histone H2B type 1-C/E/F/G/I	LLLPGELAK	2236.58	3818.04	9793.53	13528.25
Histone H2B type 1-C/E/F/G/I	LLLPGELAK	1343.91	1905.47	3793.6	5132.07
Histone H2B type 1-C/E/F/G/I	LLLPGELAK	537.5	512.93	1060.53	1094.38
Histone H2B type 1-C/E/F/G/I	LLLPGELAK	371.53	208.2	519.86	770.54
Histone H2B type 1-C/E/F/G/I	LLLPGELAK	406.7	669.84	958.67	1622.88
Histone H2B type 1-C/E/F/G/I	LLLPGELAK	2807.67	2394.98	9107.28	11461.73
Histone H2B type 1-C/E/F/G/I	LLLPGELAK	807.31	984.45	2810.32	3653.83
Histone H2B type 1-C/E/F/G/I	LLLPGELAK	8914.86	11465.29	36066.34	47495.6
Histone H2B type 1-C/E/F/G/I	LLLPGELAK	752.07	803.27	3411.88	4242.67
Histone H2B type 1-C/E/F/G/I	LLLPGELAK	2414.16	2443.86	7759.3	8630.15
Histone H2B type 1-C/E/F/G/I	LLLPGELAK	767.64	648.14	1454.45	2290.05
Histone H2B type 1-C/E/F/G/I	LLLPGELAK	1036.08	1254.15	3949.86	4804.5
Histone H2B type 1-C/E/F/G/I	LLLPGELAK	494.18	325.18	1251.89	1414.49
Histone H2B type 1-C/E/F/G/I	LLLPGELAK	484.51	642.31	971.99	1588.89
Histone H2B type 1-C/E/F/G/I	LLLPGELAK	2519.26	1846.93	8764.65	8841.66
Histone H2B type 1-C/E/F/G/I	LLLPGELAK	279.7	451.76	776.26	1161.18
Histone H2B type 1-C/E/F/G/I	LLLPGELAK	1865.12	2007.05	5369.04	7283.45
Histone H2B type 1-C/E/F/G/I	LLLPGELAK	1903.63	3627.87	11751.3	7073.72
Histone H2B type 1-C/E/F/G/I	LLLPGELAK	332.43	348.19	706.47	1127.23
Histone H2B type 1-C/E/F/G/I	LLLPGELAK	1131.41	1198.17	3869.64	4297.46
Histone H2B type 1-C/E/F/G/I	LLLPGELAK	921.28	1426.3	4631.29	2444.01
Histone H2B type 1-C/E/F/G/I	LLLPGELAK	2814.92	2849.21	11238.57	12761.31
Histone H2B type 1-C/E/F/G/I	LLLPGELAK	3666.33	3093.06	12013.12	15002.3
Histone H2B type 1-C/E/F/G/I	LLLPGELAK	177.33	352.86	1043.82	1480.29
Histone H2B type 1-C/E/F/G/I	LLLPGELAK	1701.65	1455.38	4109.02	4200.89
Histone H2B type 1-C/E/F/G/I	LLLPGELAK	2151.55	2112.26	6934.03	7809.12
Histone H2B type 1-C/E/F/G/I	LLLPGELAK	483.33	758.44	1560.89	2147.81
Histone H2B type 1-C/E/F/G/I	LLLPGELAK	3551.05	4376.92	13074.34	17459.77
Histone H2B type 1-C/E/F/G/I	LLLPGELAK	1121.8	1402.88	5214.17	5607.28
Histone H2B type 1-C/E/F/G/I	LLLPGELAK	30.4	82.05	138.21	190.53
Histone H2B type 1-C/E/F/G/I	LLLPGELAK	9182.82	9352.88	27613.06	37228.57
Histone H2B type 1-C/E/F/G/I	LLLPGELAK	6498.58	6357.97	21507.41	26017.28
Histone H2B type 1-C/E/F/G/I	LLLPGELAK	2275.72	2716.48	3078.4	4492.22
Histone H2B type 1-C/E/F/G/I	LLLPGELAK	1172.59	1491.49	4102.13	3343.25
Histone H2B type 1-C/E/F/G/I	LLLPGELAK	1994.95	1945.77	6328.86	6984.73
Histone H2B type 1-C/E/F/G/I	LLLPGELAK	145.55	88.02	219	266.7
Histone H2B type 1-C/E/F/G/I	LLLPGELAK	6223.82	5951.17	21786.56	25861.08
Histone H2B type 1-C/E/F/G/I	LLLPGELAK	179.33	154.97	612.66	777.82
Histone H2B type 1-C/E/F/G/I	LLLPGELAK	5933.46	6871.68	21091.49	25176.14
Histone H2B type 1-C/E/F/G/I	LLLPGELAK	359.25	251.06	306.26	394.04
Histone H2B type 1-C/E/F/G/I	LLLPGELAK	1574.17	1603.44	5550.22	6852.2
Histone H2B type 1-C/E/F/G/I	LLLPGELAK	2768.83	2083.76	7304.7	9668.31
Histone H2B type 1-C/E/F/G/I	LLLPGELAK	1016.43	961.54	3017.87	2748.96
Histone H2B type 1-C/E/F/G/I	LLLPGELAK	282.63	161.9	1168.04	995.8
Histone H2B type 1-C/E/F/G/I	LLLPGELAK	2971.21	3897.72	7574.76	8478
Histone H2B type 1-C/E/F/G/I	LLLPGELAK	5031.62	5333.15	17313.57	23369.74
Histone H2B type 1-C/E/F/G/I	LLLPGELAK	5442.24	6467.29	22576.94	27520.45
Histone H2B type 1-C/E/F/G/I	LLLPGELAK	1638.11	1456.21	3981.75	4054.66
Histone H2B type 1-C/E/F/G/I	LLLPGELAK	615.03	513.24	1612.08	1136.32
Histone H2B type 1-C/E/F/G/I	LLLPGELAK	890.91	703.71	1019.71	1340.11
Histone H2B type 1-C/E/F/G/I	LLLPGELAK	729.53	1008.84	3271.78	1099.75
Histone H2B type 1-C/E/F/G/I	LLLPGELAK	411.55	191.07	529.38	808.11
Histone H2B type 1-C/E/F/G/I	LLLPGELAK	1897.31	1698.78	5872.86	6434.08
Histone H2B type 1-C/E/F/G/I	LLLPGELAK	1192.42	595.61	1964.05	1989.15
Histone H2B type 1-C/E/F/G/I	LLLPGELAK	1783.99	3638.79	6915.31	7886.89
Histone H2B type 1-C/E/F/G/I	LLLPGELAK	480.34	309.21	815.79	1452.68
Histone H2B type 1-C/E/F/G/I	LLLPGELAK	332.63	328.77	389.72	634.68
Histone H2B type 1-C/E/F/G/I	LLLPGELAK	435.55	372.7	392.64	684.85
Histone H2B type 1-C/E/F/G/I	LLLPGELAK	21558.18	22970.01	84709.3	100086.6
Histone H2B type 1-C/E/F/G/I	LLLPGELAK	2433.83	2521	7275.45	6787.92

Histone H2B type 1-C/E/F/G/I	LLLPGELAK	2813.87	2633.72	6530.23	7513.52
Histone H2B type 1-C/E/F/G/I	LLLPGELAK	1660.52	1263.61	2576.05	2291.27
Histone H2B type 1-C/E/F/G/I	LLLPGELAK	828.49	1870.8	3354.73	662.9
Histone H2B type 1-C/E/F/G/I	LLLPGELAK	5611.71	6320.03	19559.82	29360.41
Histone H2B type 1-C/E/F/G/I	LLLPGELAK	360.09	168.98	534.2	663.89
Histone H2B type 1-C/E/F/G/I	LLLPGELAK	203.1	359.26	632.65	325.1
Histone H2B type 1-C/E/F/G/I	LLLPGELAK	3383.03	3317.13	8350	12691.89
Histone H2B type 1-C/E/F/G/I	LLLPGELAK	155.02	110.33	144.3	203.94
Histone H2B type 1-C/E/F/G/I	LLLPGELAK	1065.82	1507.85	3152.39	3756.56
Histone H2B type 1-C/E/F/G/I	LLLPGELAK	1131.2	898.98	2447.26	1928.78
Histone H2B type 1-C/E/F/G/I	LLLPGELAK	510.71	287.75	489.58	528.36
Histone H2B type 1-C/E/F/G/I	LLLPGELAK	469.44	641.17	1455.69	1718.37
Histone H2B type 1-C/E/F/G/I	LLLPGELAK	71637.41	77802.4	271380.3	335139
Histone H2B type 1-C/E/F/G/I	LLLPGELAK	1780.25	1455.69	4443.98	4454.83
Histone H2B type 1-C/E/F/G/I	LLLPGELAK	281.97	227.06	500.52	383.88
Histone H2B type 1-C/E/F/G/I	LLLPGELAK	2574.82	2953.29	8193.59	6726.86
Histone H2B type 1-C/E/F/G/I	LLLPGELAK	882.28	1024.07	1427.12	1576.26
Histone H2B type 1-C/E/F/G/I	LLLPGELAK	37046.08	39713.57	133930.9	177706.6
Histone H2B type 1-C/E/F/G/I	LLLPGELAK	588.57	255.22	536.63	384.05
Histone H2B type 1-C/E/F/G/I	LLLPGELAK	280.67	355.34	542.16	442.7
Histone H2B type 1-C/E/F/G/I	LLLPGELAK	52962.66	58678.16	211020.7	256683.5
Histone H2B type 1-C/E/F/G/I	LLLPGELAK	2959.02	3718.83	8224.17	11460.16
Histone H2B type 1-C/E/F/G/I	LLLPGELAK	295.42	174.48	179.74	447.7
Histone H2B type 1-C/E/F/G/I	LLLPGELAK	282.76	149.08	525.39	435.49
Histone H2B type 1-C/E/F/G/I	LLLPGELAK	255.09	329.34	645.54	170.93
Histone H2B type 1-C/E/F/G/I	LLLPGELAK	121.71	102.52	227.06	362.24
Histone H2B type 1-C/E/F/G/I	LLLPGELAK	411.56	189.89	587.55	663.02
Histone H2B type 1-C/E/F/G/I	LLLPGELAK	117258.5	120428.4	479013	579883.8
Histone H2B type 1-C/E/F/G/I	LLLPGELAK	466.04	126.8	423.51	248.12
Histone H2B type 1-C/E/F/G/I	LLLPGELAK	338.38	79.77	186.22	357.18
Histone H2B type 1-C/E/F/G/I	LLLPGELAK	3085.96	2773.44	7557.54	7742.94
Histone H2B type 1-C/E/F/G/I	LLLPGELAK	54292.88	60659.47	220208.2	270141.6
Histone H2B type 1-C/E/F/G/I	LLLPGELAK	526.54	425.81	1261.82	1652.57
Histone H2B type 1-C/E/F/G/I	LLLPGELAK	358.78	298.46	516.53	798.79
Histone H2B type 1-C/E/F/G/I	LLLPGELAK	811.75	971.29	2487.96	2266.3
Histone H2B type 1-C/E/F/G/I	LLLPGELAK	2689.15	1551.33	2286.24	2835.94
Histone H2B type 1-C/E/F/G/I	LLLPGELAK	1197.02	885.85	2376.66	2153
Histone H2B type 1-C/E/F/G/I	LLLPGELAK	116793.6	129230	493195.1	601644.1
Histone H2B type 1-C/E/F/G/I	LLLPGELAK	357.61	413.84	398.39	416.85
Histone H2B type 1-C/E/F/G/I	LLLPGELAK	1752.32	65.54	548.46	432.18
Histone H2B type 1-C/E/F/G/I	LLLPGELAK	256.09	231.23	389.12	261.97
Histone H2B type 1-C/E/F/G/I	LLLPGELAK	90979.78	95646.55	400063.9	479092.8
Histone H2B type 1-C/E/F/G/I	LLLPGELAK	280.88	334.36	302.72	344.64
Histone H2B type 1-C/E/F/G/I	LLLPGELAK	1089.71	530.93	1657.29	1765.8
Histone H2B type 1-C/E/F/G/I	LLLPGELAK	95.04	78.26	88.77	123.94
Histone H2B type 1-C/E/F/G/I	LLLPGELAK	359.56	221.4	495.88	152.01
Histone H2B type 1-C/E/F/G/I	LLLPGELAK	828.49	1870.8	3354.73	662.9
Histone H2B type 1-C/E/F/G/I	LLLPGELAK	864.21	362.28	613.43	896.84
Histone H2B type 1-C/E/F/G/I	LLLPGELAK	145.73	70.64	120.65	288.5
Histone H2B type 1-C/E/F/G/I	LLLPGELAK	1268.26	975.55	3369.84	4412.23
Histone H2B type 1-C/E/F/G/I	LLLPGELAK	581.09	355.43	713.65	674.5
Histone H2B type 1-C/E/F/G/I	LLLPGELAK	729.53	1008.84	3271.78	1099.75
Histone H2B type 1-C/E/F/G/I	LLLPGELAK	729.53	1008.84	3271.78	1099.75
Histone H2B type 1-C/E/F/G/I	LLLPGELAK	154.54	50.63	120.39	160.83
Histone H2B type 1-C/E/F/G/I	LLLPGELAK	141.21	91.13	80.69	111.67
Histone H2B type 1-C/E/F/G/I	LLLPGELAK	380.66	689.66	552.84	623.18
Histone H2B type 1-C/E/F/G/I	LLLPGELAK	585.43	884.46	1282.76	1236.83
Histone H2B type 1-C/E/F/G/I	LLLPGELAK	926.29	824.79	2240.42	2365.7
Histone H2B type 1-C/E/F/G/I	LLLPGELAK	119.42	116.92	161.32	162.56
Histone H2B type 1-C/E/F/G/I	LLPGELAK	60.02	27.75	109.43	11.39
Histone H2B type 1-C/E/F/G/I	NSFVNDIFER	596.57	102.43	406.28	786.68
Histone H2B type 1-C/E/F/G/I	PEPAKSAPAPK	1919.95	1802.57	2836.57	3816.66
Histone H2B type 1-C/E/F/G/I	PGELAK	557.17	590.73	1448.34	1954.02
Histone H2B type 1-C/E/F/G/I	PGELAK	489.62	137.57	336.64	467.89

Histone H2B type 1-C/E/F/G/I	QVHPDTGISSK	379.86	768.59	1491.64	1658.16
Histone H2B type 1-C/E/F/G/I	QVHPDTGISSK	140.61	150.6	332.41	362.73
Histone H2B type 1-C/E/F/G/I	QVHPDTGISSK	154.73	31.77	414.7	552.06
Histone H2B type 1-C/E/F/G/I	QVHPDTGISSK	2443.89	3761.5	10766.94	13051.1
Histone H2B type 1-C/E/F/G/I	QVHPDTGISSK	295.73	250.46	781.52	1019.24
Histone H2B type 1-C/E/F/G/I	QVHPDTGISSK	38.81	0	86.25	136.89
Histone H2B type 1-C/E/F/G/I	QVHPDTGISSK	511	472.12	1527.91	1174.12
Histone H2B type 1-C/E/F/G/I	QVHPDTGISSK	139.82	441.57	1527.01	342.36
Histone H2B type 1-C/E/F/G/I	QVHPDTGISSK	186.85	262.76	625.54	515.49
Histone H2B type 1-C/E/F/G/I	QVHPDTGISSK	584.76	417.97	1063.81	1181.41
Histone H2B type 1-C/E/F/G/I	QVHPDTGISSK	616.06	624.17	1706.2	2797.51
Histone H2B type 1-C/E/F/G/I	QVHPDTGISSK	383.67	285.48	854.94	1032.22
Histone H2B type 1-C/E/F/G/I	QVHPDTGISSK	178.49	237.83	718.91	747.61
Histone H2B type 1-C/E/F/G/I	QVHPDTGISSK	1096.22	1057.18	3579.36	4322.77
Histone H2B type 1-C/E/F/G/I	QVHPDTGISSK	465.8	362.77	2049.28	2417.69
Histone H2B type 1-C/E/F/G/I	QVHPDTGISSK	816.45	1039.08	2734.3	3987.61
Histone H2B type 1-C/E/F/G/I	QVHPDTGISSK	167.34	62.46	764.92	659.35
Histone H2B type 1-C/E/F/G/I	QVHPDTGISSK	165.2	274.32	452.19	614.85
Histone H2B type 1-C/E/F/G/I	QVHPDTGISSK	3475.91	3609.26	11005.28	14347.75
Histone H2B type 1-C/E/F/G/I	QVHPDTGISSK	102.56	80.32	119.03	147.92
Histone H2B type 1-C/E/F/G/I	QVHPDTGISSK	69.07	90.73	205.49	288.3
Histone H2B type 1-C/E/F/G/I	QVHPDTGISSK	69.07	90.73	205.49	288.3
Histone H2B type 1-C/E/F/G/I	QVHPDTGISSK	76.98	53.94	240.85	331.69
Histone H2B type 1-C/E/F/G/I	QVHPDTGISSK	1559.63	805.28	4555.08	3439.98
Histone H2B type 1-C/E/F/G/I	QVHPDTGISSK	25.1	73.67	93.44	162.6
Histone H2B type 1-C/E/F/G/I	QVHPDTGISSK	25.1	73.67	93.44	162.6
Histone H2B type 1-C/E/F/G/I	QVHPDTGISSK	3253.38	3572.93	11756.11	13585.31
Histone H2B type 1-C/E/F/G/I	QVHPDTGISSK	649.38	735.91	1588.29	2517.2
Histone H2B type 1-C/E/F/G/I	QVHPDTGISSK	141.56	162.83	255.98	343.08
Histone H2B type 1-C/E/F/G/I	QVHPDTGISSK	626.55	757.5	710.37	1069.79
Histone H2B type 1-C/E/F/G/I	QVHPDTGISSK	704.41	830.85	2062.74	2178.74
Histone H2B type 1-C/E/F/G/I	QVHPDTGISSK	1366.71	1355.07	4181.62	3928.41
Histone H2B type 1-C/E/F/G/I	QVHPDTGISSK	208.94	207.8	603.49	754.37
Histone H2B type 1-C/E/F/G/I	QVHPDTGISSK	823.83	1160.99	2846.69	3889.25
Histone H2B type 1-C/E/F/G/I	QVHPDTGISSK	728.14	933.21	1007.91	1012.69
Histone H2B type 1-C/E/F/G/I	QVHPDTGISSK	628.49	459.44	1192.58	1410.75
Histone H2B type 1-C/E/F/G/I	QVHPDTGISSK	200.6	393.96	733.99	842.76
Histone H2B type 1-C/E/F/G/I	QVHPDTGISSK	628.38	683.28	1822.09	2391.75
Histone H2B type 1-C/E/F/G/I	QVHPDTGISSK	724.46	765.43	1661.54	2052.16
Histone H2B type 1-C/E/F/G/I	QVHPDTGISSK	51.9	84.69	141.77	172.36
Histone H2B type 1-C/E/F/G/I	QVHPDTGISSK	1449.92	1644.33	4035.69	5830.76
Histone H2B type 1-C/E/F/G/I	QVHPDTGISSK	114.74	153.15	301.4	320.25
Histone H2B type 1-C/E/F/G/I	QVHPDTGISSK	372.47	434.36	924.64	1434.78
Histone H2B type 1-C/E/F/G/I	QVHPDTGISSK	128.54	66.01	93.27	175.52
Histone H2B type 1-C/E/F/G/I	QVHPDTGISSK	1494.85	1673.42	5144.98	6228.82
Histone H2B type 1-C/E/F/G/I	QVHPDTGISSK	542.31	463.43	1257.76	1812.53
Histone H2B type 1-C/E/F/G/I	QVHPDTGISSK	0	37.81	45.72	113.16
Histone H2B type 1-C/E/F/G/I	QVHPDTGISSK	945.16	1088.17	3930.02	4606.16
Histone H2B type 1-C/E/F/G/I	QVHPDTGISSK	229.83	378.7	1009.41	927.45
Histone H2B type 1-C/E/F/G/I	QVHPDTGISSK	502.69	442.05	1556.21	1970.14
Histone H2B type 1-C/E/F/G/I	QVHPDTGISSK	129.89	358.67	930.32	574.94
Histone H2B type 1-C/E/F/G/I	QVHPDTGISSK	1871.73	2843.05	8670.33	11042.9
Histone H2B type 1-C/E/F/G/I	QVHPDTGISSK	45.64	171.67	436.43	331.76
Histone H2B type 1-C/E/F/G/I	QVHPDTGISSK	208.94	207.8	603.49	754.37
Histone H2B type 1-C/E/F/G/I	QVHPDTGISSK	127.52	166.89	213.23	258.27
Histone H2B type 1-C/E/F/G/I	QVHPDTGISSK	3489.25	3461.14	9932.71	14329.38
Histone H2B type 1-C/E/F/G/I	QVHPDTGISSK	1311.8	1886.19	4333.22	6577.8
Histone H2B type 1-C/E/F/G/I	QVHPDTGISSK	50.21	146.14	246.45	61.03
Histone H2B type 1-C/E/F/G/I	QVHPDTGISSK	408.16	525.68	1542.45	1939.5
Histone H2B type 1-C/E/F/G/I	QVHPDTGISSK	704.41	830.85	2062.74	2178.74
Histone H2B type 1-C/E/F/G/I	QVHPDTGISSK	577.22	631.1	867.52	1190.3
Histone H2B type 1-C/E/F/G/I	QVHPDTGISSK	683.56	763.94	2948.66	2499.52
Histone H2B type 1-C/E/F/G/I	QVHPDTGISSK	381.81	575.92	802.6	875.73
Histone H2B type 1-C/E/F/G/I	QVHPDTGISSK	1726.62	2605.05	6454.36	8010.2

Histone H2B type 1-C/E/F/G/I	QVHPDTGISSK	660.21	411.13	1444.79	948.62
Histone H2B type 1-C/E/F/G/I	QVHPDTGISSK	9744.27	9839.75	42109.06	46383.64
Histone H2B type 1-C/E/F/G/I	QVHPDTGISSK	93.52	122.25	311.84	467.2
Histone H2B type 1-C/E/F/G/I	QVHPDTGISSK	407	639.99	679.14	846.6
Histone H2B type 1-C/E/F/G/I	QVHPDTGISSK	726.92	629	1593.06	1868.92
Histone H2B type 1-C/E/F/G/I	QVHPDTGISSK	176.6	105.94	175.82	340.65
Histone H2B type 1-C/E/F/G/I	QVHPDTGISSK	307.36	272.24	725.65	654.57
Histone H2B type 1-C/E/F/G/I	QVHPDTGISSK	134.52	326.5	483.2	441.9
Histone H2B type 1-C/E/F/G/I	QVHPDTGISSK	162.7	201.69	330.72	309.64
Histone H2B type 1-C/E/F/G/I	QVHPDTGISSK	2475.98	2718.65	9311.93	10968.72
Histone H2B type 1-C/E/F/G/I	QVHPDTGISSK	11194.18	11166.58	48393.93	56122.98
Histone H2B type 1-C/E/F/G/I	QVHPDTGISSK	1619.86	1238.21	2492.3	2774.98
Histone H2B type 1-C/E/F/G/I	QVHPDTGISSK	112.83	128.83	234.65	270.03
Histone H2B type 1-C/E/F/G/I	QVHPDTGISSK	366.25	304.36	622.73	540.72
Histone H2B type 1-C/E/F/G/I	QVHPDTGISSK	215.25	223.25	294.92	449.98
Histone H2B type 1-C/E/F/G/I	QVHPDTGISSK	372.47	434.36	924.64	1434.78
Histone H2B type 1-C/E/F/G/I	QVHPDTGISSK	628.38	683.28	1822.09	2391.75
Histone H2B type 1-C/E/F/G/I	QVHPDTGISSK	668.97	502.48	1255.36	1302.39
Histone H2B type 1-C/E/F/G/I	QVHPDTGISSK	668.97	502.48	1255.36	1302.39
Histone H2B type 1-C/E/F/G/I	QVHPDTGISSK	704.41	830.85	2062.74	2178.74
Histone H2B type 1-C/E/F/G/I	QVHPDTGISSK	184.1	215.86	508.84	779.88
Histone H2B type 1-C/E/F/G/I	QVHPDTGISSK	139.82	441.57	1527.01	342.36
Histone H2B type 1-C/E/F/G/I	QVHPDTGISSK	93.52	122.25	311.84	467.2
Histone H2B type 1-C/E/F/G/I	QVHPDTGISSK	1626.49	1327.99	3186.63	3806.67
Histone H2B type 1-C/E/F/G/I	QVHPDTGISSK	138.51	145.51	326.35	361.2
Histone H2B type 1-C/E/F/G/I	QVHPDTGISSK	138.51	145.51	326.35	361.2
Histone H2B type 1-C/E/F/G/I	QVHPDTGISSK	348	405.16	809.63	826.98
Histone H2B type 1-C/E/F/G/I	QVHPDTGISSK	1449.92	1644.33	4035.69	5830.76
Histone H2B type 1-C/E/F/G/I	QVHPDTGISSK	549.27	414.25	1590.63	1810.18
Histone H2B type 1-C/E/F/G/I	QVHPDTGISSK	76.62	89.54	396.22	339.24
Histone H2B type 1-C/E/F/G/I	QVHPDTGISSK	162.31	134.55	408.68	428.88
Histone H2B type 1-C/E/F/G/I	QVHPDTGISSK	465.25	417.32	1214.53	1214.44
Histone H2B type 1-C/E/F/G/I	QVHPDTGISSK	184.1	215.86	508.84	779.88
Histone H2B type 1-C/E/F/G/I	QVHPDTGISSK	76.62	89.54	396.22	339.24
Histone H2B type 1-C/E/F/G/I	QVHPDTGISSK	208.94	207.8	603.49	754.37
Histone H2B type 1-C/E/F/G/I	QVHPDTGISSK	3489.25	3461.14	9932.71	14329.38
Histone H2B type 1-C/E/F/G/I	QVHPDTGISSK	0	37.81	45.72	113.16
Histone H2B type 1-C/E/F/G/I	QVHPDTGISSK	1726.62	2605.05	6454.36	8010.2
Histone H2B type 1-C/E/F/G/I	QVHPDTGISSK	138.51	145.51	326.35	361.2
Histone H2B type 1-C/E/F/G/I	QVHPDTGISSK	944.31	639.97	2062.09	1727.19
Histone H2B type 1-C/E/F/G/I	QVHPDTGISSK	1449.92	1644.33	4035.69	5830.76
Histone H2B type 1-C/E/F/G/I	QVHPDTGISSK	317.13	476.3	754.95	890.57
Histone H2B type 1-C/E/F/G/I	QVHPDTGISSK	51.9	84.69	141.77	172.36
Histone H2B type 1-C/E/F/G/I	QVHPDTGISSK	15209.35	15752.49	59960.52	73533.41
Histone H2B type 1-C/E/F/G/I	QVHPDTGISSK	421.36	392.27	912.72	704.8
Histone H2B type 1-C/E/F/G/I	QVHPDTGISSK	279.61	460.02	633.63	890.77
Histone H2B type 1-C/E/F/G/I	QVHPDTGISSK	214.96	464.46	651.04	490.5
Histone H2B type 1-C/E/F/G/I	QVHPDTGISSK	314.44	210.69	552.98	420.37
Histone H2B type 1-C/E/F/G/I	QVHPDTGISSK	717.95	1195.61	1362.5	1629.39
Histone H2B type 1-C/E/F/G/I	QVHPDTGISSK	408.16	525.68	1542.45	1939.5
Histone H2B type 1-C/E/F/G/I	QVHPDTGISSK	93.52	122.25	311.84	467.2
Histone H2B type 1-C/E/F/G/I	QVHPDTGISSK	439.08	449.94	973.82	658.43
Histone H2B type 1-C/E/F/G/I	QVHPDTGISSK	549.27	414.25	1590.63	1810.18
Histone H2B type 1-C/E/F/G/I	QVHPDTGISSK	421.2	407.33	806.74	1016.64
Histone H2B type 1-C/E/F/G/I	QVHPDTGISSK	679.79	710.15	1314.88	1310.9
Histone H2B type 1-C/E/F/G/I	QVHPDTGISSK	372.47	434.36	924.64	1434.78
Histone H2B type 1-C/E/F/G/I	QVHPDTGISSK	628.38	683.28	1822.09	2391.75
Histone H2B type 1-C/E/F/G/I	QVHPDTGISSK	310.42	394.85	819.37	1169.3
Histone H2B type 1-C/E/F/G/I	QVHPDTGISSK	76.33	117.98	266.9	295.26
Histone H2B type 1-C/E/F/G/I	QVHPDTGISSK	76.33	117.98	266.9	295.26
Histone H2B type 1-C/E/F/G/I	QVHPDTGISSK	140.34	177.57	275.87	294.26
Histone H2B type 1-C/E/F/G/I	QVHPDTGISSK	51.9	84.69	141.77	172.36
Histone H2B type 1-C/E/F/G/I	QVHPDTGISSK	437.23	207.13	917.3	1118.55
Histone H2B type 1-C/E/F/G/I	QVHPDTGISSK	149.28	145.26	352.16	323.52

Histone H2B type 1-C/E/F/G/I	QVHPDTGISSK	387.86	403.29	1447.72	1916.54
Histone H2B type 1-C/E/F/G/I	QVHPDTGISSK	324.38	505.56	876.69	1241.13
Histone H2B type 1-C/E/F/G/I	QVHPDTGISSK	5744.36	7681.94	27925.91	30255.76
Histone H2B type 1-C/E/F/G/I	QVHPDTGISSK	16258.34	14881.07	65978.89	69315.01
Histone H2B type 1-C/E/F/G/I	RLLLPGELAK	330.49	539.6	1457.48	360.85
Histone H2B type 1-C/E/F/G/I	RLLLPGELAK	575.39	706.01	1689.55	625.7
Histone H2B type 1-C/E/F/G/I	RLLLPGELAK	947.02	1224.01	2735.03	1086.69
Histone H2B type 1-C/E/F/G/I	SFVNDIFER	636.63	719.77	465.32	944.88
Histone H2B type 1-C/E/F/G/I	SFVNDIFER	645.71	992.13	1001.56	1640.46
Histone H2B type 1-C/E/F/G/I	SFVNDIFER	153.17	186.35	433.11	613.19
Histone H2B type 1-C/E/F/G/I	SFVNDIFER	255.86	253.61	559.14	557.09
Histone H2B type 1-C/E/F/G/I	SFVNDIFER	3668.94	4218.2	9749.35	10874.69
Histone H2B type 1-C/E/F/G/I	SFVNDIFER	306.79	327.73	535.69	428.05
Histone H2B type 1-C/E/F/G/I	SFVNDIFER	612.59	541.46	757.42	718.08
Histone H2B type 1-C/E/F/G/I	STITSR	150.13	167.65	411.62	405.42
Histone H2B type 1-C/E/F/G/I	VHPDTGISSK	173.14	234.29	169.33	215.43
Histone H2B type 1-C/E/F/G/I	VLKQVHPDTGISSK	724.06	698.4	2159.61	3122.19
Histone H2B type 1-C/E/F/G/I	VLKQVHPDTGISSK	5107.65	6013.14	14567.1	18404.69
Histone H2B type 1-C/E/F/G/I	VLKQVHPDTGISSK	1913.83	2088.29	4310.21	7676.46
Sphingomyelin phosphodiesterase 2	AQAELQHVLGR	2004.27	2089	3252.15	2641.25
Sphingomyelin phosphodiesterase 2	AQAELQHVLGR	2944.88	2882.39	5557.33	5812.39
Sphingomyelin phosphodiesterase 2	AQAR	206.2	53.15	444.83	501
Sphingomyelin phosphodiesterase 2	LGLL	1296.99	1434.54	5346.49	5340.23
Sphingomyelin phosphodiesterase 2	LGLL	667.11	261.48	2492.45	2908.78
Sphingomyelin phosphodiesterase 2	LGLL	3314.76	3998.01	22099.16	20825.3
Sphingomyelin phosphodiesterase 2	LGLL	3659.85	3414.84	18140.3	21295.9
Sphingomyelin phosphodiesterase 2	LGLL	332.65	326.7	492.06	841.9
Sphingomyelin phosphodiesterase 2	LLAL	196.22	188.4	226.96	250.42
Sphingomyelin phosphodiesterase 2	LLAL	75.78	172.69	115.6	69.95
Sphingomyelin phosphodiesterase 2	VFSK	1080.67	1104.39	1567.12	2017.99
Prostate stem cell antigen	AVGLLTVISK	128.32	88.3	267.91	461.39
Prostate stem cell antigen	AVGLLTVISK	90.37	114.64	221.22	213.32
Prostate stem cell antigen	ILAL	196.22	188.4	226.96	250.42
Prostate stem cell antigen	ILAL	75.78	172.69	115.6	69.95
Prostate stem cell antigen	LGLL	1296.99	1434.54	5346.49	5340.23
Prostate stem cell antigen	LGLL	667.11	261.48	2492.45	2908.78
Prostate stem cell antigen	LGLL	3314.76	3998.01	22099.16	20825.3
Prostate stem cell antigen	LGLL	3659.85	3414.84	18140.3	21295.9
Prostate stem cell antigen	LGLL	332.65	326.7	492.06	841.9
Prostate stem cell antigen	LLAL	196.22	188.4	226.96	250.42
Prostate stem cell antigen	LLAL	75.78	172.69	115.6	69.95
Prostate stem cell antigen	TVISK	4782.88	6610.31	9727.15	9915.58
Tumor necrosis factor receptor superfamily member 12A	GSSWSADLDK	2271.1	2109.02	2985.78	2758.18
Tumor necrosis factor receptor superfamily member 12A	MARGSLR	2321.88	3049.72	3180.05	3235.44
Tumor necrosis factor receptor superfamily member 12A	GSSWSADLDK	1667.09	1784.67	2254.8	2090.29
Tumor necrosis factor receptor superfamily member 12A	LALLR	1010.67	892.3	1517.21	956.09
Tumor necrosis factor receptor superfamily member 12A	LALLR	562.37	502.42	1117.23	1193.58
Tumor necrosis factor receptor superfamily member 12A	LGLL	1296.99	1434.54	5346.49	5340.23
Tumor necrosis factor receptor superfamily member 12A	LGLL	667.11	261.48	2492.45	2908.78
Tumor necrosis factor receptor superfamily member 12A	LGLL	3314.76	3998.01	22099.16	20825.3
Tumor necrosis factor receptor superfamily member 12A	LGLL	3659.85	3414.84	18140.3	21295.9
Tumor necrosis factor receptor superfamily member 12A	LGLL	332.65	326.7	492.06	841.9
Tumor necrosis factor receptor superfamily member 12A	LLRL	912.1	950.18	1143.27	1280.65
Tumor necrosis factor receptor superfamily member 12A	LLRL	556.67	959.36	927.48	1147.92
Tumor necrosis factor receptor superfamily member 12A	LVWR	830.07	969.55	1123.99	997.82
Tumor necrosis factor receptor superfamily member 12A	SGFLVWR	229.67	287.62	249.59	498
Tumor necrosis factor receptor superfamily member 12A	SGFLVWR	256.11	229.14	492.3	334.79
Tumor necrosis factor receptor superfamily member 12A	VALI	1296.99	1434.54	5346.49	5340.23
Tumor necrosis factor receptor superfamily member 12A	VALI	3314.76	3998.01	22099.16	20825.3
Tumor necrosis factor receptor superfamily member 12A	VALI	3659.85	3414.84	18140.3	21295.9
E3 ubiquitin-protein ligase TRIM11	LQPPEVVPMELR	383.84	375.75	431.26	259.53
E3 ubiquitin-protein ligase TRIM11	EPLAAFCGDELR	398.33	220.18	414.47	287.32
E3 ubiquitin-protein ligase TRIM11	FDPGPCVLGQER	1569.13	1356.07	2646.03	3122.82
E3 ubiquitin-protein ligase TRIM11	ILVF	143.35	92.25	124.04	131.39

E3 ubiquitin-protein ligase TRIM11	LGLL	1296.99	1434.54	5346.49	5340.23
E3 ubiquitin-protein ligase TRIM11	LGLL	667.11	261.48	2492.45	2908.78
E3 ubiquitin-protein ligase TRIM11	LGLL	3314.76	3998.01	22099.16	20825.3
E3 ubiquitin-protein ligase TRIM11	LGLL	3659.85	3414.84	18140.3	21295.9
E3 ubiquitin-protein ligase TRIM11	LGLL	332.65	326.7	492.06	841.9
E3 ubiquitin-protein ligase TRIM11	LQDIK	538.29	435.34	640.67	971.58
E3 ubiquitin-protein ligase TRIM11	LQDIK	1460.7	1855.93	2358.64	2539.25
E3 ubiquitin-protein ligase TRIM11	LQDIK	1346.76	1517.15	1981.63	2159.87
E3 ubiquitin-protein ligase TRIM11	LSEDRR	493.79	363.06	655.72	665.62
E3 ubiquitin-protein ligase TRIM11	RLLAEEEQQLLQR	462.39	274.74	459.06	362.28
60S ribosomal protein L29	AQAAAPASVPAQAPK	2334.67	1680.29	6195.06	7280.77
60S ribosomal protein L29	LAYIAHPK	1091.7	1609.98	2154.12	2037.67
60S ribosomal protein L29	AEAIK	510.45	632.33	838.51	1051.07
60S ribosomal protein L29	AEAIK	735	1000.94	1249.24	1590.88
60S ribosomal protein L29	AEAIK	459.3	580.02	1060.27	1537.12
60S ribosomal protein L29	AEAIK	510.3	541.23	664.54	1100.17
60S ribosomal protein L29	AEAIK	230.24	231.67	462.05	513.79
60S ribosomal protein L29	AEAIK	691.04	450.48	608.72	1129.16
60S ribosomal protein L29	AQAAAPASVPAQAPK	140.07	203.78	363.53	394.3
60S ribosomal protein L29	AQAAAPASVPAQAPK	101.68	166.56	324.25	496.34
60S ribosomal protein L29	AQAAAPASVPAQAPK	453.21	329.94	2017.32	2868.21
60S ribosomal protein L29	AQAAAPASVPAQAPK	1621.38	1513.32	7548.06	8973.84
60S ribosomal protein L29	AQAAAPASVPAQAPK	1627.28	930.07	10367.83	12292.14
60S ribosomal protein L29	AQAAAPASVPAQAPK	1568.39	897.37	3637.37	4152.4
60S ribosomal protein L29	AQAAAPASVPAQAPK	980.84	1606.14	2910.22	2757.99
60S ribosomal protein L29	AQAAAPASVPAQAPK	345.36	347.45	748.76	759.1
60S ribosomal protein L29	AQAAAPASVPAQAPK	750.98	1124.4	1306.93	1306.04
60S ribosomal protein L29	AQAAAPASVPAQAPK	1153.05	1187.72	1435.06	1410.06
60S ribosomal protein L29	AQAAAPASVPAQAPK	657.96	1271.69	3723.19	4623.08
60S ribosomal protein L29	AQAAAPASVPAQAPK	1532.64	1557.34	2876.51	2545.81
60S ribosomal protein L29	AQAAAPASVPAQAPK	1973.91	2428.95	4684.92	4836.07
60S ribosomal protein L29	AQAAAPASVPAQAPK	513.28	353.13	1075.53	900.39
60S ribosomal protein L29	AQAAAPASVPAQAPK	634.62	705.59	1227.59	1089.49
60S ribosomal protein L29	AQAAAPASVPAQAPK	308.11	197.92	569.32	760.66
60S ribosomal protein L29	AQAAAPASVPAQAPK	1422.06	1418.12	1870.73	2336.71
60S ribosomal protein L29	AQAAAPASVPAQAPK	750.98	1124.4	1306.93	1306.04
60S ribosomal protein L29	AQAAAPASVPAQAPK	872.61	808.97	1358.11	1114.87
60S ribosomal protein L29	LAYIAHPK	2819.61	1854.19	9751.83	9282.65
60S ribosomal protein L29	LAYIAHPK	1004.74	947.02	4204.44	3493.62
60S ribosomal protein L29	LAYIAHPK	1707.08	1237.81	7671.41	10647.88
60S ribosomal protein L29	LAYIAHPK	1351.45	1054.18	3377.67	3661.6
60S ribosomal protein L29	LAYIAHPK	2719.23	2835.62	16516.98	21085.3
60S ribosomal protein L29	LAYIAHPK	1903.72	2554.39	9641.19	8533.61
60S ribosomal protein L29	LAYIAHPK	668.96	1249.03	5548.61	7579.1
60S ribosomal protein L29	LAYIAHPK	1251.21	851.16	3256.02	5664.15
60S ribosomal protein L29	LAYIAHPK	484.48	644.77	1488.64	1756.34
60S ribosomal protein L29	LAYIAHPK	1097.13	1180.38	5779.56	6660.22
60S ribosomal protein L29	LAYIAHPK	943.04	766.28	5499.29	5092.58
60S ribosomal protein L29	LAYIAHPK	1271.06	2400.96	2953.93	3174.46
60S ribosomal protein L29	LAYIAHPK	664.72	496.93	3460.61	4664.11
60S ribosomal protein L29	LAYIAHPK	577.41	506.36	2090.31	2065.44
60S ribosomal protein L29	LAYIAHPK	945.67	825.18	4384.32	4870.69
60S ribosomal protein L29	LAYIAHPK	2296.14	2508.37	5372.94	5504.61
60S ribosomal protein L29	LAYIAHPK	585.81	1059.02	1050.31	1207.89
60S ribosomal protein L29	LAYIAHPK	860.33	639.39	2930.23	4239.33
60S ribosomal protein L29	LAYIAHPK	711.75	744.74	2252.98	3594.46
60S ribosomal protein L29	LAYIAHPK	1269.33	443.59	1542.61	2421.21
60S ribosomal protein L29	LAYIAHPK	703.31	620.73	2407.84	2572.93
60S ribosomal protein L29	LAYIAHPK	658.97	533.95	2447.84	2961.15
60S ribosomal protein L29	LAYIAHPK	306.9	317.33	1422.07	1819.36
60S ribosomal protein L29	LAYIAHPK	2984.78	2238.1	5580.58	5606.15
60S ribosomal protein L29	YESLK	1538.24	1535.58	5953.25	5531.07
60S ribosomal protein L29	YESLK	969.39	1673.58	4669.1	6246.91
60S ribosomal protein L29	YESLK	4095.43	4529.43	17577.63	21368.12

60S ribosomal protein L29	YESLK	3082.65	4164.64	11409.07	13751.92
60S ribosomal protein L29	YESLK	1453.92	1568.73	7707.87	7208.38
60S ribosomal protein L29	YESLK	1297.12	1527.93	6311.22	6729.36
60S ribosomal protein L29	YESLK	1182.42	1051.88	4642.53	3828.26
60S ribosomal protein L29	YESLK	2927.21	2712.91	7250.63	7875.29
60S ribosomal protein L29	YESLK	1050.57	567.91	1567.04	2012.29
Type 1 phosphatidylinositol 4,5-bisphosphate 4-phosphatase	VCQSLINVEGK	1681.33	1654.32	2853.37	2563.92
Type 1 phosphatidylinositol 4,5-bisphosphate 4-phosphatase	LAVL	667.11	261.48	2492.45	2908.78
Type 1 phosphatidylinositol 4,5-bisphosphate 4-phosphatase	LGLL	1296.99	1434.54	5346.49	5340.23
Type 1 phosphatidylinositol 4,5-bisphosphate 4-phosphatase	LGLL	667.11	261.48	2492.45	2908.78
Type 1 phosphatidylinositol 4,5-bisphosphate 4-phosphatase	LGLL	3314.76	3998.01	22099.16	20825.3
Type 1 phosphatidylinositol 4,5-bisphosphate 4-phosphatase	LGLL	3659.85	3414.84	18140.3	21295.9
Type 1 phosphatidylinositol 4,5-bisphosphate 4-phosphatase	LGLL	332.65	326.7	492.06	841.9
Type 1 phosphatidylinositol 4,5-bisphosphate 4-phosphatase	LICK	1603.37	2122.18	1643.54	2818.13
Type 1 phosphatidylinositol 4,5-bisphosphate 4-phosphatase	VCQSLINVEGK	2048.69	1955.4	4166.09	3094.09
Type 1 phosphatidylinositol 4,5-bisphosphate 4-phosphatase	VEGK	1478.35	2133.56	2017.74	3214.35
Type 1 phosphatidylinositol 4,5-bisphosphate 4-phosphatase	VLCL	611.67	738.6	636.02	731.71
Type 1 phosphatidylinositol 4,5-bisphosphate 4-phosphatase	VLCL	526.88	1349.68	824.39	1044.25
Type 1 phosphatidylinositol 4,5-bisphosphate 4-phosphatase	VLCL	280.74	348.72	178.21	170.57
Choline kinase alpha	LEQFIPSR	358.98	278.5	226.9	404.76
Choline kinase alpha	AQAR	206.2	53.15	444.83	501
Choline kinase alpha	IFPQGR	333.91	202.2	262.98	226.54
Choline kinase alpha	LGLL	1296.99	1434.54	5346.49	5340.23
Choline kinase alpha	LGLL	667.11	261.48	2492.45	2908.78
Choline kinase alpha	LGLL	3314.76	3998.01	22099.16	20825.3
Choline kinase alpha	LGLL	3659.85	3414.84	18140.3	21295.9
Choline kinase alpha	LGLL	332.65	326.7	492.06	841.9
Choline kinase alpha	LLEV	538.71	180.42	590.2	407.12
Choline kinase alpha	LLEV	581.57	945.93	1022.29	951.69
Choline kinase alpha	LLRL	912.1	950.18	1143.27	1280.65
Choline kinase alpha	LLRL	556.67	959.36	927.48	1147.92
Choline kinase alpha	LRIK	3814.3	3262.06	4759.66	4438.43
Choline kinase alpha	MPFNK	3074.2	4254.47	3964.41	4320.72
Putative histone H2B type 2-C	ESYSIYVYK	7359.69	8622.83	20940.72	29384.54
Putative histone H2B type 2-C	KESYSIYVYK	789.86	686.94	2698.18	2518.95
Putative histone H2B type 2-C	AHNTK	306.98	309.66	152.47	248.5
Putative histone H2B type 2-C	VHPDTGIWCK	128.82	39.12	145.83	197.73
Putative histone H2B type 2-C	FAPAPK	98.6	471.68	560.73	807
Putative histone H2B type 2-C	SIYVYK	777.71	717.09	1198.1	1706.9
Putative histone H2B type 2-C	AMGIMNSF	195.49	47.46	127.29	113.32
Putative histone H2B type 2-C	AMGIMNSF	214.49	85.76	184.4	273.87
Putative histone H2B type 2-C	AMGIMNSF	188.54	96.31	289.39	332.57
Putative histone H2B type 2-C	AMGIMNSF	150.04	69.84	144.38	251.1
Putative histone H2B type 2-C	AVTK	148.31	134.21	477.05	549.01
Putative histone H2B type 2-C	DIFER	1593.21	2487.58	2488.22	3345.97
Putative histone H2B type 2-C	ESYSIY	518.32	706.43	1186.45	1567.91
Putative histone H2B type 2-C	ESYSIYVY	385.11	249.72	278.09	474.99
Putative histone H2B type 2-C	ESYSIYVYK	2407.35	2584.54	4708.27	7602
Putative histone H2B type 2-C	ESYSIYVYK	1492.24	1506.08	2953.59	4650.42
Putative histone H2B type 2-C	ESYSIYVYK	2492.72	2341.78	6297.51	8283.88
Putative histone H2B type 2-C	ESYSIYVYK	2447.35	2994.17	7542.64	7935.67
Putative histone H2B type 2-C	ESYSIYVYK	2877.15	1595.05	5232.34	5730.78
Putative histone H2B type 2-C	ESYSIYVYK	2351.57	2776.72	6203.39	7197.23
Putative histone H2B type 2-C	ESYSIYVYK	1840.84	1746.13	4535.6	4535.71
Putative histone H2B type 2-C	ESYSIYVYK	2395.5	2159.53	4711.91	6033.31
Putative histone H2B type 2-C	ESYSIYVYK	2745.17	2399.89	5423.25	6049.48
Putative histone H2B type 2-C	ESYSIYVYK	1826.93	1524.67	4690.38	5744.3
Putative histone H2B type 2-C	ESYSIYVYK	332.09	381.88	955.68	946.51
Putative histone H2B type 2-C	ESYSIYVYK	1685.23	1375.38	3924.65	4511.28
Putative histone H2B type 2-C	ESYSIYVYK	10924.99	10528.04	24797.36	28806.99
Putative histone H2B type 2-C	ESYSIYVYK	1713.97	1195.27	1080.05	1900.41
Putative histone H2B type 2-C	ESYSIYVYK	3430.56	4472.62	10590.55	5635.34

Putative histone H2B type 2-C	ESYSIYVYK	1393.64	1247.78	1730.82	2776.3
Putative histone H2B type 2-C	ESYSIYVYK	28382.45	27044.79	107648.8	136501
Putative histone H2B type 2-C	ESYSIYVYK	8105.2	10819.34	27051.29	38427.75
Putative histone H2B type 2-C	ESYSIYVYK	30458.38	31521.15	113710.2	130986.3
Putative histone H2B type 2-C	ESYSIYVYK	24313.56	26382.4	85487.27	101571.4
Putative histone H2B type 2-C	ESYSIYVYK	927.29	939.27	2105.59	1582.47
Putative histone H2B type 2-C	ESYSIYVYK	512.21	458.73	855.9	1407.22
Putative histone H2B type 2-C	ESYSIYVYK	2742.94	2088.44	4987.17	6139.46
Putative histone H2B type 2-C	ESYSIYVYK	3980.45	3442.25	6460.54	8976.7
Putative histone H2B type 2-C	ESYSIYVYK	3217.36	2450.42	4225.16	4439.98
Putative histone H2B type 2-C	ESYSIYVYK	1665.3	1322.53	2356.93	2561.51
Putative histone H2B type 2-C	ESYSIYVYK	1359.01	1477.84	1647.73	2062.43
Putative histone H2B type 2-C	ESYSIYVYK	909.45	1212.11	1866.02	3003.51
Putative histone H2B type 2-C	ESYSIYVYK	512.21	458.73	855.9	1407.22
Putative histone H2B type 2-C	ESYSIYVYK	512.21	458.73	855.9	1407.22
Putative histone H2B type 2-C	ESYSIYVYK	3275.47	3624.15	7971.1	7434.94
Putative histone H2B type 2-C	ESYSIYVYK	3081.45	3537.7	4022.62	5567.5
Putative histone H2B type 2-C	ESYSIYVYK	2455.09	2016.49	4548.33	5916.35
Putative histone H2B type 2-C	ESYSIYVYK	1776.53	1929.09	2901.41	4401.92
Putative histone H2B type 2-C	ESYSIYVYK	3336.2	4326.61	5127.11	5899.32
Putative histone H2B type 2-C	ESYSIYVYK	1570.78	1512.88	3819.04	3607.31
Putative histone H2B type 2-C	IAGEASR	165.44	144.04	353.21	503.41
Putative histone H2B type 2-C	IAGEASRL	846.05	666.97	1033.18	1010.71
Putative histone H2B type 2-C	KESYSIYVYK	2461.09	2488.12	8802.06	10451.9
Putative histone H2B type 2-C	KESYSIYVYK	9107.13	8639.05	24797.96	30946.26
Putative histone H2B type 2-C	KESYSIYVYK	1132.86	735.3	1336.04	1442.95
Putative histone H2B type 2-C	KESYSIYVYK	6623.38	9028.73	24730.19	34352.63
Putative histone H2B type 2-C	KESYSIYVYK	435.58	370.09	521.59	1042.49
Putative histone H2B type 2-C	LAHYNK	471.05	695.36	1375.76	2331.67
Putative histone H2B type 2-C	LAHYNK	2471.12	2985.83	10846.92	12348.12
Putative histone H2B type 2-C	LAHYNK	617.8	345.73	1058.58	1359.2
Putative histone H2B type 2-C	LAHYNK	539.66	299.56	1075.88	1173.62
Putative histone H2B type 2-C	LAHYNK	1469.56	2150.93	5392.58	5677.07
Putative histone H2B type 2-C	LAHYNK	4115.62	2853.34	9352.38	12624.02
Putative histone H2B type 2-C	LAHYNK	128.09	110.61	441.69	491.54
Putative histone H2B type 2-C	LAHYNK	253.54	375.86	743.47	774.99
Putative histone H2B type 2-C	LAHYNK	366.53	595.67	838.74	1024.38
Putative histone H2B type 2-C	LAHYNK	252.83	553.31	638.49	712.57
Putative histone H2B type 2-C	LNDIFER	819.97	1116.49	2189.56	2278.42
Putative histone H2B type 2-C	LNDIFER	2050.07	2563.82	5819.06	4224.69
Putative histone H2B type 2-C	LNDIFER	4086.88	5905.96	6854.98	9693.13
Putative histone H2B type 2-C	SIYVYK	550.75	587.45	995.94	1652.17
Putative histone H2B type 2-C	STITSR	420.06	413.54	1088.58	1343.96
Putative histone H2B type 2-C	STITSR	150.13	167.65	411.62	405.42
Transmembrane protein 65	DFIYSLHSTER	1477.14	1401.75	1946.74	2017.84
Transmembrane protein 65	KEPMEALNTAQGAR	504.11	620.85	897.84	1031.93
Transmembrane protein 65	QVDMWQTR	1721.91	1794.1	3609.03	3413.87
Transmembrane protein 65	IGII	1296.99	1434.54	5346.49	5340.23
Transmembrane protein 65	IGII	667.11	261.48	2492.45	2908.78
Transmembrane protein 65	IGII	3314.76	3998.01	22099.16	20825.3
Transmembrane protein 65	IGII	3659.85	3414.84	18140.3	21295.9
Transmembrane protein 65	IGII	332.65	326.7	492.06	841.9
Transmembrane protein 65	KEPMEALNTAQGAR	539.28	443.73	1605.18	2047.13
Transmembrane protein 65	KEPMEALNTAQGAR	563.81	679.61	963.86	1453.82
Transmembrane protein 65	KEPMEALNTAQGAR	408.73	469.07	829.82	1192.68
Transmembrane protein 65	LLAL	196.22	188.4	226.96	250.42
Transmembrane protein 65	LLAL	75.78	172.69	115.6	69.95
Transmembrane protein 65	LPLLR	2878.52	2629.2	4548.76	4509.69
Transmembrane protein 65	LPLLR	2910.98	2507.35	3967.9	4971.32
Transmembrane protein 65	LPLLR	2091.52	3149.96	3906.01	4705.2
Transmembrane protein 65	LPLLR	3488.74	3936.48	5409.66	6450.34
Mothers against decapentaplegic homolog 5	VESPVLPPVLVPR	191.22	256.95	577.7	439.96
Mothers against decapentaplegic homolog 5	DVQPVAYEEPK	728.76	978.85	822.35	1086.91
Mothers against decapentaplegic homolog 5	ALSSPGQPSK	627.44	775.97	1016.92	1694.25

Mothers against decapentaplegic homolog 5	EVCINPYHYK	1828.29	2240.81	2238.26	2732.97
Mothers against decapentaplegic homolog 5	ALSSPGQPSK	449.79	349.32	592.7	758.18
Mothers against decapentaplegic homolog 5	ALSSPGQPSK	460.03	401.12	652.43	519.67
Mothers against decapentaplegic homolog 5	AVDALVK	1221.87	1090.2	1910.8	2284.54
Mothers against decapentaplegic homolog 5	AVDALVK	1585.64	1321.28	3987.36	3270.08
Mothers against decapentaplegic homolog 5	DALVK	728.04	730.32	1416.93	1299.98
Mothers against decapentaplegic homolog 5	DALVK	281.16	307.2	368.8	771.11
Mothers against decapentaplegic homolog 5	DALVK	330.72	516.99	979.64	830.05
Mothers against decapentaplegic homolog 5	DALVK	541.46	973.28	1092.56	951.76
Mothers against decapentaplegic homolog 5	FCLGLLSNVNR	255.79	260.79	204.22	347.81
Mothers against decapentaplegic homolog 5	FCLGLLSNVNR	232.28	30.42	196.97	220.67
Mothers against decapentaplegic homolog 5	GLPHVIYCR	3024.81	3176.33	4659.81	6743.55
Mothers against decapentaplegic homolog 5	LGLL	1296.99	1434.54	5346.49	5340.23
Mothers against decapentaplegic homolog 5	LGLL	667.11	261.48	2492.45	2908.78
Mothers against decapentaplegic homolog 5	LGLL	3314.76	3998.01	22099.16	20825.3
Mothers against decapentaplegic homolog 5	LGLL	3659.85	3414.84	18140.3	21295.9
Mothers against decapentaplegic homolog 5	LGLL	332.65	326.7	492.06	841.9
Mothers against decapentaplegic homolog 5	LLGWK	1541.18	2095.95	2849.91	3434.84
Mothers against decapentaplegic homolog 5	LLGWK	2032.78	1824.89	2595.5	3920.65
Mothers against decapentaplegic homolog 5	MSFVK	633.47	606.02	838.1	1358.43
Mothers against decapentaplegic homolog 5	VESPVLPPVLVPR	257.37	104.51	270.03	204.42
Interferon-related developmental regulator 2	LLPDFLLER	214.48	193.2	295.09	365.08
Interferon-related developmental regulator 2	QGALESLR	1333.42	2303.09	1609.46	1920.18
Interferon-related developmental regulator 2	LPQLLSSESVNLR	89.24	119.79	130.25	121.38
Interferon-related developmental regulator 2	AVLHSVEGGECEEEIVR	384.49	311.29	1064.11	950.5
Interferon-related developmental regulator 2	EIVR	334.17	388.86	709.55	798.59
Interferon-related developmental regulator 2	EIVR	153.27	176.51	174.96	329.95
Interferon-related developmental regulator 2	EIVR	180.45	257.22	444.12	348.36
Interferon-related developmental regulator 2	FLLER	1360.38	810.33	1843.89	1805.08
Interferon-related developmental regulator 2	FLLER	1038.48	1229.85	2004.81	1668.97
Interferon-related developmental regulator 2	FLLER	4006.63	3302.59	4549.6	5389.65
Interferon-related developmental regulator 2	LFELAR	102.71	64.79	248.31	166.5
Interferon-related developmental regulator 2	LGLL	1296.99	1434.54	5346.49	5340.23
Interferon-related developmental regulator 2	LGLL	667.11	261.48	2492.45	2908.78
Interferon-related developmental regulator 2	LGLL	3314.76	3998.01	22099.16	20825.3
Interferon-related developmental regulator 2	LGLL	3659.85	3414.84	18140.3	21295.9
Interferon-related developmental regulator 2	LGLL	332.65	326.7	492.06	841.9
Interferon-related developmental regulator 2	LPQLLSSESVNLR	599.46	30.08	144.79	211.13
Interferon-related developmental regulator 2	QGALESLR	702.07	849.83	919.42	1099.1
Interferon-related developmental regulator 2	QGALESLR	2605.93	2114.26	1712.46	1675.56
60S ribosomal protein L31	NLQTVNVDEN	357.19	454.97	439.69	667.22
60S ribosomal protein L31	SAINEVVTR	821.83	826.53	781.92	1919.87
60S ribosomal protein L31	IHGVGFK	4115.55	4243.2	11343.92	13274.84
60S ribosomal protein L31	EYTINIHK	3562.01	4357.69	11432.17	12187.15
60S ribosomal protein L31	EYTINIHKR	1159.17	1433.47	4309.04	6492.08
60S ribosomal protein L31	FAMKEMGTPDVR	1015.13	1303.17	1601.8	2555.19
60S ribosomal protein L31	EYTINIH	1473.86	1194.66	2787.92	2651.19
60S ribosomal protein L31	AVWAK	2053.7	2524.3	8558.8	10216.96
60S ribosomal protein L31	AVWAK	2421.55	3095.95	8878.76	8634.07
60S ribosomal protein L31	AVWAK	693.84	1130.47	2013.71	3172.17
60S ribosomal protein L31	AVWAK	6860.86	8863.8	19731.35	24183.16
60S ribosomal protein L31	AVWAK	7516.36	8250.38	25067.54	29906.85
60S ribosomal protein L31	AVWAK	1996.27	2879.13	3769.59	4752.48
60S ribosomal protein L31	AVWAK	3263.9	2852.91	6271.94	7391.23
60S ribosomal protein L31	AVWAK	1803.44	2780.97	4172.58	4117.48
60S ribosomal protein L31	AVWAK	674.75	889.53	2566.28	2206.24
60S ribosomal protein L31	AVWAK	11592.78	13912.97	25010.45	34096.6
60S ribosomal protein L31	AVWAK	1224.3	1275.69	2194.35	2602.54
60S ribosomal protein L31	AVWAK	1130.76	1368.28	2318.7	2319.53
60S ribosomal protein L31	AVWAK	12429.93	11949.28	37327.15	43216.64
60S ribosomal protein L31	AVWAK	7576.4	9127.25	23917.18	30152.94
60S ribosomal protein L31	EYTINIHK	5198.11	4416.31	16685.11	20881.51
60S ribosomal protein L31	EYTINIHK	894.55	982.31	2437.25	2526.95
60S ribosomal protein L31	EYTINIHK	4104.43	4810.42	10718.61	11776.48

60S ribosomal protein L31	EYTINIHK	2044.79	2447.03	5232.91	5239.33
60S ribosomal protein L31	EYTINIHK	18286.22	18360.55	75200.23	92285.68
60S ribosomal protein L31	EYTINIHK	1644.06	1719.09	4147.17	5062.23
60S ribosomal protein L31	EYTINIHK	1148.34	1546.65	4335.01	4793.68
60S ribosomal protein L31	EYTINIHK	1666.3	1011.05	4676.16	6170.3
60S ribosomal protein L31	EYTINIHK	2506.66	2560.27	7938.38	9454.02
60S ribosomal protein L31	EYTINIHK	882.85	1180.32	2791.07	2735.79
60S ribosomal protein L31	EYTINIHK	1120.51	1424.04	2150.98	3628.63
60S ribosomal protein L31	EYTINIHK	6500.92	6659.46	23127.24	28455.17
60S ribosomal protein L31	EYTINIHK	4163	3810.43	9759.24	10881.79
60S ribosomal protein L31	EYTINIHK	877.12	788.84	2177.73	2673.1
60S ribosomal protein L31	EYTINIHKR	1022.43	901.18	2311.44	3685.78
60S ribosomal protein L31	FAMK	3007.55	3711.17	9382.64	10454.26
60S ribosomal protein L31	FAMK	1285.38	1836.7	3420.35	5620.44
60S ribosomal protein L31	FAMK	3003.9	3327.02	8186.22	10407.54
60S ribosomal protein L31	FAMK	2988.79	3969.03	5738.11	9092.73
60S ribosomal protein L31	FAMK	1494.31	1833.07	3100.39	4688.25
60S ribosomal protein L31	FAMK	3849.62	4453.29	8255.63	11007.05
60S ribosomal protein L31	FAMK	1137.72	1425.27	2824.93	3427.63
60S ribosomal protein L31	FAMK	4737.05	3585.63	8722.35	13012.68
60S ribosomal protein L31	IHGVGFK	968.97	544.13	2137.9	1656.79
60S ribosomal protein L31	IHGVGFK	1529.87	1617.59	5331.55	6177.91
60S ribosomal protein L31	IHGVGFK	1493.85	1878.63	4784.64	5641.07
60S ribosomal protein L31	IHGVGFK	1898.81	2189.13	5866.26	6687.39
60S ribosomal protein L31	IHGVGFK	1063.63	1724.36	2662.87	3545.57
60S ribosomal protein L31	IHGVGFK	3497.63	4334.75	7962.56	10961.42
60S ribosomal protein L31	NLQTVNVDEN	1242.2	1528.4	3829.98	3130.88
60S ribosomal protein L31	NLQTVNVDEN	639.11	474.62	934.64	1317.24
60S ribosomal protein L31	NLQTVNVDEN	329.69	618.49	1281.14	1017.07
60S ribosomal protein L31	SAINEVVTR	6716.56	6311.35	26152.61	28579.54
60S ribosomal protein L31	SAINEVVTR	2215.39	1974.97	5778.45	6287.37
60S ribosomal protein L31	SAINEVVTR	2306.3	2781.12	6045.39	6873.25
60S ribosomal protein L31	SAINEVVTR	9779.9	8340.9	32347.39	41619.07
60S ribosomal protein L31	SAINEVVTR	1883.61	2095.13	4880.27	6536.26
60S ribosomal protein L31	SAINEVVTR	1090.26	1433.15	1917.49	2598.53
60S ribosomal protein L31	SAINEVVTR	2782.22	2250.57	6450.92	8420.08
60S ribosomal protein L31	SAINEVVTR	1819.59	1824.44	6164.9	5716.81
60S ribosomal protein L31	SAINEVVTR	2407.49	2463.87	4240.74	4320
60S ribosomal protein L31	SAINEVVTR	1678.21	2175.2	3161.46	3606.64
60S ribosomal protein L31	SAINEVVTR	6950.61	7230.98	18419.75	20828.69
60S ribosomal protein L31	SAINEVVTR	1305.54	909.3	2946.87	3013.04
60S ribosomal protein L31	SAINEVVTR	2842.01	2619.23	5122.62	4162.81
60S ribosomal protein L31	SAINEVVTR	428.9	710.6	1095.42	920.33
60S ribosomal protein L31	SAINEVVTR	2264.72	1570.12	2167.59	1786.85
60S ribosomal protein L31	SAINEVVTR	4076.5	4484.17	8130.15	7974.31
60S ribosomal protein L31	SAINEVVTR	482.56	515.22	1042.44	1054.43
60S ribosomal protein L31	SAINEVVTR	1415.95	1490.57	2351.65	2401.79
60S ribosomal protein L31	SAINEVVTR	4661.06	5560.58	11835.64	12284.95
60S ribosomal protein L31	SAINEVVTR	1702.47	1480.49	3130	3364.4
60S ribosomal protein L31	SAINEVVTR	1958.81	1899.57	3480.72	2587.66
Succinate dehydrogenase [ubiquinone] cytochrome b small subunit, mitochondrial	AVAMLWK	663.66	708.83	1014.16	1068.66
Succinate dehydrogenase [ubiquinone] cytochrome b small subunit, mitochondrial	ALLLR	2985.64	3641.8	8639.29	10643.07
Succinate dehydrogenase [ubiquinone] cytochrome b small subunit, mitochondrial	ALLLR	807.23	1311.26	2069.48	2004.08
Succinate dehydrogenase [ubiquinone] cytochrome b small subunit, mitochondrial	AVAMLWK	2142.79	1488.38	1764.28	2062.66
Succinate dehydrogenase [ubiquinone] cytochrome b small subunit, mitochondrial	AVAMLWK	537.21	541.71	542.48	764.85
Succinate dehydrogenase [ubiquinone] cytochrome b small subunit, mitochondrial	AVAMLWK	652.4	438.14	775.34	694.66
Succinate dehydrogenase [ubiquinone] cytochrome b small subunit, mitochondrial	AVAMLWK	461.68	344.77	401.72	442.53
Succinate dehydrogenase [ubiquinone] cytochrome b small subunit, mitochondrial	AVAMLWK	329.31	656.17	695.4	1203.58
Succinate dehydrogenase [ubiquinone] cytochrome b small subunit, mitochondrial	AVAMLWK	538.74	816.02	1275.65	1124.36
Succinate dehydrogenase [ubiquinone] cytochrome b small subunit, mitochondrial	AVAMLWK	851.35	569.11	589	635.4
Succinate dehydrogenase [ubiquinone] cytochrome b small subunit, mitochondrial	GLLP	206.92	0	95.22	278.73
Succinate dehydrogenase [ubiquinone] cytochrome b small subunit, mitochondrial	LAAAL	360.02	175.04	234.41	373.2

Succinate dehydrogenase [ubiquinone] cytochrome b small subunit, mitochondrial	LGLL	1296.99	1434.54	5346.49	5340.23
Succinate dehydrogenase [ubiquinone] cytochrome b small subunit, mitochondrial	LGLL	667.11	261.48	2492.45	2908.78
Succinate dehydrogenase [ubiquinone] cytochrome b small subunit, mitochondrial	LGLL	3314.76	3998.01	22099.16	20825.3
Succinate dehydrogenase [ubiquinone] cytochrome b small subunit, mitochondrial	LGLL	3659.85	3414.84	18140.3	21295.9
Succinate dehydrogenase [ubiquinone] cytochrome b small subunit, mitochondrial	LGLL	332.65	326.7	492.06	841.9
Succinate dehydrogenase [ubiquinone] cytochrome b small subunit, mitochondrial	LLAL	196.22	188.4	226.96	250.42
Succinate dehydrogenase [ubiquinone] cytochrome b small subunit, mitochondrial	LLAL	75.78	172.69	115.6	69.95
Succinate dehydrogenase [ubiquinone] cytochrome b small subunit, mitochondrial	LLLR	386.99	489.76	684.01	680.12
Succinate dehydrogenase [ubiquinone] cytochrome b small subunit, mitochondrial	LLLR	769.62	771.66	1081.49	1259.46
Succinate dehydrogenase [ubiquinone] cytochrome b small subunit, mitochondrial	LLLR	864.03	699.71	1475.57	835.82
Succinate dehydrogenase [ubiquinone] cytochrome b small subunit, mitochondrial	LLLR	912.1	950.18	1143.27	1280.65
Succinate dehydrogenase [ubiquinone] cytochrome b small subunit, mitochondrial	LLLR	556.67	959.36	927.48	1147.92
Succinate dehydrogenase [ubiquinone] cytochrome b small subunit, mitochondrial	LLLR	1415.65	1626.01	1823.04	1846.6
Succinate dehydrogenase [ubiquinone] cytochrome b small subunit, mitochondrial	LLLR	279.39	481.67	627.51	552.92
Succinate dehydrogenase [ubiquinone] cytochrome b small subunit, mitochondrial	LLLR	487.03	393.55	449.11	438.94
Gamma-glutamyl hydrolase	NLDGISHAPNAVK	407.77	563.8	774.94	997.54
Gamma-glutamyl hydrolase	YYIAASYVK	611.99	494.2	1288.7	898.75
Gamma-glutamyl hydrolase	ILFK	2470.97	2256.62	2983.58	3778.41
Gamma-glutamyl hydrolase	ILFK	2258.5	2184.3	3616.36	2876.62
Gamma-glutamyl hydrolase	ILFK	2361.06	3115.46	4624.91	5380.97
Gamma-glutamyl hydrolase	ILFK	1293.11	2456.52	2411.93	2123.8
Gamma-glutamyl hydrolase	ILFK	3623.08	1834.68	3835.96	5244.06
Gamma-glutamyl hydrolase	LDLTEK	1098.04	1090.06	2158.79	1517.13
Gamma-glutamyl hydrolase	LGLL	1296.99	1434.54	5346.49	5340.23
Gamma-glutamyl hydrolase	LGLL	667.11	261.48	2492.45	2908.78
Gamma-glutamyl hydrolase	LGLL	3314.76	3998.01	22099.16	20825.3
Gamma-glutamyl hydrolase	LGLL	3659.85	3414.84	18140.3	21295.9
Gamma-glutamyl hydrolase	LGLL	332.65	326.7	492.06	841.9
Gamma-glutamyl hydrolase	NLDGISHAPNAVK	1442.1	1247.07	2652.48	2549.56
Gamma-glutamyl hydrolase	YYIAASYVK	427.49	318.34	557.89	839.59
Gamma-glutamyl hydrolase	YYIAASYVK	459.02	607.57	762.61	812.23
Y-box-binding protein 3	DGVPEGAQLQGPVHR	425.78	699.7	528.67	1432.7
Y-box-binding protein 3	GAEAANVTGPDGVPVEGSR	954.42	918.53	904.65	1139.46
Y-box-binding protein 3	IQAGEIGEMKDGVPEGAQLQGPVHR	483.24	767.17	969.97	1574.84
Y-box-binding protein 3	DGVPEGAQLQGPVHR	558.3	585.36	858.98	1321.85
Y-box-binding protein 3	DGVPEGAQLQGPVHR	380.39	716.05	737.23	1253.87
Y-box-binding protein 3	EDVFVHQTAIK	332.94	297.58	654.88	631.44
Y-box-binding protein 3	EDVFVHQTAIK	201.82	486.55	723.59	496.48
Y-box-binding protein 3	EDVFVHQTAIK	432.47	783.41	938.01	1002.68
Y-box-binding protein 3	GAEAANVTGPDGVPVEGSR	954.21	832.06	1618.96	2908.72
Y-box-binding protein 3	GAEAANVTGPDGVPVEGSR	1014.91	473.35	896.88	832.2
Y-box-binding protein 3	GAEAANVTGPDGVPVEGSR	828.04	1276.37	1698.29	1887.1
Y-box-binding protein 3	GAEAANVTGPDGVPVEGSR	202.63	405.91	879.98	677.42
Y-box-binding protein 3	GAEAANVTGPDGVPVEGSR	536.5	612.16	730.18	1121.32
Y-box-binding protein 3	GAEAANVTGPDGVPVEGSR	849.46	756.06	1932.69	1796.01
Y-box-binding protein 3	GAEAANVTGPDGVPVEGSR	849.46	756.06	1932.69	1796.01
Y-box-binding protein 3	GAEAANVTGPDGVPVEGSR	849.46	756.06	1932.69	1796.01
Y-box-binding protein 3	NDTKEDVFVHQTAIK	788.95	670.31	1662.17	1267.71
Y-box-binding protein 3	NDTKEDVFVHQTAIK	1199.14	996	3994.87	4159.1
Y-box-binding protein 3	NGYGFINR	2010.85	3140.89	7788.62	7482.09
Y-box-binding protein 3	NGYGFINR	4012.75	4612.61	18393.51	21172.73
Y-box-binding protein 3	NGYGFINR	3176.86	3260.67	7005.02	8186.4
Y-box-binding protein 3	NGYGFINR	2645.23	4294.92	7843.94	7870.42
Y-box-binding protein 3	NGYGFINR	3889.74	4531.72	9441.4	9869.68
Y-box-binding protein 3	NGYGFINR	4061.56	3513.59	8857.19	10039.02
Y-box-binding protein 3	VLGTVK	2175.54	1870.04	8402.6	9538.78
Y-box-binding protein 3	VLGTVK	1904.25	2183.47	8998.43	11036.44
Y-box-binding protein 3	VLGTVK	2813.16	2810.19	10409.63	12165.63
Y-box-binding protein 3	VLGTVK	2173.26	2094.91	7233.67	7950.31
Y-box-binding protein 3	VLGTVK	3072.44	3683.1	10746.22	12183.84
Y-box-binding protein 3	VLGTVK	1474.8	1739.5	3226.15	3037.44

Y-box-binding protein 3	VLGTVK	2893.78	2505.13	8130.23	9659.56
Y-box-binding protein 3	VLGTVK	1138.28	1050.68	2789.36	3717.21
Y-box-binding protein 3	VLGTVK	1456.23	1978.69	2259.74	2606.42
Y-box-binding protein 3	VLGTVK	720.88	586.78	1788.71	2517.57
Y-box-binding protein 3	WFNVR	2035.79	2485.23	4971.8	6900.76
Histone H4	DAVTYTEHAK	460.95	416.95	663.64	861.77
Histone H4	DNIQGITKPAIR	4922.63	4506.15	16016.59	15522.26
Histone H4	ISGLIYEETR	967.21	1356.5	1320.11	1286.92
Histone H4	KTVTAMDVVYALK	1108.02	1062.31	4239.85	4593.03
Histone H4	RISGLIYEETR	2867.89	2722.21	3550.37	4549.44
Histone H4	TVTAMDVVYALK	212.02	117.1	292.18	336.98
Histone H4	TVTAMDVVYALKR	368.44	407.26	1109.68	1693.77
Histone H4	VFLENVIR	1403.72	1102.22	3681.63	4300.5
Histone H4	TLYGFGG	1611.12	2100.96	4473.44	4447.47
Histone H4	DAVTYTEHAKR	906.86	829.78	2756.65	2939.61
Histone H4	TVTAMDVVY	359.14	262.77	1004.3	510.99
Histone H4	DNIQGITKPAIRR	1410.94	2197.67	2072.02	2740.09
Histone H4	DNIQGITK	1490.72	1336.71	2880.42	3271.52
Histone H4	TLYGF	538	357.37	775.58	900.08
Histone H4	SGLIYEETR	592.87	574.6	1264.52	850.08
Histone H4	KTVTAMDVVY	1528.96	1601.9	3185.72	4193.07
Histone H4	ISGLIYEETRGV	619.9	1201.96	1833.19	1792.92
Histone H4	TVTAMDVVYA	231.53	104.4	369.85	314.79
Histone H4	DAVTYTEHAK	228.42	411.16	685.04	576.95
Histone H4	DAVTYTEHAK	1293.31	1692.14	6180.8	7217.49
Histone H4	DAVTYTEHAK	2230.34	1988.05	8374.44	8726.53
Histone H4	DAVTYTEHAK	331.2	469.86	1074.88	762.75
Histone H4	DAVTYTEHAK	152.37	264.96	377.22	593.54
Histone H4	DAVTYTEHAK	294.49	479.92	605.33	754.44
Histone H4	DAVTYTEHAK	610.45	539.73	1116.47	1276.32
Histone H4	DAVTYTEHAK	459.11	385.45	792.68	850.8
Histone H4	DAVTYTEHAK	222.29	165.93	551.54	570.42
Histone H4	DAVTYTEHAK	25.54	30.07	333.77	402.61
Histone H4	DAVTYTEHAK	1063.2	1340.66	2389.92	4212.07
Histone H4	DAVTYTEHAK	860.01	1309.72	3282.36	3161.97
Histone H4	DAVTYTEHAK	201.4	527.52	719.73	679.19
Histone H4	DAVTYTEHAK	324.83	1098.96	1999.42	2181.08
Histone H4	DAVTYTEHAK	397.85	693.74	766.33	939.75
Histone H4	DAVTYTEHAK	504.43	482.89	1338.05	1286.74
Histone H4	DAVTYTEHAK	989.45	967.6	3295.66	3850.01
Histone H4	DAVTYTEHAK	570	706.97	1023.91	2180.3
Histone H4	DAVTYTEHAK	510.32	326.5	648.81	756.32
Histone H4	DAVTYTEHAK	457.15	791.95	903.44	1626.65
Histone H4	DAVTYTEHAK	1664	3260.23	7198.88	8230.62
Histone H4	DAVTYTEHAK	285.1	343.54	1003.44	270.16
Histone H4	DAVTYTEHAK	7121.58	8104.95	27751.19	33434.08
Histone H4	DAVTYTEHAK	355.94	578.63	1614.36	1015.84
Histone H4	DAVTYTEHAK	206.01	72.11	784.11	215.52
Histone H4	DAVTYTEHAK	593.29	426.29	514.59	609.69
Histone H4	DAVTYTEHAK	7632.55	9005.43	31173.71	39230.44
Histone H4	DAVTYTEHAK	1891	1683.45	4952.19	5164.98
Histone H4	DAVTYTEHAK	563.98	450.37	1133.03	1702.69
Histone H4	DAVTYTEHAK	892.86	1148.9	3032.13	2111.56
Histone H4	DAVTYTEHAK	597.7	629.18	993.52	931.24
Histone H4	DAVTYTEHAK	38.29	46.85	95.53	147.56
Histone H4	DAVTYTEHAK	2192.38	3184.81	7504.02	9114.84
Histone H4	DAVTYTEHAK	230.56	199.86	757.58	786.33
Histone H4	DAVTYTEHAK	440.51	414.93	997.91	1638.26
Histone H4	DAVTYTEHAK	357.84	390.67	1064.75	870.89
Histone H4	DAVTYTEHAK	680.89	814.57	1092.56	1555.45
Histone H4	DAVTYTEHAK	153.8	123.66	340.27	265.07
Histone H4	DAVTYTEHAK	892.86	1148.9	3032.13	2111.56
Histone H4	DAVTYTEHAK	788.37	621.78	1086.63	941.37
Histone H4	DAVTYTEHAK	716.67	364.79	1030.21	1433.7

Histone H4	DAVTYTEHAK	153.8	123.66	340.27	265.07
Histone H4	DAVTYTEHAK	2073.94	2120.65	7228.98	8626.74
Histone H4	DAVTYTEHAK	382.85	472.71	744.53	704.82
Histone H4	DAVTYTEHAK	4076.22	4193.14	14921.88	18354.12
Histone H4	DAVTYTEHAK	1379.41	1270.51	5658.57	7637.76
Histone H4	DAVTYTEHAK	563.98	450.37	1133.03	1702.69
Histone H4	DAVTYTEHAK	510.32	326.5	648.81	756.32
Histone H4	DAVTYTEHAK	357.48	426.89	1827.81	1450.68
Histone H4	DAVTYTEHAK	153.8	123.66	340.27	265.07
Histone H4	DAVTYTEHAK	487.44	352.11	1220.87	786.41
Histone H4	DAVTYTEHAK	140.46	165.71	224.22	322.17
Histone H4	DAVTYTEHAK	563.98	450.37	1133.03	1702.69
Histone H4	DAVTYTEHAK	383.84	375.23	456.82	727.68
Histone H4	DAVTYTEHAK	808.4	663.55	1818.96	2379.31
Histone H4	DAVTYTEHAK	345	595.53	711.84	785.85
Histone H4	DAVTYTEHAK	776.41	1058.6	1702.33	2036.64
Histone H4	DAVTYTEHAK	1094.35	816.54	2192.2	3150.44
Histone H4	DAVTYTEHAK	3546.09	3590.44	10402.51	12794.54
Histone H4	DAVTYTEHAK	1891	1683.45	4952.19	5164.98
Histone H4	DAVTYTEHAK	801.97	766.67	2007.68	1783.5
Histone H4	DAVTYTEHAK	1117.06	807.18	2838.28	2826.3
Histone H4	DAVTYTEHAK	366.89	348.1	584.3	831.76
Histone H4	DAVTYTEHAK	776.41	1058.6	1702.33	2036.64
Histone H4	DAVTYTEHAK	153.8	123.66	340.27	265.07
Histone H4	DAVTYTEHAK	9012.78	10833.43	36273.07	44679.3
Histone H4	DAVTYTEHAK	337.81	241.71	794.42	998.16
Histone H4	DAVTYTEHAK	202.12	137.55	539.99	551.71
Histone H4	DAVTYTEHAK	140.46	165.71	224.22	322.17
Histone H4	DAVTYTEHAKR	692.67	714.61	2164.26	2653.41
Histone H4	DNIQGITK	3273.18	3106.14	5147.02	5957.24
Histone H4	DNIQGITK	307.57	250.74	511.87	382.21
Histone H4	DNIQGITK	1580.46	1939.18	4822.8	5240.22
Histone H4	DNIQGITK	1849.32	1440.43	2392.87	3059.47
Histone H4	DNIQGITK	7139.57	6647.18	16713.2	18168.82
Histone H4	DNIQGITK	179.32	155.97	563.43	627.99
Histone H4	DNIQGITK	256.15	225.17	688.34	819.96
Histone H4	DNIQGITK	6513.32	9051.19	13330.95	17462.74
Histone H4	DNIQGITK	7429.96	7744.65	12742.3	14629.75
Histone H4	DNIQGITK	2989.85	3545.25	5101.93	5695.82
Histone H4	DNIQGITKPAIR	1003.05	794.62	2281.76	3650.81
Histone H4	DNIQGITKPAIR	1968.5	5197.35	14406.2	7746.2
Histone H4	DNIQGITKPAIR	2585.84	4843.53	17252.81	9938.51
Histone H4	DNIQGITKPAIR	787.12	957.28	2898.62	2424.27
Histone H4	DNIQGITKPAIR	1994.96	3221.71	7731.97	8793.85
Histone H4	DNIQGITKPAIR	1658.3	950.96	3422.81	3956.04
Histone H4	DNIQGITKPAIR	955.18	1161.75	1795.75	2799.92
Histone H4	DNIQGITKPAIR	1094.74	1309.83	2044.35	3523.84
Histone H4	DNIQGITKPAIR	890.32	868.48	1753.83	2779.12
Histone H4	DNIQGITKPAIR	1089.96	1144.1	2281.29	3459
Histone H4	DNIQGITKPAIR	3217.32	3411.96	9000.03	9223.78
Histone H4	DNIQGITKPAIR	891.34	980.2	1382.92	2100.83
Histone H4	DNIQGITKPAIR	705.74	700.15	1502.09	2101.43
Histone H4	DNIQGITKPAIR	2051.27	2019.74	7091.13	7257.52
Histone H4	DNIQGITKPAIR	1948.07	1576.96	5862.07	7036.92
Histone H4	DNIQGITKPAIR	1718.38	1184.97	2690.61	3134.55
Histone H4	DNIQGITKPAIR	702.77	567.65	2375.74	3246.5
Histone H4	DNIQGITKPAIR	1128.29	973.79	2480.43	3224.49
Histone H4	DNIQGITKPAIR	830.95	776.31	1743.4	2472.43
Histone H4	DNIQGITKPAIR	1538.87	1898.72	2348.92	2444.05
Histone H4	DNIQGITKPAIR	1934.23	1242.26	3108.12	3285.57
Histone H4	DNIQGITKPAIR	535.68	586.22	792.98	1407.15
Histone H4	DNIQGITKPAIR	457.64	530.69	1325.99	1269.61
Histone H4	DNIQGITKPAIR	5083.26	4487.31	11093.72	10944.07
Histone H4	DNIQGITKPAIR	539.52	419.8	926.37	894.31

Histone H4	DNIQGITKPAIR	457.46	1080.93	1873.37	1760
Histone H4	DNIQGITKPAIR	3160.81	2825.07	6697.11	10656.38
Histone H4	DNIQGITKPAIR	980.98	1060.22	1830.09	1731.28
Histone H4	DNIQGITKPAIR	539.3	548.03	1338.99	1435.49
Histone H4	DNIQGITKPAIR	892.25	996.8	1196.75	1795.71
Histone H4	DNIQGITKPAIR	3296.59	3346.84	8314.25	9304.78
Histone H4	DNIQGITKPAIR	14096.2	14984.59	51867.17	64125.11
Histone H4	DNIQGITKPAIR	3630.08	4121.47	11538.48	15265.72
Histone H4	DNIQGITKPAIR	5174.58	4188.45	11394.45	12155.1
Histone H4	DNIQGITKPAIR	597.7	523.25	2457.48	2388
Histone H4	DNIQGITKPAIR	534.8	673.97	1295.67	1575
Histone H4	DNIQGITKPAIR	3764.33	3518.12	7169.34	7598.84
Histone H4	DNIQGITKPAIR	16789.32	20293.36	54435.57	72800.74
Histone H4	DNIQGITKPAIR	6249.22	5037.36	12855.57	12610.84
Histone H4	DNIQGITKPAIR	774.33	945.01	2169.81	1165.3
Histone H4	DNIQGITKPAIR	1195.33	733.32	1577.12	1213.06
Histone H4	DNIQGITKPAIR	5309.26	6839.52	20395.7	23523.11
Histone H4	DNIQGITKPAIR	3866.14	4629.73	11487.89	10003.77
Histone H4	DNIQGITKPAIR	481.03	879.38	1810.18	1146.86
Histone H4	DNIQGITKPAIR	3869.02	5302.23	13156.85	16352.93
Histone H4	DNIQGITKPAIR	4442.47	3965.78	8602.99	9300.03
Histone H4	DNIQGITKPAIR	1239.5	1263.62	2044.07	3440.17
Histone H4	DNIQGITKPAIR	1480.12	2384.77	3733.29	4054.34
Histone H4	DNIQGITKPAIR	382.68	489.7	596.34	739.48
Histone H4	DNIQGITKPAIR	3764.33	3518.12	7169.34	7598.84
Histone H4	DNIQGITKPAIR	2493.33	2706.81	5950.71	7162.52
Histone H4	DNIQGITKPAIR	1326.83	3060.47	10177.83	1191.86
Histone H4	DNIQGITKPAIR	176.53	431.36	993.16	717.75
Histone H4	DNIQGITKPAIR	1554.21	1873.29	2327.57	2598.83
Histone H4	DNIQGITKPAIR	7183.93	8541.03	24833.65	23891.31
Histone H4	DNIQGITKPAIR	2201.2	2313.53	4508.39	4355.41
Histone H4	DNIQGITKPAIR	4952.8	8014.37	18797.28	13841.14
Histone H4	DNIQGITKPAIR	5475.11	5772.92	14784.5	14492.76
Histone H4	DNIQGITKPAIR	1199.43	1179.77	3413.54	2863.29
Histone H4	DNIQGITKPAIR	4503.65	7069.78	18606.64	12319.48
Histone H4	DNIQGITKPAIR	1412.67	1920.99	3145.91	4318.24
Histone H4	DNIQGITKPAIR	1684.48	1449.65	3551.2	3070.27
Histone H4	DNIQGITKPAIR	3230.01	5350.09	10524.73	7551.36
Histone H4	DNIQGITKPAIR	8004.32	10679.77	28687.1	32990.99
Histone H4	DNIQGITKPAIR	4391.8	6099.47	13558.37	11632.42
Histone H4	DNIQGITKPAIR	27609.3	28323.27	99844.62	113320.4
Histone H4	DNIQGITKPAIR	1326.83	3060.47	10177.83	1191.86
Histone H4	DNIQGITKPAIR	2542.09	2463.76	4923.15	4929.32
Histone H4	DNIQGITKPAIR	2173.09	2218.66	5740.31	6361.11
Histone H4	DNIQGITKPAIR	9651.96	11194.05	34336.01	44633.75
Histone H4	DNIQGITKPAIR	459.58	445.66	844.46	1356.25
Histone H4	DNIQGITKPAIR	2173.09	2218.66	5740.31	6361.11
Histone H4	DNIQGITKPAIR	8084.43	8829.01	25585.22	34200.88
Histone H4	DNIQGITKPAIR	1310.58	1368.95	3541.15	2963.53
Histone H4	DNIQGITKPAIR	461.99	314.3	631.64	927.1
Histone H4	DNIQGITKPAIR	5010.18	4579.79	15191	15841.33
Histone H4	DNIQGITKPAIR	10493.49	10393.25	31929.51	41229.09
Histone H4	DNIQGITKPAIR	11801	11958.16	24192.52	29003.18
Histone H4	DNIQGITKPAIR	1326.83	3060.47	10177.83	1191.86
Histone H4	DNIQGITKPAIR	4259.6	5011.29	9681.35	11838.49
Histone H4	DNIQGITKPAIR	4101.47	4783.39	9886.15	10453.87
Histone H4	DNIQGITKPAIR	5010.18	4579.79	15191	15841.33
Histone H4	DNIQGITKPAIR	750.68	515.59	1584.23	1509.52
Histone H4	DNIQGITKPAIR	4805.93	4759.27	8603.78	11556.6
Histone H4	DNIQGITKPAIR	1198.25	1403.06	3222.92	3371.67
Histone H4	DNIQGITKPAIR	3399.33	2983.66	11062.83	10922.17
Histone H4	DNIQGITKPAIR	1850.34	1339.16	3409.16	4218.22
Histone H4	DNIQGITKPAIR	457.88	720.06	1212.51	1749.09
Histone H4	DNIQGITKPAIR	1688.67	1673.29	2419.92	3183.26

Histone H4	DNIQGITKPAIR	3431.08	3144.36	6613.81	4411.7
Histone H4	DNIQGITKPAIR	5132.5	5047.34	11826.62	13718.17
Histone H4	DNIQGITKPAIR	3296.59	3346.84	8314.25	9304.78
Histone H4	DNIQGITKPAIR	331.37	453.28	618.09	914.81
Histone H4	DNIQGITKPAIR	604.82	670.13	963.24	1524.86
Histone H4	DNIQGITKPAIR	12624.28	14024.72	33255.01	36173.29
Histone H4	DNIQGITKPAIR	1808.15	2209.39	5355.4	4159.78
Histone H4	DNIQGITKPAIR	1497.35	2171.39	3321.14	3921.96
Histone H4	DNIQGITKPAIR	600.21	594.18	1492.67	798.5
Histone H4	DNIQGITKPAIR	1116.73	1477.59	2175.28	2497.05
Histone H4	DNIQGITKPAIR	822.08	801.26	1605.67	1223.3
Histone H4	DNIQGITKPAIR	999.08	1611.92	1599.77	1615.33
Histone H4	DNIQGITKPAIR	1311.51	1063.9	1766.47	2450.39
Histone H4	DNIQGITKPAIR	592.21	852.63	1130.53	1613.53
Histone H4	DNIQGITKPAIR	2226.93	2111.59	3711.84	4773.17
Histone H4	DNIQGITKPAIR	18758.08	20784.43	58353.54	77052.05
Histone H4	DNIQGITKPAIRR	2352.21	2393.95	2104.42	2882.84
Histone H4	DNIQGITKPAIRR	1700.74	2182.85	2115.16	2352.15
Histone H4	DNIQGITKPAIRR	3495.89	4400.84	6295.35	7329.65
Histone H4	DNIQGITKPAIRR	3496.7	3788.55	5570.69	6013.07
Histone H4	DNIQGITKPAIRR	2090.64	1853.2	1887.27	1665.81
Histone H4	DNIQGITKPAIRR	305.11	493.82	1155.7	988.56
Histone H4	DNIQGITKPAIRR	1153.96	1203.67	1387.16	2032.29
Histone H4	DNIQGITKPAIRR	3311.39	4118.24	5100.01	7622.97
Histone H4	DNIQGITKPAIRR	3083.95	4141.82	4607.74	5967.51
Histone H4	DVVYALK	2336.17	2276.24	4687.94	4165.44
Histone H4	DVVYALK	2366.66	2561.91	3795.63	4131.2
Histone H4	GGAK	216.55	307.68	954	707.01
Histone H4	GGAK	91.68	90.7	336.75	278.3
Histone H4	GGAK	255.36	302.47	590.89	932.43
Histone H4	GLGK	314.32	435.35	609.97	798.82
Histone H4	GLGK	272.38	216.83	403.38	919.32
Histone H4	ISGLIYEETR	2156.8	1486.72	2273.73	2367.04
Histone H4	ISGLIYEETR	517.63	455.28	1006.8	1431.55
Histone H4	ISGLIYEETR	2678.95	1708.39	6149.84	6880.7
Histone H4	ISGLIYEETR	8446.71	8887.17	21859.31	26695.22
Histone H4	ISGLIYEETR	894.97	834.53	1661.04	1871.64
Histone H4	ISGLIYEETR	8611.9	8418.08	19884.27	24760.31
Histone H4	ISGLIYEETR	1112.2	967.82	1717.83	1472.91
Histone H4	ISGLIYEETR	408.13	528.74	380.26	735.67
Histone H4	ISGLIYEETR	10617.03	12442.75	31634.65	34579.53
Histone H4	ISGLIYEETR	10789.69	10597.16	27937.95	29464.33
Histone H4	ISGLIYEETR	23066.08	19312.08	57101.7	68794.46
Histone H4	ISGLIYEETR	346.88	196.7	487.79	437.41
Histone H4	ISGLIYEETR	1308.36	1374.82	1610.05	2213.99
Histone H4	ISGLIYEETR	2949.28	3085.11	4874.81	5868.43
Histone H4	ISGLIYEETR	46299.8	53975.5	153177	168432.1
Histone H4	ISGLIYEETR	583.11	900.18	1843.53	1052.73
Histone H4	ISGLIYEETR	3269.56	3251.47	4939.39	6411.69
Histone H4	ISGLIYEETR	1602.46	1467.88	2152.28	3198.96
Histone H4	ISGLIYEETR	1465.46	1385.64	2148.29	3128.14
Histone H4	ISGLIYEETR	179.75	113.06	131.23	95.63
Histone H4	ISGLIYEETR	790.12	554.63	465.79	413.03
Histone H4	ISGLIYEETR	128.09	111.41	402.35	369.88
Histone H4	ISGLIYEETR	110.07	83.16	110.36	144.11
Histone H4	ISGLIYEETR	116	28.78	66.14	86.23
Histone H4	ISGLIYEETR	72.71	50.72	43.44	119.83
Histone H4	ISGLIYEETR	306.83	324.41	699.85	546.25
Histone H4	ISGLIYEETR	290.19	53.08	140.61	43.75
Histone H4	ISGLIYEETR	1677.69	843.68	1477.78	1384.67
Histone H4	ISGLIYEETR	552.4	424.42	1235.45	606.21
Histone H4	ISGLIYEETR	359.76	200.88	233.55	347.61
Histone H4	ISGLIYEETR	879.06	385.06	442.96	627.48
Histone H4	KTVTAMDVVYALK	178.01	285.46	758.48	794.19

Histone H4	KTVTAMDVVYALK	381.6	596.3	1390.93	1194.97
Histone H4	KTVTAMDVVYALK	307.39	268.61	904.98	1087.94
Histone H4	LENVIR	1329.32	1325.13	1225.56	2319.11
Histone H4	LENVIR	1682.15	1785.71	2414.36	2775.06
Histone H4	LYGFGG	1642.73	1212.21	450.08	333.24
Histone H4	LYGFGG	496.74	923.42	318.79	264.54
Histone H4	LYGFGG	1359.64	1628.18	271.84	797.62
Histone H4	LYGFGG	940.94	547.56	262.48	319.56
Histone H4	LYGFGG	1958.2	2066.16	507.94	864
Histone H4	RDNIQGITKPAIR	1146.57	2998.17	8015.96	1871.12
Histone H4	RDNIQGITKPAIR	1341.32	3012.14	8100.69	1938.45
Histone H4	RISGLIYEETR	3005.31	2549.88	5029.15	5359.45
Histone H4	RISGLIYEETR	1776.42	1834.01	2870.92	2872.48
Histone H4	RISGLIYEETR	1827.58	1992.09	4065.58	3802.21
Histone H4	RISGLIYEETR	487.38	358.08	925.83	622.42
Histone H4	RISGLIYEETR	307.56	252.17	440.9	204.32
Histone H4	RISGLIYEETR	1048.88	1054.09	1618.74	1095.36
Histone H4	RISGLIYEETR	835.23	566.35	1263.04	1535.3
Histone H4	RISGLIYEETR	473.8	423.47	294.24	421.38
Histone H4	RISGLIYEETR	473.8	423.47	294.24	421.38
Histone H4	SGLIYEETR	653.72	520.12	811.15	859.8
Histone H4	SGLIYEETR	389.18	592.86	894.9	657.94
Histone H4	TLYGF	488.85	213.67	404.65	581.32
Histone H4	TLYGF	411.04	240.98	615.24	457.28
Histone H4	TLYGFGG	1014.59	824.05	1900.61	2451.06
Histone H4	TLYGFGG	4807.53	5983.26	14698.57	17769.06
Histone H4	TLYGFGG	2816.33	2071.66	4644.32	6356.28
Histone H4	TLYGFGG	2531.08	2381.08	6074.76	6968.55
Histone H4	TLYGFGG	153.65	138.61	239.58	296.68
Histone H4	TLYGFGG	965.5	781.01	1975.28	2740.5
Histone H4	TLYGFGG	25.11	72.71	140.85	300.98
Histone H4	TLYGFGG	492.63	158.56	563.74	408.53
Histone H4	TLYGFGG	101.41	193.75	256.62	474.09
Histone H4	TLYGFGG	101.93	141.94	264.55	320.26
Histone H4	TLYGFGG	67.95	94.91	162.24	76.24
Histone H4	TLYGFGG	1052.49	1016.15	1301.39	2294.13
Histone H4	TLYGFGG	179.5	137.85	182.49	478.12
Histone H4	TLYGFGG	229.15	339.62	232.11	116.16
Histone H4	TLYGFGG	73.47	400.81	63.18	47.6
Histone H4	TLYGFGG	257.37	103.98	296.26	190.57
Histone H4	TLYGFGG	204.89	183.23	398.04	497.86
Histone H4	TLYGFGG	63.25	133.86	167.76	132.28
Histone H4	TLYGFGG	64.55	5.49	23.74	50.33
Histone H4	TLYGFGG	102.96	40.55	170.09	245.77
Histone H4	TLYGFGG	42.41	65.27	20.01	50.11
Histone H4	TLYGFGG	723.16	787.85	1621.2	2130.57
Histone H4	TLYGFGG	1364.16	969	1534	2238.98
Histone H4	TLYGFGG	671.11	930.08	1903.33	2769.95
Histone H4	TLYGFGG	205.09	162.79	131.53	405.47
Histone H4	TLYGFGG	358.08	367.57	930.48	922.11
Histone H4	TLYGFGG	205.12	159.7	284.31	472.87
Histone H4	TLYGFGG	76.57	95.11	120.9	57.52
Histone H4	TLYGFGG	18472.24	16258.2	51762.74	57833.91
Histone H4	TLYGFGG	128.61	59.85	397.97	339
Histone H4	TLYGFGG	427.99	1120.21	1155.23	1778.31
Histone H4	TLYGFGG	977.9	726.01	2788.8	1308.25
Histone H4	TLYGFGG	48.93	271.82	416.81	131.31
Histone H4	TLYGFGG	154.19	85.36	318.86	342.08
Histone H4	TLYGFGG	128.11	109.23	510.23	358.8
Histone H4	TLYGFGG	696.43	342.91	910.04	730.85
Histone H4	TLYGFGG	231.53	104.37	371.4	263.99
Histone H4	TLYGFGG	76.82	70.35	68.1	150.61
Histone H4	TLYGFGG	304.28	576.01	816.11	1430.54
Histone H4	TLYGFGG	384.77	283.43	1005.39	1209.47

Histone H4	TLYGFGG	155.68	44.99	182.71	135.77
Histone H4	TLYGFGG	521.01	333.44	1117.32	908.97
Histone H4	TLYGFGG	1255.3	1830.07	3273.87	4164.25
Histone H4	TLYGFGG	1604.63	1572.36	3465.41	3806.55
Histone H4	TLYGFGG	76.87	64.75	25.82	69.2
Histone H4	TLYGFGG	879.35	568.85	1988.37	2010.64
Histone H4	TLYGFGG	1059.27	665.84	1158.86	1950.94
Histone H4	TLYGFGG	154.66	38.69	72.25	73.51
Histone H4	TLYGFGG	41.71	28.19	48.43	75.64
Histone H4	TLYGFGG	348.11	75.69	86.36	80.6
Histone H4	TLYGFGG	1067.09	1063.28	1894.98	1847.97
Histone H4	TLYGFGG	306.79	327.9	527.28	776.68
Histone H4	TLYGFGG	382.09	548.6	823.35	885.45
Histone H4	TLYGFGG	510.49	416.17	1797.33	1721.05
Histone H4	TLYGFGG	1336.12	866.81	1319.6	1234.92
Histone H4	TLYGFGG	705.27	640.3	2974.67	3243.95
Histone H4	TLYGFGG	433.58	567.07	1476.33	698.46
Histone H4	TLYGFGG	77.01	50.8	77.35	99.01
Histone H4	TLYGFGG	103.21	15.4	136.44	134.65
Histone H4	TLYGFGG	232.27	30.87	175.02	93.91
Histone H4	TLYGFGG	17201.03	17170.37	46031.98	53595.04
Histone H4	TLYGFGG	124.57	458.69	44.52	112.93
Histone H4	TLYGFGG	50.19	147.82	163.38	249.09
Histone H4	TLYGFGG	1591.97	1333.41	2566.1	2586.11
Histone H4	TLYGFGG	167.74	23.25	149.85	95.27
Histone H4	TLYGFGG	51.35	32.91	98.69	72.13
Histone H4	TVTAMDVVY	947.92	1241.28	1505.31	2754
Histone H4	TVTAMDVVY	1470.9	1167.6	4031.22	2719.88
Histone H4	TVTAMDVVY	434.79	448.23	488.9	538.93
Histone H4	TVTAMDVVY	282.99	125.95	391.82	391.05
Histone H4	TVTAMDVVY	475.66	240.13	420.63	815.95
Histone H4	TVTAMDVVY	179.25	162.6	235.66	373.16
Histone H4	TVTAMDVVY	178.52	235.42	465.9	490.92
Histone H4	TVTAMDVVY	102.97	39.91	201.37	66.74
Histone H4	TVTAMDVVY	434.79	448	500.3	697.25
Histone H4	TVTAMDVVY	475.66	240.13	420.63	815.95
Histone H4	TVTAMDVVYALK	2139.93	2302.7	6651.33	8734.72
Histone H4	TVTAMDVVYALK	211.31	186.39	590.47	803.18
Histone H4	TVTAMDVVYALK	394.08	640.2	1248.09	1385.69
Histone H4	TVTAMDVVYALK	534.2	732.92	1732.84	2371.89
Histone H4	TVTAMDVVYALK	290.41	137.44	647.62	678.58
Histone H4	TVTAMDVVYALK	3641.43	3958.3	11749.48	14488.25
Histone H4	TVTAMDVVYALK	1020.63	1185.09	3007.79	4000.29
Histone H4	TVTAMDVVYALK	155.07	104.91	252.7	252.57
Histone H4	TVTAMDVVYALK	88.87	49.92	130.21	91.93
Histone H4	TVTAMDVVYALK	229.7	284.9	383.73	329.87
Histone H4	TVTAMDVVYALK	1133.77	1496.29	2304.82	3537.75
Histone H4	TVTAMDVVYALK	512.03	476.63	1407.13	1357.88
Histone H4	TVTAMDVVYALK	2482.4	2935.61	8342.7	11884.13
Histone H4	TVTAMDVVYALK	371.36	437.82	916.89	867.23
Histone H4	TVTAMDVVYALK	316	162.76	365.06	414.71
Histone H4	TVTAMDVVYALK	1162.49	680.01	2526.41	2889.86
Histone H4	TVTAMDVVYALK	2051.04	2042.78	6111.49	7679.43
Histone H4	TVTAMDVVYALK	38.16	59.33	116.65	276.41
Histone H4	TVTAMDVVYALK	688.37	1139.02	1078.94	1150.33
Histone H4	TVTAMDVVYALK	741.67	660.33	1478.41	1543.95
Histone H4	TVTAMDVVYALK	1191.43	1225.03	3384.46	3553.49
Histone H4	TVTAMDVVYALK	1059.27	1410.08	3012.86	2933.13
Histone H4	TVTAMDVVYALK	347.32	259.9	551.03	663.24
Histone H4	TVTAMDVVYALK	204.37	233.73	454.55	618.54
Histone H4	TVTAMDVVYALK	1004.11	1008.63	2382.26	2509.14
Histone H4	TVTAMDVVYALK	615.21	495.3	1381.85	1436.99
Histone H4	TVTAMDVVYALK	256.62	177.99	467.58	336.23
Histone H4	TVTAMDVVYALK	108.37	143.86	145.98	188.52

Histone H4	TVTAMDVVYALK	373.65	424.74	1609.12	1617.69
Histone H4	TVTAMDVVYALK	529.49	453.32	634.35	788.68
Histone H4	TVTAMDVVYALK	93.68	106.25	251.67	218.18
Histone H4	TVTAMDVVYALK	11.8	4.31	115.9	134.68
Histone H4	TVTAMDVVYALK	690.93	779.74	2459.29	2703.68
Histone H4	TVTAMDVVYALK	248.32	41.23	241.9	166.96
Histone H4	TVTAMDVVYALK	2157.29	2183.77	6194.48	6833.86
Histone H4	TVTAMDVVYALK	484.07	685.6	1438.4	2019.66
Histone H4	TVTAMDVVYALK	1550.96	704.17	2247.77	2185.74
Histone H4	TVTAMDVVYALK	204.39	232.12	481.17	345.86
Histone H4	TVTAMDVVYALK	154.5	54.4	146.88	129.05
Histone H4	TVTAMDVVYALK	204.39	232.53	513.85	400.49
Histone H4	TVTAMDVVYALK	975.03	371.55	812.64	390.42
Histone H4	TVTAMDVVYALK	1240.03	572.86	1372.4	1888.16
Histone H4	TVTAMDVVYALK	400.12	256.09	280.14	250.3
Histone H4	TVTAMDVVYALK	535.62	592.44	698.32	1045.56
Histone H4	TVTAMDVVYALK	162.54	111.15	129.11	312.93
Histone H4	TVTAMDVVYALK	486.95	401.07	981.72	920.38
Histone H4	TVTAMDVVYALK	436.43	286.29	833.95	938.02
Histone H4	TVTAMDVVYALK	1329.79	640.39	1639.13	2541.41
Histone H4	TVTAMDVVYALK	1482.98	1569.89	4299.15	3773.46
Histone H4	TVTAMDVVYALK	162.86	79.59	146.63	225.74
Histone H4	TVTAMDVVYALK	1556.37	1127.42	2407.82	2892.47
Histone H4	TVTAMDVVYALK	131.53	90.23	160.82	163.34
Histone H4	TVTAMDVVYALK	153.68	136.18	359.96	580.58
Histone H4	TVTAMDVVYALK	119.98	60.94	375.62	82.97
Histone H4	TVTAMDVVYALK	38.29	46.8	97.85	72.16
Histone H4	TVTAMDVVYALK	282.5	175.08	516.72	383.12
Histone H4	TVTAMDVVYALK	204.22	249.11	971.35	683.6
Histone H4	TVTAMDVVYALK	381.97	134.55	241.98	206.28
Histone H4	TVTAMDVVYALK	551.54	508.94	2217.49	2578.92
Histone H4	TVTAMDVVYALK	25.6	23.82	4.39	51.31
Histone H4	TVTAMDVVYALK	296.22	202.65	1017.22	925.22
Histone H4	TVTAMDVVYALK	1654.33	492.08	1350.35	622.38
Histone H4	TVTAMDVVYALK	761.77	376.76	857.85	784.93
Histone H4	TVTAMDVVYALK	157.88	146.23	591.87	490.89
Histone H4	TVTAMDVVYALK	69.33	65.21	137.02	84.84
Histone H4	TVTAMDVVYALK	383.1	448.43	668.26	578.86
Histone H4	TVTAMDVVYALK	1715.21	1817.81	1716.06	2151.59
Histone H4	TVTAMDVVYALK	235.28	158.78	219.38	134.03
Histone H4	TVTAMDVVYALK	1715.21	1817.81	1716.06	2151.59
Histone H4	TVTAMDVVYALK	410.26	318	638.1	604.29
Histone H4	TVTAMDVVYALK	1961.32	2077.22	6917.62	533.09
Histone H4	TVTAMDVVYALK	255.08	330.81	573.01	328.28
Histone H4	TVTAMDVVYALK	178.99	188.48	233.22	400.1
Histone H4	TVTAMDVVYALK	759.16	1166.61	1741.72	1931.11
Histone H4	TVTAMDVVYALK	333.68	224.95	415.15	259.39
Histone H4	TVTAMDVVYALK	128.4	80.71	377.51	226.39
Histone H4	TVTAMDVVYALK	180.58	31.66	325.3	240.78
Histone H4	TVTAMDVVYALK	133.23	135.16	165.96	182.89
Histone H4	TVTAMDVVYALK	133.23	135.16	165.96	182.89
Histone H4	TVTAMDVVYALK	164.02	71.6	165.42	154
Histone H4	TVTAMDVVYALK	235.28	158.78	219.38	134.03
Histone H4	TVTAMDVVYALK	1715.21	1817.81	1716.06	2151.59
Histone H4	TVTAMDVVYALK	1715.21	1817.81	1716.06	2151.59
Histone H4	TVTAMDVVYALK	547.98	116.07	122.54	202.69
Histone H4	TVTAMDVVYALKR	89.83	60.68	179.48	356.94
Histone H4	TVTAMDVVYALKR	624.52	745.35	2436.13	3688.29
Histone H4	TVTAMDVVYALKR	404.19	279.23	876.04	1091.57
Histone H4	TVTAMDVVYALKR	83.07	90.79	310.67	359.66
Histone H4	TVTAMDVVYALKR	386.21	459.93	1151.91	1534.22
Histone H4	TVTAMDVVYALKR	947.85	1779.54	3495.91	4304.26
Histone H4	TVTAMDVVYALKR	218.7	201.43	723.4	636.55
Histone H4	TVTAMDVVYALKR	863.13	682.36	1911.44	1941.31

Histone H4	TVTAMDVVYALKR	1474.87	1626.14	4205.74	4899.15
Histone H4	TVTAMDVVYALKR	83.2	77.92	148.9	338.76
Histone H4	TVTAMDVVYALKR	1475.03	1823.28	3602.31	4129.81
Histone H4	TVTAMDVVYALKR	462.04	309.15	885.8	1340.32
Histone H4	TVTAMDVVYALKR	673.97	1391.99	1915.61	2212.91
Histone H4	TVTAMDVVYALKR	1185.34	869.45	2007.63	2670.06
Histone H4	TVTAMDVVYALKR	614.8	322.74	930.42	1386.47
Histone H4	TVTAMDVVYALKR	160.19	130.62	398.1	396.66
Histone H4	TVTAMDVVYALKR	1242.01	1334.68	2083.46	2275.06
Histone H4	TVTAMDVVYALKR	848.13	780.89	2092.26	1755.6
Histone H4	TVTAMDVVYALKR	1283.31	1722	4155.07	4223.51
Histone H4	TVTAMDVVYALKR	255.45	187.92	459.49	653.31
Histone H4	TVTAMDVVYALKR	435.94	334.34	1012.32	693.12
Histone H4	TVTAMDVVYALKR	130.99	143.77	173.97	361.92
Histone H4	TVTAMDVVYALKR	133.23	135.16	165.96	182.89
Histone H4	TVTAMDVVYALKR	306.84	323.37	751.24	835.93
Histone H4	TVTAMDVVYALKR	427.17	669.14	1237.87	985.85
Histone H4	TVTAMDVVYALKR	1241.55	954.7	1892.97	2017.17
Histone H4	TVTAMDVVYALKR	179.93	95.47	362.7	432.14
Histone H4	TVTAMDVVYALKR	752.47	550.92	627.77	1033.25
Histone H4	TYTEHAK	101.32	202.2	583.81	502.57
Histone H4	VFLENVIR	254.79	359.2	446.26	625.12
Histone H4	VFLENVIR	492.22	305.69	946.69	965.31
Histone H4	VFLENVIR	1055.58	710.89	2188.63	2463.99
Histone H4	VFLENVIR	776.09	771.2	1505.72	2393.51
Histone H4	VFLENVIR	226.01	223.53	1093.05	1008.2
Histone H4	VFLENVIR	281.97	227.19	493.86	567.98
Histone H4	VFLENVIR	203.36	334.05	602.32	636.18
Histone H4	VFLENVIR	611.23	781.94	2483.28	1912.2
Histone H4	VFLENVIR	1047.15	1331.48	3807.07	4817.28
Histone H4	VFLENVIR	645.39	917.91	2759.52	3568.53
Histone H4	VFLENVIR	460.68	443.44	630.93	680.76
Histone H4	VFLENVIR	570.27	680.13	1712.08	1958.25
Histone H4	VFLENVIR	1548.2	1722.32	4285.7	4569.04
Histone H4	VFLENVIR	1183.1	878.43	4869.15	4932.29
Histone H4	VFLENVIR	11620.7	10729.82	43276.81	49944.15
Histone H4	VFLENVIR	2830.24	2612.6	8472.07	10848.16
Histone H4	VFLENVIR	2516.57	1793.64	6783.11	6975.01
Histone H4	VFLENVIR	3118.66	1883.43	7847.37	7551.8
Histone H4	VFLENVIR	2830.29	1862.29	7715.92	9586.64
Histone H4	VFLENVIR	1601.79	1746.58	4112.63	4982.11
Histone H4	VFLENVIR	4863.89	4032.88	18207.48	20052.28
Histone H4	VFLENVIR	1492	1316.74	3704.1	4272.59
Histone H4	VFLENVIR	1212	789.2	1943.6	2186.64
Histone H4	VFLENVIR	1025.76	784.42	1000.83	1321.66
Histone H4	VFLENVIR	901.32	1057.98	2605.3	2139.58
Histone H4	VFLENVIR	6849.22	7460.79	31253.03	33096.69
Histone H4	VFLENVIR	6719.59	6118.54	25786.26	29564.57
Histone H4	VFLENVIR	7936.19	8261.8	26421.38	28186.75
Histone H4	VFLENVIR	5763.78	4167.41	17031.1	20175
Histone H4	VFLENVIR	2421.85	1577.51	8194.38	9665.17
Histone H4	VFLENVIR	257.29	112.41	517.76	411.09
Histone H4	VFLENVIR	631.27	717.01	1047.27	1842.72
Histone H4	VFLENVIR	1068.16	744.32	1658.96	1940.49
Histone H4	VFLENVIR	702.01	323.7	1201.79	1064.83
Histone H4	VFLENVIR	613.84	736.81	3323.19	3100.85
Histone H4	VFLENVIR	633.04	329.68	1796.87	2190.83
Histone H4	VFLENVIR	225.08	315.32	597.87	713.33
Histone H4	VFLENVIR	229.26	328.54	779.72	1701.61
Histone H4	VFLENVIR	2436.84	2436.8	7174.22	9390.71
Histone H4	VFLENVIR	14133.58	11505.17	50339.68	51907.64
Histone H4	VFLENVIR	10380.69	11005.62	34816.27	37295.36
Histone H4	VFLENVIR	230.79	176.8	620.76	353.04
Histone H4	VFLENVIR	1301.03	1141.87	4011.74	4566.24

Histone H4	VFLENVIR	257.14	127.42	414.12	504.66
Histone H4	VFLENVIR	14459.43	12608.92	47630.13	52160
Histone H4	VFLENVIR	13125.08	14015.14	49287.52	55141.61
Histone H4	VFLENVIR	9074.04	10419.29	36268.84	40568.48
Histone H4	VFLENVIR	13082.49	14499.23	46666.65	51912.41
Histone H4	VFLENVIR	16571.04	17177.67	59256.57	65214.6
Histone H4	VFLENVIR	333.2	272.16	634.8	532.69
Histone H4	VFLENVIR	619.66	268.46	1044.53	1379.54
Histone H4	VFLENVIR	128.12	107.78	581.66	591.5
Histone H4	VFLENVIR	407.15	625.5	544.06	406.68
Histone H4	VFLENVIR	307.04	303.21	471.12	767.86
Histone H4	VFLENVIR	229.05	348.59	1065.46	557.04
Histone H4	VFLENVIR	152.31	271.03	77.11	277.1
Histone H4	VFLENVIR	375.77	1172.61	2477.67	556.57
Histone H4	VFLENVIR	12870.23	11533.38	46021.14	45481.41
Histone H4	VFLENVIR	308.07	201.83	376.38	740.71
Histone H4	VFLENVIR	204.6	211.4	281.78	448.2
Histone H4	VFLENVIR	411.02	242.76	527.12	560.83
Histone H4	VFLENVIR	615.5	785.93	2057.17	1930.94
Histone H4	VFLENVIR	307.02	305.55	355.37	364.53
Histone H4	VFLENVIR	384.86	275.01	304.4	367.29
Histone H4	VFLENVIR	230.21	234.25	334.27	365.76
Histone H4	VFLENVIR	270.12	333.93	363.47	892.78
Histone H4	VFLENVIR	192.05	174.73	226.92	209.2
Histone H4	VFLENVIR	577.99	1087.57	2964.84	4482.25
Histone H4	VFLENVIR	51.39	29.47	56.05	33.54
Histone H4	VFLENVIR	307.76	232.21	151.14	453.82
Histone H4	VFLENVIR	100.6	273.75	131.83	171.05
Histone H4	VFLENVIR	308.06	202.72	332.13	497.17
Histone H4	VFLENVIR	460.94	417.87	617.99	984.54
Histone H4	VFLENVIR	821.14	574.84	2916.15	3104.27
Histone H4	VFLENVIR	53.46	37.19	38.67	23.43
Histone H4	VFLENVIR	434.93	433.62	1104.37	992.26
Histone H4	VFLENVIR	588.5	474.87	1107.82	527.85
Histone H4	VFLENVIR	139.8	230.92	191.92	401.76
Histone H4	VFLENVIR	358.26	348.96	573.72	611.99
Histone H4	VFLENVIR	77.2	31.97	50.25	40.29
Histone H4	VFLENVIR	44.06	8.4	8.25	25.21
Histone H4	VFLENVIR	126.74	244.55	203.99	401.52
Histone H4	VFLENVIR	44716.94	47274.21	175637.5	189045.3
Histone H4	VFLENVIR	178.77	210.25	433.62	545.35
Histone H4	VFLENVIR	255.03	335.43	557.17	380.72
Histone H4	VFLENVIR	178.74	213.13	291.14	136.98
Histone H4	VFLENVIR	686.89	434.97	1603.97	1881.64
Histone H4	VFLENVIR	546.43	587.94	508.94	782.58
Histone H4	VFLENVIR	193.25	56.55	324.18	224.14
Histone H4	VFLENVIR	748.43	524.45	1473.17	2036.71
Histone H4	VFLENVIR	230.11	243.92	281.8	377.24
Histone H4	VFLENVIR	204.66	205.68	564.74	620.14
Histone H4	VFLENVIR	438.45	85.95	524.43	408.63
Histone H4	VFLENVIR	231.26	130.29	366.63	367.36
Three-prime repair exonuclease 1	ACSPAASEITGLSTAVLAAHGR	50.7	97.06	119.27	109.62
Three-prime repair exonuclease 1	VTELCLLAVHR	564.13	435.35	651.76	425.04
Three-prime repair exonuclease 1	ACSPAASEITGLSTAVLAAHGR	89.63	80.76	144.46	159.47
Three-prime repair exonuclease 1	ACSPAASEITGLSTAVLAAHGR	229.72	282.44	505.29	680.26
Three-prime repair exonuclease 1	ACSPAASEITGLSTAVLAAHGR	154.04	100.73	197.42	64.76
Three-prime repair exonuclease 1	ACSPAASEITGLSTAVLAAHGR	268.2	310.9	233.05	567.03
Three-prime repair exonuclease 1	ACSPAASEITGLSTAVLAAHGR	362.01	297.89	1756.22	2454.59
Three-prime repair exonuclease 1	ITALK	1251.83	790.37	1367.36	1774.23
Three-prime repair exonuclease 1	ITALK	5207.99	6738.58	7781.5	8787.21
Three-prime repair exonuclease 1	ITALK	2346.25	3940.06	4738.81	5784.97
Three-prime repair exonuclease 1	ITALK	855.8	661.48	896.53	1208.04
Three-prime repair exonuclease 1	LAFLR	788	657.64	909.87	1063.43
Three-prime repair exonuclease 1	LGLL	1296.99	1434.54	5346.49	5340.23

Three-prime repair exonuclease 1	LGLL	667.11	261.48	2492.45	2908.78
Three-prime repair exonuclease 1	LGLL	3314.76	3998.01	22099.16	20825.3
Three-prime repair exonuclease 1	LGLL	3659.85	3414.84	18140.3	21295.9
Three-prime repair exonuclease 1	LGLL	332.65	326.7	492.06	841.9
Three-prime repair exonuclease 1	LLAI	196.22	188.4	226.96	250.42
Three-prime repair exonuclease 1	LLAI	75.78	172.69	115.6	69.95
Three-prime repair exonuclease 1	VTELCLLAVHR	416.35	248.87	205.4	317.8
Three-prime repair exonuclease 1	VTELCLLAVHR	486.76	419.96	420.36	518.87
Three-prime repair exonuclease 1	VTELCLLAVHR	411.49	197.03	234.46	320.56
Three-prime repair exonuclease 1	VTELCLLAVHR	614.31	477.22	1056.14	455.28
Three-prime repair exonuclease 1	VTELCLLAVHR	589.13	625.16	745.43	905.53
Three-prime repair exonuclease 1	VTELCLLAVHR	435.82	345.67	451.89	485.21
TATA-binding protein-associated factor 2N	QSSYSQQPYNNQGQQQNMESSGSQGGR	25.48	36.12	34.8	49.69
TATA-binding protein-associated factor 2N	SGDWVCPNPSCGNMNFAR	179.5	138.23	163.69	246.98
TATA-binding protein-associated factor 2N	GGFQGRGGDPK	2648.94	1907.22	3817.53	3799.47
TATA-binding protein-associated factor 2N	NSCNQCNEPRPEDSRPSGGDFRGR	153.84	120.11	302.94	476.11
TATA-binding protein-associated factor 2N	AAIDWFDGK	1690.87	2200.75	3872.62	4678.16
TATA-binding protein-associated factor 2N	AAIDWFDGK	1124.95	772.47	1547.74	1504.67
TATA-binding protein-associated factor 2N	AAIDWFDGK	973.1	774.48	1419.34	1356.64
TATA-binding protein-associated factor 2N	AAIDWFDGK	1137.38	927.55	1809.4	2608.44
TATA-binding protein-associated factor 2N	AAIDWFDGK	841.14	727.19	1636.12	1857.17
TATA-binding protein-associated factor 2N	AAIDWFDGK	1734.33	1099.03	2095.94	2309.46
TATA-binding protein-associated factor 2N	EFFK	2347.59	2318.67	2866.59	4261.27
TATA-binding protein-associated factor 2N	GEATVSFDDPPSAK	462.84	230.06	965.8	920.35
TATA-binding protein-associated factor 2N	GEATVSFDDPPSAK	844.55	709.19	1982.01	2143.95
TATA-binding protein-associated factor 2N	GEATVSFDDPPSAK	1302.43	1216.16	1714.64	1859.46
TATA-binding protein-associated factor 2N	GEATVSFDDPPSAK	533.26	506.89	1055.69	379.99
TATA-binding protein-associated factor 2N	GGGYGGDRGGGYGGDRGGGYGGDR	68.25	65.79	218.83	254.5
TATA-binding protein-associated factor 2N	GGGYGGDRGGGYGGDRGGYGGDR	68.25	65.79	218.83	254.5
TATA-binding protein-associated factor 2N	GGGYGGDRGGGYGGDRGGYGGDR	68.25	65.79	218.83	254.5
TATA-binding protein-associated factor 2N	GGGYGGDRGGYGGDRGGGYGGDR	68.25	65.79	218.83	254.5
TATA-binding protein-associated factor 2N	GGGYGGDRGGYGGDRGGGYGGDR	68.25	65.79	218.83	254.5
TATA-binding protein-associated factor 2N	GGGYGGDRGGYGGK	214.94	254.04	847.4	900.72
TATA-binding protein-associated factor 2N	IGII	1296.99	1434.54	5346.49	5340.23
TATA-binding protein-associated factor 2N	IGII	667.11	261.48	2492.45	2908.78
TATA-binding protein-associated factor 2N	IGII	3314.76	3998.01	22099.16	20825.3
TATA-binding protein-associated factor 2N	IGII	3659.85	3414.84	18140.3	21295.9
TATA-binding protein-associated factor 2N	IGII	332.65	326.7	492.06	841.9
TATA-binding protein-associated factor 2N	NIIK	540.63	204.34	676.13	887.3
TATA-binding protein-associated factor 2N	NIIK	359.27	249.9	363.8	1049.81
TATA-binding protein-associated factor 2N	NIIK	202.84	385.47	613.68	578.56
TATA-binding protein-associated factor 2N	QIGIIK	552.18	339.74	1595.82	1830.59
TATA-binding protein-associated factor 2N	QIGIIK	408.77	465.35	1013.97	1253.81
TATA-binding protein-associated factor 2N	QIGIIK	1245.03	1355.72	2361.43	2766.64
TATA-binding protein-associated factor 2N	QIGIIK	1695.09	1570.93	4108.48	5299.81
TATA-binding protein-associated factor 2N	QIGIIK	1965.56	1231.93	3610.65	3949.82
TATA-binding protein-associated factor 2N	QIGIIK	408.27	514.39	1036.59	1219.28
TATA-binding protein-associated factor 2N	QIGIIK	1484.27	1974.38	2636.06	3057.4
TATA-binding protein-associated factor 2N	QIGIIK	1188.09	1236.48	2617.86	3275.82
TATA-binding protein-associated factor 2N	QIGIIK	508.25	743.06	1805	2664.69
TATA-binding protein-associated factor 2N	QIGIIK	1162.86	1175.69	3164.38	3516.78
TATA-binding protein-associated factor 2N	QIGIIK	554.16	675.22	2067.25	2864.93
TATA-binding protein-associated factor 2N	QIGIIK	2166.04	2276.32	4084.24	4616.54
TATA-binding protein-associated factor 2N	QIGIIK	1521.86	920.27	3206.97	2824.07
TATA-binding protein-associated factor 2N	QIGIIK	961.18	1101.74	1338.3	2712.24
TATA-binding protein-associated factor 2N	QIGIIK	1012.87	781.27	3171.9	3076.45
TATA-binding protein-associated factor 2N	QSSYSQQPYNNQGQQQNMESSGSQGGR	25.62	22.18	85.75	98.62
NEDD4 family-interacting protein 2	AAAMAAAAAETSQR	10142.08	9260.43	11862.39	16951.67
NEDD4 family-interacting protein 2	SQRVCASGPSMLNSAR	21.59	0	178.63	67.99
NEDD4 family-interacting protein 2	LGLL	1296.99	1434.54	5346.49	5340.23
NEDD4 family-interacting protein 2	LGLL	667.11	261.48	2492.45	2908.78
NEDD4 family-interacting protein 2	LGLL	3314.76	3998.01	22099.16	20825.3
NEDD4 family-interacting protein 2	LGLL	3659.85	3414.84	18140.3	21295.9
NEDD4 family-interacting protein 2	LGLL	332.65	326.7	492.06	841.9

NEDD4 family-interacting protein 2	LLLF	125.2	77.21	136.77	133.97
NEDD4 family-interacting protein 2	LSLIK	460.8	430.99	342.12	528.29
NEDD4 family-interacting protein 2	LSLIK	967.35	810.9	490.09	723.21
NEDD4 family-interacting protein 2	LSLIK	939.79	1299.55	2110.11	2415.64
NEDD4 family-interacting protein 2	LSLIK	993.34	1754.1	2140.52	2229.4
NEDD4 family-interacting protein 2	LSLIK	886.34	1687.27	1636.55	3061.11
NEDD4 family-interacting protein 2	LSLIK	1495.08	1863.91	2316.71	3550.47
Platelet-activating factor acetylhydrolase IB subunit beta	IIVLGLLPR	213.18	215	125.73	202.83
Platelet-activating factor acetylhydrolase IB subunit beta	SQGDSNPAAIPHAAEDIQGDDRWMSQHNR	412.32	114.31	493.66	409.19
Platelet-activating factor acetylhydrolase IB subunit beta	VNQLLK	610.52	746.25	1325.93	1587.32
Platelet-activating factor acetylhydrolase IB subunit beta	GLLP	206.92	0	95.22	278.73
Platelet-activating factor acetylhydrolase IB subunit beta	IIVLGLLPR	212.61	270.69	245.07	259.14
Platelet-activating factor acetylhydrolase IB subunit beta	IIVLGLLPR	231.62	201.69	184.73	195.67
Platelet-activating factor acetylhydrolase IB subunit beta	IIVLGLLPR	261.2	257.44	137.58	250.8
Platelet-activating factor acetylhydrolase IB subunit beta	IIVLGLLPR	288.67	309.87	209.05	260.66
Platelet-activating factor acetylhydrolase IB subunit beta	IIVLGLLPR	163.13	159.34	136.74	137.31
Platelet-activating factor acetylhydrolase IB subunit beta	IIVLGLLPR	287.64	305.41	220.62	242.73
Platelet-activating factor acetylhydrolase IB subunit beta	LENIK	1091.11	1455.77	1220.86	2041.96
Platelet-activating factor acetylhydrolase IB subunit beta	LENIK	678.07	667.87	700.09	817.51
Platelet-activating factor acetylhydrolase IB subunit beta	LENIK	424.89	574.85	644.34	1207.51
Platelet-activating factor acetylhydrolase IB subunit beta	LENIK	1392.58	1033.36	1969.34	1944.17
Platelet-activating factor acetylhydrolase IB subunit beta	LGLL	1296.99	1434.54	5346.49	5340.23
Platelet-activating factor acetylhydrolase IB subunit beta	LGLL	667.11	261.48	2492.45	2908.78
Platelet-activating factor acetylhydrolase IB subunit beta	LGLL	3314.76	3998.01	22099.16	20825.3
Platelet-activating factor acetylhydrolase IB subunit beta	LGLL	3659.85	3414.84	18140.3	21295.9
Platelet-activating factor acetylhydrolase IB subunit beta	LGLL	332.65	326.7	492.06	841.9
Platelet-activating factor acetylhydrolase IB subunit beta	LLPR	640.2	366.14	1243.74	910.95
Platelet-activating factor acetylhydrolase IB subunit beta	NQLLK	2432.28	4270.55	5946.87	8139.86
Platelet-activating factor acetylhydrolase IB subunit beta	QLLK	2159.8	3638.02	3341.89	4582.17
Platelet-activating factor acetylhydrolase IB subunit beta	QLLK	1331.14	1464.69	1921.29	2240.57
Platelet-activating factor acetylhydrolase IB subunit beta	VNQLLK	80.06	69.44	154.56	134.16
Platelet-activating factor acetylhydrolase IB subunit beta	VSLPK	1556.8	2468.41	1974.36	2483.26
Platelet-activating factor acetylhydrolase IB subunit beta	VSLPK	1437.17	1521	2098.27	3542.22
Nuclease-sensitive element-binding protein 1	EDGNEEDKENQGDETQGQQPPQR	96.02	87.43	482.63	609.36
Nuclease-sensitive element-binding protein 1	EDVFVHQTAIK	332.94	297.58	654.88	631.44
Nuclease-sensitive element-binding protein 1	GAEAANVTGPGGVPVQGSK	2378.55	1919.19	4908.02	6298.8
Nuclease-sensitive element-binding protein 1	RPQYSNPPVQGEVMEGADNQGAGEQGRPVR	25.39	44.33	267.03	449.09
Nuclease-sensitive element-binding protein 1	NGYGFINR	2010.85	3140.89	7788.62	7482.09
Nuclease-sensitive element-binding protein 1	RPQYSNPPVQGEVMEGADNQGAGEQGR	128.03	116.68	141.69	172.89
Nuclease-sensitive element-binding protein 1	VLGTVK	2175.54	1870.04	8402.6	9538.78
Nuclease-sensitive element-binding protein 1	NDTKEDVFVHQTAIK	788.95	670.31	1662.17	1267.71
Nuclease-sensitive element-binding protein 1	WFNVR	2035.79	2485.23	4971.8	6900.76
Nuclease-sensitive element-binding protein 1	GAEAANVTGPGGVPVQGSKYA	76.62	90.03	371.94	288.91
Nuclease-sensitive element-binding protein 1	EDGNEEDKENQGDETQGQQPPQR	12.73	18.84	85.28	51.37
Nuclease-sensitive element-binding protein 1	EDGNEEDKENQGDETQGQQPPQR	16.02	13.45	73.75	123.55
Nuclease-sensitive element-binding protein 1	EDGNEEDKENQGDETQGQQPPQR	46.96	41.13	452.74	562.6
Nuclease-sensitive element-binding protein 1	EDGNEEDKENQGDETQGQQPPQR	25.87	0	53.97	136.87
Nuclease-sensitive element-binding protein 1	EDGNEEDKENQGDETQGQQPPQR	7.39	15.02	80.91	132.97
Nuclease-sensitive element-binding protein 1	EDVFVHQTAIK	201.82	486.55	723.59	496.48
Nuclease-sensitive element-binding protein 1	EDVFVHQTAIK	432.47	783.41	938.01	1002.68
Nuclease-sensitive element-binding protein 1	GAEAANVTGPGGVPVQGSK	1529.65	1746.64	4639.9	5519.73
Nuclease-sensitive element-binding protein 1	GAEAANVTGPGGVPVQGSK	3541.01	2708.69	4411.92	4267.34
Nuclease-sensitive element-binding protein 1	GAEAANVTGPGGVPVQGSK	307.37	270.78	797.74	652.95
Nuclease-sensitive element-binding protein 1	GAEAANVTGPGGVPVQGSK	1258.04	1452.65	1242.57	890.97
Nuclease-sensitive element-binding protein 1	GAEAANVTGPGGVPVQGSK	2199.47	2165.54	3856.74	4167.58
Nuclease-sensitive element-binding protein 1	GAEAANVTGPGGVPVQGSK	987.98	1113.08	2593.81	2608.67
Nuclease-sensitive element-binding protein 1	GAEAANVTGPGGVPVQGSK	4916.71	4132.66	11322.39	14972.61
Nuclease-sensitive element-binding protein 1	NDTKEDVFVHQTAIK	1199.14	996	3994.87	4159.1
Nuclease-sensitive element-binding protein 1	NGYGFINR	4012.75	4612.61	18393.51	21172.73
Nuclease-sensitive element-binding protein 1	NGYGFINR	3176.86	3260.67	7005.02	8186.4
Nuclease-sensitive element-binding protein 1	NGYGFINR	2645.23	4294.92	7843.94	7870.42
Nuclease-sensitive element-binding protein 1	NGYGFINR	3889.74	4531.72	9441.4	9869.68
Nuclease-sensitive element-binding protein 1	NGYGFINR	4061.56	3513.59	8857.19	10039.02
Nuclease-sensitive element-binding protein 1	RPQYSNPPVQGEVMEGADNQGAGEQGR	49.14	251.73	133.33	173.1

Nuclease-sensitive element-binding protein 1	RPQYSNPPVQGEVMEGADNQGAGEQGR	473.78	318.79	365.86	829.19
Nuclease-sensitive element-binding protein 1	RPQYSNPPVQGEVMEGADNQGAGEQGR	20.53	99.33	117.25	174.2
Nuclease-sensitive element-binding protein 1	RPQYSNPPVQGEVMEGADNQGAGEQGR	51.02	65.5	402.49	185.05
Nuclease-sensitive element-binding protein 1	RPQYSNPPVQGEVMEGADNQGAGEQGR	76.58	93.61	194.92	169.26
Nuclease-sensitive element-binding protein 1	RPQYSNPPVQGEVMEGADNQGAGEQGRPVR	89.74	70.47	653.19	438.92
Nuclease-sensitive element-binding protein 1	RPQYSNPPVQGEVMEGADNQGAGEQGRPVR	282.55	169.97	769.15	1492.78
Nuclease-sensitive element-binding protein 1	RPQYSNPPVQGEVMEGADNQGAGEQGRPVR	154.48	56.66	460.76	822.59
Nuclease-sensitive element-binding protein 1	RPQYSNPPVQGEVMEGADNQGAGEQGRPVR	435.55	372.5	402.41	363
Nuclease-sensitive element-binding protein 1	RPQYSNPPVQGEVMEGADNQGAGEQGRPVR	51.3	38.22	474.64	412.38
Nuclease-sensitive element-binding protein 1	RPQYSNPPVQGEVMEGADNQGAGEQGRPVR	25.66	17.67	95.82	201.26
Nuclease-sensitive element-binding protein 1	RPQYSNPPVQGEVMEGADNQGAGEQGRPVR	21.1	43.05	346.11	394.39
Nuclease-sensitive element-binding protein 1	RPQYSNPPVQGEVMEGADNQGAGEQGRPVR	89.68	76.14	372.81	252.7
Nuclease-sensitive element-binding protein 1	RPQYSNPPVQGEVMEGADNQGAGEQGRPVR	38.29	46.81	97.08	96.95
Nuclease-sensitive element-binding protein 1	RPQYSNPPVQGEVMEGADNQGAGEQGRPVR	25.67	17.03	127.16	18.2
Nuclease-sensitive element-binding protein 1	RPQYSNPPVQGEVMEGADNQGAGEQGRPVR	0	49.69	96.98	97.98
Nuclease-sensitive element-binding protein 1	RPQYSNPPVQGEVMEGADNQGAGEQGRPVR	21.1	43.05	346.11	394.39
Nuclease-sensitive element-binding protein 1	VLGTVK	1904.25	2183.47	8998.43	11036.44
Nuclease-sensitive element-binding protein 1	VLGTVK	2813.16	2810.19	10409.63	12165.63
Nuclease-sensitive element-binding protein 1	VLGTVK	2173.26	2094.91	7233.67	7950.31
Nuclease-sensitive element-binding protein 1	VLGTVK	3072.44	3683.1	10746.22	12183.84
Nuclease-sensitive element-binding protein 1	VLGTVK	1474.8	1739.5	3226.15	3037.44
Nuclease-sensitive element-binding protein 1	VLGTVK	2893.78	2505.13	8130.23	9659.56
Nuclease-sensitive element-binding protein 1	VLGTVK	1138.28	1050.68	2789.36	3717.21
Nuclease-sensitive element-binding protein 1	VLGTVK	1456.23	1978.69	2259.74	2606.42
Nuclease-sensitive element-binding protein 1	VLGTVK	720.88	586.78	1788.71	2517.57
Presenilins-associated rhomboid-like protein, mitochondrial	IWHEIR	937.5	568.53	567.94	932.22
Presenilins-associated rhomboid-like protein, mitochondrial	YFTSNPASK	2498.89	3327.91	5897.58	6690.17
Presenilins-associated rhomboid-like protein, mitochondrial	FNFF	212.65	161.31	336.92	469.41
Presenilins-associated rhomboid-like protein, mitochondrial	IVNI	1633.97	1864.79	2774.58	2886.85
Presenilins-associated rhomboid-like protein, mitochondrial	IVNI	920.29	673.13	1254.48	1664.15
Presenilins-associated rhomboid-like protein, mitochondrial	IVNI	687.79	771.45	2083.14	1738.66
Presenilins-associated rhomboid-like protein, mitochondrial	IWHEIR	977.81	415.99	519.31	585.71
Presenilins-associated rhomboid-like protein, mitochondrial	IWHEIR	587.16	606.78	331.77	491.01
Presenilins-associated rhomboid-like protein, mitochondrial	LDSI	357.3	444.42	376.14	235.28
Presenilins-associated rhomboid-like protein, mitochondrial	LDSI	382.05	658.05	515.6	643.4
Presenilins-associated rhomboid-like protein, mitochondrial	LIWK	2403.98	3023.9	3730.95	5019.55
Presenilins-associated rhomboid-like protein, mitochondrial	LLGRR	1293.45	1358.52	2364.85	2041.05
Presenilins-associated rhomboid-like protein, mitochondrial	VLVF	214.94	147.47	214.9	200.87
Presenilins-associated rhomboid-like protein, mitochondrial	VLVF	191.24	148.49	198.22	255.16
Presenilins-associated rhomboid-like protein, mitochondrial	VLVF	263.78	215.47	236.31	234.14
Presenilins-associated rhomboid-like protein, mitochondrial	WWNNLSDGQR	573.03	406.66	761.71	1004.9
Presenilins-associated rhomboid-like protein, mitochondrial	YESLK	1538.24	1535.58	5953.25	5531.07
Presenilins-associated rhomboid-like protein, mitochondrial	YESLK	969.39	1673.58	4669.1	6246.91
Presenilins-associated rhomboid-like protein, mitochondrial	YESLK	4095.43	4529.43	17577.63	21368.12
Presenilins-associated rhomboid-like protein, mitochondrial	YESLK	3082.65	4164.64	11409.07	13751.92
Presenilins-associated rhomboid-like protein, mitochondrial	YESLK	1453.92	1568.73	7707.87	7208.38
Presenilins-associated rhomboid-like protein, mitochondrial	YESLK	1297.12	1527.93	6311.22	6729.36
Presenilins-associated rhomboid-like protein, mitochondrial	YESLK	1182.42	1051.88	4642.53	3828.26
Presenilins-associated rhomboid-like protein, mitochondrial	YESLK	2927.21	2712.91	7250.63	7875.29
Presenilins-associated rhomboid-like protein, mitochondrial	YESLK	1050.57	567.91	1567.04	2012.29
60S ribosomal protein L36	EELSNVLAAMR	114.46	287.36	205.61	188.23
60S ribosomal protein L36	EVCGFAPYER	1019.22	473.6	2144.93	2704.23
60S ribosomal protein L36	KREELSNVLAAMR	2315.28	2319.24	7371.62	7309.64
60S ribosomal protein L36	YPMAVGLNK	2738.18	2877.79	7127.4	10627.19
60S ribosomal protein L36	RAMELLK	2301.97	2144.31	5009.86	6124.33
60S ribosomal protein L36	ALRYPMAVGLNK	918.95	1018.53	1779.5	1047.27
60S ribosomal protein L36	AMELLK	6962.89	5698.45	21473.25	25668.64
60S ribosomal protein L36	AMELLK	1318.32	923.14	2215.35	2853.39
60S ribosomal protein L36	AMELLK	4575.27	3824.04	16617.53	18873.96
60S ribosomal protein L36	AMELLK	1538.52	444.75	1722.42	2308.58

60S ribosomal protein L36	AMELLK	1227.7	1471.35	1651.67	2169.75
60S ribosomal protein L36	AMELLK	1851.25	2845.47	5061.6	4615.99
60S ribosomal protein L36	AMELLK	2044.9	1585.36	3283.01	3666.71
60S ribosomal protein L36	AMELLK	1095.22	943.52	1599.4	1605.94
60S ribosomal protein L36	AMELLK	1861.68	1814.25	4227.74	5236.07
60S ribosomal protein L36	EELSNVLAAMR	70.14	91.68	260.71	271.29
60S ribosomal protein L36	EELSNVLAAMR	146.74	183.33	287.09	265.63
60S ribosomal protein L36	EELSNVLAAMR	177.85	194.78	457.57	496.41
60S ribosomal protein L36	EELSNVLAAMR	255.17	215.61	579.98	694.9
60S ribosomal protein L36	EELSNVLAAMR	498.85	607.87	2090.74	2307.01
60S ribosomal protein L36	EELSNVLAAMR	214.64	176.73	683.72	687.62
60S ribosomal protein L36	EELSNVLAAMR	139.37	166.81	386.28	465.31
60S ribosomal protein L36	EELSNVLAAMR	174.03	146.4	417.96	487.14
60S ribosomal protein L36	EELSNVLAAMR	77.8	79.68	241.19	231.13
60S ribosomal protein L36	EELSNVLAAMR	141.63	49.33	232.38	296.15
60S ribosomal protein L36	EELSNVLAAMR	231.64	92.8	305.16	453.5
60S ribosomal protein L36	EELSNVLAAMR	335.66	135.43	368.42	430.67
60S ribosomal protein L36	EELSNVLAAMR	210.86	231.17	983.25	1215.69
60S ribosomal protein L36	EELSNVLAAMR	222.38	156.79	311.74	351.65
60S ribosomal protein L36	EELSNVLAAMR	255.05	226.67	671.33	740.41
60S ribosomal protein L36	EELSNVLAAMR	238.23	80.71	240.93	365.51
60S ribosomal protein L36	EELSNVLAAMR	244.05	143.83	288.66	359.19
60S ribosomal protein L36	EELSNVLAAMR	271.01	139.33	199.57	258.31
60S ribosomal protein L36	EELSNVLAAMR	128.45	75.56	472.54	610.17
60S ribosomal protein L36	EELSNVLAAMR	127.93	127	376.14	245.99
60S ribosomal protein L36	EELSNVLAAMR	189.05	152.37	599.29	798.27
60S ribosomal protein L36	EELSNVLAAMR	272.4	108.7	326.54	314.05
60S ribosomal protein L36	EELSNVLAAMR	141.86	133.51	163.05	175.47
60S ribosomal protein L36	EELSNVLAAMR	488.18	279.78	753.46	628.37
60S ribosomal protein L36	EELSNVLAAMR	2191.15	1498.13	4083.36	3896.81
60S ribosomal protein L36	EELSNVLAAMR	2092.69	1970.44	4963.26	3542.27
60S ribosomal protein L36	EELSNVLAAMR	593.32	317.19	1283.81	793.9
60S ribosomal protein L36	EELSNVLAAMR	460.47	463.71	799.31	1567.17
60S ribosomal protein L36	EELSNVLAAMR	3135.82	3804.41	5098.12	6833.38
60S ribosomal protein L36	EELSNVLAAMR	307.52	255.52	275.61	551.43
60S ribosomal protein L36	EELSNVLAAMR	49.41	224.86	185.09	222
60S ribosomal protein L36	EELSNVLAAMR	568.75	511.19	660	1092.98
60S ribosomal protein L36	EELSNVLAAMR	1647.19	2261.29	2526.93	3066.14
60S ribosomal protein L36	EELSNVLAAMR	126.53	264.72	483.64	451.25
60S ribosomal protein L36	ELLK	1331.14	1464.69	1921.29	2240.57
60S ribosomal protein L36	ELLK	569.43	869.56	642.15	1040.57
60S ribosomal protein L36	ELLK	631.39	918.11	938.93	1167.85
60S ribosomal protein L36	ELLK	485.14	579.53	777.31	880.2
60S ribosomal protein L36	ELLK	1331.98	1275.66	2386.22	2606.35
60S ribosomal protein L36	ELLK	1118.92	1368.09	1892.76	1206.11
60S ribosomal protein L36	ELLK	1114.11	1205	1892.83	2006.06
60S ribosomal protein L36	ELLK	2703.21	2609.44	4732.29	5170.87
60S ribosomal protein L36	ELLK	2218.14	2554.59	1989.37	2671.55
60S ribosomal protein L36	EVCGFAPYER	1787.75	2097.58	4791.26	7174.91
60S ribosomal protein L36	EVCGFAPYER	3088.36	2749.01	9448.33	8791.11
60S ribosomal protein L36	EVCGFAPYER	4636.12	4620.72	10654.49	12296.17
60S ribosomal protein L36	EVCGFAPYER	7120.9	7533.96	34775.89	39081.61
60S ribosomal protein L36	EVCGFAPYER	2758.01	2620.41	7818.74	8188.49
60S ribosomal protein L36	EVCGFAPYER	1754.44	2090.7	4615.29	5942.11
60S ribosomal protein L36	EVCGFAPYER	855.21	826.29	2267.21	2267.41
60S ribosomal protein L36	EVCGFAPYER	8279.18	8524.19	23726.99	23846.98
60S ribosomal protein L36	EVCGFAPYER	5637.5	6005.61	19912.65	24442.69
60S ribosomal protein L36	EVCGFAPYER	1508.04	2285.03	3298.62	3256.4
60S ribosomal protein L36	EVCGFAPYER	7713.18	8592.68	27306.78	32132.83
60S ribosomal protein L36	EVCGFAPYER	1556.58	2596.27	3309.79	3045.34
60S ribosomal protein L36	EVCGFAPYER	1728.11	2989.36	3661.04	2906.31
60S ribosomal protein L36	EVCGFAPYER	152.37	264.88	381	468.5
60S ribosomal protein L36	EVCGFAPYER	8384.15	8471.92	32151.29	31483.2
60S ribosomal protein L36	EVCGFAPYER	10696.41	8855.6	40441.77	39046.21

60S ribosomal protein L36	EVCGFAPYER	4380.25	4474.2	10116.89	11647.72
60S ribosomal protein L36	EVCGFAPYER	1692.53	2143.55	2546.98	3143.68
60S ribosomal protein L36	EVCGFAPYER	751	1015.93	2576.65	2548.45
60S ribosomal protein L36	EVCGFAPYER	9655.37	8729.61	36819.42	36203.77
60S ribosomal protein L36	EVCGFAPYER	757.32	603.57	1037.13	840.97
60S ribosomal protein L36	EVCGFAPYER	2474.53	2011.26	4735.86	4120.77
60S ribosomal protein L36	EVCGFAPYER	1524.49	1085.41	1783.49	2179.61
60S ribosomal protein L36	EVCGFAPYER	1065.74	877.69	2250.94	2218.68
60S ribosomal protein L36	EVCGFAPYER	565	668.73	752.71	1356.43
60S ribosomal protein L36	EVCGFAPYERR	1787.75	2097.58	4791.26	7174.91
60S ribosomal protein L36	KREELSNVLAAMR	2433.82	2734.85	8452.01	10544.9
60S ribosomal protein L36	KREELSNVLAAMR	1787.93	3144.03	7865.32	9185.53
60S ribosomal protein L36	KREELSNVLAAMR	2672.78	3381.97	8437.06	9516.36
60S ribosomal protein L36	KREELSNVLAAMR	1123.87	1198.53	2602.86	3575.19
60S ribosomal protein L36	KREELSNVLAAMR	786.86	770.08	990.09	1512.58
60S ribosomal protein L36	KREELSNVLAAMR	1531.72	1116.45	2509.01	2837.76
60S ribosomal protein L36	KREELSNVLAAMR	2119.79	2696.5	4265.91	4654.86
60S ribosomal protein L36	KREELSNVLAAMR	354.19	751.06	1442.19	1911.02
60S ribosomal protein L36	KREELSNVLAAMR	1138.74	1005.34	1360.19	1571.13
60S ribosomal protein L36	LAAMRK	1507.23	1514.13	2181.49	2895.17
60S ribosomal protein L36	MELLK	645.9	228.68	363.23	238.44
60S ribosomal protein L36	YPMAVGLNK	203.09	360.73	559.79	918.98
60S ribosomal protein L36	YPMAVGLNK	2338.43	2585.36	7632.89	7984.36
60S ribosomal protein L36	YPMAVGLNK	1444.5	1754.94	3318.06	3338
60S ribosomal protein L36	YPMAVGLNK	4446.6	3664.16	12710.69	13966.16
60S ribosomal protein L36	YPMAVGLNK	1976.04	1686.68	5650.83	5096.96
60S ribosomal protein L36	YPMAVGLNK	3194.73	3729.65	5601.38	7134.7
60S ribosomal protein L36	YPMAVGLNK	1429.82	1609.62	3379.95	2984.53
60S ribosomal protein L36	YPMAVGLNK	713.2	601.17	1264.66	1741.33
60S ribosomal protein L36	YPMAVGLNK	332.85	306.65	1483.15	1170.26
60S ribosomal protein L36	YPMAVGLNK	773.67	584.06	1357.16	1167.77
60S ribosomal protein L36	YPMAVGLNK	205.95	77.91	497.66	372.41
Estradiol 17-beta-dehydrogenase 11	NPSTSLGPTLEPEEVVNR	100.63	270.76	279.65	190.29
Estradiol 17-beta-dehydrogenase 11	LVLWDINK	152.77	226	388.17	312.4
Estradiol 17-beta-dehydrogenase 11	ESFVK	1599.27	2207.85	2846.15	3988.64
Estradiol 17-beta-dehydrogenase 11	FIPK	3105.58	3175	3950.66	5222.85
Estradiol 17-beta-dehydrogenase 11	LAVL	667.11	261.48	2492.45	2908.78
Estradiol 17-beta-dehydrogenase 11	LAVLK	2035.83	1523.99	9543.57	10535.47
Estradiol 17-beta-dehydrogenase 11	LAVLK	1665.34	1744.59	6637.53	7664.58
Estradiol 17-beta-dehydrogenase 11	LAVLK	815.91	1091.67	2953.61	4086.81
Estradiol 17-beta-dehydrogenase 11	LAVLK	561.34	604.43	1712.88	1985.18
Estradiol 17-beta-dehydrogenase 11	LAVLK	1483	1461.14	5370.26	5191.79
Estradiol 17-beta-dehydrogenase 11	LAVLK	540.21	670.58	1922.34	2521.13
Estradiol 17-beta-dehydrogenase 11	LAVLK	648.3	1055.44	2647.25	4185.83
Estradiol 17-beta-dehydrogenase 11	LAVLK	1782.02	1493.33	8054.43	9583.17
Estradiol 17-beta-dehydrogenase 11	LAVLK	871.54	595.46	1179.4	1649.04
Estradiol 17-beta-dehydrogenase 11	LAVLK	1192.9	654.58	2609.21	2263.56
Estradiol 17-beta-dehydrogenase 11	LAVLK	1512.71	653.93	830.6	1005.18
Estradiol 17-beta-dehydrogenase 11	LAVLK	1185.07	1321.63	1980.6	2865.3
Estradiol 17-beta-dehydrogenase 11	LAVLK	3228.25	4566.36	4088.17	6488.61
Estradiol 17-beta-dehydrogenase 11	LAVLK	875.25	973.71	2507.6	2814.37
Estradiol 17-beta-dehydrogenase 11	LAVLK	1012.58	1129.67	1470.03	1562.85
Estradiol 17-beta-dehydrogenase 11	LFIPK	1353.59	1055.15	4970.4	6172.1
Estradiol 17-beta-dehydrogenase 11	LFIPK	1202.21	1330.66	3439.14	2901.53
Estradiol 17-beta-dehydrogenase 11	LFIPK	690.28	737.49	3647.59	4405.51
Estradiol 17-beta-dehydrogenase 11	LTEQK	584.78	948.71	1138.98	1630.23
Estradiol 17-beta-dehydrogenase 11	NPSTSLGPTLEPEEVVNR	282.45	179.73	286.76	341.52
Estradiol 17-beta-dehydrogenase 11	NPSTSLGPTLEPEEVVNR	153.1	193.44	82.78	253.23
Zinc finger protein-like 1	LCNIPLASR	409.21	422.03	602.13	724.21
Zinc finger protein-like 1	LATVNWAR	2320.76	1351.97	3072.66	2071.43
Zinc finger protein-like 1	VNVCEHCLVANHAK	2968.29	3015.86	3868.94	3302.54
Zinc finger protein-like 1	VYDTR	1357.7	1713.52	525.75	533.65
Zinc finger protein-like 1	ARLLR	652.99	911.35	1673.27	1931.99
Zinc finger protein-like 1	ARLLR	1080.13	732.01	1638.98	1877.24

Zinc finger protein-like 1	LATVNWAR	1728.52	1353.27	3317.12	2628.22
Zinc finger protein-like 1	LCNIPLASR	1054.26	841.82	1726.87	2154.96
Zinc finger protein-like 1	LCNIPLASR	622.91	904.7	1261.24	1012.43
Zinc finger protein-like 1	LCNIPLASR	2011.24	1400	2629	2426.83
Zinc finger protein-like 1	LCNIPLASR	460.86	425.88	647.68	979.77
Zinc finger protein-like 1	LCNIPLASR	456.83	823.15	850.56	861.96
Zinc finger protein-like 1	LCNIPLASR	498.79	508.14	694.8	808.48
Zinc finger protein-like 1	LGLL	1296.99	1434.54	5346.49	5340.23
Zinc finger protein-like 1	LGLL	667.11	261.48	2492.45	2908.78
Zinc finger protein-like 1	LGLL	3314.76	3998.01	22099.16	20825.3
Zinc finger protein-like 1	LGLL	3659.85	3414.84	18140.3	21295.9
Zinc finger protein-like 1	LGLL	332.65	326.7	492.06	841.9
Zinc finger protein-like 1	LLAL	196.22	188.4	226.96	250.42
Zinc finger protein-like 1	LLAL	75.78	172.69	115.6	69.95
Zinc finger protein-like 1	LLGF	658.89	647.68	705.21	939.96
Zinc finger protein-like 1	LTLL	114.69	158.48	37.77	113.82
Zinc finger protein-like 1	LTLL	201.09	132.94	186.59	209.92
Zinc finger protein-like 1	LTLL	192.95	85.86	151.74	325.05
Putative transferase CAF17, mitochondrial	LLTQDEGPALVPGGR	793.24	1310.37	1845.59	2098.48
Putative transferase CAF17, mitochondrial	GCYIGQELTAR	315.48	320.15	879.01	418.59
Putative transferase CAF17, mitochondrial	AGYAHFLNVQGR	1181.92	1207.58	2155.28	1769.64
Putative transferase CAF17, mitochondrial	AGAAAILIRDPR	410.48	296.48	424.97	311.38
Putative transferase CAF17, mitochondrial	AILIR	2985.64	3641.8	8639.29	10643.07
Putative transferase CAF17, mitochondrial	AILIR	807.23	1311.26	2069.48	2004.08
Putative transferase CAF17, mitochondrial	GCYIGQELTAR	357.99	376.29	499.28	460.02
Putative transferase CAF17, mitochondrial	ILIR	386.99	489.76	684.01	680.12
Putative transferase CAF17, mitochondrial	ILIR	912.1	950.18	1143.27	1280.65
Putative transferase CAF17, mitochondrial	ILIR	556.67	959.36	927.48	1147.92
Putative transferase CAF17, mitochondrial	ILIR	1415.65	1626.01	1823.04	1846.6
Putative transferase CAF17, mitochondrial	ILIR	279.39	481.67	627.51	552.92
Putative transferase CAF17, mitochondrial	ILIR	487.03	393.55	449.11	438.94
Putative transferase CAF17, mitochondrial	LALYR	2078.86	1421.47	2542.53	3101.04
Putative transferase CAF17, mitochondrial	LGLL	1296.99	1434.54	5346.49	5340.23
Putative transferase CAF17, mitochondrial	LGLL	667.11	261.48	2492.45	2908.78
Putative transferase CAF17, mitochondrial	LGLL	3314.76	3998.01	22099.16	20825.3
Putative transferase CAF17, mitochondrial	LGLL	3659.85	3414.84	18140.3	21295.9
Putative transferase CAF17, mitochondrial	LGLL	332.65	326.7	492.06	841.9
Putative transferase CAF17, mitochondrial	LLRVR	5138.54	4769.86	11009.6	13833.13
Putative transferase CAF17, mitochondrial	LLTQDEGPALVPGGR	2129.58	2049.29	2948.53	3945.9
Putative transferase CAF17, mitochondrial	LLTQDEGPALVPGGR	1893.53	1966.14	2235.31	2760.61
Putative transferase CAF17, mitochondrial	LLTQDEGPALVPGGR	2035.5	1769.72	2389.16	2902.2
Serine protease 56	ELLAWDPPQELQADAAR	153.64	139.66	187.9	298.33
Serine protease 56	LAPALALPAPALR	150.89	92.13	208.83	203.35
Serine protease 56	VPLLSTDTCR	731.55	809.16	802.13	1154.8
Serine protease 56	AGLGGR	510.98	580.38	533.95	755.53
Serine protease 56	AAQR	206.2	53.15	444.83	501
Serine protease 56	AGLGGR	511.45	534.11	640.39	1026.37
Serine protease 56	AGLGGR	680.96	382.53	761.99	753.45
Serine protease 56	ELLAWDPPQELQADAAR	128.29	90.91	139.23	236.51
Serine protease 56	ELLAWDPPQELQADAAR	68.29	61.97	88.47	63.72
Serine protease 56	LAVL	667.11	261.48	2492.45	2908.78
Serine protease 56	LGLL	1296.99	1434.54	5346.49	5340.23
Serine protease 56	LGLL	667.11	261.48	2492.45	2908.78
Serine protease 56	LGLL	3314.76	3998.01	22099.16	20825.3
Serine protease 56	LGLL	3659.85	3414.84	18140.3	21295.9
Serine protease 56	LGLL	332.65	326.7	492.06	841.9
Serine protease 56	LGLLR	772.6	1009.43	1169.95	1102.84
Serine protease 56	LGLLR	484.95	598.57	527.29	833.2
Serine protease 56	LGLLR	889.11	1094.25	961.9	1189.95
Serine protease 56	LGLLR	1108.53	1543.55	1229.93	1769.51
Serine protease 56	LGLLR	980.63	1519.85	1222.12	845.75
Serine protease 56	LGLLR	881.63	875.5	1172.56	719.34
Serine protease 56	LGLLR	988.01	896.86	1055.42	1203.53
Serine protease 56	LGLLR	252.97	539.23	483.12	768.99

Serine protease 56	LGLLR	610.41	650.31	1125.48	924.24
Serine protease 56	LGLLR	584.6	647.49	986.93	1677.42
Serine protease 56	LGLLR	1447.92	1203.54	1220.32	1386.83
Serine protease 56	LGLLR	801.22	734.4	1425.97	1094.17
Serine protease 56	LLGLLR	483.61	518.69	653.99	1107.19
Serine protease 56	LLGLLR	256.04	236.08	148.95	522.7
Serine protease 56	LLGLLR	615.77	227.28	542.91	587.06
Serine protease 56	LLGLLR	653.77	834.29	1705.62	3119
Serine protease 56	LLGLLR	382.83	475.12	625.77	933.52
Serine protease 56	LLGLLR	1067.95	871.8	2002.55	1568.66
Serine protease 56	VPLLSTDTCR	917.96	477.34	1214.3	455.52
Serine protease 56	VPLLSTDTCR	328.07	247.41	601.49	796.62
Serine protease 56	VPLLSTDTCR	581.86	705.01	864.41	1347.42
40S ribosomal protein S29	GHQQLYWSHPR	248.74	319.03	700.09	625.75
40S ribosomal protein S29	YGLNMCR	2285.36	2190.09	4454.89	4537.8
40S ribosomal protein S29	DIGFIKLD	11087.82	11355.44	32419.86	41222.38
40S ribosomal protein S29	DIGFIK	2220.52	3382.97	4193.79	3923.4
40S ribosomal protein S29	QYAKDIGFIKLD	1007.21	1128.21	2253.41	2398.64
40S ribosomal protein S29	FRQYAK	673.48	1014.93	1261.42	2580.31
40S ribosomal protein S29	DIGFIK	2284.69	1617.45	4912.44	6015.3
40S ribosomal protein S29	DIGFIK	3793.57	3607.42	8602.48	10475.49
40S ribosomal protein S29	DIGFIK	3787.58	3454.83	2183.08	2736.91
40S ribosomal protein S29	DIGFIK	1660.79	1980.87	2678.95	2073.93
40S ribosomal protein S29	DIGFIK	3867.68	4370.6	6535.15	7775.89
40S ribosomal protein S29	DIGFIKLD	13050.58	14885.72	46813.57	55837.01
40S ribosomal protein S29	DIGFIKLD	26102.08	28190.88	71172.73	90047.1
40S ribosomal protein S29	DIGFIKLD	3935.97	2304.71	7044.35	7977.99
40S ribosomal protein S29	DIGFIKLD	811.79	1179.41	3203.13	3393.27
40S ribosomal protein S29	DIGFIKLD	2141.39	2051.88	5755.72	6349.43
40S ribosomal protein S29	DIGFIKLD	8598.66	9512.96	17420.31	19058.96
40S ribosomal protein S29	GHQQLYWSHPR	229.75	279.81	369.51	384.23
40S ribosomal protein S29	GHQQLYWSHPR	1984.18	2265.45	2682.26	3230.04
40S ribosomal protein S29	GHQQLYWSHPR	1461.63	1339.09	2390.54	4011.44
40S ribosomal protein S29	GHQQLYWSHPR	1935.59	1427.12	2575.47	2827.64
40S ribosomal protein S29	GHQQLYWSHPR	341.71	388.57	589.52	719.92
40S ribosomal protein S29	YGLNMCR	1212.4	1600.36	4528.28	4927.27
40S ribosomal protein S29	YGLNMCR	969.85	883.34	2216.58	2073.24
40S ribosomal protein S29	YGLNMCR	1135.67	776.78	1295.1	2144.42
40S ribosomal protein S29	YGLNMCR	4962.66	4912.04	12865.5	14438.77
40S ribosomal protein S29	YGLNMCR	3999.81	3869.48	10414.79	12588.92
40S ribosomal protein S29	YGLNMCR	860.09	1301.74	1974.37	2183.16
Prostaglandin E synthase 3	KGESGQSWPR	1003.92	1347.13	3191.3	3344.77
Prostaglandin E synthase 3	SILCCLR	723.13	897.05	1644.88	1861.72
Prostaglandin E synthase 3	DVNVNFEK	1039.94	1298.61	2055.59	1862.69
Prostaglandin E synthase 3	SKLTFSCLGGSDNFK	153.11	192.47	130.58	378.63
Prostaglandin E synthase 3	KGESGQSWPR	577.09	857.15	2717.48	2962.58
Prostaglandin E synthase 3	KGESGQSWPR	429.05	589.56	965.06	1046.95
Prostaglandin E synthase 3	KGESGQSWPR	576.01	857.7	2641.29	2709.29
Prostaglandin E synthase 3	KGESGQSWPR	666.92	705.9	1199.99	1749.05
Prostaglandin E synthase 3	KGESGQSWPR	924.09	616.96	2157.42	2673.3
Prostaglandin E synthase 3	SILCCLR	1051.58	680.83	2520.15	1896.49
Prostaglandin E synthase 3	SILCCLR	2176.64	3143.82	7674.32	7789.98
Prostaglandin E synthase 3	SILCCLR	2906.25	3612.9	8338.28	10294.41
Prostaglandin E synthase 3	SILCCLR	1944.39	1622.22	4008.95	4271.17
Prostaglandin E synthase 3	SILCCLR	1332.41	1552.18	3014.5	4081.83
Prostaglandin E synthase 3	SILCCLR	5750.32	4965.42	15795.9	18690.34
Prostaglandin E synthase 3	SILCCLR	806.47	535.59	1453.99	1861.51
Prostaglandin E synthase 3	SILCCLR	1518.18	1496.84	3475.36	3571.97
Prostaglandin E synthase 3	SILCCLR	604.08	424.99	964.75	1101.78
Prostaglandin E synthase 3	SILCCLR	1385.54	1515.99	3811.76	5485.4
Prostaglandin E synthase 3	SILCCLR	1113.35	854.51	1468.08	2792.15
Prostaglandin E synthase 3	SILCCLR	1090.4	1525.95	2592.38	4656.44
Prostaglandin E synthase 3	SILCCLR	3898.41	3355.93	8531.04	7568.51
Prostaglandin E synthase 3	SILCCLR	612.31	781.86	1100.27	1519.65

Prostaglandin E synthase 3	SILCCLR	1006.78	1277.31	2006.98	2742.34
Prostaglandin E synthase 3	SILCCLR	670.69	1077.68	1677.91	2813.75
Prostaglandin E synthase 3	SILCCLR	408.84	458.25	566.83	890.52
Prostaglandin E synthase 3	SILCCLR	1316.78	1181.06	3557.83	4363.56
Prostaglandin E synthase 3	SILCCLR	1128.29	867.82	1978.32	1838.67
Phosphatidylserine decarboxylase proenzyme, mitochondrial	GSYNDFSFVTHTNR	243.81	167.13	413.55	299.83
Phosphatidylserine decarboxylase proenzyme, mitochondrial	NLSEFFR	653.09	901.83	813.78	724.08
Phosphatidylserine decarboxylase proenzyme, mitochondrial	GVTYSLESFLGPR	413.7	297.53	1052.72	785.14
Phosphatidylserine decarboxylase proenzyme, mitochondrial	ILNFGQVK	984.73	902.6	1368.73	1163.47
Phosphatidylserine decarboxylase proenzyme, mitochondrial	DFNFQLK	804.64	503.27	1357.23	1470.14
Phosphatidylserine decarboxylase proenzyme, mitochondrial	EITAL	696.39	346.62	567.28	483.28
Phosphatidylserine decarboxylase proenzyme, mitochondrial	EITAL	846.76	809.64	1207.1	642.43
Phosphatidylserine decarboxylase proenzyme, mitochondrial	EITAL	840.21	712.18	1371.35	1274.4
Phosphatidylserine decarboxylase proenzyme, mitochondrial	FLLR	993.65	1617.09	1683.05	1522.96
Phosphatidylserine decarboxylase proenzyme, mitochondrial	FLLR	1306.18	1590.35	2020.16	2264.02
Phosphatidylserine decarboxylase proenzyme, mitochondrial	FLLR	2250.35	2670.49	3744.21	4025.15
Phosphatidylserine decarboxylase proenzyme, mitochondrial	FLLR	7295.87	8226.42	10784.35	13712.01
Phosphatidylserine decarboxylase proenzyme, mitochondrial	FLLR	1721.03	1135.92	2128.23	2658.95
Phosphatidylserine decarboxylase proenzyme, mitochondrial	GSYNDFSFVTHTNR	360.04	173.92	290.01	241.52
Phosphatidylserine decarboxylase proenzyme, mitochondrial	GSYNDFSFVTHTNR	360.04	173.92	290.01	241.52
Phosphatidylserine decarboxylase proenzyme, mitochondrial	GSYNDFSFVTHTNR	348.98	202.7	183.38	299.51
Phosphatidylserine decarboxylase proenzyme, mitochondrial	GSYNDFSFVTHTNR	436.11	317.98	544.13	619
Phosphatidylserine decarboxylase proenzyme, mitochondrial	GSYNDFSFVTHTNR	306.23	383.13	350.45	364.65
Phosphatidylserine decarboxylase proenzyme, mitochondrial	ILNF	423.9	353.62	687.59	880.8
Phosphatidylserine decarboxylase proenzyme, mitochondrial	LGLL	1296.99	1434.54	5346.49	5340.23
Phosphatidylserine decarboxylase proenzyme, mitochondrial	LGLL	667.11	261.48	2492.45	2908.78
Phosphatidylserine decarboxylase proenzyme, mitochondrial	LGLL	3314.76	3998.01	22099.16	20825.3
Phosphatidylserine decarboxylase proenzyme, mitochondrial	LGLL	3659.85	3414.84	18140.3	21295.9
Phosphatidylserine decarboxylase proenzyme, mitochondrial	LGLL	332.65	326.7	492.06	841.9
Phosphatidylserine decarboxylase proenzyme, mitochondrial	LSEF	256.82	158.75	141.81	69.66
Phosphatidylserine decarboxylase proenzyme, mitochondrial	MFLLR	1319.09	1379.31	2837.98	3218.58
40S ribosomal protein S26	NIVEAAAVR	1406.43	1686.12	3564.35	4573.86
40S ribosomal protein S26	DISEASVFDAYVLPK	282.25	198.94	613.84	529.54
40S ribosomal protein S26	LHYCVSCAIHSK	1316.59	2158.1	3098.78	3433.8
40S ribosomal protein S26	DISEASVFDAYVLPK	358.27	348.04	619.27	707.29
40S ribosomal protein S26	DISEASVFDAYVLPK	386.98	65.03	471.29	438.48
40S ribosomal protein S26	DISEASVFDAYVLPK	641.63	438.37	218.57	319.91
40S ribosomal protein S26	DISEASVFDAYVLPK	307.01	306.09	328.71	394.04
40S ribosomal protein S26	FVIR	976.09	1117.79	4000.14	3822.14
40S ribosomal protein S26	FVIR	1115.99	1019.13	2835.9	3064.67
40S ribosomal protein S26	FVIR	1107.62	888.16	2537	2709.99
40S ribosomal protein S26	KFVIR	2145.23	1673.2	2346.46	3611.83
40S ribosomal protein S26	NIVEAAAVR	2241.64	2253.7	6628.23	6751.44
40S ribosomal protein S26	NIVEAAAVR	12780.12	12031.88	44723.49	47193.19
40S ribosomal protein S26	NIVEAAAVR	1235.82	988.44	1574.6	2106.91
40S ribosomal protein S26	NIVEAAAVR	1449.35	1381.6	3311.11	3179.89
40S ribosomal protein S26	NIVEAAAVR	1691.46	972.73	2649.84	2798.51
40S ribosomal protein S26	NIVEAAAVR	937.18	1025.19	1489.23	1929.84
40S ribosomal protein S26	NIVEAAAVR	1292.48	1348.35	2977.96	2596.68
40S ribosomal protein S26	NIVEAAAVR	1132.59	1719.44	2600.12	2676.27
40S ribosomal protein S26	NIVEAAAVR	6736.26	6599.67	11711.49	14637.64
Plasminogen activator inhibitor 1 RNA-binding protein	EMTLDEWK	1452.26	1200.97	9150.69	11934.84
Plasminogen activator inhibitor 1 RNA-binding protein	PGHLQEGFGCVVTNR	1774.88	1241.05	2049.47	2129.75
Plasminogen activator inhibitor 1 RNA-binding protein	RPDQQLQGEGK	53	82.66	344.44	398.78
Plasminogen activator inhibitor 1 RNA-binding protein	SAAQAAAQTNSNAAGK	238.03	207.02	535.88	786.67
Plasminogen activator inhibitor 1 RNA-binding protein	SSASAPDVDDPEAFPALA	25.87	0	50.18	48.97
Plasminogen activator inhibitor 1 RNA-binding protein	SSFSHYSGLK	406.32	707.08	1777.59	1915.03
Plasminogen activator inhibitor 1 RNA-binding protein	EETQPPVALK	1452.65	1162.59	3335.52	4561.14
Plasminogen activator inhibitor 1 RNA-binding protein	GFVLHK	676.94	459.93	724.61	1186.35
Plasminogen activator inhibitor 1 RNA-binding protein	SEEAHAEDSVMDHHFR	487.13	383.02	969.52	936.04

Plasminogen activator inhibitor 1 RNA-binding protein	AKVEFNIR	383.65	393.93	809.11	1184.08
Plasminogen activator inhibitor 1 RNA-binding protein	SSASAPDVDDPEAFPAL	0	25.35	23.43	24.52
Plasminogen activator inhibitor 1 RNA-binding protein	VEFNIR	3936.18	4517.93	9155.57	8664.04
Plasminogen activator inhibitor 1 RNA-binding protein	SKSEEAHAEDSVMDHHFR	287.97	378.59	163.21	176.2
Plasminogen activator inhibitor 1 RNA-binding protein	EAGGGGVGGPGAKSAAQAAAQTNSNAAGK	595.87	172.05	794.64	725.26
Plasminogen activator inhibitor 1 RNA-binding protein	AAGK	63.27	132.37	188.19	240.83
Plasminogen activator inhibitor 1 RNA-binding protein	AAGK	301.5	425.68	922.42	964.99
Plasminogen activator inhibitor 1 RNA-binding protein	AKVEFNIR	1401.52	2065.14	6375.2	6247
Plasminogen activator inhibitor 1 RNA-binding protein	AKVEFNIR	1389.73	1527.41	3604.02	2995.45
Plasminogen activator inhibitor 1 RNA-binding protein	AKVEFNIR	2275.43	2426.25	3713.14	5345.04
Plasminogen activator inhibitor 1 RNA-binding protein	AKVEFNIR	3870.6	3231.14	6082.73	7437.14
Plasminogen activator inhibitor 1 RNA-binding protein	EMTLDEWK	1399.62	1294.96	9800.66	9921.68
Plasminogen activator inhibitor 1 RNA-binding protein	EMTLDEWK	853.45	1000.31	2335.35	2419.38
Plasminogen activator inhibitor 1 RNA-binding protein	EMTLDEWK	458.33	675.49	862.34	816.48
Plasminogen activator inhibitor 1 RNA-binding protein	EMTLDEWK	561.34	711.1	2606.29	3499.64
Plasminogen activator inhibitor 1 RNA-binding protein	EMTLDEWK	724.31	461.38	3032.34	2384.75
Plasminogen activator inhibitor 1 RNA-binding protein	EMTLDEWK	581.21	556.26	884.65	848.92
Plasminogen activator inhibitor 1 RNA-binding protein	EMTLDEWK	900.96	880.83	3390.82	5117.41
Plasminogen activator inhibitor 1 RNA-binding protein	EMTLDEWK	574.54	683.67	1468.05	1141.57
Plasminogen activator inhibitor 1 RNA-binding protein	EMTLDEWK	203.12	357.72	708.93	717.64
Plasminogen activator inhibitor 1 RNA-binding protein	EMTLDEWK	256.88	152.32	459.7	614.98
Plasminogen activator inhibitor 1 RNA-binding protein	EVLK	796.48	777.65	1165.19	1070.14
Plasminogen activator inhibitor 1 RNA-binding protein	EVLK	1332.13	1047.96	1681.8	2087.14
Plasminogen activator inhibitor 1 RNA-binding protein	FEVLK	3498.29	2355.17	2641.23	2554.76
Plasminogen activator inhibitor 1 RNA-binding protein	FEVLK	2048.16	2007.96	1514.82	2979.76
Plasminogen activator inhibitor 1 RNA-binding protein	FEVLK	2999	1790.05	2214.13	2602.7
Plasminogen activator inhibitor 1 RNA-binding protein	FEVLK	386.68	95.14	259.6	556.91
Plasminogen activator inhibitor 1 RNA-binding protein	GFVLHK	1198.35	967.54	5192.52	6979.42
Plasminogen activator inhibitor 1 RNA-binding protein	GFVLHK	2131.97	1493.67	3457.2	4836.67
Plasminogen activator inhibitor 1 RNA-binding protein	GFVLHK	1295.22	971.1	1895.77	3058.31
Plasminogen activator inhibitor 1 RNA-binding protein	GFVLHK	318.36	674.84	1833.4	1529.57
Plasminogen activator inhibitor 1 RNA-binding protein	GGVGGPGAK	2153.8	2315.32	3157.32	3761.39
Plasminogen activator inhibitor 1 RNA-binding protein	PGHLQEGFGCVVTNR	357.73	401.87	830.54	389.87
Plasminogen activator inhibitor 1 RNA-binding protein	PGHLQEGFGCVVTNR	1239.06	1094.5	1529.02	1827.69
Plasminogen activator inhibitor 1 RNA-binding protein	PGHLQEGFGCVVTNR	729.25	1142.84	1591.04	2279.86
Plasminogen activator inhibitor 1 RNA-binding protein	PGHLQEGFGCVVTNR	657.52	996.23	2085.26	2868.7
Plasminogen activator inhibitor 1 RNA-binding protein	PGHLQEGFGCVVTNR	1269.31	1297.4	2657.47	1799.99
Plasminogen activator inhibitor 1 RNA-binding protein	PGHLQEGFGCVVTNR	357.76	399.15	645.85	1066.09
Plasminogen activator inhibitor 1 RNA-binding protein	PGHLQEGFGCVVTNR	152.35	267.35	258.81	242.97
Plasminogen activator inhibitor 1 RNA-binding protein	PGHLQEGFGCVVTNR	813.49	1544.03	1915.16	1685
Plasminogen activator inhibitor 1 RNA-binding protein	PGHLQEGFGCVVTNR	385.65	196.84	338.57	289.29
Plasminogen activator inhibitor 1 RNA-binding protein	PGHLQEGFGCVVTNR	696.65	852.83	950.38	1736.96
Plasminogen activator inhibitor 1 RNA-binding protein	PGHLQEGFGCVVTNR	3438.74	2919.76	6526.53	5743.21
Plasminogen activator inhibitor 1 RNA-binding protein	PGHLQEGFGCVVTNR	939.71	562.79	1481.7	1658.9
Plasminogen activator inhibitor 1 RNA-binding protein	RPDQQLQGEGK	118.49	208.02	1137.12	1047.95
Plasminogen activator inhibitor 1 RNA-binding protein	RPDQQLQGEGK	126.16	194.84	803.38	783.68
Plasminogen activator inhibitor 1 RNA-binding protein	RPDQQLQGEGK	117.47	202.28	839.44	961.32
Plasminogen activator inhibitor 1 RNA-binding protein	RPDQQLQGEGK	197.34	183.94	1081.71	1006.19
Plasminogen activator inhibitor 1 RNA-binding protein	RPDQQLQGEGK	218.39	231.66	984.63	493.5
Plasminogen activator inhibitor 1 RNA-binding protein	RPDQQLQGEGK	901.42	729.02	4941.52	5506.33
Plasminogen activator inhibitor 1 RNA-binding protein	RPDQQLQGEGK	170.23	203.27	1182.61	1111.54
Plasminogen activator inhibitor 1 RNA-binding protein	RPDQQLQGEGK	277.53	346.28	842.44	724.26
Plasminogen activator inhibitor 1 RNA-binding protein	SAAQAAAQTNSNAAGK	209.16	292.74	656.41	566.57
Plasminogen activator inhibitor 1 RNA-binding protein	SAAQAAAQTNSNAAGK	68.48	43.07	171.44	200.47
Plasminogen activator inhibitor 1 RNA-binding protein	SAAQAAAQTNSNAAGK	182.36	175.14	1359.24	1578.44
Plasminogen activator inhibitor 1 RNA-binding protein	SAAQAAAQTNSNAAGK	380.78	358.14	1845.43	1895.96
Plasminogen activator inhibitor 1 RNA-binding protein	SAAQAAAQTNSNAAGK	185.32	201.24	908.3	1023.93
Plasminogen activator inhibitor 1 RNA-binding protein	SAAQAAAQTNSNAAGK	301.26	235.67	854.67	797.76
Plasminogen activator inhibitor 1 RNA-binding protein	SAAQAAAQTNSNAAGK	484.99	807.1	3988.2	4659.67
Plasminogen activator inhibitor 1 RNA-binding protein	SAAQAAAQTNSNAAGK	67.45	144.42	268.12	200.23
Plasminogen activator inhibitor 1 RNA-binding protein	SAAQAAAQTNSNAAGK	67.45	144.42	268.12	200.23
Plasminogen activator inhibitor 1 RNA-binding protein	SAAQAAAQTNSNAAGK	51.33	35.24	196.44	243.26
Plasminogen activator inhibitor 1 RNA-binding protein	SAAQAAAQTNSNAAGK	114.34	192.55	268.48	508.95
Plasminogen activator inhibitor 1 RNA-binding protein	SAAQAAAQTNSNAAGK	1246.29	911.41	6091.31	7696.12

Plasminogen activator inhibitor 1 RNA-binding protein	SAAQAAAQTNSNAAGK	1013.8	1221.68	4786.37	5369.67
Plasminogen activator inhibitor 1 RNA-binding protein	SAAQAAAQTNSNAAGK	20.82	70.96	243.44	321.34
Plasminogen activator inhibitor 1 RNA-binding protein	SAAQAAAQTNSNAAGK	37.44	131.23	392.4	453.08
Plasminogen activator inhibitor 1 RNA-binding protein	SAAQAAAQTNSNAAGK	877.6	1167.49	3551.57	4496.62
Plasminogen activator inhibitor 1 RNA-binding protein	SAAQAAAQTNSNAAGK	42.14	91.74	148	200.97
Plasminogen activator inhibitor 1 RNA-binding protein	SAAQAAAQTNSNAAGK	42.14	91.74	148	200.97
Plasminogen activator inhibitor 1 RNA-binding protein	SAAQAAAQTNSNAAGK	252.09	307.05	1121.07	1252.77
Plasminogen activator inhibitor 1 RNA-binding protein	SAAQAAAQTNSNAAGK	67.9	100.36	318.42	652.62
Plasminogen activator inhibitor 1 RNA-binding protein	SAAQAAAQTNSNAAGK	127.84	135.46	489.88	515.62
Plasminogen activator inhibitor 1 RNA-binding protein	SAAQAAAQTNSNAAGK	127.84	135.46	489.88	515.62
Plasminogen activator inhibitor 1 RNA-binding protein	SAAQAAAQTNSNAAGK	101.72	162.79	510.65	730.58
Plasminogen activator inhibitor 1 RNA-binding protein	SAAQAAAQTNSNAAGK	327.34	851.08	1965.02	2312.21
Plasminogen activator inhibitor 1 RNA-binding protein	SAAQAAAQTNSNAAGK	230.49	206.41	433.79	827.96
Plasminogen activator inhibitor 1 RNA-binding protein	SAAQAAAQTNSNAAGK	826.75	552.61	1548.51	2214.78
Plasminogen activator inhibitor 1 RNA-binding protein	SAAQAAAQTNSNAAGK	101.39	195.64	163.34	145.82
Plasminogen activator inhibitor 1 RNA-binding protein	SAAQAAAQTNSNAAGK	1498.8	1389.97	4524.58	4648.62
Plasminogen activator inhibitor 1 RNA-binding protein	SAAQAAAQTNSNAAGK	1498.8	1389.97	4524.58	4648.62
Plasminogen activator inhibitor 1 RNA-binding protein	SAAQAAAQTNSNAAGK	252.09	307.05	1121.07	1252.77
Plasminogen activator inhibitor 1 RNA-binding protein	SAAQAAAQTNSNAAGK	230.49	206.41	433.79	827.96
Plasminogen activator inhibitor 1 RNA-binding protein	SAAQAAAQTNSNAAGK	205.12	160.26	256.61	570.36
Plasminogen activator inhibitor 1 RNA-binding protein	SAAQAAAQTNSNAAGK	205.12	160.26	256.61	570.36
Plasminogen activator inhibitor 1 RNA-binding protein	SAAQAAAQTNSNAAGK	230.49	206.41	433.79	827.96
Plasminogen activator inhibitor 1 RNA-binding protein	SAAQAAAQTNSNAAGK	67.9	100.36	318.42	652.62
Plasminogen activator inhibitor 1 RNA-binding protein	SAAQAAAQTNSNAAGK	205.12	160.26	256.61	570.36
Plasminogen activator inhibitor 1 RNA-binding protein	SAAQAAAQTNSNAAGK	205.12	160.26	256.61	570.36
Plasminogen activator inhibitor 1 RNA-binding protein	SAAQAAAQTNSNAAGK	205.12	160.26	256.61	570.36
Plasminogen activator inhibitor 1 RNA-binding protein	SAAQAAAQTNSNAAGK	205.12	160.26	256.61	570.36
Plasminogen activator inhibitor 1 RNA-binding protein	SAAQAAAQTNSNAAGK	20.82	70.96	243.44	321.34
Plasminogen activator inhibitor 1 RNA-binding protein	SAAQAAAQTNSNAAGK	205.12	160.26	256.61	570.36
Plasminogen activator inhibitor 1 RNA-binding protein	SAAQAAAQTNSNAAGK	205.12	160.26	256.61	570.36
Plasminogen activator inhibitor 1 RNA-binding protein	SAAQAAAQTNSNAAGK	1498.8	1389.97	4524.58	4648.62
Plasminogen activator inhibitor 1 RNA-binding protein	SAAQAAAQTNSNAAGK	177.27	358.89	373.43	519.28
Plasminogen activator inhibitor 1 RNA-binding protein	SAAQAAAQTNSNAAGKQLR	68.1	80.28	87.7	50.48
Plasminogen activator inhibitor 1 RNA-binding protein	SEEAHAEDSVMDHHFR	565.16	227.03	740.69	905.3
Plasminogen activator inhibitor 1 RNA-binding protein	SEEAHAEDSVMDHHFR	487.13	383.02	969.52	936.04
Plasminogen activator inhibitor 1 RNA-binding protein	SEEAHAEDSVMDHHFR	2452.89	2872.51	4657.35	7613.77
Plasminogen activator inhibitor 1 RNA-binding protein	SEEAHAEDSVMDHHFR	6892.5	6588.82	11569.3	12600.35
Plasminogen activator inhibitor 1 RNA-binding protein	SEEAHAEDSVMDHHFR	565.16	227.03	740.69	905.3
Plasminogen activator inhibitor 1 RNA-binding protein	SEEAHAEDSVMDHHFR	2062.64	1641.47	3261.41	3977.26
Plasminogen activator inhibitor 1 RNA-binding protein	SKSEEAHAEDSVMDHHFR	450.42	393.23	492.75	633.3
Plasminogen activator inhibitor 1 RNA-binding protein	SSASAPDVDDPEAFPALA	51.01	66.61	347.7	289.01
Plasminogen activator inhibitor 1 RNA-binding protein	SSASAPDVDDPEAFPALA	229.87	268.24	1207.36	862.44
Plasminogen activator inhibitor 1 RNA-binding protein	SSASAPDVDDPEAFPALA	178.58	229.1	778.15	831.5
Plasminogen activator inhibitor 1 RNA-binding protein	SSASAPDVDDPEAFPALA	77.33	19.7	18.89	179.52
Plasminogen activator inhibitor 1 RNA-binding protein	SSASAPDVDDPEAFPALA	76.85	67.18	224.74	91.23
Plasminogen activator inhibitor 1 RNA-binding protein	SSASAPDVDDPEAFPALA	0	0	50.13	48.95
Plasminogen activator inhibitor 1 RNA-binding protein	SSASAPDVDDPEAFPALA	25.34	49.82	0	25.92
Plasminogen activator inhibitor 1 RNA-binding protein	SSASAPDVDDPEAFPALA	0	10.01	37.31	23.81
Plasminogen activator inhibitor 1 RNA-binding protein	SSASAPDVDDPEAFPALA	0	10.01	36.91	36.73
Plasminogen activator inhibitor 1 RNA-binding protein	SSASAPDVDDPEAFPALA	1231.26	801.13	2133.7	2231.31
Plasminogen activator inhibitor 1 RNA-binding protein	SSASAPDVDDPEAFPALA	949.11	910.99	1456.66	1579.07
Plasminogen activator inhibitor 1 RNA-binding protein	SSASAPDVDDPEAFPALA	763.18	875.82	1697.12	1528.9
Plasminogen activator inhibitor 1 RNA-binding protein	SSASAPDVDDPEAFPALA	1443.83	1501.72	2123.37	2575.74
Plasminogen activator inhibitor 1 RNA-binding protein	SSASAPDVDDPEAFPALA	21.55	0	0	0
Plasminogen activator inhibitor 1 RNA-binding protein	SSFSHYSGLK	518.11	513.61	1364	1570.66
Plasminogen activator inhibitor 1 RNA-binding protein	VEFNIR	10218.06	8243.4	25139.02	30840.79
Plasminogen activator inhibitor 1 RNA-binding protein	YIQK	1033.73	1380.04	1879.71	2790.94
Methyl-CpG-binding domain protein 1	SDTYYQSPTGDR	461.14	398.15	475.9	930.99
Methyl-CpG-binding domain protein 1	FAMK	3007.55	3711.17	9382.64	10454.26
Methyl-CpG-binding domain protein 1	FAMK	1285.38	1836.7	3420.35	5620.44
Methyl-CpG-binding domain protein 1	FAMK	3003.9	3327.02	8186.22	10407.54
Methyl-CpG-binding domain protein 1	FAMK	2988.79	3969.03	5738.11	9092.73
Methyl-CpG-binding domain protein 1	FAMK	1494.31	1833.07	3100.39	4688.25
Methyl-CpG-binding domain protein 1	FAMK	3849.62	4453.29	8255.63	11007.05

Methyl-CpG-binding domain protein 1	FAMK	1137.72	1425.27	2824.93	3427.63
Methyl-CpG-binding domain protein 1	FAMK	4737.05	3585.63	8722.35	13012.68
Protein YIF1B	RIPVSQPGMADPHQLFDDTSSAQSR	561.78	561.27	973.93	873.13
Protein YIF1B	ILADAAAEGVPVR	598.68	532.14	737.5	1327.31
Protein YIF1B	MHPAGLAAAAAGTPR	385.16	244.52	534.92	479.98
Protein YIF1B	ADPHQLFDDTSSAQSR	51.74	0	74.52	99.26
Protein YIF1B	ADPHQLFDDTSSAQSR	102.15	120.59	43.07	19.02
Protein YIF1B	ILADAAAEGVPVR	510.07	563.4	951.88	1280.58
Protein YIF1B	ILADAAAEGVPVR	1069.64	704.56	1067.04	1817.04
Protein YIF1B	LFGK	9062.24	8818.6	14750.69	20824.39
Protein YIF1B	LFGK	3401.04	3453.03	6827.18	7258.8
Protein YIF1B	LFGK	5285.29	7186.07	10414.68	15434.6
Protein YIF1B	LFGK	1512.12	2840.11	3378.09	4968.55
Protein YIF1B	LFGK	1118.23	1329.95	1654.16	2890.87
Protein YIF1B	LFGK	2473.12	2469.67	3872.74	3759.92
Protein YIF1B	LFGK	6430.53	8720.22	14102.57	17149.91
Protein YIF1B	LGLL	1296.99	1434.54	5346.49	5340.23
Protein YIF1B	LGLL	667.11	261.48	2492.45	2908.78
Protein YIF1B	LGLL	3314.76	3998.01	22099.16	20825.3
Protein YIF1B	LGLL	3659.85	3414.84	18140.3	21295.9
Protein YIF1B	LGLL	332.65	326.7	492.06	841.9
Protein YIF1B	LRLK	3814.3	3262.06	4759.66	4438.43
Protein YIF1B	MHPAGLAAAAAGTPR	77.09	43.48	119.89	197.87
Cofilin-1	ASGVAVSDGVIK	409.96	348.25	419.01	719.95
Cofilin-1	HELQANCYEEVKDR	1036.62	669.12	5015.88	3869.9
Cofilin-1	KSSTPEEVK	143.75	159.6	461.01	636.08
Cofilin-1	YALYDATYETK	76.35	116.54	338.1	499.02
Cofilin-1	MLPDKDCR	2437.85	1805.34	9631.12	10574.73
Cofilin-1	AVLFCLSEDKK	332.77	314.68	1086.15	1249.33
Cofilin-1	LGGSAVISLEGKPL	1098.29	534.07	1364.21	975.19
Cofilin-1	NIILEEGK	1215.19	898.92	2195.69	1715.47
Cofilin-1	SSTPEEVKK	257.54	299.93	708.54	1096.01
Cofilin-1	MIYASSK	489.91	746.97	1891.6	1463.07
Cofilin-1	CTLAEK	1441.53	1516.06	2752.15	3945.22
Cofilin-1	VFNDMK	1718.58	1803.63	2883.56	4230.03
Cofilin-1	HELQANCYEEVK	253.33	503.76	747.22	738.06
Cofilin-1	LTGIKHELQANCYEEVKDR	2366.39	1312.47	2998.04	3725.28
Cofilin-1	ASGVAVSDGVIK	912.86	768.68	1608.18	2267.64
Cofilin-1	ASGVAVSDGVIK	332.81	310.66	1285.05	1212.98
Cofilin-1	ASGVAVSDGVIK	76.65	86.37	552.8	565.17
Cofilin-1	ASGVAVSDGVIK	384.18	341.86	830.02	957.56
Cofilin-1	ASGVAVSDGVIK	3239.26	2308.09	9271.03	10069.33
Cofilin-1	ASGVAVSDGVIK	354.51	720.2	1158.86	1153.3
Cofilin-1	ASGVAVSDGVIK	153.69	134.7	433.06	546.78
Cofilin-1	ASGVAVSDGVIK	333.1	282.23	403.06	540.36
Cofilin-1	ASGVAVSDGVIK	437.41	189.26	523.96	456.78
Cofilin-1	ASGVAVSDGVIK	5337.72	4772.07	15968.2	18228.72
Cofilin-1	ASGVAVSDGVIK	411.8	166.32	476.05	386.47
Cofilin-1	ASGVAVSDGVIK	320.25	275	754.74	609
Cofilin-1	ASGVAVSDGVIK	436.37	291.98	552.71	852.95
Cofilin-1	ASGVAVSDGVIK	281.29	294.55	993.92	1326.09
Cofilin-1	ASGVAVSDGVIK	152.4	262.78	484.64	559.87
Cofilin-1	ASGVAVSDGVIK	656.09	818.26	1585.69	2286.15
Cofilin-1	ASGVAVSDGVIK	229.97	258.61	406.58	692.96
Cofilin-1	ASGVAVSDGVIK	281.95	228.89	409.92	429.4
Cofilin-1	ASGVAVSDGVIK	522.32	735.85	993.04	1189.02
Cofilin-1	ASGVAVSDGVIK	308.88	122.11	487.26	861.89
Cofilin-1	ASGVAVSDGVIK	13061.64	9962.8	38226.1	43702.24
Cofilin-1	ASGVAVSDGVIK	253.6	476.4	1036.1	830.35
Cofilin-1	ASGVAVSDGVIK	799.01	633.54	944.48	1390.46
Cofilin-1	ASGVAVSDGVIK	792.23	1091.27	2421.91	1965.34
Cofilin-1	ASGVAVSDGVIK	204.91	181.16	500.36	102.34
Cofilin-1	ASGVAVSDGVIK	986.88	1008.59	4096.44	4520.31
Cofilin-1	ASGVAVSDGVIK	333.15	276.54	418.46	646.18

Cofilin-1	ASGVAVSDGVIK	462.78	235.83	680.7	321.74
Cofilin-1	ASGVAVSDGVIK	125.58	358.48	370.5	622.54
Cofilin-1	ASGVAVSDGVIK	868.72	980.94	1854.96	2364.06
Cofilin-1	ASGVAVSDGVIK	505.84	237.2	454.8	612.97
Cofilin-1	ASGVAVSDGVIK	153.72	132.39	546.99	868.34
Cofilin-1	ASGVAVSDGVIK	308.07	202.07	364.24	289.12
Cofilin-1	ASGVAVSDGVIK	279.45	476.08	903.98	935.16
Cofilin-1	ASGVAVSDGVIK	152.93	209.97	542.2	331.33
Cofilin-1	ASGVAVSDGVIK	178.49	237.94	341.16	136.42
Cofilin-1	AVLF	438.29	421.77	774.37	921.2
Cofilin-1	AVLFCLSEDKK	1063.84	1065.38	3558.4	4606.62
Cofilin-1	AVLFCLSEDKK	357.85	389.52	1122.01	1749.1
Cofilin-1	CTLAEK	900.14	642.42	2049.52	2566.23
Cofilin-1	CTLAEK	2117.17	1998.61	3426.81	3367.17
Cofilin-1	CTLAEK	2651.97	2353.09	4196.9	5130.43
Cofilin-1	CTLAEK	615.9	852.71	1465.36	1369.25
Cofilin-1	DGVIK	510.3	541.23	664.54	1100.17
Cofilin-1	DGVIK	691.04	450.48	608.72	1129.16
Cofilin-1	HELQANCYEEVKDR	191.16	156.59	436.17	465.79
Cofilin-1	HELQANCYEEVKDR	1911.16	2245.05	3054.31	4037.46
Cofilin-1	HELQANCYEEVKDR	1926.21	2141.42	7966.3	8401.51
Cofilin-1	HELQANCYEEVKDR	574.85	333.52	1181.72	1720.24
Cofilin-1	HELQANCYEEVKDR	140.76	135.59	330.04	401.29
Cofilin-1	HELQANCYEEVKDR	217.46	217.27	582.6	501.59
Cofilin-1	HELQANCYEEVKDR	283.54	72.45	483.49	845.12
Cofilin-1	HELQANCYEEVKDR	1406.44	1472.69	3856.32	5023.27
Cofilin-1	HELQANCYEEVKDR	2425.7	2261.21	7284.35	7893.59
Cofilin-1	HELQANCYEEVKDR	611.35	1089.56	2371.45	2515.07
Cofilin-1	HELQANCYEEVKDR	332.68	642.27	1051.3	861.5
Cofilin-1	HELQANCYEEVKDR	824.64	760.92	1198.03	973.95
Cofilin-1	HELQANCYEEVKDR	231.53	104.46	367.22	400.76
Cofilin-1	KSSTPEEVK	287.88	388.31	1119.15	1427.08
Cofilin-1	KSSTPEEVK	343.12	355.75	1197.34	1391.54
Cofilin-1	KSSTPEEVK	80.63	118.8	317.11	443.45
Cofilin-1	LAEKL	4468.26	3651.83	5688.17	8123.33
Cofilin-1	LEGK	2875.13	2113.23	3383.03	3619.79
Cofilin-1	LGGSAVISLEGKPL	375.74	324.96	419.96	313.75
Cofilin-1	LGGSAVISLEGKPL	785	315.58	660.65	399.01
Cofilin-1	LTGIK	1005.95	1146.6	1933.42	2676.77
Cofilin-1	LTGIK	5316.1	4568.04	8427.77	8182.08
Cofilin-1	LTGIK	5139.77	4223.03	8907.06	12769.64
Cofilin-1	LTGIK	4238.08	4477.84	6415.28	8007.41
Cofilin-1	LTGIK	2581.48	2401.89	5339.46	5211.2
Cofilin-1	LTGIK	3339.67	3771.5	5021.52	7282.55
Cofilin-1	LTGIK	10055.94	10004.87	19181.96	23484.29
Cofilin-1	LTGIK	4297.9	5483.04	7534.69	10990.4
Cofilin-1	MIYASSK	1083.65	809.94	1440.91	1554.28
Cofilin-1	MIYASSK	1970.13	3121.74	4416.81	6706.2
Cofilin-1	MIYASSK	12769.82	13475.62	31587.11	38304.7
Cofilin-1	MIYASSK	1717.83	2835.37	3656.9	4686.6
Cofilin-1	MIYASSK	1009.54	1536.56	2896.02	2947.78
Cofilin-1	MIYASSK	2878.08	1821.94	5069.04	6039.82
Cofilin-1	MIYASSK	2613.95	2066.32	2937.49	4909.18
Cofilin-1	MIYASSK	988.23	875.45	1741.55	1746.75
Cofilin-1	MIYASSK	505.09	416.85	719.44	1001.8
Cofilin-1	MIYASSK	906.01	1019.65	1558.58	1854.05
Cofilin-1	MIYASSK	1203.67	1186.18	1698.06	2369
Cofilin-1	MIYASSK	2285.84	3099.96	5507.7	6484.2
Cofilin-1	MIYASSK	1384.64	2349.36	3069.03	3550.08
Cofilin-1	MIYASSK	624.08	469.55	1293.71	890.85
Cofilin-1	MIYASSK	1989.09	1567.23	2469.66	3403.32
Cofilin-1	MIYASSK	586.7	546.18	778.35	1365.06
Cofilin-1	MIYASSK	2555.14	3089.9	6295.1	8316.57
Cofilin-1	MIYASSK	114.4	187.37	311.82	365.42

Cofilin-1	MLPDKDCR	834.02	1430.02	5032.87	6096.36
Cofilin-1	MLPDKDCR	2441.5	2189.62	8267.57	11494.94
Cofilin-1	MLPDKDCR	284.18	434.53	1452.35	1710.5
Cofilin-1	MLPDKDCR	1497.24	1330.84	2189.52	2835.99
Cofilin-1	MLPDKDCR	978.28	1007.71	2416.34	3762.82
Cofilin-1	MLPDKDCR	378.54	367.28	444.19	594.59
Cofilin-1	MLPDKDCR	410.32	312.59	905.11	947.07
Cofilin-1	MLPDKDCR	383.17	441.31	1020.44	702.2
Cofilin-1	MLPDKDCR	526.76	829.97	3076.07	3418.64
Cofilin-1	MLPDKDCR	682.07	591.9	2049.77	2717.81
Cofilin-1	NIILEEGK	1192.39	1130.88	2932.25	1627.87
Cofilin-1	SKMIYASSK	63.24	135.23	100	239.56
Cofilin-1	SKMIYASSK	905.72	836.07	2077.02	2744.76
Cofilin-1	SSTPEEVKK	567.54	736.77	1686.37	1873.03
Cofilin-1	VFNDMK	2431.36	2871.56	7336.64	5704.85
Cofilin-1	VFNDMK	2718.07	3800.53	8948.26	11505.85
Cofilin-1	VFNDMK	6842.93	8613.82	18546.37	21670.38
Cofilin-1	VFNDMK	1502.45	1773.85	1798.03	2212.04
Cofilin-1	YALYDATYETK	75.88	162.85	602.05	1369.41
Cofilin-1	YALYDATYETK	1380.67	1252.71	4938.68	4107.86
Cofilin-1	YALYDATYETK	3219.85	2417.17	10169.27	11420.24
Cofilin-1	YALYDATYETK	2456.74	2066.43	5475.96	3792.33
Cofilin-1	YALYDATYETK	2075.91	1713.45	4811.79	5681.65
Cofilin-1	YALYDATYETK	5287.08	4988.5	22256	29181.92
Cofilin-1	YALYDATYETK	3372.64	4874.97	18452.42	21150.57
Cofilin-1	YALYDATYETK	51.51	17.51	221.71	297.81
Cofilin-1	YALYDATYETK	386.64	843.32	1067.68	675.5
Cofilin-1	YALYDATYETK	280.29	392.8	1243.64	1063.58
Cofilin-1	YALYDATYETK	901.3	528.03	1267.92	1700.39
Cofilin-1	YALYDATYETK	1513.95	1807.94	2250.28	1779.79
Cofilin-1	YALYDATYETK	609.44	958.98	1710.76	1663.35
Cofilin-1	YALYDATYETK	280.29	392.8	1243.64	1063.58
Cofilin-1	YALYDATYETK	280.29	392.8	1243.64	1063.58
Cofilin-1	YALYDATYETK	1161.33	901.35	836.54	1063.67
60S ribosomal protein L23	ECADLWPR	1943.86	2099.55	4760.01	5813.83
60S ribosomal protein L23	GSAITGPVAK	4595.47	4275.66	10691.75	12786.79
60S ribosomal protein L23	ISLGLPVGAVINCADNTGAK	333.56	236.81	1105.74	800.71
60S ribosomal protein L23	LPAAGVGDMVMATVK	287.35	439.74	1076.86	1226.54
60S ribosomal protein L23	NLYIISVK	4310.44	4137.4	16288.94	17809.01
60S ribosomal protein L23	VHPAVVIR	768.41	891.4	2609.43	2326.69
60S ribosomal protein L23	IASNAGSIA	1604.1	1305.82	2237.71	3520.27
60S ribosomal protein L23	GSAITGPVAKECADLWPR	448.68	564.56	587.61	618.32
60S ribosomal protein L23	GKPELR	325.62	276.48	874.2	1058.51
60S ribosomal protein L23	ISLGLPVGAVIN	89.09	134.55	38.97	126.24
60S ribosomal protein L23	ECADLWPR	4837.89	4899.24	12839.32	16144.97
60S ribosomal protein L23	ECADLWPR	1724.14	1786.23	2926.42	3522.97
60S ribosomal protein L23	ECADLWPR	7160.48	8517.35	25399.5	26283.57
60S ribosomal protein L23	ECADLWPR	4136.71	3004.14	10751.87	13678.76
60S ribosomal protein L23	ECADLWPR	7114.68	7510.59	28188.83	30508.13
60S ribosomal protein L23	ECADLWPR	3092.55	3079.49	9414.84	9242
60S ribosomal protein L23	ECADLWPR	3462.35	3139.98	8312.05	10009.92
60S ribosomal protein L23	ECADLWPR	3223.95	4034.25	9522.02	10828.21
60S ribosomal protein L23	ECADLWPR	5087.1	4958.54	12715.69	14443.43
60S ribosomal protein L23	ECADLWPR	5895.88	5052.56	15685.96	14677.11
60S ribosomal protein L23	ECADLWPR	4579.14	3654.88	12471	12424.52
60S ribosomal protein L23	ECADLWPR	3988.82	4423.99	10418.22	14147.6
60S ribosomal protein L23	ECADLWPR	3236.2	4419.13	9746.62	11685.26
60S ribosomal protein L23	ECADLWPR	3566.8	3883.96	10061.94	11580.45
60S ribosomal protein L23	ECADLWPR	3834.59	3810.1	10553.46	11474.74
60S ribosomal protein L23	ECADLWPR	1090.66	2031.98	2878.27	2882.16
60S ribosomal protein L23	ECADLWPR	633.95	877.95	2331.47	2929.13
60S ribosomal protein L23	ECADLWPR	835.11	896.92	2942.69	2229.5
60S ribosomal protein L23	ECADLWPR	849.11	1003.35	1084.03	1745.93
60S ribosomal protein L23	ECADLWPR	3014.38	4207.24	7903.06	9296.91

60S ribosomal protein L23	ECADLWPR	1699.01	1822.51	4468.55	4657.06
60S ribosomal protein L23	ECADLWPR	5831.78	8620.09	5079.98	6344.65
60S ribosomal protein L23	ECADLWPR	4238.94	3967.28	11827.9	12326.3
60S ribosomal protein L23	ECADLWPR	5203.32	5710.61	14713.85	14572.22
60S ribosomal protein L23	ECADLWPR	1001.07	1096.83	2126.32	2548.31
60S ribosomal protein L23	ECADLWPR	548.97	443.94	1506.14	1522.47
60S ribosomal protein L23	ECADLWPR	2265.73	3491.33	6114.65	7604.15
60S ribosomal protein L23	ECADLWPR	1670.25	1153.13	2314.37	2864.09
60S ribosomal protein L23	ECADLWPR	1170	1214.91	1463.87	2317.54
60S ribosomal protein L23	ECADLWPR	918.25	555.55	1067.59	1518.58
60S ribosomal protein L23	ECADLWPR	432.64	660.17	1395.58	1256.02
60S ribosomal protein L23	ECADLWPR	674.02	961.13	623.04	1294.96
60S ribosomal protein L23	ECADLWPR	1996.52	1790.66	3783.34	4625.66
60S ribosomal protein L23	ECADLWPR	2073.43	2276.83	3547.43	3734.41
60S ribosomal protein L23	ECADLWPR	1789.06	1117.33	1894.4	1833.01
60S ribosomal protein L23	ECADLWPR	436.99	230.24	1051.42	646.22
60S ribosomal protein L23	GKPELR	216.8	282.74	431.75	701.15
60S ribosomal protein L23	GSAITGPVAK	485.71	523.06	1015.18	1263.32
60S ribosomal protein L23	GSAITGPVAK	5504.86	5812.67	10528.35	12505.47
60S ribosomal protein L23	GSAITGPVAK	255.93	246.33	919.03	674.56
60S ribosomal protein L23	GSAITGPVAK	513.48	333.38	775.49	1274.14
60S ribosomal protein L23	GSAITGPVAK	5716.29	5668.45	13908.95	15881.44
60S ribosomal protein L23	GSAITGPVAK	7748.96	6971.97	16072.62	18294.97
60S ribosomal protein L23	GSAITGPVAK	253.73	463.77	702.95	1185.33
60S ribosomal protein L23	GSAITGPVAK	4216.23	5147.4	8533.15	10535.08
60S ribosomal protein L23	GSAITGPVAK	755.22	598.84	1385.32	835.87
60S ribosomal protein L23	GSAITGPVAK	308.08	200.19	457.2	592.68
60S ribosomal protein L23	GSAITGPVAKECADLWPR	75.6	190.02	163.26	162
60S ribosomal protein L23	GSAITGPVAKECADLWPR	562.75	359.41	584.01	554.4
60S ribosomal protein L23	IASNAGSIA	953.83	763.37	1667.19	2094.07
60S ribosomal protein L23	IASNAGSIA	1587.72	1540.88	3010.1	3233.28
60S ribosomal protein L23	IASNAGSIA	1955.89	1868.16	3821.79	3621.94
60S ribosomal protein L23	IASNAGSIA	1669.01	1594.98	3280.34	2984.66
60S ribosomal protein L23	IASNAGSIA	2023.65	1663.19	4032.14	4293.86
60S ribosomal protein L23	IASNAGSIA	1965.93	2153.6	4717.99	4672.55
60S ribosomal protein L23	IASNAGSIA	1412.76	1379.95	1665.5	2947.8
60S ribosomal protein L23	IASNAGSIA	679.81	921.64	1810.54	2303.24
60S ribosomal protein L23	IASNAGSIA	1148.38	1542.13	1845.51	2222.81
60S ribosomal protein L23	IASNAGSIA	2006.01	1171.83	2127.01	3061.98
60S ribosomal protein L23	IASNAGSIA	9556.42	8824.95	23742.57	25824.08
60S ribosomal protein L23	IASNAGSIA	1929.36	2149.4	3198.26	3374.27
60S ribosomal protein L23	IASNAGSIA	1476.51	1251.39	2205.93	1642.52
60S ribosomal protein L23	IASNAGSIA	1797.53	1131.44	2282.44	2146.73
60S ribosomal protein L23	IISVK	2044.28	1859.27	2501.39	4084.44
60S ribosomal protein L23	ISLGLPVGAVINCADNTGAK	189.2	243.88	646.63	461.62
60S ribosomal protein L23	ISLGLPVGAVINCADNTGAK	281.47	276.41	614.04	752.83
60S ribosomal protein L23	ISLGLPVGAVINCADNTGAK	230.11	244.68	456.94	443.6
60S ribosomal protein L23	ISLGLPVGAVINCADNTGAK	270.12	227.73	510.72	721.26
60S ribosomal protein L23	ISLGLPVGAVINCADNTGAK	987.74	1030.86	3736.51	3699.59
60S ribosomal protein L23	ISLGLPVGAVINCADNTGAK	76.61	90.99	324.46	188.06
60S ribosomal protein L23	ISLGLPVGAVINCADNTGAK	536.09	758.45	2218.21	3454.26
60S ribosomal protein L23	ISLGLPVGAVINCADNTGAK	218.87	78.23	434.14	375.72
60S ribosomal protein L23	ISLGLPVGAVINCADNTGAK	140.85	127.13	216.03	247.97
60S ribosomal protein L23	ISLGLPVGAVINCADNTGAK	218.47	117.91	175.28	187.63
60S ribosomal protein L23	ISLGLPVGAVINCADNTGAK	536.09	758.45	2218.21	3454.26
60S ribosomal protein L23	ISLGLPVGAVINCADNTGAK	12.86	6.23	176.37	119.23
60S ribosomal protein L23	LPAAGVGDMVMATVK	355.52	300.56	690.75	964.24
60S ribosomal protein L23	LPAAGVGDMVMATVK	958.81	590.99	1612.8	1949.67
60S ribosomal protein L23	LPAAGVGDMVMATVK	688.95	337.52	1044.8	1118.04
60S ribosomal protein L23	LPAAGVGDMVMATVK	746.37	90.06	469.15	425.52
60S ribosomal protein L23	LPAAGVGDMVMATVK	205.63	109.43	216.28	271.1
60S ribosomal protein L23	LPAAGVGDMVMATVK	281.42	281.71	352.26	520.49
60S ribosomal protein L23	LPAAGVGDMVMATVK	255.62	276.9	684.74	298.79
60S ribosomal protein L23	LPAAGVGDMVMATVK	438.2	110.92	566.64	627.82

60S ribosomal protein L23	LPAAGVGDMVMATVK	453.41	310.1	498.32	445.61
60S ribosomal protein L23	LPAAGVGDMVMATVK	206.44	29.77	323.8	291.37
60S ribosomal protein L23	LPAAGVGDMVMATVK	308.04	204.21	258.5	371.88
60S ribosomal protein L23	LPAAGVGDMVMATVK	308.04	204.21	258.5	371.88
60S ribosomal protein L23	LPAAGVGDMVMATVK	1005.34	1099.61	1654	2215.03
60S ribosomal protein L23	LPAAGVGDMVMATVK	386.16	146.12	292.49	215.65
60S ribosomal protein L23	NLYIISVK	256.4	199.77	667.5	602.95
60S ribosomal protein L23	NLYIISVK	4974.55	5758.81	21572.13	24229.53
60S ribosomal protein L23	NLYIISVK	6386.3	7239.02	22430.64	26427.46
60S ribosomal protein L23	NLYIISVK	256.82	158.97	130.86	431.3
60S ribosomal protein L23	NLYIISVK	794.24	1637.37	1676.79	2090.87
60S ribosomal protein L23	NLYIISVK	734.25	649.6	1344.73	1372.56
60S ribosomal protein L23	NLYIISVK	588.31	387	1678.37	1518.87
60S ribosomal protein L23	NLYIISVK	634.1	1076.08	2582.85	2866.18
60S ribosomal protein L23	NLYIISVK	1806.77	1069.5	3344.62	3830.96
60S ribosomal protein L23	NLYIISVK	514.01	280.27	847.65	1237.09
60S ribosomal protein L23	NLYIISVK	535.13	746.88	1836.78	1958.06
60S ribosomal protein L23	NLYIISVK	451.79	364.21	752.91	933.46
60S ribosomal protein L23	NLYIISVK	614.47	355.52	1863.44	2031.49
60S ribosomal protein L23	NLYIISVK	1587.99	1301.11	4125.28	5283.21
60S ribosomal protein L23	NLYIISVK	2996.22	3128.92	8758.87	10743.05
60S ribosomal protein L23	NLYIISVK	2911.55	3834.37	10235.78	13411.16
60S ribosomal protein L23	NLYIISVK	1755.04	1925.2	1842.72	2988.45
60S ribosomal protein L23	NLYIISVK	1223.24	1380.62	3762.85	4135.12
60S ribosomal protein L23	NLYIISVK	1529.55	1649.81	4324.2	4369.58
60S ribosomal protein L23	NLYIISVK	329.83	605.18	1088.34	1314.13
60S ribosomal protein L23	NLYIISVK	332.99	292.31	915.29	516.77
60S ribosomal protein L23	NLYIISVK	687.74	776.38	722.63	908.56
60S ribosomal protein L23	NLYIISVK	231.67	90.69	409.44	170.73
60S ribosomal protein L23	NLYIISVK	231.56	100.78	549.04	433.13
60S ribosomal protein L23	NLYIISVK	308.31	177.84	285.21	396.32
60S ribosomal protein L23	NLYIISVK	192.06	173.69	278.25	254.33
60S ribosomal protein L23	NLYIISVK	318.8	98.85	324.6	288.51
60S ribosomal protein L23	NLYIISVK	255.55	283.71	347.9	662.58
60S ribosomal protein L23	NLYIISVK	918.91	1234.85	1504.01	1362.08
60S ribosomal protein L23	NLYIISVK	787.49	1027.54	1868.46	2077.32
60S ribosomal protein L23	NLYIISVK	701.57	473.63	1020.4	997.74
60S ribosomal protein L23	VAKECADLWPR	111.4	58.12	120.67	139.41
60S ribosomal protein L23	VHPAVVIR	858.99	772.34	1411.86	1229.08
60S ribosomal protein L23	VHPAVVIR	230.52	204.14	546.29	592.29
60S ribosomal protein L23	VHPAVVIR	230.22	233.76	358.52	418.35
Protein JTB	TTPECGPTGYVEK	510.83	594.52	952.01	1210.7
Protein JTB	FTLK	1809.78	1729.15	3574.53	4790.2
Protein JTB	FTLK	1767.73	2160.22	2989.2	3960.75
Protein JTB	FTLK	547.31	607.39	873.36	1048.26
Protein JTB	FTLK	1627.32	2734.63	3759.35	4574.7
Protein JTB	FTLK	1911.43	2218.46	4688.15	4534.27
Protein JTB	FTLK	4138.82	4284.51	6236.36	8080.89
Protein JTB	FTLK	4718.26	5654.16	7475.93	9239.33
Protein JTB	FTLK	8334.66	9744.92	15902.56	18922.15
Protein JTB	LVIIR	3653.4	2562.22	4702.84	4564.1
Protein JTB	LVIIR	2066.67	3158	6897.09	4862.41
Protein JTB	TTPECGPTGYVEK	789.29	955.81	942.23	1074.1
Protein JTB	TTPECGPTGYVEK	513.34	347.07	949.58	674.93
Protein JTB	TTPECGPTGYVEK	408.49	493.34	534.28	794.26
Protein JTB	VALI	1296.99	1434.54	5346.49	5340.23
Protein JTB	VALI	3314.76	3998.01	22099.16	20825.3
Protein JTB	VALI	3659.85	3414.84	18140.3	21295.9
Myeloid-associated differentiation marker	ALTQPLGLLR	611.23	250.51	367.43	695.44
Myeloid-associated differentiation marker	TTITTTTTSSSGLGSPMIVGSPR	178.7	216.92	103.97	351.09
Myeloid-associated differentiation marker	FPLSWR	712.36	790.41	1427.67	857.56
Myeloid-associated differentiation marker	MPVTVTR	1292.85	1418.39	2596.94	3151.88
Myeloid-associated differentiation marker	ALTQPLGLLR	231.21	135.44	112.1	249.7
Myeloid-associated differentiation marker	ALTQPLGLLR	154.42	62.41	176.34	0

Myeloid-associated differentiation marker	FPLSWR	1264.83	782.61	639.78	816.21
Myeloid-associated differentiation marker	FPLSWR	488.59	238.51	453.1	205.81
Myeloid-associated differentiation marker	LGLL	1296.99	1434.54	5346.49	5340.23
Myeloid-associated differentiation marker	LGLL	667.11	261.48	2492.45	2908.78
Myeloid-associated differentiation marker	LGLL	3314.76	3998.01	22099.16	20825.3
Myeloid-associated differentiation marker	LGLL	3659.85	3414.84	18140.3	21295.9
Myeloid-associated differentiation marker	LGLL	332.65	326.7	492.06	841.9
Myeloid-associated differentiation marker	LGLLR	772.6	1009.43	1169.95	1102.84
Myeloid-associated differentiation marker	LGLLR	484.95	598.57	527.29	833.2
Myeloid-associated differentiation marker	LGLLR	889.11	1094.25	961.9	1189.95
Myeloid-associated differentiation marker	LGLLR	1108.53	1543.55	1229.93	1769.51
Myeloid-associated differentiation marker	LGLLR	980.63	1519.85	1222.12	845.75
Myeloid-associated differentiation marker	LGLLR	881.63	875.5	1172.56	719.34
Myeloid-associated differentiation marker	LGLLR	988.01	896.86	1055.42	1203.53
Myeloid-associated differentiation marker	LGLLR	252.97	539.23	483.12	768.99
Myeloid-associated differentiation marker	LGLLR	610.41	650.31	1125.48	924.24
Myeloid-associated differentiation marker	LGLLR	584.6	647.49	986.93	1677.42
Myeloid-associated differentiation marker	LGLLR	1447.92	1203.54	1220.32	1386.83
Myeloid-associated differentiation marker	LGLLR	801.22	734.4	1425.97	1094.17
Myeloid-associated differentiation marker	LLRL	912.1	950.18	1143.27	1280.65
Myeloid-associated differentiation marker	LLRL	556.67	959.36	927.48	1147.92
Myeloid-associated differentiation marker	TTITTTTTSSSGLGSPMIVGSPR	808.24	573.42	1225.34	998.61
Myeloid-associated differentiation marker	TTITTTTTSSSGLGSPMIVGSPR	153.12	191.37	185.14	281.37
Myeloid-associated differentiation marker	TTITTTTTSSSGLGSPMIVGSPR	1363.87	1422.58	2022.93	3901.98
Myeloid-associated differentiation marker	TTITTTTTSSSGLGSPMIVGSPR	178.7	216.92	103.97	351.09
Myeloid-associated differentiation marker	VFVK	984.88	780.68	1814.1	1640.08
Myeloid-associated differentiation marker	VFVK	921.32	571.73	1745.12	992.7
Myeloid-associated differentiation marker	VFVK	905.51	1814.23	2307.38	1956.18
RNA-binding protein FUS	APKPDGPGGGPGGSHMGGNYGDDR	218.2	144.88	331.2	514.92
RNA-binding protein FUS	GEATVSFDDPPSAK	462.84	230.06	965.8	920.35
RNA-binding protein FUS	TGQPMINLYTDR	456.59	208.92	757.52	678.83
RNA-binding protein FUS	AAIDWFDGK	1690.87	2200.75	3872.62	4678.16
RNA-binding protein FUS	CPNPTCENMNFSWR	231.04	152.22	559.19	379.89
RNA-binding protein FUS	QIGIIK	552.18	339.74	1595.82	1830.59
RNA-binding protein FUS	LKGEATVSFDDPPSAK	404.58	453.58	601.01	765.47
RNA-binding protein FUS	GGRGGGDRGGFGPGK	742.53	575.85	813.88	861.27
RNA-binding protein FUS	FGGPR	1838.5	1870.35	526.74	380.73
RNA-binding protein FUS	GGDRGGFR	1217.24	1016.16	2665.32	2309.57
RNA-binding protein FUS	AAIDWFDGK	1124.95	772.47	1547.74	1504.67
RNA-binding protein FUS	AAIDWFDGK	973.1	774.48	1419.34	1356.64
RNA-binding protein FUS	AAIDWFDGK	1137.38	927.55	1809.4	2608.44
RNA-binding protein FUS	AAIDWFDGK	841.14	727.19	1636.12	1857.17
RNA-binding protein FUS	AAIDWFDGK	1734.33	1099.03	2095.94	2309.46
RNA-binding protein FUS	APKPDGPGGGPGGSHMGGNYGDDR	42.51	55.15	94.7	158.27
RNA-binding protein FUS	APKPDGPGGGPGGSHMGGNYGDDR	196.1	200.22	493.63	604.55
RNA-binding protein FUS	CPNPTCENMNFSWR	126.55	263.27	555.12	433.73
RNA-binding protein FUS	CPNPTCENMNFSWR	231.04	152.22	559.19	379.89
RNA-binding protein FUS	FGGPR	3785.59	3119.23	440.73	513.11
RNA-binding protein FUS	GEATVSFDDPPSAK	844.55	709.19	1982.01	2143.95
RNA-binding protein FUS	GEATVSFDDPPSAK	1302.43	1216.16	1714.64	1859.46
RNA-binding protein FUS	GEATVSFDDPPSAK	533.26	506.89	1055.69	379.99
RNA-binding protein FUS	GGGNGR	1039.82	778.62	1129.88	1362.16
RNA-binding protein FUS	IGII	1296.99	1434.54	5346.49	5340.23
RNA-binding protein FUS	IGII	667.11	261.48	2492.45	2908.78
RNA-binding protein FUS	IGII	3314.76	3998.01	22099.16	20825.3
RNA-binding protein FUS	IGII	3659.85	3414.84	18140.3	21295.9
RNA-binding protein FUS	IGII	332.65	326.7	492.06	841.9
RNA-binding protein FUS	QIGIIK	408.77	465.35	1013.97	1253.81
RNA-binding protein FUS	QIGIIK	1245.03	1355.72	2361.43	2766.64
RNA-binding protein FUS	QIGIIK	1695.09	1570.93	4108.48	5299.81
RNA-binding protein FUS	QIGIIK	1965.56	1231.93	3610.65	3949.82
RNA-binding protein FUS	QIGIIK	408.27	514.39	1036.59	1219.28
RNA-binding protein FUS	QIGIIK	1484.27	1974.38	2636.06	3057.4
RNA-binding protein FUS	QIGIIK	1188.09	1236.48	2617.86	3275.82

RNA-binding protein FUS	QIGIIK	508.25	743.06	1805	2664.69
RNA-binding protein FUS	QIGIIK	1162.86	1175.69	3164.38	3516.78
RNA-binding protein FUS	QIGIIK	554.16	675.22	2067.25	2864.93
RNA-binding protein FUS	QIGIIK	2166.04	2276.32	4084.24	4616.54
RNA-binding protein FUS	QIGIIK	1521.86	920.27	3206.97	2824.07
RNA-binding protein FUS	QIGIIK	961.18	1101.74	1338.3	2712.24
RNA-binding protein FUS	QIGIIK	1012.87	781.27	3171.9	3076.45
RNA-binding protein FUS	TGQPMINLYTDR	225.51	698.6	1104.52	694.34
RNA-binding protein FUS	TGQPMINLYTDR	1283.68	1047.76	1815.74	1956.74
RNA-binding protein FUS	TGQPMINLYTDR	251.9	644.89	1483.85	870.92
RNA-binding protein FUS	TGQPMINLYTDR	350.97	218.64	664.2	948.12
Cellular nucleic acid-binding protein	CGETGHVAINCSK	288.51	431.71	1203.73	1096.57
Cellular nucleic acid-binding protein	EQCCYNCGKPGHLAR	427.45	321.74	1028.19	975.52
Cellular nucleic acid-binding protein	TSEVNCYR	214.44	90.61	263.86	370
Cellular nucleic acid-binding protein	CGETGHVAINCSK	621.15	652.71	1977.91	1883.76
Cellular nucleic acid-binding protein	EQCCYNCGKPGHLAR	133.45	113.68	376.95	459.39
Cellular nucleic acid-binding protein	EQCCYNCGKPGHLAR	275.38	346.14	697.86	675.36
Cellular nucleic acid-binding protein	EQCCYNCGKPGHLAR	59.67	61.8	128.65	156.47
Cellular nucleic acid-binding protein	TSEVNCYR	114.59	168.43	184.08	367.79
Cellular nucleic acid-binding protein	TSEVNCYR	286.05	462.32	602.85	484.09
60S ribosomal protein L37	GTSSFGK	863.37	445.23	1725.98	2356.64
60S ribosomal protein L37	AYHLQK	781.61	437.73	2237.35	1767.75
60S ribosomal protein L37	YNWSAK	3172.16	4257.33	7506.21	8840.63
60S ribosomal protein L37	AYHLQK	634.77	584.37	2276.64	2583.5
60S ribosomal protein L37	AYHLQK	388.74	422.75	2237.2	2474.5
60S ribosomal protein L37	GTSSFGK	1184.79	1561.93	2860.02	3463.73
60S ribosomal protein L37	GTSSFGK	1016.71	827.7	2669.62	1941.09
60S ribosomal protein L37	GTSSFGK	956.45	930.76	2366.66	2086.95
60S ribosomal protein L37	STCGKCGYPAK	140.98	114.3	211.92	411
60S ribosomal protein L37	YNWSAK	458.9	618.66	1118.7	1194.26
60S ribosomal protein L37	YNWSAK	2360.84	2072.92	3910.81	5118.02
60S ribosomal protein L37	YNWSAK	3116.97	3326.67	6991.39	9748.11
Sperm-associated antigen 7	SIEEAMNEIR	447.9	322.55	274.91	421.36
Sperm-associated antigen 7	AREQAARLK	880.39	1317.05	1515.37	1420.48
Sperm-associated antigen 7	LIGK	3497.2	4271.32	5463.79	5805.31
Sperm-associated antigen 7	LIGK	6358.35	8191.63	19930.68	27872.12
Sperm-associated antigen 7	LIGK	5627.84	5364.58	18307.75	21015.58
Sperm-associated antigen 7	LIGK	4093.22	3045.08	7609.02	8332.05
Sperm-associated antigen 7	LIGK	2955.88	3816.51	8510.03	11071
Sperm-associated antigen 7	LIGK	1741.15	1701.46	2482.79	3844.17
Sperm-associated antigen 7	MIFK	677.07	1085.37	1380.47	1550.73
Sperm-associated antigen 7	MIFK	1096.1	1069.33	1436.07	1561.67
Sperm-associated antigen 7	SIEEAMNEIR	229.69	285.48	355.26	417.19
Sperm-associated antigen 7	SIEEAMNEIR	346.9	301.12	428.38	511.26
Sperm-associated antigen 7	SIEEAMNEIR	409.67	376.09	319.63	539.21
Sperm-associated antigen 7	SIEEAMNEIR	384.58	301.97	248.29	497
Sperm-associated antigen 7	SIEEAMNEIR	280.9	333.25	357.59	235.46
Sperm-associated antigen 7	SIEEAMNEIR	410.17	326.83	201.68	279.17
Sperm-associated antigen 7	SIEEAMNEIR	383.66	393.13	476.31	437.47
Zinc transporter 9	LTELLENDPSVR	192.83	98.22	179.46	235.41
Zinc transporter 9	TPEELETFMLK	165.79	215.77	207.97	326.68
Zinc transporter 9	HVDLEIL	360.39	139.16	731.44	348.21
Zinc transporter 9	SLEVWGSPEALAR	334.71	123.12	342.58	316.19
Zinc transporter 9	SIQPEQVQR	180.04	84.87	248.1	231.39
Zinc transporter 9	EALLGR	3705.63	3359.52	6506.88	4776.22
Zinc transporter 9	EALLGR	4614.58	5365.6	6119.38	7833.73
Zinc transporter 9	EALLGR	4502.03	4145.08	6217.26	6139.92
Zinc transporter 9	EALLGR	4435.04	5764.05	5843.65	7256.25
Zinc transporter 9	EALLGR	2749.49	3250.34	3230.94	5672.09
Zinc transporter 9	EALLGR	9819.95	9810.07	14204.4	16157.99
Zinc transporter 9	EALLGR	1474.27	2004.88	2547.12	2951.51
Zinc transporter 9	EALLGR	8154.17	6831.84	10721.44	11973.49
Zinc transporter 9	EALLGR	2867.72	3377.92	4571.59	4349.36
Zinc transporter 9	GLGK	314.32	435.35	609.97	798.82

Zinc transporter 9	GLGK	272.38	216.83	403.38	919.32
Zinc transporter 9	HVDLEIL	230.2	235.92	252.04	525.85
Zinc transporter 9	LGISK	6212.44	5798.33	2687.24	3461.96
Zinc transporter 9	LGISK	6356.18	7980.2	3642.09	3873.59
Zinc transporter 9	LGISK	4918.09	6230.62	3546.17	4981.69
Zinc transporter 9	LGISK	6682.11	8544.92	2065.25	4492.65
Zinc transporter 9	LGISK	2985.09	2632.98	3439.12	3614.87
Zinc transporter 9	LGLGK	2356.27	2843.71	1808.85	1955.98
Zinc transporter 9	LGLGK	626.52	548.15	1811.38	2284.23
Zinc transporter 9	LGLGK	3374.36	2790.27	3114.67	3541.4
Zinc transporter 9	LGLGK	2417.43	3184.19	1443.74	1517.98
Zinc transporter 9	LGLGK	1274.08	1890.03	664.07	1384.76
Zinc transporter 9	LGLGK	1116.54	1602.81	1088.33	1783.18
Zinc transporter 9	LGLGK	2618.42	2689.07	1529.65	1972.05
Zinc transporter 9	LGLGK	2948.83	3980.86	3490.53	3780.81
Zinc transporter 9	LLAL	196.22	188.4	226.96	250.42
Zinc transporter 9	LLAL	75.78	172.69	115.6	69.95
Zinc transporter 9	LQVR	1159.51	1400.24	1323.59	1349.84
Zinc transporter 9	LQVR	482.56	728.27	822.48	814.74
Zinc transporter 9	LTELLENDPSVR	204.74	198.14	299.04	189.83
Zinc transporter 9	LTELLENDPSVR	152.87	215.86	251.13	178.29
Zinc transporter 9	LTELLENDPSVR	222.75	120.22	257.86	221.12
Zinc transporter 9	LTELLENDPSVR	1008.35	696.39	951.03	775.41
Zinc transporter 9	LTELLENDPSVR	335.25	69.8	424.95	261.26
Zinc transporter 9	SLEVWGSPEALAR	356.98	476.2	613.65	835.38
Zinc transporter 9	SYLEK	3331.68	2645.63	3511.26	3945.15
Zinc transporter 9	YLEK	1033.73	1380.04	1879.71	2790.94
Zinc transporter 9	YLEK	721.87	808.39	1247.96	1184.11
Zinc transporter 9	YLEK	430.2	901.5	955.69	1126.27
[Pyruvate dehydrogenase (acetyl-transferring)] kinase isozyme 2, mitochondrial	HYQTIQEAGDWCVPSTEPK	799.12	516.47	937.45	1085.92
[Pyruvate dehydrogenase (acetyl-transferring)] kinase isozyme 2, mitochondrial	DLSIK	1090.73	1281.11	2415.67	2601.54
[Pyruvate dehydrogenase (acetyl-transferring)] kinase isozyme 2, mitochondrial	EFLDK	29470.13	33626.17	1498.56	2512.54
[Pyruvate dehydrogenase (acetyl-transferring)] kinase isozyme 2, mitochondrial	EFLDK	7395.71	9212.98	1561.97	1459.32
[Pyruvate dehydrogenase (acetyl-transferring)] kinase isozyme 2, mitochondrial	EFLDK	1350.11	2569.58	812.22	1303.88
[Pyruvate dehydrogenase (acetyl-transferring)] kinase isozyme 2, mitochondrial	EFLDK	7015.5	9010.84	1252.65	1811.17
[Pyruvate dehydrogenase (acetyl-transferring)] kinase isozyme 2, mitochondrial	ELFK	2341.82	4378.22	2760.69	5011.31
[Pyruvate dehydrogenase (acetyl-transferring)] kinase isozyme 2, mitochondrial	ELFK	812.14	826.51	925.19	1053.71
[Pyruvate dehydrogenase (acetyl-transferring)] kinase isozyme 2, mitochondrial	ELFK	944.52	832.03	698.7	1348.21
[Pyruvate dehydrogenase (acetyl-transferring)] kinase isozyme 2, mitochondrial	ELFK	613.05	814.67	804	1196.58
[Pyruvate dehydrogenase (acetyl-transferring)] kinase isozyme 2, mitochondrial	FLDK	2467.9	2133.81	4495.56	5351.67
[Pyruvate dehydrogenase (acetyl-transferring)] kinase isozyme 2, mitochondrial	FLDK	1675.88	1128.61	2070.47	2141.71
[Pyruvate dehydrogenase (acetyl-transferring)] kinase isozyme 2, mitochondrial	IYLK	2324.6	1717.67	1510.6	2637.76
[Pyruvate dehydrogenase (acetyl-transferring)] kinase isozyme 2, mitochondrial	IYLK	743.59	896.22	1185.32	1083.92
[Pyruvate dehydrogenase (acetyl-transferring)] kinase isozyme 2, mitochondrial	IYLK	1557.06	1484.75	2494.9	3453.63
[Pyruvate dehydrogenase (acetyl-transferring)] kinase isozyme 2, mitochondrial	IYLK	1927.03	2272.69	2160.85	2555.31
[Pyruvate dehydrogenase (acetyl-transferring)] kinase isozyme 2, mitochondrial	IYLK	1294.53	2316.11	1214.3	1930.17
[Pyruvate dehydrogenase (acetyl-transferring)] kinase isozyme 2, mitochondrial	IYLK	518.49	582.81	492.64	1086.48
[Pyruvate dehydrogenase (acetyl-transferring)] kinase isozyme 2, mitochondrial	IYLK	2216.55	1647.85	2062.68	2686.04
[Pyruvate dehydrogenase (acetyl-transferring)] kinase isozyme 2, mitochondrial	IYLK	2343.22	3069.36	2768.3	3350.47
[Pyruvate dehydrogenase (acetyl-transferring)] kinase isozyme 2, mitochondrial	IYLK	2473.25	3626.5	3667.43	4183.62
[Pyruvate dehydrogenase (acetyl-transferring)] kinase isozyme 2, mitochondrial	IYLK	2509.04	2856.39	3440.44	4955.48
[Pyruvate dehydrogenase (acetyl-transferring)] kinase isozyme 2, mitochondrial	IYLK	1494.13	1319.79	2002.86	1611.01
[Pyruvate dehydrogenase (acetyl-transferring)] kinase isozyme 2, mitochondrial	IYLK	1695.62	1412.87	1872.62	2723.39
[Pyruvate dehydrogenase (acetyl-transferring)] kinase isozyme 2, mitochondrial	IYLK	1283.89	814.02	1090.45	971.03
[Pyruvate dehydrogenase (acetyl-transferring)] kinase isozyme 2, mitochondrial	LEYK	809.45	1091.77	1855.35	1415.93
[Pyruvate dehydrogenase (acetyl-transferring)] kinase isozyme 2, mitochondrial	LEYK	708.12	1102.77	1629.39	2302.64
[Pyruvate dehydrogenase (acetyl-transferring)] kinase isozyme 2, mitochondrial	LEYK	1804.24	1319.01	3138.79	3512.38
[Pyruvate dehydrogenase (acetyl-transferring)] kinase isozyme 2, mitochondrial	LSIK	4176.21	4527.06	6015.48	7909.73
[Pyruvate dehydrogenase (acetyl-transferring)] kinase isozyme 2, mitochondrial	LSIK	4182.42	4658.64	5547.87	6978.64

[Pyruvate dehydrogenase (acetyl-transferring)] kinase isozyme 2, mitochondrial	LSIK	1606.57	2657.57	2434.23	3278.86
[Pyruvate dehydrogenase (acetyl-transferring)] kinase isozyme 2, mitochondrial	LSIK	1409.61	1797.21	2312.38	3838.89
[Pyruvate dehydrogenase (acetyl-transferring)] kinase isozyme 2, mitochondrial	LSIK	3456	3022.5	4359.44	4742.76
[Pyruvate dehydrogenase (acetyl-transferring)] kinase isozyme 2, mitochondrial	LSIK	2878.2	3831.54	5604.06	6155.41
[Pyruvate dehydrogenase (acetyl-transferring)] kinase isozyme 2, mitochondrial	LSIK	2937.04	4188.04	5253.42	5847.66
[Pyruvate dehydrogenase (acetyl-transferring)] kinase isozyme 2, mitochondrial	LSIK	3252.42	4306.48	6749.05	8451.88
[Pyruvate dehydrogenase (acetyl-transferring)] kinase isozyme 2, mitochondrial	LSIK	3070.36	2718.79	3562.78	5338.49
[Pyruvate dehydrogenase (acetyl-transferring)] kinase isozyme 2, mitochondrial	LSIK	2693.93	4164.81	3942.42	5022.55
[Pyruvate dehydrogenase (acetyl-transferring)] kinase isozyme 2, mitochondrial	LSIK	3177.26	3220.87	3812.72	3898.27
[Pyruvate dehydrogenase (acetyl-transferring)] kinase isozyme 2, mitochondrial	LSIK	2575.5	4162.63	6452.12	7482.72
[Pyruvate dehydrogenase (acetyl-transferring)] kinase isozyme 2, mitochondrial	LSIK	2873	3068.29	6039.6	6558.52
[Pyruvate dehydrogenase (acetyl-transferring)] kinase isozyme 2, mitochondrial	LSIK	2476.65	2865.48	4811.9	4826.53
[Pyruvate dehydrogenase (acetyl-transferring)] kinase isozyme 2, mitochondrial	LSIK	2667.5	1988.95	4121.63	4224.93
[Pyruvate dehydrogenase (acetyl-transferring)] kinase isozyme 2, mitochondrial	LSIK	4234.88	3411.05	6964.9	7478.5
[Pyruvate dehydrogenase (acetyl-transferring)] kinase isozyme 2, mitochondrial	LSIK	8445.18	8612.45	10773.67	13482.85
[Pyruvate dehydrogenase (acetyl-transferring)] kinase isozyme 2, mitochondrial	LSIK	4313.71	6580.89	8061.56	9728.5
[Pyruvate dehydrogenase (acetyl-transferring)] kinase isozyme 2, mitochondrial	LSIK	1762.81	1369.86	2370.95	3090.65
[Pyruvate dehydrogenase (acetyl-transferring)] kinase isozyme 2, mitochondrial	LSIK	3641.4	3109.72	4843.86	6104.42
[Pyruvate dehydrogenase (acetyl-transferring)] kinase isozyme 2, mitochondrial	LSIK	2637.23	2851.18	4505.17	4971.66
[Pyruvate dehydrogenase (acetyl-transferring)] kinase isozyme 2, mitochondrial	LSIK	2350.83	1572.36	3336.75	3843.43
Thioredoxin-related transmembrane protein 4	VDVIQEPGLSGR	802.35	729.24	1198.37	1315.72
Thioredoxin-related transmembrane protein 4	ILEK	2168.52	2775.92	4147.86	5694.36
Thioredoxin-related transmembrane protein 4	ILEK	11589.07	11195.23	10157.11	11323.28
Thioredoxin-related transmembrane protein 4	ILEK	9225.81	10849.63	8041.91	10038.98
Thioredoxin-related transmembrane protein 4	ILEK	2738.47	3806.76	4044.26	5133.94
Thioredoxin-related transmembrane protein 4	ILEK	7311.03	8430.18	5489.29	8351.38
Thioredoxin-related transmembrane protein 4	ILEK	11190.85	11602.14	12444.6	16285.48
Thioredoxin-related transmembrane protein 4	VDVIQEPGLSGR	154.2	84.43	364.67	533.14
Thioredoxin-related transmembrane protein 4	VDVIQEPGLSGR	203.81	289.82	235.33	116.1
Thioredoxin-related transmembrane protein 4	VDVIQEPGLSGR	253.23	513.37	963.5	917.83
Thioredoxin-related transmembrane protein 4	VDVIQEPGLSGR	2019.36	2193.39	2606.89	2556.9
Thioredoxin-related transmembrane protein 4	VDVIQEPGLSGR	783.11	1140.93	1062.68	1318.93
Thioredoxin-related transmembrane protein 4	VDVIQEPGLSGR	1490.56	1991.4	2622.45	2512.22
ER membrane protein complex subunit 9	VPPVIVLENQGLR	101.68	167.32	286.41	396.44
ER membrane protein complex subunit 9	QMVGALLEDR	731.88	670.34	912.4	710.78
ER membrane protein complex subunit 9	ALAYVK	20198.58	21870.54	1155.09	2786.22
ER membrane protein complex subunit 9	ALAYVK	1749.86	2224.25	791.52	903.55
ER membrane protein complex subunit 9	ALAYVK	18297.25	19397.98	1074.27	2632.4
ER membrane protein complex subunit 9	ALAYVK	6984	8399.77	1703.03	2226.99
ER membrane protein complex subunit 9	ALAYVK	11990.65	15349.26	1757.26	2523.08
ER membrane protein complex subunit 9	ALAYVK	17732.8	18143.57	1313.2	2768.24
ER membrane protein complex subunit 9	ALAYVK	8779.03	9778.43	1392.69	3092.05
ER membrane protein complex subunit 9	ALAYVK	2791.5	3886.45	2088.83	2123.22
ER membrane protein complex subunit 9	ALAYVK	14059.6	17537.3	2957.6	5475.9
ER membrane protein complex subunit 9	LALK	4807.41	3973.87	7291.02	8675.79
ER membrane protein complex subunit 9	LALK	6462.81	5104.97	9207	10203.54
ER membrane protein complex subunit 9	LALK	4935.22	6984.92	7871.44	8905.76
ER membrane protein complex subunit 9	LALK	2767.42	2648.94	5681.47	7251.8
ER membrane protein complex subunit 9	LALK	5309.85	5717.08	7567.24	9480.53
ER membrane protein complex subunit 9	LALK	4754.16	5298.9	8162.92	10410.83
ER membrane protein complex subunit 9	LALK	2595.08	2972.84	5981.54	5760.8
ER membrane protein complex subunit 9	LALK	4855.52	4859.74	9143.68	9749.7
ER membrane protein complex subunit 9	LALK	3910.89	4463.17	9457.04	12051.94
ER membrane protein complex subunit 9	LALK	3537.6	4003.62	5686.03	6397.93
ER membrane protein complex subunit 9	LALK	3317.37	3314.27	5540.3	6042.49
ER membrane protein complex subunit 9	LALK	7119.65	6806.23	12375.83	13831.81
ER membrane protein complex subunit 9	LALK	6439.08	6598.25	14200.82	13875.28
ER membrane protein complex subunit 9	LALK	2604.25	1961	3076.21	4309.8
ER membrane protein complex subunit 9	LALK	1965.68	1964.77	3847.48	3742.81
ER membrane protein complex subunit 9	LALK	6416.21	8858.25	12646.49	15126.99
ER membrane protein complex subunit 9	LALK	2500.28	3296.77	4134.6	4446.36

ER membrane protein complex subunit 9	LALK	8357.22	9218.16	13686.1	18634.37
ER membrane protein complex subunit 9	LALK	6876.88	8344.44	12332.76	20178.62
ER membrane protein complex subunit 9	LALK	9922.49	10742.99	18210.58	20949.86
ER membrane protein complex subunit 9	LALK	3382.8	4084.33	5771.32	7290.97
ER membrane protein complex subunit 9	LALK	4034.46	4488.61	7107.29	7558.56
ER membrane protein complex subunit 9	LALK	7226.89	11105.02	14595.61	18163.31
ER membrane protein complex subunit 9	LALK	2073.81	2558.7	3804.49	3804.21
ER membrane protein complex subunit 9	LALK	4276.08	4872.5	7603.66	9278.12
ER membrane protein complex subunit 9	LALK	5269.75	6274.35	9402.47	9238.55
ER membrane protein complex subunit 9	LALK	4830.63	4339.64	6665.33	9453.04
ER membrane protein complex subunit 9	LALK	2033.88	1716.56	3641.52	4247.94
ER membrane protein complex subunit 9	LALK	2069.95	3366.15	4620.13	6283.3
ER membrane protein complex subunit 9	LDDIR	2720.26	2946.52	3686.77	4520.29
Potassium channel subfamily K member 1	QNEPFVATQSSACVDGPANH	180.83	6.66	71.86	150.96
Potassium channel subfamily K member 1	MLQSLAGSSCVR	127.54	165.74	269.99	88.68
Potassium channel subfamily K member 1	MFYVK	1331.23	1243.61	1527.88	1830.59
Potassium channel subfamily K member 1	EDLLR	6160.1	5225.04	4802.29	6456.38
Potassium channel subfamily K member 1	EDLLR	2113.85	2113.69	2054.56	2233.39
Potassium channel subfamily K member 1	EDLLR	6989.02	6521.22	4719.3	4355.54
Potassium channel subfamily K member 1	ELHELK	1561.36	1166.18	1961.66	2367.84
Potassium channel subfamily K member 1	LHELK	235.25	268.84	306.82	349.39
Potassium channel subfamily K member 1	LHELK	1088.91	1140.98	1960.61	1714.44
Potassium channel subfamily K member 1	LLGF	658.89	647.68	705.21	939.96
Potassium channel subfamily K member 1	LRQELR	1553.76	1172.45	2317.46	2589.96
Potassium channel subfamily K member 1	MLQSLAGSSCVR	814.87	662.55	1525.65	1464.16
Syntaxin-binding protein 6	EIFAPLDER	382.43	514.12	187.05	374.07
Syntaxin-binding protein 6	NSAQQFAETAHK	128.35	85.64	399.48	234.67
Syntaxin-binding protein 6	EIFAPLDER	318.93	405.16	224.62	218.06
Syntaxin-binding protein 6	EIFAPLDER	410.98	246.8	327.47	548.79
Syntaxin-binding protein 6	EIFAPLDER	204.86	185.5	285.79	369.44
dCTP pyrophosphatase 1	TDGEPGPQGWSPR	1518.47	2212.45	2489.38	3108.86
dCTP pyrophosphatase 1	FSFSPEPTLEDIRR	865.15	588.8	1160.13	1731.74
dCTP pyrophosphatase 1	VDLPLAVLSK	423.97	133.76	127.34	159.67
dCTP pyrophosphatase 1	AVLSK	6212.44	5798.33	2687.24	3461.96
dCTP pyrophosphatase 1	AVLSK	983.21	946.4	1601.52	1150.15
dCTP pyrophosphatase 1	AVLSK	6356.18	7980.2	3642.09	3873.59
dCTP pyrophosphatase 1	LAVL	667.11	261.48	2492.45	2908.78
dCTP pyrophosphatase 1	LAVLSK	774.48	1142.47	911.53	597.21
dCTP pyrophosphatase 1	LAVLSK	1770.01	2467.45	4485.07	3923.33
dCTP pyrophosphatase 1	LAVLSK	2288.26	2967.11	4198.11	4220.49
dCTP pyrophosphatase 1	LEDIR	1167.11	862.64	1826.34	1507.41
dCTP pyrophosphatase 1	LLAL	196.22	188.4	226.96	250.42
dCTP pyrophosphatase 1	LLAL	75.78	172.69	115.6	69.95
dCTP pyrophosphatase 1	TDGEPGPQGWSPR	177.67	319.4	410.35	233.69
dCTP pyrophosphatase 1	TDGEPGPQGWSPR	255.84	255.7	562.49	408.43
DNA mismatch repair protein Mlh1	RLDETVVNR	715.81	662.51	826.79	966.8
DNA mismatch repair protein Mlh1	STTSLTSSSTSGSSDK	204.39	231.92	171.67	175.79
DNA mismatch repair protein Mlh1	SIFGNAVSR	1112.83	693.32	1145.65	1295.03
DNA mismatch repair protein Mlh1	TLPNASTVDNIR	2571.57	1359.78	3309.85	3207.85
DNA mismatch repair protein Mlh1	EMIENCLDAK	126.02	315.79	512.62	556.12
DNA mismatch repair protein Mlh1	SFVAGVIR	385.62	198.98	232.81	374.31
DNA mismatch repair protein Mlh1	GPTSSNPR	269.77	156.24	218.6	91.43
DNA mismatch repair protein Mlh1	LIQIQDNGTGIR	101.66	168.92	207.32	450.97
DNA mismatch repair protein Mlh1	EFLK	1494.32	1619.85	2211.89	2094.92
DNA mismatch repair protein Mlh1	EFLK	2907.52	2317.5	3531.57	4064.15
DNA mismatch repair protein Mlh1	EFLK	2062.34	1776.67	3117.12	3247.33
DNA mismatch repair protein Mlh1	EFLK	1122.01	955.96	1896.21	2169.41
DNA mismatch repair protein Mlh1	EFLK	1210.3	957.17	1938.28	1796.53
DNA mismatch repair protein Mlh1	EFLK	1428.95	2014.48	3514.61	4106.41
DNA mismatch repair protein Mlh1	EFLK	748.57	1149.49	2042.55	1821.84
DNA mismatch repair protein Mlh1	EFLK	1505.78	1977.03	2054.07	2870.97
DNA mismatch repair protein Mlh1	EFLK	1768.23	3281.77	4421.66	4871.36
DNA mismatch repair protein Mlh1	EFLK	714.1	831.54	1513.97	1681.51
DNA mismatch repair protein Mlh1	EVIQR	2308.53	2347.95	3741.81	5374.7

DNA mismatch repair protein Mlh1	FILR	993.65	1617.09	1683.05	1522.96
DNA mismatch repair protein Mlh1	FILR	1306.18	1590.35	2020.16	2264.02
DNA mismatch repair protein Mlh1	FILR	2250.35	2670.49	3744.21	4025.15
DNA mismatch repair protein Mlh1	FILR	7295.87	8226.42	10784.35	13712.01
DNA mismatch repair protein Mlh1	FILR	1721.03	1135.92	2128.23	2658.95
DNA mismatch repair protein Mlh1	FSVK	534.5	703.32	802.58	916.36
DNA mismatch repair protein Mlh1	IFILR	978.96	1046.62	2244.37	2762.61
DNA mismatch repair protein Mlh1	ILEV	538.71	180.42	590.2	407.12
DNA mismatch repair protein Mlh1	ILEV	581.57	945.93	1022.29	951.69
DNA mismatch repair protein Mlh1	IVEFLK	1329.98	2005.4	71.25	146.71
DNA mismatch repair protein Mlh1	IVEFLK	2702.73	2656.93	150.76	297.03
DNA mismatch repair protein Mlh1	IVEFLK	2120.57	1981.25	17.53	202.03
DNA mismatch repair protein Mlh1	IVEFLK	2269.44	1954.78	302.8	186.17
DNA mismatch repair protein Mlh1	IVEFLK	5865.71	6757.3	0	602.32
DNA mismatch repair protein Mlh1	IVEFLK	484.78	402.94	46.09	146.9
DNA mismatch repair protein Mlh1	IVEFLK	5834.69	5565.98	246.3	610.54
DNA mismatch repair protein Mlh1	IVEFLK	1757.64	1455.41	179.32	245.98
DNA mismatch repair protein Mlh1	IVEFLK	868.11	1040.98	908.43	835.97
DNA mismatch repair protein Mlh1	IVEFLK	3024.77	4031.55	279.43	411.61
DNA mismatch repair protein Mlh1	IVEFLK	1868.48	2312.93	172.41	371.74
DNA mismatch repair protein Mlh1	IVEFLK	5143.14	6549.99	114.29	381.52
DNA mismatch repair protein Mlh1	IVEFLK	5091.47	5803.33	271.14	385.35
DNA mismatch repair protein Mlh1	IVEFLK	1554.2	1128.85	164.18	169.44
DNA mismatch repair protein Mlh1	IVEFLK	692.25	543.66	37.29	125.19
DNA mismatch repair protein Mlh1	IVEFLK	417.44	353.62	125.93	172.27
DNA mismatch repair protein Mlh1	LAFK	1793.47	1000.15	1876.92	2158.65
DNA mismatch repair protein Mlh1	LAFK	1711.02	1593.23	1883.12	3627.6
DNA mismatch repair protein Mlh1	LIGL	667.11	261.48	2492.45	2908.78
DNA mismatch repair protein Mlh1	STTSLTSSSTSGSSDK	235.67	226.84	468.05	325.91
DNA mismatch repair protein Mlh1	STTSLTSSSTSGSSDK	603.9	761.55	805.63	1185.49
DNA mismatch repair protein Mlh1	TLAFK	2011.31	2456.94	3162.99	4707.72
DNA mismatch repair protein Mlh1	TLPNASTVDNIR	2001.6	2458.73	3801.54	4188.27
DNA mismatch repair protein Mlh1	TLPNASTVDNIR	1536.45	2031.81	2316.85	3297.57
DNA mismatch repair protein Mlh1	TLPNASTVDNIR	1987.39	1734.93	4020.55	4891.46
DNA mismatch repair protein Mlh1	TLPNASTVDNIR	2693.47	2188.41	4531.64	5086.66
DNA mismatch repair protein Mlh1	TLPNASTVDNIR	1536.45	2031.81	2316.85	3297.57
DNA mismatch repair protein Mlh1	VEFLK	2458.61	1881.36	688.33	925.75
DNA mismatch repair protein Mlh1	VEFLK	3498.29	2355.17	2641.23	2554.76
DNA mismatch repair protein Mlh1	VEFLK	2048.16	2007.96	1514.82	2979.76
DNA mismatch repair protein Mlh1	VIVK	8409.48	7565.38	10626.73	12828.12
DNA mismatch repair protein Mlh1	VIVK	7855.86	9176.85	13631.04	15549.12
DNA mismatch repair protein Mlh1	YLPK	1983.48	2653.6	4494.01	4354.62
Vacuolar protein sorting-associated protein 4B	LHLGTTQNSLTEADFR	1315.03	1248.49	1824.63	2058.88
Vacuolar protein sorting-associated protein 4B	WSDVAGLEGAK	1066.87	1191.22	1630.65	2507.62
Vacuolar protein sorting-associated protein 4B	IYIPLPEPHAR	436.39	290.26	637.8	350.74
Vacuolar protein sorting-associated protein 4B	FTEDFGQEG	1242.34	664.1	1212.29	1202.26
Vacuolar protein sorting-associated protein 4B	GILLFGPPGTGK	49.65	201.01	87.27	97.36
Vacuolar protein sorting-associated protein 4B	AIDLASK	7987.47	8727.37	11234.52	14939.53
Vacuolar protein sorting-associated protein 4B	DALMQPVR	1757.36	1589.8	3585.42	3342.3
Vacuolar protein sorting-associated protein 4B	AAMFK	861.87	913.12	1351.77	1073.02
Vacuolar protein sorting-associated protein 4B	LGESEK	17899.53	24649.29	3557.38	5854.46
Vacuolar protein sorting-associated protein 4B	ARENK	693.54	1160.22	1659.89	1887.07
Vacuolar protein sorting-associated protein 4B	DLLK	483.65	620.77	657.51	919.8
Vacuolar protein sorting-associated protein 4B	DLLK	540.63	204.34	676.13	887.3
Vacuolar protein sorting-associated protein 4B	DLLK	521.31	729.63	932.37	1400.54
Vacuolar protein sorting-associated protein 4B	DLLK	1797.5	1985.09	1756.43	1876.51
Vacuolar protein sorting-associated protein 4B	DLLK	1306.03	1180	1309.5	1375.78
Vacuolar protein sorting-associated protein 4B	DLLK	359.27	249.9	363.8	1049.81
Vacuolar protein sorting-associated protein 4B	DLLK	2094.81	1972.93	2278.93	2601.34
Vacuolar protein sorting-associated protein 4B	DLLK	1492.76	1560.71	2483.4	2262.26
Vacuolar protein sorting-associated protein 4B	DLLK	619.46	926.01	1177.19	1371.24
Vacuolar protein sorting-associated protein 4B	DLLK	1375.58	904.24	1557.19	1713.44
Vacuolar protein sorting-associated protein 4B	DLLK	1112.05	1196.14	1062.07	1621.92
Vacuolar protein sorting-associated protein 4B	DLLK	202.84	385.47	613.68	578.56

Vacuolar protein sorting-associated protein 4B	DLLK	255.67	272.64	895.21	413.55
Vacuolar protein sorting-associated protein 4B	DLVSK	483.17	774.52	872.62	1082.38
Vacuolar protein sorting-associated protein 4B	DLVSK	266.6	256.48	377.74	584.72
Vacuolar protein sorting-associated protein 4B	EYLK	430.2	901.5	955.69	1126.27
Vacuolar protein sorting-associated protein 4B	FTEDFGQEG	860.64	715.45	1453.96	2120.11
Vacuolar protein sorting-associated protein 4B	GILLFGPPGTGK	76.82	70.32	69.66	99.38
Vacuolar protein sorting-associated protein 4B	GILLFGPPGTGK	102.7	66.3	173.9	67.24
Vacuolar protein sorting-associated protein 4B	GILLFGPPGTGK	153.9	113.69	195	118.5
Vacuolar protein sorting-associated protein 4B	IDSL	357.3	444.42	376.14	235.28
Vacuolar protein sorting-associated protein 4B	IDSL	382.05	658.05	515.6	643.4
Vacuolar protein sorting-associated protein 4B	ILLF	125.2	77.21	136.77	133.97
Vacuolar protein sorting-associated protein 4B	ILPIK	934.54	754.68	1316.7	1138.52
Vacuolar protein sorting-associated protein 4B	ILPIK	975.58	529.77	1175.11	747.74
Vacuolar protein sorting-associated protein 4B	ILPIK	411.8	166.2	481.82	692.3
Vacuolar protein sorting-associated protein 4B	ILPIK	637.98	692.09	659.66	508.99
Vacuolar protein sorting-associated protein 4B	ILPIK	3774.52	3787.88	7564.15	8710.41
Vacuolar protein sorting-associated protein 4B	ILPIK	978.43	673.44	1877.09	1558.47
Vacuolar protein sorting-associated protein 4B	ILPIK	2794.73	1972.22	4961.82	5548.74
Vacuolar protein sorting-associated protein 4B	ILPIK	4116.57	3823.81	8416.42	11247.83
Vacuolar protein sorting-associated protein 4B	ILPIK	2468.36	2194.89	4663.89	4451.87
Vacuolar protein sorting-associated protein 4B	ILPIK	1118.31	1853.37	3421	4338.18
Vacuolar protein sorting-associated protein 4B	ILPIK	1142.35	968.15	2229.61	2232.48
Vacuolar protein sorting-associated protein 4B	ILPIK	606.41	619.66	847.61	875.39
Vacuolar protein sorting-associated protein 4B	IYIPLPEPHAR	1151.48	1023.06	1500.66	1256.32
Vacuolar protein sorting-associated protein 4B	IYIPLPEPHAR	384.35	324.77	398.23	285.91
Vacuolar protein sorting-associated protein 4B	IYIPLPEPHAR	487.56	340.31	527.35	483.36
Vacuolar protein sorting-associated protein 4B	IYIPLPEPHAR	281.92	232.48	232.59	317.44
Vacuolar protein sorting-associated protein 4B	LGESEK	8089.46	7055	1940.82	2306.14
Vacuolar protein sorting-associated protein 4B	LGESEK	10756.59	11739.75	3771.54	5510.9
Vacuolar protein sorting-associated protein 4B	LPIK	607.14	1185.62	1411.98	1268.22
Vacuolar protein sorting-associated protein 4A	IYIPLPEEAAR	934.43	978.11	1275.16	941.6
Vacuolar protein sorting-associated protein 4A	LHLGSTPHNLTDANIHELAR	484.72	621.47	1842.09	1375.56
Vacuolar protein sorting-associated protein 4A	NLFELAR	356.86	487.73	1320.61	1262.36
Vacuolar protein sorting-associated protein 4A	DLLK	483.65	620.77	657.51	919.8
Vacuolar protein sorting-associated protein 4A	DLLK	540.63	204.34	676.13	887.3
Vacuolar protein sorting-associated protein 4A	DLLK	521.31	729.63	932.37	1400.54
Vacuolar protein sorting-associated protein 4A	DLLK	1797.5	1985.09	1756.43	1876.51
Vacuolar protein sorting-associated protein 4A	DLLK	1306.03	1180	1309.5	1375.78
Vacuolar protein sorting-associated protein 4A	DLLK	359.27	249.9	363.8	1049.81
Vacuolar protein sorting-associated protein 4A	DLLK	2094.81	1972.93	2278.93	2601.34
Vacuolar protein sorting-associated protein 4A	DLLK	1492.76	1560.71	2483.4	2262.26
Vacuolar protein sorting-associated protein 4A	DLLK	619.46	926.01	1177.19	1371.24
Vacuolar protein sorting-associated protein 4A	DLLK	1375.58	904.24	1557.19	1713.44
Vacuolar protein sorting-associated protein 4A	DLLK	1112.05	1196.14	1062.07	1621.92
Vacuolar protein sorting-associated protein 4A	DLLK	202.84	385.47	613.68	578.56
Vacuolar protein sorting-associated protein 4A	DLLK	255.67	272.64	895.21	413.55
Vacuolar protein sorting-associated protein 4A	DLMSK	3795.24	4931.86	7812.64	8787.54
Vacuolar protein sorting-associated protein 4A	DLMSK	1288.35	2075.07	3798.72	5146.57
Vacuolar protein sorting-associated protein 4A	GILLFGPPGTGK	49.65	201.01	87.27	97.36
Vacuolar protein sorting-associated protein 4A	GILLFGPPGTGK	76.82	70.32	69.66	99.38
Vacuolar protein sorting-associated protein 4A	GILLFGPPGTGK	102.7	66.3	173.9	67.24
Vacuolar protein sorting-associated protein 4A	GILLFGPPGTGK	153.9	113.69	195	118.5
Vacuolar protein sorting-associated protein 4A	ILLF	125.2	77.21	136.77	133.97
Vacuolar protein sorting-associated protein 4A	ILPIK	934.54	754.68	1316.7	1138.52
Vacuolar protein sorting-associated protein 4A	ILPIK	975.58	529.77	1175.11	747.74
Vacuolar protein sorting-associated protein 4A	ILPIK	411.8	166.2	481.82	692.3
Vacuolar protein sorting-associated protein 4A	ILPIK	637.98	692.09	659.66	508.99
Vacuolar protein sorting-associated protein 4A	ILPIK	3774.52	3787.88	7564.15	8710.41
Vacuolar protein sorting-associated protein 4A	ILPIK	978.43	673.44	1877.09	1558.47
Vacuolar protein sorting-associated protein 4A	ILPIK	2794.73	1972.22	4961.82	5548.74
Vacuolar protein sorting-associated protein 4A	ILPIK	4116.57	3823.81	8416.42	11247.83
Vacuolar protein sorting-associated protein 4A	ILPIK	2468.36	2194.89	4663.89	4451.87
Vacuolar protein sorting-associated protein 4A	ILPIK	1118.31	1853.37	3421	4338.18
Vacuolar protein sorting-associated protein 4A	ILPIK	1142.35	968.15	2229.61	2232.48

Vacuolar protein sorting-associated protein 4A	ILPIK	606.41	619.66	847.61	875.39
Vacuolar protein sorting-associated protein 4A	IYIPLPEEAAR	177.46	340.08	398.97	565.34
Vacuolar protein sorting-associated protein 4A	IYIPLPEEAAR	533.61	790.72	577.7	996.76
Vacuolar protein sorting-associated protein 4A	IYIPLPEEAAR	358.81	295.31	672.27	875.53
Vacuolar protein sorting-associated protein 4A	IYIPLPEEAAR	25.64	19.66	0	50.83
Vacuolar protein sorting-associated protein 4A	IYIPLPEEAAR	281.7	254.14	438.44	286
Vacuolar protein sorting-associated protein 4A	LFELAR	102.71	64.79	248.31	166.5
Vacuolar protein sorting-associated protein 4A	LGESEK	17899.53	24649.29	3557.38	5854.46
Vacuolar protein sorting-associated protein 4A	LGESEK	8089.46	7055	1940.82	2306.14
Vacuolar protein sorting-associated protein 4A	LGESEK	10756.59	11739.75	3771.54	5510.9
Vacuolar protein sorting-associated protein 4A	LPIK	607.14	1185.62	1411.98	1268.22
Exocyst complex component 7	AVEYFQDNSPDSPELNK	494.01	448.29	640.99	300.82
Exocyst complex component 7	GFNDGLEELCK	307.03	304.51	407.06	361.86
Exocyst complex component 7	LEEYLGSMAK	128.46	74.29	109.45	190.82
Exocyst complex component 7	EHIEQQIQTYQR	511.6	519.28	681.89	820.93
Exocyst complex component 7	YGVEQVGDMIDR	154.2	84.8	346.25	291.14
Exocyst complex component 7	AWAIPDTEQR	510.97	581.54	1061.64	987.78
Exocyst complex component 7	VTDYIAEK	10012.46	11215.91	1188.7	1072.57
Exocyst complex component 7	LLFERGK	461.42	370.03	323.47	418.78
Exocyst complex component 7	TRHSK	461.54	358.59	995.08	676.82
Exocyst complex component 7	AVEYFQDNSPDSPELNK	385.9	172.02	288.85	282.19
Exocyst complex component 7	DSLEK	2289.17	2770.89	3050.25	4134.82
Exocyst complex component 7	DSLEK	693.72	610.56	764.89	887.31
Exocyst complex component 7	DSLEK	1632.91	1331.37	2250.92	2214.68
Exocyst complex component 7	DSLEK	2723.3	2858.68	5520.85	8149.8
Exocyst complex component 7	DSLEK	669.08	811.25	1246.73	1469.16
Exocyst complex component 7	FLQK	3312.82	3551.44	3186.68	3370.69
Exocyst complex component 7	FLQK	2051.3	3399.92	2911.57	3980.78
Exocyst complex component 7	FLQK	6492.49	5363.91	7352.81	7479.64
Exocyst complex component 7	GFNDGLEELCK	256.08	231.94	353.96	569
Exocyst complex component 7	GFNDGLEELCK	334.24	169.5	602.93	558.07
Exocyst complex component 7	GLLP	206.92	0	95.22	278.73
Exocyst complex component 7	GNLQLNLLSK	582.77	721.84	453.84	471.62
Exocyst complex component 7	GNLQLNLLSK	582.77	721.84	453.84	471.62
Exocyst complex component 7	ILDL	538.71	180.42	590.2	407.12
Exocyst complex component 7	ILDL	581.57	945.93	1022.29	951.69
Exocyst complex component 7	LLFER	1951.11	1064.14	1644.48	1353.09
Exocyst complex component 7	LLFERGK	409.53	390.51	883.34	652.58
Exocyst complex component 7	LLSK	4234.88	3411.05	6964.9	7478.5
Exocyst complex component 7	LLSK	3980.54	4816.7	5542.92	8038.88
Exocyst complex component 7	LLSK	8445.18	8612.45	10773.67	13482.85
Exocyst complex component 7	LLSK	1471.64	1945.58	3628.55	5240.62
Exocyst complex component 7	LLSK	2693.93	4164.81	3942.42	5022.55
Exocyst complex component 7	LLSK	2350.83	1572.36	3336.75	3843.43
Exocyst complex component 7	LLSK	3177.26	3220.87	3812.72	3898.27
Exocyst complex component 7	LLSK	1518.04	2042.29	3833.99	4348.4
Exocyst complex component 7	LLSK	1351.59	1891.18	2291.31	2357.75
Exocyst complex component 7	LLSK	3181.41	3130.42	4495.21	6836.12
Exocyst complex component 7	LLSK	7071.17	9255.36	11240.11	15044.85
Exocyst complex component 7	LLSK	8217.78	10548.13	12023.17	14109.44
Exocyst complex component 7	LLSK	4182.42	4658.64	5547.87	6978.64
Exocyst complex component 7	LLSK	4313.71	6580.89	8061.56	9728.5
Exocyst complex component 7	LLSK	3641.4	3109.72	4843.86	6104.42
Exocyst complex component 7	LLSK	2637.23	2851.18	4505.17	4971.66
Exocyst complex component 7	LLSK	1994.71	2607.99	3198.42	3294.41
Exocyst complex component 7	LLSK	12618.85	11156.78	16157.18	19470.8
Exocyst complex component 7	LLSK	1409.61	1797.21	2312.38	3838.89
Exocyst complex component 7	LLSK	3887.16	3190.46	4151.19	5313.17
Exocyst complex component 7	LLSK	2878.2	3831.54	5604.06	6155.41
Exocyst complex component 7	LNKV	984.03	651.82	851.56	1355.47
Exocyst complex component 7	LSFIR	2258.1	2330.02	3802.96	3642.53
Exocyst complex component 7	LSFIR	3557.89	4232.24	5844.4	6219.67
Exocyst complex component 7	LSFIR	3643.36	3022.31	3469.21	5265.32
Exocyst complex component 7	LSFIR	2946.79	3331.18	3615.59	5125.07

Exocyst complex component 7	LSFIR	1558.08	1490.97	2023.86	1900.88
Exocyst complex component 7	NLLK	540.63	204.34	676.13	887.3
Exocyst complex component 7	NLLK	359.27	249.9	363.8	1049.81
Exocyst complex component 7	NLLK	202.84	385.47	613.68	578.56
Exocyst complex component 7	NLLSK	1332.71	1629.09	2612.92	3422.51
Exocyst complex component 7	QIIK	2159.8	3638.02	3341.89	4582.17
Exocyst complex component 7	QIIK	1331.14	1464.69	1921.29	2240.57
Exocyst complex component 7	QNLLK	2432.28	4270.55	5946.87	8139.86
Exocyst complex component 7	QNLLK	1200.97	1347.17	2360.93	3907
Exocyst complex component 7	VTDYIAEK	7086.88	9618.79	970.21	1845.43
Exocyst complex component 7	VTDYIAEK	5248.62	4958.24	5117.58	6610.07
Exocyst complex component 7	VTDYIAEK	7498.43	7362.27	715.31	1011.62
Exocyst complex component 7	VTDYIAEK	10794.14	10795.77	703.18	1500.68
Exocyst complex component 7	VTDYIAEK	10238.38	11236.13	956.23	1419.97
Exocyst complex component 7	VTDYIAEK	15878.62	17502.82	1666.98	1522.23
Exocyst complex component 7	VTDYIAEK	2739.31	3724.16	1531.38	2042.22
Exocyst complex component 7	VTDYIAEK	11357.74	11070.67	943.14	1394.14
Exocyst complex component 7	VTDYIAEK	9261.23	8414.13	1742.44	1623.11
Exocyst complex component 7	VTDYIAEK	1156.27	762.81	793.67	999.41
Exocyst complex component 7	VTDYIAEK	853.9	742.45	355.94	237.1
Exocyst complex component 7	YGVEQVGDMIDR	102.44	91.74	192.62	247.63
Exocyst complex component 7	YGVEQVGDMIDR	255.02	336.31	301.19	390.04
Exocyst complex component 7	YIAEK	20413.75	23803.27	25981.3	33100.46
Exocyst complex component 7	YIAEK	7649.31	9371.31	9608.52	12986.74
Exocyst complex component 7	YIAEK	1607.76	1050.49	2035.66	1826.77
Exocyst complex component 7	YIAEK	5789.75	7452.73	8459.87	9026.57
Exocyst complex component 7	YIAEK	2365.18	3559.61	3914.83	4209.53
Exocyst complex component 7	YILK	1494.13	1319.79	2002.86	1611.01
Exocyst complex component 7	YILK	1695.62	1412.87	1872.62	2723.39
Exocyst complex component 7	YILK	1557.06	1484.75	2494.9	3453.63
Exocyst complex component 7	YILK	1283.89	814.02	1090.45	971.03
Probable palmitoyltransferase ZDHHC20	LVGMDPEQASVTNQNEYAR	333.7	222.6	531.31	614.37
Probable palmitoyltransferase ZDHHC20	EGIVK	153.4	164.03	259.77	426.73
Probable palmitoyltransferase ZDHHC20	EGIVK	728.04	730.32	1416.93	1299.98
Probable palmitoyltransferase ZDHHC20	EGIVK	541.46	973.28	1092.56	951.76
Probable palmitoyltransferase ZDHHC20	EGIVK	205.13	159.21	308.71	517.72
Probable palmitoyltransferase ZDHHC20	EGIVK	281.16	307.2	368.8	771.11
Probable palmitoyltransferase ZDHHC20	EGIVK	330.72	516.99	979.64	830.05
Probable palmitoyltransferase ZDHHC20	ITFV	6247.1	4395.82	7418.04	8582.38
Probable palmitoyltransferase ZDHHC20	ITFV	8484.71	6941.7	11735.1	12459.63
Probable palmitoyltransferase ZDHHC20	ITFV	1720.85	1366.21	2386.29	2665.33
Probable palmitoyltransferase ZDHHC20	ITFV	462.46	267.13	409.78	338.23
Probable palmitoyltransferase ZDHHC20	QLIK	2159.8	3638.02	3341.89	4582.17
Probable palmitoyltransferase ZDHHC20	QLIK	1331.14	1464.69	1921.29	2240.57
Probable palmitoyltransferase ZDHHC20	QVFGDEK	19152.47	18840.85	7787.54	9315.26
Aldo-keto reductase family 1 member C2	YKPVCNQVECHPYFNQR	356	572.87	515.94	279.31
Aldo-keto reductase family 1 member C2	SIGVSNFNHR	832.48	624.86	1304.12	1337.2
Aldo-keto reductase family 1 member C2	AIDGLNR	1840.27	1908.16	1468.77	2316.03
Aldo-keto reductase family 1 member C2	AIRSK	1299.13	1116.91	2965.91	2381.52
Aldo-keto reductase family 1 member C2	ALIALR	2130.13	1462.15	2629.49	3688.47
Aldo-keto reductase family 1 member C2	ALIALR	1445.17	1901.1	2734.3	3054.56
Aldo-keto reductase family 1 member C2	ALIALR	891.42	971.87	1954.45	1432.67
Aldo-keto reductase family 1 member C2	DAGLAK	2440.6	3129.01	4354.65	6223.49
Aldo-keto reductase family 1 member C2	DIVLVAY	204.85	186.51	235.66	320.49
Aldo-keto reductase family 1 member C2	EDIFYTSK	487.59	337.43	670.15	1093.76
Aldo-keto reductase family 1 member C2	EDIFYTSK	5896.95	6117.37	6944.08	8426.08
Aldo-keto reductase family 1 member C2	EDIFYTSK	4211.43	4558.22	5515.5	6482.61
Aldo-keto reductase family 1 member C2	EDIFYTSK	5308.44	4899.55	5567.29	5847.32
Aldo-keto reductase family 1 member C2	EDIFYTSK	1530.18	1906.3	2803.82	2017.02
Aldo-keto reductase family 1 member C2	ELVR	334.17	388.86	709.55	798.59
Aldo-keto reductase family 1 member C2	ELVR	153.27	176.51	174.96	329.95
Aldo-keto reductase family 1 member C2	ELVR	180.45	257.22	444.12	348.36
Aldo-keto reductase family 1 member C2	GVVVLAK	899.73	1002.41	732.53	1190.29
Aldo-keto reductase family 1 member C2	GVVVLAK	2292.22	2470.17	1851.35	2830.15

Aldo-keto reductase family 1 member C2	GVVVLAK	6137.6	5852.19	4924.8	5955.8
Aldo-keto reductase family 1 member C2	GVVVLAK	2182.99	1559.03	3334.64	2677.53
Aldo-keto reductase family 1 member C2	GVVVLAK	2191.13	2031.97	3420.12	4326.19
Aldo-keto reductase family 1 member C2	IADGSVK	7063.25	8016.85	6099.47	7417.4
Aldo-keto reductase family 1 member C2	IADGSVK	8993.66	8785.51	6319.86	8334.01
Aldo-keto reductase family 1 member C2	IADGSVK	7400.73	6802	7086.63	6840.74
Aldo-keto reductase family 1 member C2	IADGSVK	4734.21	5142.79	4735.17	6269.9
Aldo-keto reductase family 1 member C2	IADGSVK	1865.02	2548.89	2395.49	2782.13
Aldo-keto reductase family 1 member C2	IADGSVK	4990.08	6671.64	5189.46	7127.81
Aldo-keto reductase family 1 member C2	IADGSVK	1332.42	1444.62	781.64	1763.02
Aldo-keto reductase family 1 member C2	IADGSVK	2473.89	2393.91	4213.56	4508.53
Aldo-keto reductase family 1 member C2	ILNK	3059.4	3270.14	3615.93	4312.52
Aldo-keto reductase family 1 member C2	ILNK	1259.27	1224.83	1231.17	1605.55
Aldo-keto reductase family 1 member C2	ILNK	1325.13	782.29	1817.38	2421.19
Aldo-keto reductase family 1 member C2	ILNK	3077.34	3518.82	4227.04	4991.5
Aldo-keto reductase family 1 member C2	ILNK	2975.59	3251.68	4836.32	5153.56
Aldo-keto reductase family 1 member C2	ILNK	2814.58	2350.52	3781.49	3872.46
Aldo-keto reductase family 1 member C2	ILNK	2417.43	3184.19	1443.74	1517.98
Aldo-keto reductase family 1 member C2	ILNK	5458.27	8180.95	5991.74	5731.38
Aldo-keto reductase family 1 member C2	ILNK	2618.42	2689.07	1529.65	1972.05
Aldo-keto reductase family 1 member C2	ILNK	1264.79	1317.9	1128.14	873.49
Aldo-keto reductase family 1 member C2	ILNK	12239	11238.33	15004.76	17913
Aldo-keto reductase family 1 member C2	ILNK	2356.27	2843.71	1808.85	1955.98
Aldo-keto reductase family 1 member C2	ILNK	4307.1	5106.36	6027.83	7668.98
Aldo-keto reductase family 1 member C2	ILNK	3115.72	4088.26	4055.47	4179.6
Aldo-keto reductase family 1 member C2	ILNK	1621.83	2851.96	1751.8	2219.03
Aldo-keto reductase family 1 member C2	LEAVK	770.82	759.85	1767.79	820.31
Aldo-keto reductase family 1 member C2	LEAVKL	2519.48	2463.71	1125.39	1156.47
Aldo-keto reductase family 1 member C2	LIAL	196.22	188.4	226.96	250.42
Aldo-keto reductase family 1 member C2	LIAL	75.78	172.69	115.6	69.95
Aldo-keto reductase family 1 member C2	LIALR	1064.7	1086.65	1758.36	1345.73
Aldo-keto reductase family 1 member C2	LLDFCK	2443.36	2430.64	1018.86	3437.38
Aldo-keto reductase family 1 member C2	LLDFCK	3156.71	2166.28	950.11	1164.04
Aldo-keto reductase family 1 member C2	LLDFCK	584.25	1213.06	297.88	341.24
Aldo-keto reductase family 1 member C2	LLDFCK	1598.31	1451.62	1375.91	2029.51
Aldo-keto reductase family 1 member C2	REDIFYTSK	3968.48	4412.33	7824.67	6872.69
Aldo-keto reductase family 1 member C2	REDIFYTSK	2379.98	2948.08	6399.02	5902.75
Aldo-keto reductase family 1 member C2	REDIFYTSK	2340.04	2851.25	3409.78	4893.78
Aldo-keto reductase family 1 member C2	REDIFYTSK	4228.89	4641.33	6233.41	8846.47
Aldo-keto reductase family 1 member C2	SKIADGSVK	1191.1	1577.59	1902.78	1815.65
Aldo-keto reductase family 1 member C2	TPALIALR	3378.64	2686.5	1051.33	1289.96
Aldo-keto reductase family 1 member C2	TPALIALR	2239.19	2602.1	1264.15	1806.51
Aldo-keto reductase family 1 member C2	TPALIALR	2255.81	2343.77	524.96	1222.51
Aldo-keto reductase family 1 member C2	TPALIALR	1179.07	1382.32	596.2	688.49
Aldo-keto reductase family 1 member C2	TPALIALR	6453.89	6518.47	4954.79	5246.53
Aldo-keto reductase family 1 member C2	TPALIALR	6915.88	6938.27	4888.33	5957.99
Aldo-keto reductase family 1 member C2	TPALIALR	3156.15	2753.16	2718.22	2439.14
Aldo-keto reductase family 1 member C2	TPALIALR	201.3	537.14	137.52	275.55
Aldo-keto reductase family 1 member C2	TPALIALR	1254.39	537.03	805.25	1115.26
Aldo-keto reductase family 1 member C2	TPALIALR	10811.29	10377.82	7326.01	7582.83
Aldo-keto reductase family 1 member C2	TPALIALR	13336.11	14548.57	9094.25	8652.33
Aldo-keto reductase family 1 member C2	TPALIALR	5367.82	4989.71	4134.26	4747.75
Aldo-keto reductase family 1 member C2	TPALIALR	1507.94	1231.21	1067.8	1360.47
Aldo-keto reductase family 1 member C2	TPALIALR	1140.76	1550.64	1399.39	1654.04
Aldo-keto reductase family 1 member C2	TPALIALR	154.15	89.45	116.76	199.02
Aldo-keto reductase family 1 member C2	YKPVCNQVECHPYFNQR	467.67	284.64	801.34	597.97
Aldo-keto reductase family 1 member C2	YKPVCNQVECHPYFNQR	4088.88	5070.41	786.79	1395.52
Aldo-keto reductase family 1 member C2	YQCVK	3918.4	3721.27	2193.51	2460.86
Monoacylglycerol lipase ABHD6	SSLDDLSIDGQVK	381.03	652.73	729.25	372.14
Monoacylglycerol lipase ABHD6	NLHLVCVDMPGHEGTTR	1967.3	1911.36	2813.41	2894.67
Monoacylglycerol lipase ABHD6	AAVEK	166.31	164.88	276.95	282.6
Monoacylglycerol lipase ABHD6	FLPK	3105.58	3175	3950.66	5222.85
Monoacylglycerol lipase ABHD6	LSVVK	1792.18	2723.96	1448.89	2084.68
Monoacylglycerol lipase ABHD6	SIDGQVK	3955.18	3917.87	2599.33	2547.64

NADH dehydrogenase [ubiquinone] 1 alpha subcomplex subunit 3	YSVMINK	815.52	1130.84	1177.21	1810.58
NADH dehydrogenase [ubiquinone] 1 alpha subcomplex subunit 3	VGAFLK	1544.64	1541.76	1635.03	1374.1
NADH dehydrogenase [ubiquinone] 1 alpha subcomplex subunit 3	VGAFLK	558.23	592.68	922.43	1231.57
NADH dehydrogenase [ubiquinone] 1 alpha subcomplex subunit 3	VGAFLK	947.18	994.66	1633.22	1815.68
NADH dehydrogenase [ubiquinone] 1 alpha subcomplex subunit 3	VGAFLK	1025.43	923.82	1871.92	1235.89
NADH dehydrogenase [ubiquinone] 1 alpha subcomplex subunit 3	VGAFLK	957.55	714.86	755.37	926.43
NADH dehydrogenase [ubiquinone] 1 alpha subcomplex subunit 3	VGAFLK	822.08	695.16	524.43	484.92
NADH dehydrogenase [ubiquinone] 1 alpha subcomplex subunit 3	VGAFLK	3542.88	3375.58	564.06	463.31
NADH dehydrogenase [ubiquinone] 1 alpha subcomplex subunit 3	VGAFLK	807.52	1176.54	1711.74	2755.84
26S proteasome non-ATPase regulatory subunit 3	AIQLEYSEAR	961.69	1369.79	1373.96	1220.45
26S proteasome non-ATPase regulatory subunit 3	AIQLEYSEARR	2968.81	3390.4	4168.99	4526.28
26S proteasome non-ATPase regulatory subunit 3	DGVIEASINHEK	409.22	420.88	658.79	627.77
26S proteasome non-ATPase regulatory subunit 3	ELDTVTLEDIK	835.69	308.31	834.88	792.8
26S proteasome non-ATPase regulatory subunit 3	ELDTVTLEDIKEHVK	937.14	1136.01	1911.93	2067.39
26S proteasome non-ATPase regulatory subunit 3	EMIDIYSTR	788.21	850.09	1122.89	1321.66
26S proteasome non-ATPase regulatory subunit 3	EPQLAFHQR	1741.91	2052.27	2519.61	2920
26S proteasome non-ATPase regulatory subunit 3	HDADGQATLLNLLLR	312.22	323.44	515.53	500.02
26S proteasome non-ATPase regulatory subunit 3	LQLDSPEDAEFIVAK	203.77	293.01	77.9	201.73
26S proteasome non-ATPase regulatory subunit 3	MISLSYSR	1212.16	1624.32	1907.92	2033.92
26S proteasome non-ATPase regulatory subunit 3	SVFPEQANNNEWAR	612.63	430.67	705.68	708.04
26S proteasome non-ATPase regulatory subunit 3	AVQGFFTSNNATR	744.72	784.69	1006.57	1935.05
26S proteasome non-ATPase regulatory subunit 3	EREQQDLEFAK	1314.14	910.1	1332.95	1734.9
26S proteasome non-ATPase regulatory subunit 3	LNHYVLYK	2089.79	2256.98	3139.26	5374.37
26S proteasome non-ATPase regulatory subunit 3	YLYYTGR	1562.48	1374.81	3073.17	2247.67
26S proteasome non-ATPase regulatory subunit 3	EQQDLEFAK	4919.45	4820.08	6189.96	7822.54
26S proteasome non-ATPase regulatory subunit 3	ISDDLMQK	3053.39	3118.51	4596.2	5304.04
26S proteasome non-ATPase regulatory subunit 3	FNQVLDQFGEK	115.35	92.77	94.35	84.63
26S proteasome non-ATPase regulatory subunit 3	ALDLVAAK	10562.66	12602.16	3522.41	5318.37
26S proteasome non-ATPase regulatory subunit 3	VYEFLDK	870.41	920.18	1290.71	626.98
26S proteasome non-ATPase regulatory subunit 3	GYVQSK	354.01	556.26	600.47	573.62
26S proteasome non-ATPase regulatory subunit 3	SFLHARLR	408.19	522.38	641.55	1198.07
26S proteasome non-ATPase regulatory subunit 3	AIQLEYSEAR	867.59	1092.95	1264.91	1368.42
26S proteasome non-ATPase regulatory subunit 3	AIQLEYSEAR	696.72	420.71	836.88	1141.74
26S proteasome non-ATPase regulatory subunit 3	AIQLEYSEAR	2968.81	3390.4	4168.99	4526.28
26S proteasome non-ATPase regulatory subunit 3	AIQLEYSEAR	2137.58	2003.14	4138.51	3898.15
26S proteasome non-ATPase regulatory subunit 3	ALDLVAAK	3965.27	4303.67	3371.44	4437.55
26S proteasome non-ATPase regulatory subunit 3	ALDLVAAK	2355.55	2383.23	2675.49	2562.48
26S proteasome non-ATPase regulatory subunit 3	ALDLVAAK	2845.83	1816.91	4106.39	3335.07
26S proteasome non-ATPase regulatory subunit 3	ALDLVAAK	23795.87	26252.41	1634.26	3374.62
26S proteasome non-ATPase regulatory subunit 3	AVGFK	1779.23	1130.29	2193.44	2355.82
26S proteasome non-ATPase regulatory subunit 3	AVQGFFTSNNATR	1803.14	2172.78	2450.98	2198.18
26S proteasome non-ATPase regulatory subunit 3	AVQGFFTSNNATR	1521.67	939.08	1692.17	1814.91
26S proteasome non-ATPase regulatory subunit 3	AVQGFFTSNNATR	1560.47	1466.7	2311.27	1822.42
26S proteasome non-ATPase regulatory subunit 3	AVQGFFTSNNATR	1521.67	939.08	1692.17	1814.91
26S proteasome non-ATPase regulatory subunit 3	AVQGFFTSNNATR	559.02	514.74	573.13	872.75
26S proteasome non-ATPase regulatory subunit 3	AVQGFFTSNNATR	559.02	514.74	573.13	872.75
26S proteasome non-ATPase regulatory subunit 3	DGVIEASINHEK	926.66	1426.19	2063.42	1885.91
26S proteasome non-ATPase regulatory subunit 3	DGVIEASINHEK	2119.99	2251.65	2760.43	2428.24
26S proteasome non-ATPase regulatory subunit 3	DGVIEASINHEK	1670.52	1764.63	3074.01	4090.1
26S proteasome non-ATPase regulatory subunit 3	EFLDK	29470.13	33626.17	1498.56	2512.54
26S proteasome non-ATPase regulatory subunit 3	EFLDK	7395.71	9212.98	1561.97	1459.32
26S proteasome non-ATPase regulatory subunit 3	EFLDK	1350.11	2569.58	812.22	1303.88
26S proteasome non-ATPase regulatory subunit 3	EFLDK	7015.5	9010.84	1252.65	1811.17
26S proteasome non-ATPase regulatory subunit 3	ELDTVTLEDIK	617.49	482.71	613.78	762.21
26S proteasome non-ATPase regulatory subunit 3	ELDTVTLEDIK	358.22	353.58	345.22	596.22
26S proteasome non-ATPase regulatory subunit 3	ELDTVTLEDIKEHVK	985.53	1674.28	3352	3349.13
26S proteasome non-ATPase regulatory subunit 3	ELDTVTLEDIKEHVK	915.45	1044.75	1718.55	1908.99
26S proteasome non-ATPase regulatory subunit 3	ELDTVTLEDIKEHVK	154.45	59.94	298.57	214.25
26S proteasome non-ATPase regulatory subunit 3	ELDTVTLEDIKEHVK	2631.15	2601.53	3579.26	4261.96
26S proteasome non-ATPase regulatory subunit 3	EMIDIYSTR	2764.34	3484.7	3870.45	4809.27
26S proteasome non-ATPase regulatory subunit 3	EMIDIYSTR	2557.65	3373.83	4420.87	5073.94
26S proteasome non-ATPase regulatory subunit 3	EMIDIYSTR	981.17	721.61	1132.84	1210.61

26S proteasome non-ATPase regulatory subunit 3	EMIDIYSTR	1972.93	1568.4	2843.51	3007.54
26S proteasome non-ATPase regulatory subunit 3	EMIDIYSTR	1844.02	1431.31	2703.77	3417.08
26S proteasome non-ATPase regulatory subunit 3	EMIDIYSTR	2106.74	2390.58	2851.64	2565.21
26S proteasome non-ATPase regulatory subunit 3	EMIDIYSTR	461.22	390.22	761.77	1010.42
26S proteasome non-ATPase regulatory subunit 3	EMIDIYSTR	1399.6	1722.7	1566.65	2379.12
26S proteasome non-ATPase regulatory subunit 3	EMIDIYSTR	405.52	787.02	964.64	896.94
26S proteasome non-ATPase regulatory subunit 3	EPQLAFHQR	609.08	569.23	832.28	917.36
26S proteasome non-ATPase regulatory subunit 3	EQQDLEFAK	1523.89	1889.93	2519.74	1857.95
26S proteasome non-ATPase regulatory subunit 3	EQQDLEFAK	589.73	1204.21	874.92	1552.34
26S proteasome non-ATPase regulatory subunit 3	EQQDLEFAK	2016.94	1475.39	2495.92	1945.86
26S proteasome non-ATPase regulatory subunit 3	EQQDLEFAK	4022.25	3674.12	5634.86	4152.76
26S proteasome non-ATPase regulatory subunit 3	EQQDLEFAK	1205.39	1654.72	1386.02	1656.93
26S proteasome non-ATPase regulatory subunit 3	EREQQDLEFAK	1572.98	1613.93	2695.2	4156.64
26S proteasome non-ATPase regulatory subunit 3	EREQQDLEFAK	1596.01	2104.39	2976.33	3099.55
26S proteasome non-ATPase regulatory subunit 3	FLDK	2467.9	2133.81	4495.56	5351.67
26S proteasome non-ATPase regulatory subunit 3	FLDK	1675.88	1128.61	2070.47	2141.71
26S proteasome non-ATPase regulatory subunit 3	FVLR	976.09	1117.79	4000.14	3822.14
26S proteasome non-ATPase regulatory subunit 3	FVLR	1115.99	1019.13	2835.9	3064.67
26S proteasome non-ATPase regulatory subunit 3	FVLR	1107.62	888.16	2537	2709.99
26S proteasome non-ATPase regulatory subunit 3	ISDDLMQK	2983.43	3435.25	1417.82	2509.81
26S proteasome non-ATPase regulatory subunit 3	ISDDLMQK	3746.53	3573.79	5651.18	7329.46
26S proteasome non-ATPase regulatory subunit 3	ISDDLMQK	3351.89	5116.76	3588.86	3831.77
26S proteasome non-ATPase regulatory subunit 3	LDKL	2055.16	1635.69	2617.23	3027.78
26S proteasome non-ATPase regulatory subunit 3	LEDIK	1127.43	1165.45	1779.47	1825.99
26S proteasome non-ATPase regulatory subunit 3	LEDIK	1091.11	1455.77	1220.86	2041.96
26S proteasome non-ATPase regulatory subunit 3	LEDIK	2805.77	3966.34	4999.15	6632.05
26S proteasome non-ATPase regulatory subunit 3	LEDIK	1160.78	955.7	2033.29	2250.35
26S proteasome non-ATPase regulatory subunit 3	LEDIK	1141.76	1451.42	1942.23	2126.54
26S proteasome non-ATPase regulatory subunit 3	LEDIK	1292.9	881.59	1917.22	1970.76
26S proteasome non-ATPase regulatory subunit 3	LEFAK	1205.33	1128.44	2580.21	2581.45
26S proteasome non-ATPase regulatory subunit 3	LIIR	386.99	489.76	684.01	680.12
26S proteasome non-ATPase regulatory subunit 3	LIIR	912.1	950.18	1143.27	1280.65
26S proteasome non-ATPase regulatory subunit 3	LIIR	556.67	959.36	927.48	1147.92
26S proteasome non-ATPase regulatory subunit 3	LIIR	1415.65	1626.01	1823.04	1846.6
26S proteasome non-ATPase regulatory subunit 3	LIIR	279.39	481.67	627.51	552.92
26S proteasome non-ATPase regulatory subunit 3	LIIR	487.03	393.55	449.11	438.94
26S proteasome non-ATPase regulatory subunit 3	LIIRL	229.51	303.98	717.46	474.46
26S proteasome non-ATPase regulatory subunit 3	LLLR	386.99	489.76	684.01	680.12
26S proteasome non-ATPase regulatory subunit 3	LLLR	769.62	771.66	1081.49	1259.46
26S proteasome non-ATPase regulatory subunit 3	LLLR	864.03	699.71	1475.57	835.82
26S proteasome non-ATPase regulatory subunit 3	LLLR	912.1	950.18	1143.27	1280.65
26S proteasome non-ATPase regulatory subunit 3	LLLR	556.67	959.36	927.48	1147.92
26S proteasome non-ATPase regulatory subunit 3	LLLR	1415.65	1626.01	1823.04	1846.6
26S proteasome non-ATPase regulatory subunit 3	LLLR	279.39	481.67	627.51	552.92
26S proteasome non-ATPase regulatory subunit 3	LLLR	487.03	393.55	449.11	438.94
26S proteasome non-ATPase regulatory subunit 3	LNHYVLYK	1991.45	3036.33	4734.22	4417.53
26S proteasome non-ATPase regulatory subunit 3	LNHYVLYK	491.74	672.08	748.99	767.94
26S proteasome non-ATPase regulatory subunit 3	LNHYVLYK	1122.8	1516.55	2034.41	1962.55
26S proteasome non-ATPase regulatory subunit 3	LQLDSPEDAEFIVAK	358.5	325.38	462.56	716.68
26S proteasome non-ATPase regulatory subunit 3	MISLSYSR	612.88	724.89	831.12	985.8
26S proteasome non-ATPase regulatory subunit 3	MISLSYSR	1067.11	848.45	1086.09	1945.01
26S proteasome non-ATPase regulatory subunit 3	SLYDQAEK	205.91	82.49	271.17	216.85
26S proteasome non-ATPase regulatory subunit 3	SVFPEQANNNEWAR	1334.05	1177.78	1635.38	1967.76
26S proteasome non-ATPase regulatory subunit 3	SVFPEQANNNEWAR	333.43	249.19	493.37	179.95
26S proteasome non-ATPase regulatory subunit 3	SVFPEQANNNEWAR	729.82	767.74	995.75	1584.23
26S proteasome non-ATPase regulatory subunit 3	SVFPEQANNNEWAR	757.97	964.91	1670.91	1887.02
26S proteasome non-ATPase regulatory subunit 3	SVFPEQANNNEWAR	757.97	964.91	1670.91	1887.02
26S proteasome non-ATPase regulatory subunit 3	SVFPEQANNNEWAR	757.97	964.91	1670.91	1887.02
26S proteasome non-ATPase regulatory subunit 3	SVFPEQANNNEWAR	774.5	927.46	1867.02	1798.41
26S proteasome non-ATPase regulatory subunit 3	SVFPEQANNNEWAR	774.5	927.46	1867.02	1798.41
26S proteasome non-ATPase regulatory subunit 3	SVFPEQANNNEWAR	459.25	584.63	566.64	912.8
26S proteasome non-ATPase regulatory subunit 3	SVFPEQANNNEWAR	799.43	911.81	1166.78	1871.64
26S proteasome non-ATPase regulatory subunit 3	SVFPEQANNNEWAR	1045.43	1075.05	1285.68	1585.83
26S proteasome non-ATPase regulatory subunit 3	SVFPEQANNNEWAR	774.5	927.46	1867.02	1798.41

26S proteasome non-ATPase regulatory subunit 3	SVFPEQANNNEWAR	777.54	627.71	832.87	1115.95
26S proteasome non-ATPase regulatory subunit 3	SVFPEQANNNEWAR	610.5	640.86	1007.18	772.48
26S proteasome non-ATPase regulatory subunit 3	VYEFLDK	681.65	845.79	662.19	737.56
26S proteasome non-ATPase regulatory subunit 3	YLYYTGR	2091.53	1765.2	3258.21	3611.67
ER membrane protein complex subunit 2	ILQEDPTNTAAR	359.04	271.96	550.19	486.52
ER membrane protein complex subunit 2	YDDAIQLYDR	411	245.15	408.95	416.52
ER membrane protein complex subunit 2	AVEDMLETLQITQS	38.41	34.64	60.68	48.34
ER membrane protein complex subunit 2	FEAMER	541.24	888.68	1023.08	1366.69
ER membrane protein complex subunit 2	AQALK	510.45	632.33	838.51	1051.07
ER membrane protein complex subunit 2	AQALK	735	1000.94	1249.24	1590.88
ER membrane protein complex subunit 2	AVEDMLETLQITQS	51.74	0	102.72	20.4
ER membrane protein complex subunit 2	ELSRK	839	1470.76	1722.88	1731.87
ER membrane protein complex subunit 2	IAIRK	3277.22	3558.51	945.89	791.13
ER membrane protein complex subunit 2	IAIRK	1433.28	1054.93	1125.54	466.81
ER membrane protein complex subunit 2	IAIRK	1352.52	1267.71	1002.16	1089.73
ER membrane protein complex subunit 2	IAIRK	2126.67	2336.79	599.04	502.96
ER membrane protein complex subunit 2	ILQEDPTNTAAR	333.42	250.79	414.76	467.79
ER membrane protein complex subunit 2	ILQEDPTNTAAR	205.89	84.12	190.53	322
ER membrane protein complex subunit 2	ILQEDPTNTAAR	612.04	1020.84	1566.53	1438.68
ER membrane protein complex subunit 2	YDDAIQLYDR	643.53	676.23	838.89	899.42
ER membrane protein complex subunit 2	YDDAIQLYDR	368.49	508.38	681.81	858.51
ER membrane protein complex subunit 2	YDDAIQLYDR	152.35	266.85	283.86	268.34
ER membrane protein complex subunit 2	YDDAIQLYDR	254.27	410.09	324.22	329.35
ER membrane protein complex subunit 2	YDDAIQLYDR	659.91	759.33	1455.28	1719.03
Cystathionine gamma-lyase	QGAPGQHSGFEYSR	228.15	225.33	464.15	387.57
Cystathionine gamma-lyase	NDRDVLGISDTLIR	1045.38	1186.4	2267.25	1812.36
Cystathionine gamma-lyase	EIFLK	1147.73	1181.67	1541	2287.84
Cystathionine gamma-lyase	EIFLK	255.87	252.53	612.43	432.7
Cystathionine gamma-lyase	EIFLK	6520.37	6439.62	685.95	1228.61
Cystathionine gamma-lyase	EIFLK	868.14	718.71	1265.36	1637.86
Cystathionine gamma-lyase	EIFLK	3981.09	3378.83	392.91	538.85
Cystathionine gamma-lyase	EIFLK	5703.03	5382.34	451.18	623.75
Cystathionine gamma-lyase	ELVK	309.61	49.15	263.79	522.29
Cystathionine gamma-lyase	ELVK	152.9	212.58	413.29	361.64
Cystathionine gamma-lyase	ELVK	332.91	301.03	484.35	333
Cystathionine gamma-lyase	FGLK	838.74	964.25	1992.45	1300.43
Cystathionine gamma-lyase	FYIK	1069.88	1425.98	2772.48	2325.73
Cystathionine gamma-lyase	FYIK	1397.9	1784.48	2240.2	2349.9
Cystathionine gamma-lyase	IFLK	2800.73	2655.93	2713.72	2747.15
Cystathionine gamma-lyase	IFLK	848.14	885.96	2426.69	2268.47
Cystathionine gamma-lyase	IFLK	1293.11	2456.52	2411.93	2123.8
Cystathionine gamma-lyase	IFLK	2470.97	2256.62	2983.58	3778.41
Cystathionine gamma-lyase	IFLK	1295.7	1136.43	2013.16	2203.91
Cystathionine gamma-lyase	IFLK	1376.94	1088.83	1995.35	2068.54
Cystathionine gamma-lyase	IFLK	3125.37	3879.87	3903.38	4277.33
Cystathionine gamma-lyase	IFLK	562.75	571.7	1039.53	1023.44
Cystathionine gamma-lyase	IFLK	1216.57	1507.36	1879.53	2265.18
Cystathionine gamma-lyase	NDRDVLGISDTLIR	1363.15	1281.23	1996.51	1524.21
Cystathionine gamma-lyase	QGAPGQHSGFEYSR	0	102.93	18.65	19.47
Cystathionine gamma-lyase	QGAPGQHSGFEYSR	38.28	47.57	59.86	48.35
Synaptogyrin-2	QMYCVFNR	1488.53	1446.63	1652.8	1699.04
Synaptogyrin-2	MESGAYGAAK	714.27	1346.66	925.06	1148.46
Synaptogyrin-2	AGGSFDLR	1375.59	2285.72	3005.32	2870.36
Synaptogyrin-2	AAITF	1906.79	1506.97	326.64	367.1
Synaptogyrin-2	AAITF	1609.38	1422.49	436.23	283.24
Synaptogyrin-2	MESGAYGAAK	2606.58	2369.02	2511.71	3086.91
Synaptogyrin-2	MESGAYGAAK	202.54	414.75	708.91	244.83
Synaptogyrin-2	MESGAYGAAK	333.63	229.77	177.01	454.55
Synaptogyrin-2	QMYCVFNR	805.03	783.99	979.91	968.12
Synaptogyrin-2	QMYCVFNR	1061.34	992.55	1266.13	951.71
N-acetylglucosamine-1-phosphodiester alpha-N-acetylglucosaminidase	QVSTVVCVHEPR	436.9	239	618.51	1060.13
N-acetylglucosamine-1-phosphodiester alpha-N-acetylglucosaminidase	VAQNGGFFR	2220.52	3382.97	4193.79	3923.4
N-acetylglucosamine-1-phosphodiester alpha-N-acetylglucosaminidase	EQPGGAHNPFK	656.7	651.37	1860.76	1249.86

N-acetylglucosamine-1-phosphodiester alpha-N-acetylglucosaminidase	EFLLK	308.85	124.53	367.58	341.56
N-acetylglucosamine-1-phosphodiester alpha-N-acetylglucosaminidase	EFLLK	3981.09	3378.83	392.91	538.85
N-acetylglucosamine-1-phosphodiester alpha-N-acetylglucosaminidase	EFLLK	2209.46	2454.97	1843.05	2609.3
N-acetylglucosamine-1-phosphodiester alpha-N-acetylglucosaminidase	EFLLK	3394.01	3614.75	4810.19	6039.68
N-acetylglucosamine-1-phosphodiester alpha-N-acetylglucosaminidase	EFLLK	5703.03	5382.34	451.18	623.75
N-acetylglucosamine-1-phosphodiester alpha-N-acetylglucosaminidase	EFLLK	6520.37	6439.62	685.95	1228.61
N-acetylglucosamine-1-phosphodiester alpha-N-acetylglucosaminidase	EFLLK	3244.52	4341.7	2323.16	2256.4
N-acetylglucosamine-1-phosphodiester alpha-N-acetylglucosaminidase	EFLLK	502.26	271.51	1324.03	136.2
N-acetylglucosamine-1-phosphodiester alpha-N-acetylglucosaminidase	EFLLK	3992.84	5622	2629.55	3009.99
N-acetylglucosamine-1-phosphodiester alpha-N-acetylglucosaminidase	FLLK	1512.27	2612.26	3319.52	3799.41
N-acetylglucosamine-1-phosphodiester alpha-N-acetylglucosaminidase	FLLK	848.14	885.96	2426.69	2268.47
N-acetylglucosamine-1-phosphodiester alpha-N-acetylglucosaminidase	FLLK	690.7	802.52	1226.74	1724.59
N-acetylglucosamine-1-phosphodiester alpha-N-acetylglucosaminidase	FLLK	1293.11	2456.52	2411.93	2123.8
N-acetylglucosamine-1-phosphodiester alpha-N-acetylglucosaminidase	FLLK	1075.62	1284.45	1614.56	3127.26
N-acetylglucosamine-1-phosphodiester alpha-N-acetylglucosaminidase	FLLK	1216.57	1507.36	1879.53	2265.18
N-acetylglucosamine-1-phosphodiester alpha-N-acetylglucosaminidase	LLLR	386.99	489.76	684.01	680.12
N-acetylglucosamine-1-phosphodiester alpha-N-acetylglucosaminidase	LLLR	769.62	771.66	1081.49	1259.46
N-acetylglucosamine-1-phosphodiester alpha-N-acetylglucosaminidase	LLLR	864.03	699.71	1475.57	835.82
N-acetylglucosamine-1-phosphodiester alpha-N-acetylglucosaminidase	LLLR	912.1	950.18	1143.27	1280.65
N-acetylglucosamine-1-phosphodiester alpha-N-acetylglucosaminidase	LLLR	556.67	959.36	927.48	1147.92
N-acetylglucosamine-1-phosphodiester alpha-N-acetylglucosaminidase	LLLR	1415.65	1626.01	1823.04	1846.6
N-acetylglucosamine-1-phosphodiester alpha-N-acetylglucosaminidase	LLLR	279.39	481.67	627.51	552.92
N-acetylglucosamine-1-phosphodiester alpha-N-acetylglucosaminidase	LLLR	487.03	393.55	449.11	438.94
N-acetylglucosamine-1-phosphodiester alpha-N-acetylglucosaminidase	LLLRL	229.51	303.98	717.46	474.46
N-acetylglucosamine-1-phosphodiester alpha-N-acetylglucosaminidase	LLRL	912.1	950.18	1143.27	1280.65
N-acetylglucosamine-1-phosphodiester alpha-N-acetylglucosaminidase	LLRL	556.67	959.36	927.48	1147.92
N-acetylglucosamine-1-phosphodiester alpha-N-acetylglucosaminidase	VAQNGGFFR	2220.52	3382.97	4193.79	3923.4
Transcription factor A, mitochondrial	SAYNVYVAER	1685.1	1707.04	3316.25	2272.13
Transcription factor A, mitochondrial	SWEEQMIEVGR	204.84	187.57	183.15	351.18
Transcription factor A, mitochondrial	SSVLASCPK	359.02	273.97	450.43	727.14
Transcription factor A, mitochondrial	EQLPIFK	1123.71	682	1505.95	1905.66
Transcription factor A, mitochondrial	FVYL	187.44	98.1	151.65	161.09
Transcription factor A, mitochondrial	LLGK	3497.2	4271.32	5463.79	5805.31
Transcription factor A, mitochondrial	LLGK	6358.35	8191.63	19930.68	27872.12
Transcription factor A, mitochondrial	LLGK	5627.84	5364.58	18307.75	21015.58
Transcription factor A, mitochondrial	LLGK	4093.22	3045.08	7609.02	8332.05
Transcription factor A, mitochondrial	LLGK	2955.88	3816.51	8510.03	11071
Transcription factor A, mitochondrial	LLGK	1741.15	1701.46	2482.79	3844.17
Transcription factor A, mitochondrial	LRFSK	1996.71	1346.75	2869.9	3484.67
Transcription factor A, mitochondrial	LRFSK	2869.99	1664.06	4982.92	4850.74
Transcription factor A, mitochondrial	LRFSK	3955.2	4234.57	5975.25	8638.17
Transcription factor A, mitochondrial	LRFSK	1515.26	1358.97	1369.93	2181.45
Transcription factor A, mitochondrial	LRFSK	774.5	821.67	1302.27	1311.11
Transcription factor A, mitochondrial	LRFSK	1300.41	884.15	1124.43	1583.67
Transcription factor A, mitochondrial	LTLL	114.69	158.48	37.77	113.82
Transcription factor A, mitochondrial	LTLL	201.09	132.94	186.59	209.92
Transcription factor A, mitochondrial	LTLL	192.95	85.86	151.74	325.05
Transcription factor A, mitochondrial	SAYNVYVAER	659	424.26	799.75	625.72
Transcription factor A, mitochondrial	SAYNVYVAER	498.2	353.68	1103.82	1244.04
Transcription factor A, mitochondrial	SAYNVYVAER	2509.42	2393.34	4058.25	5095.87
Transcription factor A, mitochondrial	SAYNVYVAER	1719.76	1687.56	3833.99	3227.23
Transcription factor A, mitochondrial	SAYNVYVAER	2149.01	2256.48	3426.11	3768.81
Transcription factor A, mitochondrial	SWEEQMIEVGR	101.8	155.1	252.21	274.52
Transcription factor A, mitochondrial	SWEEQMIEVGR	153.11	191.87	160.32	249.15
Transcription factor A, mitochondrial	SWEEQMIEVGR	357.86	389.21	445.78	603.66
Transcription factor A, mitochondrial	VYLPR	13648.57	13252.75	1997.47	2740.61
Transcription factor A, mitochondrial	VYLPR	3904.22	3313.83	1443.65	1622.89
Transcription factor A, mitochondrial	VYLPR	995.72	879.99	745.14	843.82
Transcription factor A, mitochondrial	VYLPR	5197.78	4768.5	2509.15	3073.21

Transcription factor A, mitochondrial	VYLPR	19493.74	20975.31	3448.98	3610.95
Transcription factor A, mitochondrial	VYLPR	4456.27	5368.31	2310.28	3587.92
Transcription factor A, mitochondrial	VYLPR	35819.26	35069.48	4118.84	4872.14
Aldose reductase	DYPFHEEF	2189.29	2319.33	1020.16	1556.64
Aldose reductase	EELFIVSK	1528.95	2134.48	2105.95	2419.95
Aldose reductase	LIQYCQSK	456.58	848.12	574.05	435.94
Aldose reductase	LLLNNGAK	3085.24	3163.42	1991.82	2911.01
Aldose reductase	MPILGLGTWK	520.29	617.65	305.37	387.79
Aldose reductase	NLVVIPK	3258.74	3149.65	2356.32	2840.21
Aldose reductase	SPPGQVTEAVK	2723.21	3399.65	1792.49	2119.11
Aldose reductase	TTAQVLIR	4738.96	3396.84	4330.96	4365.33
Aldose reductase	VCALLSCTSHK	1867.31	2322.14	1795.43	1830.63
Aldose reductase	VAIDVGYR	6427.71	5169.16	5202.99	6013.11
Aldose reductase	FPMQR	1265.27	1270.27	982.41	1458.42
Aldose reductase	AIAAKHNK	513.4	235.09	531.68	595.02
Aldose reductase	LWCTYHEK	2237.56	848.86	1191.33	1457.91
Aldose reductase	REELFIVSK	2938.3	3000.57	3618.01	3854.42
Aldose reductase	HIDCAHVYQNENEVGVAIQEK	598.23	683.18	979.94	1018.85
Aldose reductase	HNKTTAQVLIR	312.68	278.13	361.59	571.11
Aldose reductase	VCALLSC	281.44	279.16	478.14	127.05
Aldose reductase	AQVLIR	645.23	614.29	652	1173.64
Aldose reductase	DYPFHEEF	3196.08	4340.44	1498.93	1185.09
Aldose reductase	DYPFHEEF	3184.72	3335.16	1801.58	1276.14
Aldose reductase	DYPFHEEF	2051.14	2138.56	841.25	1083.68
Aldose reductase	DYPFHEEF	1252.56	1462.57	399.44	665.35
Aldose reductase	DYPFHEEF	1758.91	1649	491.32	672.06
Aldose reductase	DYPFHEEF	1029.76	921.63	1219.96	1068.06
Aldose reductase	DYPFHEEF	485.7	524.42	841.52	683.68
Aldose reductase	DYPFHEEF	1302.65	769.2	898.5	773.55
Aldose reductase	DYPFHEEF	1158.1	1220.76	739.16	812.29
Aldose reductase	DYPFHEEF	540.04	369.01	191.98	502.08
Aldose reductase	DYPFHEEF	800.02	427.7	165.61	377.45
Aldose reductase	DYPFHEEF	460.67	444.19	540.49	544.76
Aldose reductase	DYPFHEEF	786.19	942.7	644.9	1027.55
Aldose reductase	EELFIVSK	2923.42	2448.75	2938.13	4449.7
Aldose reductase	EGLVK	153.4	164.03	259.77	426.73
Aldose reductase	EGLVK	728.04	730.32	1416.93	1299.98
Aldose reductase	EGLVK	541.46	973.28	1092.56	951.76
Aldose reductase	EGLVK	205.13	159.21	308.71	517.72
Aldose reductase	EGLVK	281.16	307.2	368.8	771.11
Aldose reductase	EGLVK	330.72	516.99	979.64	830.05
Aldose reductase	FIVSK	905.86	1460.48	1507.68	1406.73
Aldose reductase	ILNK	3059.4	3270.14	3615.93	4312.52
Aldose reductase	ILNK	1259.27	1224.83	1231.17	1605.55
Aldose reductase	ILNK	1325.13	782.29	1817.38	2421.19
Aldose reductase	ILNK	3077.34	3518.82	4227.04	4991.5
Aldose reductase	ILNK	2975.59	3251.68	4836.32	5153.56
Aldose reductase	ILNK	2814.58	2350.52	3781.49	3872.46
Aldose reductase	ILNK	2417.43	3184.19	1443.74	1517.98
Aldose reductase	ILNK	5458.27	8180.95	5991.74	5731.38
Aldose reductase	ILNK	2618.42	2689.07	1529.65	1972.05
Aldose reductase	ILNK	1264.79	1317.9	1128.14	873.49
Aldose reductase	ILNK	12239	11238.33	15004.76	17913
Aldose reductase	ILNK	2356.27	2843.71	1808.85	1955.98
Aldose reductase	ILNK	4307.1	5106.36	6027.83	7668.98
Aldose reductase	ILNK	3115.72	4088.26	4055.47	4179.6
Aldose reductase	ILNK	1621.83	2851.96	1751.8	2219.03
Aldose reductase	LIQYCQSK	4127.02	4280.17	2557.79	3110.58
Aldose reductase	LIQYCQSK	7096.8	6617.34	5313.98	6274.65
Aldose reductase	LIQYCQSK	2111.52	2449.75	2034.25	1844.95
Aldose reductase	LIQYCQSK	2166.32	2674.31	3012.11	3190.32
Aldose reductase	LIRF	611.59	533.85	711.67	1287.87
Aldose reductase	LLLNNGAK	4885.17	4696.27	2014.92	3011.21
Aldose reductase	LLLNNGAK	1925.06	2573.87	1737.24	2081.75

Aldose reductase	LLLNNGAK	10831.74	9847.43	8701.19	6678.52
Aldose reductase	LLLNNGAK	13577.88	15130.07	6463.88	9096.93
Aldose reductase	LLLNNGAK	10855.14	11790.64	7275.73	8830.18
Aldose reductase	LLLNNGAK	10831.74	9847.43	8701.19	6678.52
Aldose reductase	LLLNNGAK	10855.14	11790.64	7275.73	8830.18
Aldose reductase	LLLNNGAK	4424.17	4071.53	5726.02	5180.4
Aldose reductase	LLLNNGAK	6011.16	7173.67	8421.37	9444.99
Aldose reductase	LLLNNGAK	6011.16	7173.67	8421.37	9444.99
Aldose reductase	LLLNNGAK	13577.88	15130.07	6463.88	9096.93
Aldose reductase	LLLNNGAK	4885.17	4696.27	2014.92	3011.21
Aldose reductase	LLLNNGAK	4424.17	4071.53	5726.02	5180.4
Aldose reductase	LLLNNGAK	3085.24	3163.42	1991.82	2911.01
Aldose reductase	LSDLK	1103.46	1300.11	1588.59	863.17
Aldose reductase	LSDLK	1090.73	1281.11	2415.67	2601.54
Aldose reductase	LSDLK	1116.61	1064.15	1511.15	2352
Aldose reductase	LSDLK	16862.64	17198.44	38064.49	49856.14
Aldose reductase	LTQEK	1360.56	1537.25	1468.89	1957.78
Aldose reductase	LWCTYHEK	1200.12	1324.56	1089.11	1907.21
Aldose reductase	LWCTYHEK	207.13	174.64	176.14	183.87
Aldose reductase	MPILGLGTWK	1269.72	1256.54	741.35	1045.9
Aldose reductase	MPILGLGTWK	205.58	114.81	109.61	271.94
Aldose reductase	MPILGLGTWK	356.82	172.95	136.85	194.84
Aldose reductase	MPILGLGTWK	538.01	462.96	339.34	545.61
Aldose reductase	MPILGLGTWK	330.92	497.76	334.23	482.38
Aldose reductase	NLVVIPK	2583.38	2426.91	2126.69	2172.01
Aldose reductase	NLVVIPK	2992.48	3817.36	2376.19	2548.81
Aldose reductase	NLVVIPK	15453.47	17804.41	9370.04	12770.19
Aldose reductase	NLVVIPK	958.61	1036.46	1279.04	974.54
Aldose reductase	NLVVIPK	2452.83	3090.94	2628.91	2329.32
Aldose reductase	NLVVIPK	1357.04	1246.16	1466.47	1474.88
Aldose reductase	NLVVIPK	2124.11	1844.26	1195.89	2037.21
Aldose reductase	NLVVIPK	877.5	1070.71	1105.33	936.45
Aldose reductase	NLVVIPK	2122.53	1788.1	2118.33	2542.44
Aldose reductase	NLVVIPK	1400.21	1129.87	1422.59	859.33
Aldose reductase	NLVVIPK	1061.06	808.25	969.2	1035.39
Aldose reductase	NLVVIPK	1773.88	2191.01	1922.53	2454.01
Aldose reductase	NLVVIPK	2257.54	1746.9	1693.53	2002.6
Aldose reductase	NLVVIPK	509.94	470.25	347.53	261.49
Aldose reductase	NLVVIPK	764.33	974.75	1426.44	685.33
Aldose reductase	NLVVIPK	561.04	527.59	408.81	632.12
Aldose reductase	NLVVIPK	482.34	643.48	336.6	262.81
Aldose reductase	NLVVIPK	797.01	938.33	502.19	356.76
Aldose reductase	NLVVIPK	535.33	515	647.02	455.15
Aldose reductase	NLVVIPK	414.56	319.32	556.69	381.79
Aldose reductase	NLVVIPK	624.96	382.61	484.58	307.05
Aldose reductase	NLVVIPK	2858.35	3452.52	2085.95	2551.96
Aldose reductase	SPPGQVTEAVK	6384.36	7111.46	3215.75	4026.22
Aldose reductase	SPPGQVTEAVK	6253.69	7467.74	4527.93	4750.72
Aldose reductase	SPPGQVTEAVK	2940.58	3200.32	2452.15	2787.83
Aldose reductase	SPPGQVTEAVK	6777.65	6977.98	6090.11	5667.92
Aldose reductase	SPPGQVTEAVK	8406.69	8266.97	4707.12	5845.68
Aldose reductase	SPPGQVTEAVK	5152.64	5504.63	5940.16	6750.85
Aldose reductase	SPPGQVTEAVK	5871.04	6442.85	5353.13	6667.23
Aldose reductase	SPPGQVTEAVK	1318.27	1247.58	2176.69	1782.42
Aldose reductase	SPPGQVTEAVK	1707.7	1921.22	1734.26	2893.26
Aldose reductase	SPPGQVTEAVK	1438.88	1459.09	2069.76	2200.44
Aldose reductase	TLSDLK	986.02	881.18	1040.6	1190.56
Aldose reductase	TLSDLK	1928.35	2036.58	2718.97	2882.09
Aldose reductase	TTAQVLIR	2177.85	2599.23	3067.19	3236.72
Aldose reductase	TTAQVLIR	12169.03	12193.54	7902.12	10352.37
Aldose reductase	TTAQVLIR	2127.92	2106.74	2452.97	2388.72
Aldose reductase	TTAQVLIR	3432.03	3050.3	1901.98	2612.74
Aldose reductase	TTAQVLIR	2576.36	2162.79	2664.58	3117.97
Aldose reductase	TTAQVLIR	1819.79	1379.08	2078.23	2729.94

Aldose reductase	TTAQVLIR	1037.16	1360.65	2081.81	2115.97
Aldose reductase	VAIDVGYR	14168.46	14653.9	14769.45	14927.15
Aldose reductase	VAIDVGYR	2977.44	2005.82	3215.16	4275.66
Aldose reductase	VAIDVGYR	43109.25	43025.74	23501.78	27315.28
Aldose reductase	VAIDVGYR	11054.42	9442.27	12289.48	14939.11
Aldose reductase	VCALLSC	383.9	369.37	746.71	139.75
Aldose reductase	VCALLSCTSHK	1057.13	877.19	1296.7	645.04
Aldose reductase	VCALLSCTSHK	1612.32	1344.06	2283.98	2517.05
Aldose reductase	VVIPK	4659.01	6083.08	4294.28	5569.28
Aldose reductase	VVIPK	6031.82	7047.29	6991.05	8216.71
Aldose reductase	VVIPK	3661.58	3137.71	4396.13	4941.47
Aldose reductase	VVIPK	10341.22	8947.54	10174.57	11018.72
Aldose reductase	VVIPK	9190.29	9678.51	8977.17	11470.38
Aldose reductase	VVIPK	3186.44	2739.64	3382.57	4214.1
Surfeit locus protein 2	QGVEYVPACLVHR	512.89	497.89	831.14	1076.54
Surfeit locus protein 2	LPELQVYTR	435.35	392.81	674.96	647.33
Surfeit locus protein 2	LGSLK	6356.18	7980.2	3642.09	3873.59
Surfeit locus protein 2	LGSLK	1023.54	1004.19	2424.74	2389.86
Surfeit locus protein 2	LGSLK	6682.11	8544.92	2065.25	4492.65
Surfeit locus protein 2	LGSLK	6212.44	5798.33	2687.24	3461.96
Surfeit locus protein 2	LGSLK	3330.6	3497.26	5748.44	6514.95
Surfeit locus protein 2	LGSLK	1080.35	1136.45	2269.27	1460.74
Surfeit locus protein 2	LPGDVRAFLR	1222.7	689.24	1317.26	1974.84
Surfeit locus protein 2	QGVEYVPACLVHR	644.26	178.26	310.14	393.96
Surfeit locus protein 2	QGVEYVPACLVHR	676.74	266.94	910.72	515.89
Surfeit locus protein 2	QLGSLK	5145.29	2614.06	8745.64	4022.5
Surfeit locus protein 2	RAFLR	2273.81	1735.66	1870.69	2133.95
Serine/threonine-protein kinase MARK2	NSATSADEQPHIGNYR	89	143.54	233.01	225.01
Serine/threonine-protein kinase MARK2	TTPTPSTNSVLSTSTNR	357.19	454.87	444.58	506.77
Serine/threonine-protein kinase MARK2	VPVASPSAHNISSSGGAPDR	100.62	271.33	251.65	475.47
Serine/threonine-protein kinase MARK2	EEIQDSLVGQR	127.28	190.71	312.22	344.35
Serine/threonine-protein kinase MARK2	VPASPLPGLER	381.95	562.25	680.54	573.75
Serine/threonine-protein kinase MARK2	IANELKL	1763.96	1043.96	1201.19	1227.92
Serine/threonine-protein kinase MARK2	IPFYMSTDCENLLK	332.38	353.31	453.35	361.57
Serine/threonine-protein kinase MARK2	FLILNPSK	180.02	87.01	142.65	198.7
Serine/threonine-protein kinase MARK2	EEIQDSLVGQR	204.85	186.92	215.27	142.23
Serine/threonine-protein kinase MARK2	ENLLK	999.94	888.79	1889.7	2038.79
Serine/threonine-protein kinase MARK2	ENLLK	1203	720.98	1415.15	2014.67
Serine/threonine-protein kinase MARK2	ENLLK	380.23	731.62	446.44	716.79
Serine/threonine-protein kinase MARK2	EVAVK	541.46	973.28	1092.56	951.76
Serine/threonine-protein kinase MARK2	IANELKL	1981.46	1469.88	2047.41	2623.95
Serine/threonine-protein kinase MARK2	IANELKL	1774.55	1805.83	2516.73	2219.34
Serine/threonine-protein kinase MARK2	IANELKL	30848.87	35282.73	2264.81	3591.41
Serine/threonine-protein kinase MARK2	IANELKL	21733.49	23202.9	1428.84	2562.22
Serine/threonine-protein kinase MARK2	IANELKL	47661.21	49897.16	786.09	2683.3
Serine/threonine-protein kinase MARK2	IANELKL	2665.49	2718.95	3176.73	2726.96
Serine/threonine-protein kinase MARK2	IANELKL	3322.55	3228.11	953.92	719.21
Serine/threonine-protein kinase MARK2	IANELKL	14520.77	17400.01	1835.78	2128.39
Serine/threonine-protein kinase MARK2	IANELKL	2582.63	2075.33	1276.81	1927.3
Serine/threonine-protein kinase MARK2	IANELKL	19547.32	19298.13	634.25	2656.79
Serine/threonine-protein kinase MARK2	IANELKL	31566.22	33132.98	1671	3414.74
Serine/threonine-protein kinase MARK2	IANELKL	10562.66	12602.16	3522.41	5318.37
Serine/threonine-protein kinase MARK2	IANELKL	1124	972.62	745.74	797.41
Serine/threonine-protein kinase MARK2	IANELKL	23795.87	26252.41	1634.26	3374.62
Serine/threonine-protein kinase MARK2	IANELKL	2331.47	1783.78	1087.49	1328.42
Serine/threonine-protein kinase MARK2	IANELKL	2463.29	1951.25	671.24	987.84
Serine/threonine-protein kinase MARK2	IANELKL	1438.56	1384.45	1454.58	758.28
Serine/threonine-protein kinase MARK2	IFSK	3056.35	3570.66	4399.12	4841.04
Serine/threonine-protein kinase MARK2	IFSK	1389.34	1672.03	2302.68	2188.84
Serine/threonine-protein kinase MARK2	IIDK	1325.13	782.29	1817.38	2421.19
Serine/threonine-protein kinase MARK2	IIDK	1259.27	1224.83	1231.17	1605.55
Serine/threonine-protein kinase MARK2	IIDK	11494.35	11829.41	19877.02	24045.93
Serine/threonine-protein kinase MARK2	IIDK	8107.16	8072.27	13308.71	15925.9
Serine/threonine-protein kinase MARK2	IIDK	1686.04	2252.31	2137.37	2898.31

Serine/threonine-protein kinase MARK2	IIDK	1799.72	1871.91	3038.68	3494.33
Serine/threonine-protein kinase MARK2	IIDK	2542.42	2325.42	3467.44	4284.44
Serine/threonine-protein kinase MARK2	IIDK	2044.79	2765.98	3071.91	3593.15
Serine/threonine-protein kinase MARK2	IIDK	11419.16	12769	18247.56	23009.46
Serine/threonine-protein kinase MARK2	IIDK	4581.13	5798.65	7107.07	10954.17
Serine/threonine-protein kinase MARK2	IIDK	1817.73	1794.65	2751.75	3695.78
Serine/threonine-protein kinase MARK2	IIDK	6734.04	6925.33	10184.37	13283.95
Serine/threonine-protein kinase MARK2	IIDK	2816.96	2116	3299.59	5206.11
Serine/threonine-protein kinase MARK2	IIDK	6381.53	5263.29	12843.03	15057.89
Serine/threonine-protein kinase MARK2	IIDK	1634.62	1694.21	1902.07	2653.86
Serine/threonine-protein kinase MARK2	IIDK	2610.13	3614.15	4202.4	5576.11
Serine/threonine-protein kinase MARK2	IIDK	1520.78	1558.44	3184.54	2618.89
Serine/threonine-protein kinase MARK2	IIDK	8800.84	9644.57	14104.83	17354.68
Serine/threonine-protein kinase MARK2	IIDK	994.06	1469.88	1517.2	2486.21
Serine/threonine-protein kinase MARK2	ILTGK	1237.32	946.94	1760.25	1809.14
Serine/threonine-protein kinase MARK2	ITLK	1465.44	1175.18	2133.76	2134.03
Serine/threonine-protein kinase MARK2	ITLK	2032.65	1625.52	2511.72	3093.68
Serine/threonine-protein kinase MARK2	ITLK	6851.05	8875.36	12546.63	18495.69
Serine/threonine-protein kinase MARK2	ITLK	9475.64	11060.57	17223.33	22883.07
Serine/threonine-protein kinase MARK2	ITLK	11098.45	12432.88	20943.33	25785.42
Serine/threonine-protein kinase MARK2	ITLK	2987.22	3060.11	4110.36	4527.78
Serine/threonine-protein kinase MARK2	ITLK	8161.22	8475.75	11768.29	12932.92
Serine/threonine-protein kinase MARK2	ITLK	3087.61	3461.58	4680.58	6551.89
Serine/threonine-protein kinase MARK2	ITLK	5883.14	6843.59	12399.68	15763.23
Serine/threonine-protein kinase MARK2	ITLK	6196.24	6974.04	13256.44	14651.59
Serine/threonine-protein kinase MARK2	ITLK	8833.79	6920.92	9303.18	12602.44
Serine/threonine-protein kinase MARK2	ITLK	10036.18	11531.95	17595.63	21037.98
Serine/threonine-protein kinase MARK2	ITLK	1293.32	2009.6	2763.76	4385.45
Serine/threonine-protein kinase MARK2	ITLK	5768.95	4827.06	8848.78	10292.34
Serine/threonine-protein kinase MARK2	ITLK	5226.47	6720.84	6463.66	9144.31
Serine/threonine-protein kinase MARK2	ITLK	5559.77	6026.14	8012.02	9833.91
Serine/threonine-protein kinase MARK2	ITLK	11357.95	13390.3	20715.65	25330.2
Serine/threonine-protein kinase MARK2	ITLK	3511.51	3389.71	4235.7	6499.2
Serine/threonine-protein kinase MARK2	ITLK	2408.07	2513.67	2679.12	3761.43
Serine/threonine-protein kinase MARK2	ITLK	1554.97	1584.93	1376.55	1981.94
Serine/threonine-protein kinase MARK2	ITLK	6275.11	6415.87	6323.86	7462.86
Serine/threonine-protein kinase MARK2	ITLK	2869	2293.12	3549.31	5310.84
Serine/threonine-protein kinase MARK2	ITLK	1402.72	2052.61	2469.56	3793.38
Serine/threonine-protein kinase MARK2	ITLK	3464.79	3962.55	5377.96	6842.07
Serine/threonine-protein kinase MARK2	ITLK	9629.82	11573.31	16506.83	20170.67
Serine/threonine-protein kinase MARK2	ITLK	2220.2	2883.35	2155.12	3678.62
Serine/threonine-protein kinase MARK2	IVKL	1071.45	1590.3	2510.33	3331.23
Serine/threonine-protein kinase MARK2	IVKL	2944.6	2377.74	4777.68	5411.7
Serine/threonine-protein kinase MARK2	IVKL	2298.42	2602.15	3385.07	5172.57
Serine/threonine-protein kinase MARK2	IVKL	3592.95	2897	5810.44	6800.76
Serine/threonine-protein kinase MARK2	LEQIMK	999.93	1315.06	1228.43	1775.26
Serine/threonine-protein kinase MARK2	LEQIMK	662.14	752.56	398.88	515.18
Serine/threonine-protein kinase MARK2	NIVK	127.27	191.64	266.02	165.17
Serine/threonine-protein kinase MARK2	NLLK	540.63	204.34	676.13	887.3
Serine/threonine-protein kinase MARK2	NLLK	359.27	249.9	363.8	1049.81
Serine/threonine-protein kinase MARK2	NLLK	202.84	385.47	613.68	578.56
Serine/threonine-protein kinase MARK2	NSATSADEQPHIGNYR	192.18	162.34	201.21	233.73
Serine/threonine-protein kinase MARK2	NSATSADEQPHIGNYR	167.4	57.18	387.47	191.88
Serine/threonine-protein kinase MARK2	QNLK	1331.98	1275.66	2386.22	2606.35
Serine/threonine-protein kinase MARK2	QNLK	1114.11	1205	1892.83	2006.06
Serine/threonine-protein kinase MARK2	TTPTPSTNSVLSTSTNR	4253.4	4879.22	6556.69	7601.61
Serine/threonine-protein kinase MARK2	TTPTPSTNSVLSTSTNR	301.64	837.07	677.56	635.74
Serine/threonine-protein kinase MARK2	VPASPLPGLER	409.18	425.28	388.06	940.8
Serine/threonine-protein kinase MARK2	VPASPLPGLER	544.04	612.16	809.13	925.81
Transmembrane and ubiquitin-like domain-containing protein 1	FLNDSEQVAR	763.65	723.53	983.93	1106.67
Transmembrane and ubiquitin-like domain-containing protein 1	FLNDSEQVAR	823.35	1207.6	1180.97	1882.15
Transmembrane and ubiquitin-like domain-containing protein 1	FLNDSEQVAR	462.44	268.87	323.92	616.75
Transmembrane and ubiquitin-like domain-containing protein 1	IGSLK	6356.18	7980.2	3642.09	3873.59

Transmembrane and ubiquitin-like domain-containing protein 1	IGSLK	6682.11	8544.92	2065.25	4492.65
Transmembrane and ubiquitin-like domain-containing protein 1	IGSLK	1023.54	1004.19	2424.74	2389.86
Transmembrane and ubiquitin-like domain-containing protein 1	IGSLK	7326.05	8542.57	9555.7	11849.41
Transmembrane and ubiquitin-like domain-containing protein 1	IGSLK	6212.44	5798.33	2687.24	3461.96
Transmembrane and ubiquitin-like domain-containing protein 1	IGSLK	4918.09	6230.62	3546.17	4981.69
Transmembrane and ubiquitin-like domain-containing protein 1	IGSLK	3330.6	3497.26	5748.44	6514.95
Transmembrane and ubiquitin-like domain-containing protein 1	IGSLK	1080.35	1136.45	2269.27	1460.74
Transmembrane and ubiquitin-like domain-containing protein 1	LRLK	3814.3	3262.06	4759.66	4438.43
Transmembrane and ubiquitin-like domain-containing protein 1	VTVLF	152.87	109.27	98.32	128.99
Hypoxia-inducible factor 1-alpha inhibitor	EEAGALGPAWDESQLR	335.22	72.92	270.65	243.55
Hypoxia-inducible factor 1-alpha inhibitor	AATAAEAVASGSGEPR	279.14	506.66	722.06	640.53
Hypoxia-inducible factor 1-alpha inhibitor	AATAAEAVASGSGEPR	818.62	504.9	693.37	1581.21
Hypoxia-inducible factor 1-alpha inhibitor	AATAAEAVASGSGEPR	759.12	958	1369.96	1024.76
Hypoxia-inducible factor 1-alpha inhibitor	DFSV	1502.4	1459.39	2566.55	3024.36
Hypoxia-inducible factor 1-alpha inhibitor	EEAGALGPAWDESQLR	413.34	546.27	770.65	855.92
Hypoxia-inducible factor 1-alpha inhibitor	EFVEK	1350.11	2569.58	812.22	1303.88
Hypoxia-inducible factor 1-alpha inhibitor	EFVEK	7972.18	8110.09	5336.64	7017.85
Hypoxia-inducible factor 1-alpha inhibitor	EFVEK	6530.19	7277.48	4787.9	5370.37
Hypoxia-inducible factor 1-alpha inhibitor	FAQIK	1904.53	1623.93	2315.05	1269.4
Hypoxia-inducible factor 1-alpha inhibitor	FAQIK	2591.63	2463.16	2750.06	2395.08
Hypoxia-inducible factor 1-alpha inhibitor	FAQIK	4575.5	3269.45	2721.44	3363.76
Hypoxia-inducible factor 1-alpha inhibitor	FAQIK	1894.75	1951.68	1244.07	1874.41
Hypoxia-inducible factor 1-alpha inhibitor	NTMIK	463.15	198.91	1228.71	141.37
Vitronectin	GQYCYELDEK	717.84	674.84	528.68	1101.98
Vitronectin	AVRPGYPK	596.89	390.26	900.61	952.03
Vitronectin	ILAL	196.22	188.4	226.96	250.42
Vitronectin	ILAL	75.78	172.69	115.6	69.95
Vitronectin	VYFFK	1130.71	1160.74	883.52	1099.53
Vitronectin	VYFFK	1462.06	870.29	898.42	940.1
Vitronectin	VYFFK	990.64	743.71	589.99	1003.86
Vitronectin	VYFFK	1010.29	717.9	624.52	554.94
Vitronectin	VYFFK	945.97	902.08	632.42	730.42
Vitronectin	VYFFK	332.92	299.67	551.33	251.11
Vitronectin	VYFFK	777.35	646	832.95	544.13
Vitronectin	VYFFK	848.72	616.21	662.19	636
Vitronectin	VYFFK	1109.94	1191.79	593.73	713.78
All-trans retinoic acid-induced differentiation factor	VAFYCK	1087.21	1096.96	1644.73	1245.52
All-trans retinoic acid-induced differentiation factor	GDLANTFR	2439.54	2596.04	3648.87	3974.72
All-trans retinoic acid-induced differentiation factor	LGLDL	460.61	449.76	1116.4	1020.61
All-trans retinoic acid-induced differentiation factor	LLAL	196.22	188.4	226.96	250.42
All-trans retinoic acid-induced differentiation factor	LLAL	75.78	172.69	115.6	69.95
All-trans retinoic acid-induced differentiation factor	QNLSK	1618.69	2523.3	1266.18	2347.16
All-trans retinoic acid-induced differentiation factor	QNLSK	7313.04	7380.36	3957.27	3509.56
All-trans retinoic acid-induced differentiation factor	QNLSK	231.49	107.54	214.75	323.87
All-trans retinoic acid-induced differentiation factor	QNLSK	1028.13	1720.45	1284.23	1423.38
F-box only protein 9	TDAILLGHYR	1550.41	2035.56	2079.88	2600.87
F-box only protein 9	VFAVITK	736.82	501.23	483.27	695.98
F-box only protein 9	AIKF	2230.81	2047.23	2573.04	2653.27
F-box only protein 9	AVITK	3009.15	4191.8	5547.77	5900.88
F-box only protein 9	AVITK	4610.26	4941.44	6745.51	6877.07
F-box only protein 9	ELFLK	1147.73	1181.67	1541	2287.84
F-box only protein 9	ELFLK	255.87	252.53	612.43	432.7
F-box only protein 9	ELFLK	6520.37	6439.62	685.95	1228.61
F-box only protein 9	ELFLK	868.14	718.71	1265.36	1637.86
F-box only protein 9	ELFLK	3981.09	3378.83	392.91	538.85
F-box only protein 9	ELFLK	5703.03	5382.34	451.18	623.75
F-box only protein 9	ESVLK	384.25	547.34	877.47	803.1
F-box only protein 9	ESVLK	260.95	282.07	409.37	337.03
F-box only protein 9	FEIDK	2432.72	3375.8	1696.48	2205.95
F-box only protein 9	IEFK	5980.65	6890.07	700.28	851.97
F-box only protein 9	IEFK	2365.31	3014.86	702.85	1203.49
F-box only protein 9	LFLK	2800.73	2655.93	2713.72	2747.15

F-box only protein 9	LFLK	848.14	885.96	2426.69	2268.47
F-box only protein 9	LFLK	1293.11	2456.52	2411.93	2123.8
F-box only protein 9	LFLK	2470.97	2256.62	2983.58	3778.41
F-box only protein 9	LFLK	1295.7	1136.43	2013.16	2203.91
F-box only protein 9	LFLK	1376.94	1088.83	1995.35	2068.54
F-box only protein 9	LFLK	3125.37	3879.87	3903.38	4277.33
F-box only protein 9	LFLK	562.75	571.7	1039.53	1023.44
F-box only protein 9	LFLK	1216.57	1507.36	1879.53	2265.18
F-box only protein 9	YEAIK	1208.75	1429.12	2001.89	2221.1
F-box only protein 9	YIFR	1666.34	2177.73	2122.15	2490.05
Squalene synthase	ANSMGLFLQK	696.56	542.7	1558.17	1430.33
Squalene synthase	CLGHPEEFYNLVR	359.08	268.47	722.4	553.67
Squalene synthase	MDQDSLSSSLK	1612.55	1747.28	3772.43	4898.22
Squalene synthase	TQNLPNCQLISR	458.16	691.82	1012.57	1460.86
Squalene synthase	YQTVIADICR	563.75	366.79	215.26	586.72
Squalene synthase	FMESK	1473.05	1806.05	2440.55	3131.28
Squalene synthase	MEFVK	1243.36	1094.87	2079.76	2187.62
Squalene synthase	ANSMGLFLQK	891.23	459.25	1994.34	2327.44
Squalene synthase	ANSMGLFLQK	824.32	793.28	1726.17	1763.98
Squalene synthase	ANSMGLFLQK	179.27	160.97	316.32	266.79
Squalene synthase	ANSMGLFLQK	623.36	222.23	764.68	968.58
Squalene synthase	CLGHPEEFYNLVR	385.41	220.28	456.74	310.91
Squalene synthase	CLGHPEEFYNLVR	446.28	270.02	325.96	548.73
Squalene synthase	CLGHPEEFYNLVR	571.43	459.16	617.39	942.65
Squalene synthase	EFLDK	29470.13	33626.17	1498.56	2512.54
Squalene synthase	EFLDK	7395.71	9212.98	1561.97	1459.32
Squalene synthase	EFLDK	1350.11	2569.58	812.22	1303.88
Squalene synthase	EFLDK	7015.5	9010.84	1252.65	1811.17
Squalene synthase	EFVK	708.78	612.34	781.34	623.17
Squalene synthase	EFVK	1101.33	977.95	1150.01	1409.18
Squalene synthase	FLDK	2467.9	2133.81	4495.56	5351.67
Squalene synthase	FLDK	1675.88	1128.61	2070.47	2141.71
Squalene synthase	FLQK	3312.82	3551.44	3186.68	3370.69
Squalene synthase	FLQK	2051.3	3399.92	2911.57	3980.78
Squalene synthase	FLQK	6492.49	5363.91	7352.81	7479.64
Squalene synthase	FMESK	1676.86	2414.6	2972.57	3210.72
Squalene synthase	IGGK	272.38	216.83	403.38	919.32
Squalene synthase	IPDSDPSSSK	961.3	1514.91	1005.22	1426.65
Squalene synthase	ISVEK	1540.16	1558.88	1655.49	2261.64
Squalene synthase	LSSSLK	6305.55	6918.32	9229.28	9757.53
Squalene synthase	LSSSLK	842.46	916.03	215.13	268.73
Squalene synthase	LSSSLK	2600.52	3180.77	3548.45	4035.74
Squalene synthase	LSSSLK	822.08	695.16	524.43	484.92
Squalene synthase	LSSSLK	3542.88	3375.58	564.06	463.31
Squalene synthase	LSSSLK	1303.2	927.49	1736.49	1706.18
Squalene synthase	LSSSLK	5904.56	5578.24	7309	9026.91
Squalene synthase	MDQDSLSSSLK	2132.65	1958.31	4459.03	3652.79
Squalene synthase	TQNLPNCQLISR	460.39	472.25	589.85	701.19
Squalene synthase	TQNLPNCQLISR	203.81	289.52	250.22	476.38
Squalene synthase	TQNLPNCQLISR	773.46	924.08	1080.76	1529.11
Squalene synthase	TQNLPNCQLISR	332.16	374.98	658.78	410.77
Squalene synthase	VMPK	1180.91	1413.31	1556.35	2003.9
Squalene synthase	YLVLR	2851.67	2196.8	2845.35	4536.69
Squalene synthase	YQTVIADICR	884.4	388.83	502.63	844.63
Integrator complex subunit 10	SLFETLPGR	435.75	352.65	745.29	712.6
Integrator complex subunit 10	YIIEGLTEK	2296.8	2017.11	3927.7	3760.85
Integrator complex subunit 10	SAQGDCEFLVQR	410.23	321.33	473.39	682.83
Integrator complex subunit 10	EFLDK	29470.13	33626.17	1498.56	2512.54
Integrator complex subunit 10	EFLDK	7395.71	9212.98	1561.97	1459.32
Integrator complex subunit 10	EFLDK	1350.11	2569.58	812.22	1303.88
Integrator complex subunit 10	EFLDK	7015.5	9010.84	1252.65	1811.17
Integrator complex subunit 10	EIDR	103.21	15.61	126.25	44.94
Integrator complex subunit 10	EMLLK	693.2	768.54	983.92	629.55
Integrator complex subunit 10	EMLLK	621.52	403.4	745.44	552.25

Integrator complex subunit 10	FLDK	2467.9	2133.81	4495.56	5351.67
Integrator complex subunit 10	FLDK	1675.88	1128.61	2070.47	2141.71
Integrator complex subunit 10	LFLK	2800.73	2655.93	2713.72	2747.15
Integrator complex subunit 10	LFLK	848.14	885.96	2426.69	2268.47
Integrator complex subunit 10	LFLK	1293.11	2456.52	2411.93	2123.8
Integrator complex subunit 10	LFLK	2470.97	2256.62	2983.58	3778.41
Integrator complex subunit 10	LFLK	1295.7	1136.43	2013.16	2203.91
Integrator complex subunit 10	LFLK	1376.94	1088.83	1995.35	2068.54
Integrator complex subunit 10	LFLK	3125.37	3879.87	3903.38	4277.33
Integrator complex subunit 10	LFLK	562.75	571.7	1039.53	1023.44
Integrator complex subunit 10	LFLK	1216.57	1507.36	1879.53	2265.18
Integrator complex subunit 10	LLLLLR	451.94	349.07	384.46	330.16
Integrator complex subunit 10	LLLLLR	229.82	273.13	273.95	291.83
Integrator complex subunit 10	LLLLLR	278.69	338.3	328.78	362.21
Integrator complex subunit 10	LLLLLR	200.42	199.21	155.7	240.44
Integrator complex subunit 10	LLLLR	1750.41	1105.72	1972.26	1170.05
Integrator complex subunit 10	LLLLR	229.51	303.98	717.46	474.46
Integrator complex subunit 10	LLLLR	1332.93	2564.35	5070.19	1069.01
Integrator complex subunit 10	LLLLR	639	592	926.35	1025
Integrator complex subunit 10	LLLLR	469.82	390.94	641.42	692.34
Integrator complex subunit 10	LLLLR	562.7	682.61	659.6	1176.4
Integrator complex subunit 10	LLLR	386.99	489.76	684.01	680.12
Integrator complex subunit 10	LLLR	769.62	771.66	1081.49	1259.46
Integrator complex subunit 10	LLLR	864.03	699.71	1475.57	835.82
Integrator complex subunit 10	LLLR	912.1	950.18	1143.27	1280.65
Integrator complex subunit 10	LLLR	556.67	959.36	927.48	1147.92
Integrator complex subunit 10	LLLR	1415.65	1626.01	1823.04	1846.6
Integrator complex subunit 10	LLLR	279.39	481.67	627.51	552.92
Integrator complex subunit 10	LLLR	487.03	393.55	449.11	438.94
Integrator complex subunit 10	LLYK	2343.22	3069.36	2768.3	3350.47
Integrator complex subunit 10	LLYK	1494.13	1319.79	2002.86	1611.01
Integrator complex subunit 10	LLYK	1695.62	1412.87	1872.62	2723.39
Integrator complex subunit 10	LWLR	1901.95	2198.09	3549.51	5049.93
Integrator complex subunit 10	MLIK	3797.99	4660.04	5610.85	7416.45
Integrator complex subunit 10	MLIK	1569.66	1197.98	2060.11	1919.83
Integrator complex subunit 10	MLLK	3797.99	4660.04	5610.85	7416.45
Integrator complex subunit 10	MLLK	1569.66	1197.98	2060.11	1919.83
Integrator complex subunit 10	MLLLL	105.71	87.7	114.57	102.15
Integrator complex subunit 10	MLLLL	138.51	144.93	195.45	216.9
Integrator complex subunit 10	MLLLL	73.75	53.94	93.46	92.57
Integrator complex subunit 10	NLFLK	587.9	533.46	764.83	386.79
Integrator complex subunit 10	NLFLK	947.18	994.66	1633.22	1815.68
Integrator complex subunit 10	SLFETLPGR	206.4	33.96	117.01	303.29
Integrator complex subunit 10	SLFETLPGR	435.81	346.85	393.53	674.16
Integrator complex subunit 10	YGDILHR	358.42	333.41	916.52	1241.93
Formin-like protein 2	AQGPAIDLSSSK	1202.46	986.4	1531.9	1183.92
Formin-like protein 2	QQQELIAELR	435.6	368.35	607.75	505.88
Formin-like protein 2	GSNLPSPVGNSVSR	1154.75	1444.79	1424.71	1994.72
Formin-like protein 2	IHELEK	13613.97	13479.75	1746.26	2478.26
Formin-like protein 2	VTLLEANR	1680.33	1539.99	2714.43	2957.36
Formin-like protein 2	IAQDAFDDVVK	204.34	237.14	286.41	241.4
Formin-like protein 2	KWELICDQER	789.28	1382.68	1049.18	1717.09
Formin-like protein 2	EFILNNEGK	2178.01	2689.34	3875.14	4591.54
Formin-like protein 2	NLAITLR	760.89	1740.5	2016.5	1406.46
Formin-like protein 2	AIHVFDLK	587.81	542.27	1126.88	967.18
Formin-like protein 2	NPPHTYIQK	749.71	717.6	1009.26	1170.93
Formin-like protein 2	FLPTENEVK	1273.54	1091.62	1757.7	2256.4
Formin-like protein 2	QSTLEK	1175.78	643.47	1519.32	579.02
Formin-like protein 2	TLKNSR	359.28	248.78	419.13	750.29
Formin-like protein 2	VLLDVK	2497.29	2422.19	3899.09	6358.26
Formin-like protein 2	ARLLR	652.99	911.35	1673.27	1931.99
Formin-like protein 2	ARLLR	1080.13	732.01	1638.98	1877.24
Formin-like protein 2	EIFK	2341.82	4378.22	2760.69	5011.31
Formin-like protein 2	EIFK	812.14	826.51	925.19	1053.71

Formin-like protein 2	EIFK	944.52	832.03	698.7	1348.21
Formin-like protein 2	EIFK	613.05	814.67	804	1196.58
Formin-like protein 2	ELLAA	180.45	257.22	444.12	348.36
Formin-like protein 2	FDLK	1243.81	1369.26	1430.49	1988.22
Formin-like protein 2	GENPK	921.58	652.16	1063.56	1202.54
Formin-like protein 2	GENPK	1129.65	840.08	1217.73	1481.08
Formin-like protein 2	GLAAVK	1596.32	1755.28	1095.8	1390.3
Formin-like protein 2	GSNLPSPVGNSVSR	1004.97	1668.4	2241.9	2070.87
Formin-like protein 2	GSNLPSPVGNSVSR	803.96	1846.91	1541.6	1717.31
Formin-like protein 2	IAELR	3086.24	5085.77	6680.64	9480.31
Formin-like protein 2	IAELR	4316.75	2982.25	7344.61	7849.54
Formin-like protein 2	IAELR	2604.82	2223.75	4012.92	4068.83
Formin-like protein 2	IAELR	912.31	1035.72	1378.93	1830.16
Formin-like protein 2	IHELEK	16461.74	16593.67	1365.73	2398.48
Formin-like protein 2	IHELEK	9411.09	10736.22	1536.96	1621.45
Formin-like protein 2	IHELEK	16814.38	19732.11	1735.49	2350.08
Formin-like protein 2	IHELEK	717.53	599.08	234.75	295.28
Formin-like protein 2	IHELEK	16742.99	16253.36	2435.27	1810.95
Formin-like protein 2	IHELEK	18432.73	17608.64	2497.19	2959.35
Formin-like protein 2	IHELEK	1910.76	2497.88	2516.81	1712.13
Formin-like protein 2	IHELEK	1728.3	2226.3	1513.86	984.89
Formin-like protein 2	ILAL	196.22	188.4	226.96	250.42
Formin-like protein 2	ILAL	75.78	172.69	115.6	69.95
Formin-like protein 2	IPQK	730.39	1562.49	1678.65	3257.86
Formin-like protein 2	ITVLK	967.35	810.9	490.09	723.21
Formin-like protein 2	IVELEK	1002.8	1245.06	1755.47	2852.44
Formin-like protein 2	LDKL	2055.16	1635.69	2617.23	3027.78
Formin-like protein 2	LDVK	809.25	1005.58	960.41	1254.65
Formin-like protein 2	LDVK	984.03	651.82	851.56	1355.47
Formin-like protein 2	LDVK	1530.77	891.31	2692.25	3075.59
Formin-like protein 2	LLDTK	2426.96	3200	4828.71	4852
Formin-like protein 2	LLDTK	1161.53	1201.1	2124.68	2616.16
Formin-like protein 2	LLDVK	1102.04	1121.04	877.82	1658.6
Formin-like protein 2	LNNEGK	995.91	755.13	1335.55	1679.24
Formin-like protein 2	NTLLK	502.26	271.51	1324.03	136.2
Formin-like protein 2	NTLLK	785.69	460.72	1563.83	378.08
Formin-like protein 2	NTLLK	899.48	388.99	1982.44	100.49
Formin-like protein 2	NTLLK	2246.5	816.78	5723.81	368.79
Formin-like protein 2	NTLLK	281	322.53	887.34	105.42
Formin-like protein 2	QQQELIAELR	178.02	283.96	619.68	275.42
Formin-like protein 2	QQQELIAELR	1622	1132.45	4205.73	2406.75
Formin-like protein 2	QQQELIAELR	634.38	622.91	1116.39	498.22
Formin-like protein 2	QSTLEK	847.84	596.6	1901.48	745.95
Formin-like protein 2	TLLK	6196.24	6974.04	13256.44	14651.59
Formin-like protein 2	VELEK	6326.73	6847.57	9406.85	9969.75
Formin-like protein 2	VELEK	1301.43	889.66	2124.72	2193.09
Formin-like protein 2	VELEK	1614.61	1863.07	2877.67	3387.75
Formin-like protein 2	VELEK	537.08	874.14	1225.58	1270.01
Formin-like protein 2	VFDLK	2338.99	1571.98	3080.12	2709.11
Formin-like protein 2	YIQK	1033.73	1380.04	1879.71	2790.94
Ras-related protein Rab-9A	DATNVAAAFEEAVR	177.95	290.9	276.86	447.06
Ras-related protein Rab-9A	QVSTEEAQAWCR	1488.44	1243.26	1660.14	2486.7
Ras-related protein Rab-9A	VILLGDGGVGK	482.7	608.32	531.95	837.2
Ras-related protein Rab-9A	SDHLIQTDTVNLHR	1361.19	1262.12	1832.11	2133.78
Ras-related protein Rab-9A	DNGDYPYFETSAK	335.69	345.64	820.49	627.07
Ras-related protein Rab-9A	VLATEDR	740.87	420.36	2181.68	454.15
Ras-related protein Rab-9A	DATNVAAAFEEAVRR	230.23	232.67	412.55	374.46
Ras-related protein Rab-9A	DATNVAAAFEEAVR	914.37	726.35	1249.91	1136.12
Ras-related protein Rab-9A	DATNVAAAFEEAVR	193.12	68.86	354.14	344.05
Ras-related protein Rab-9A	DATNVAAAFEEAVR	257.14	126.7	449.71	182.46
Ras-related protein Rab-9A	DATNVAAAFEEAVR	230.23	232.67	412.55	374.46
Ras-related protein Rab-9A	DATNVAAAFEEAVRR	483.47	744.95	791.88	796.46
Ras-related protein Rab-9A	DATNVAAAFEEAVRR	280.4	381.99	501.72	694.11
Ras-related protein Rab-9A	DNGDYPYFETSAK	701.73	671.04	669.27	1046.92

Ras-related protein Rab-9A	DNGDYPYFETSAK	385.73	188.56	747.78	1051.12
Ras-related protein Rab-9A	DNGDYPYFETSAK	152.87	215.65	261.29	269.59
Ras-related protein Rab-9A	DNGDYPYFETSAK	49.93	173.06	192.29	92.75
Ras-related protein Rab-9A	DNGDYPYFETSAK	49.93	173.06	192.29	92.75
Ras-related protein Rab-9A	EFLNK	29470.13	33626.17	1498.56	2512.54
Ras-related protein Rab-9A	EFLNK	8418.44	9978.23	3628.36	5543.91
Ras-related protein Rab-9A	EFLNK	3317.01	3562.82	1863.66	2212.89
Ras-related protein Rab-9A	EFLNK	7015.5	9010.84	1252.65	1811.17
Ras-related protein Rab-9A	FLNK	1734	1981.99	3684.87	4026.39
Ras-related protein Rab-9A	ILLGD	1243.43	1194.05	2279.76	3355.83
Ras-related protein Rab-9A	QVSTEEAQAWCR	1648.49	1601.11	2573.66	3755.69
Ras-related protein Rab-9A	QVSTEEAQAWCR	750.53	530.03	1295.6	1150.84
Ras-related protein Rab-9A	QVSTEEAQAWCR	737.56	641.56	1145.26	810.37
Ras-related protein Rab-9A	QVSTEEAQAWCR	176.63	421.67	195.8	295.37
Ras-related protein Rab-9A	QVSTEEAQAWCR	408.96	447.02	589.98	752.81
Ras-related protein Rab-9A	QVSTEEAQAWCR	231.3	126.41	558.43	460.45
Ras-related protein Rab-9A	SDHLIQTDTVNLHR	1051.07	1581.88	2044.26	2356.5
Ras-related protein Rab-9A	SDHLIQTDTVNLHR	1393.86	1012.62	2299.21	1835.75
Ras-related protein Rab-9A	SDHLIQTDTVNLHR	708.45	1176.42	2080.44	1790.98
Ras-related protein Rab-9A	SDHLIQTDTVNLHR	896.44	1540.11	2283.53	2430.9
Ras-related protein Rab-9A	SLFK	2176.43	1888	4372.49	4064.77
Ras-related protein Rab-9A	SLFK	1602.55	2203.52	2250.56	3383.6
Ras-related protein Rab-9A	SLFK	876.9	1342.5	1332.59	2049.06
Ras-related protein Rab-9A	SLFK	379.51	803.01	1066.55	1188.16
Ras-related protein Rab-9A	SLFK	1564.59	1379.73	2130.2	3378.26
Ras-related protein Rab-9A	SLFK	582.6	951.15	1398.38	1676.65
Ras-related protein Rab-9A	SSLFK	1141.35	1279.85	1866.96	1780.44
Ras-related protein Rab-9A	VILLGDGGVGK	714.67	668.98	936.6	1442.33
Ras-related protein Rab-9A	VILLGDGGVGK	480.55	395.11	555.28	831.03
Ras-related protein Rab-9A	VILLGDGGVGK	664.72	603.73	1102.85	1268.3
Ras-related protein Rab-9A	VILLGDGGVGK	757.96	647.45	1257.89	1548.21
Ras-related protein Rab-9A	VILLGDGGVGK	998.47	1140.47	1998.26	2248.94
Acyl-protein thioesterase 1	ASFPQGPIGGANR	1170.14	1200.86	1413.49	2448.29
Acyl-protein thioesterase 1	TLVNPANVTFK	1179.69	1533.87	1394.91	1016.16
Acyl-protein thioesterase 1	STPLPAIVPAAR	491.89	444.41	468.71	570.1
Acyl-protein thioesterase 1	ALIDQEVKNGIPSNR	1937.56	1871.33	3572.86	4250.75
Acyl-protein thioesterase 1	ASFPQGPIGGANR	1149.07	836.15	968.8	1728.46
Acyl-protein thioesterase 1	ASFPQGPIGGANR	1911.43	2218.65	3242.49	2732.96
Acyl-protein thioesterase 1	ASFPQGPIGGANR	2527.39	2533.53	4291.53	4083.01
Acyl-protein thioesterase 1	AVIF	438.29	421.77	774.37	921.2
Acyl-protein thioesterase 1	FIDK	2467.9	2133.81	4495.56	5351.67
Acyl-protein thioesterase 1	FIDK	1675.88	1128.61	2070.47	2141.71
Acyl-protein thioesterase 1	IDKL	2055.16	1635.69	2617.23	3027.78
Acyl-protein thioesterase 1	LPLR	640.2	366.14	1243.74	910.95
Acyl-protein thioesterase 1	LTVEK	3245.52	4988.12	3905.94	6233.01
Acyl-protein thioesterase 1	LTVEK	1566.15	1225.5	3317.83	2637.04
Acyl-protein thioesterase 1	LTVEK	4152.7	4189.91	5386.15	7810.68
Acyl-protein thioesterase 1	LTVEK	1494.4	1611.53	2729.44	3567.07
Acyl-protein thioesterase 1	LTVEK	1278	1715.42	2266.08	2856.94
Acyl-protein thioesterase 1	NVTFK	3043.9	3205.78	3877.43	4922.46
Acyl-protein thioesterase 1	QFIDK	29470.13	33626.17	1498.56	2512.54
Acyl-protein thioesterase 1	QFIDK	7015.5	9010.84	1252.65	1811.17
Acyl-protein thioesterase 1	STPLPAIVPAAR	463.25	189.55	414.99	363.67
Acyl-protein thioesterase 1	STPLPAIVPAAR	853.39	793.24	1727.87	1140.48
Acyl-protein thioesterase 1	STPLPAIVPAAR	488.09	394.56	395.19	551.53
WW domain-binding protein 11	AQLSQYFDAVK	569.35	451.79	617.55	670.84
WW domain-binding protein 11	ELTPLQAMMLR	278.53	566.54	315.41	289.87
WW domain-binding protein 11	FMNPTDQAR	899.46	922.74	1642.46	2124.94
WW domain-binding protein 11	RRDEDMLYSPELAQR	512.96	384.17	1137.38	812.34
WW domain-binding protein 11	FVPTALR	3407.89	3413.78	605.29	628.7
WW domain-binding protein 11	AVSILPLLGHGVPR	231.22	134.83	142.52	133.69
WW domain-binding protein 11	SEDDSAVPLAK	965.42	1320.22	1844.34	1604.32
WW domain-binding protein 11	EMEGLL	128.29	91.33	118.26	148.43
WW domain-binding protein 11	ELTPLQAM	250.18	176.83	335.69	332.07

WW domain-binding protein 11	AFMK	4525.53	4695.89	4415.49	6751.71
WW domain-binding protein 11	AFMK	3007.55	3711.17	9382.64	10454.26
WW domain-binding protein 11	AQLSQYFDAVK	634.91	676.88	678.77	193.16
WW domain-binding protein 11	AVSILPLLGHGVPR	137.42	146.99	97.51	148.68
WW domain-binding protein 11	DAVK	1441.21	2292.24	3984.99	3276.45
WW domain-binding protein 11	ELTPLQAMMLR	193.77	110.8	192	220.09
WW domain-binding protein 11	ELTPLQAMMLR	229.94	260.69	304.13	455.39
WW domain-binding protein 11	EMEGLL	229.93	261.68	255.06	371.99
WW domain-binding protein 11	EMEGLL	360.04	173.46	312.72	344.34
WW domain-binding protein 11	EMEGLL	257.1	131.42	216.31	219.65
WW domain-binding protein 11	EMEGLL	204.05	265.63	154.73	275.11
WW domain-binding protein 11	FMNPTDQAR	229.44	310.29	405.24	381.74
WW domain-binding protein 11	FVPTALR	2415.33	2327.54	211.1	683.57
WW domain-binding protein 11	FVPTALR	2035.75	1957.82	370.45	410.41
WW domain-binding protein 11	FVPTALR	2247.21	2660.94	3058.38	3106.15
WW domain-binding protein 11	FVPTALR	2510.02	2440.5	286.83	490.34
WW domain-binding protein 11	FVPTALR	13233.75	12852.85	2076.87	3223.95
WW domain-binding protein 11	FVPTALR	3796.69	3086.41	534.56	495.4
WW domain-binding protein 11	FVPTALR	1550.46	1818.06	2573.22	2475.31
WW domain-binding protein 11	FVPTALR	3887.79	3980.04	1526.15	1975.23
WW domain-binding protein 11	FVPTALR	4576.35	4248.86	1130.24	1445.56
WW domain-binding protein 11	FVPTALR	4131.9	3797.86	1984.09	1821.54
WW domain-binding protein 11	LYEK	708.12	1102.77	1629.39	2302.64
WW domain-binding protein 11	RRDEDMLYSPELAQR	714.84	652	977.97	1138.3
WW domain-binding protein 11	VLKD	1985.44	2459.42	2975.86	4539.97
ADP-ribosylation factor-like protein 1	AILVVFANK	460.54	244.13	278.3	473.04
ADP-ribosylation factor-like protein 1	ILILGLDGAGK	165.11	283.6	152.93	299.93
ADP-ribosylation factor-like protein 1	WQIFK	900.25	844.23	1108.55	877.42
ADP-ribosylation factor-like protein 1	AILVVFANK	384.61	299.03	393.59	280.27
ADP-ribosylation factor-like protein 1	AILVVFANK	1010.18	90.34	126.61	225.34
ADP-ribosylation factor-like protein 1	GAGK	216.55	307.68	954	707.01
ADP-ribosylation factor-like protein 1	IGISK	6212.44	5798.33	2687.24	3461.96
ADP-ribosylation factor-like protein 1	IGISK	6356.18	7980.2	3642.09	3873.59
ADP-ribosylation factor-like protein 1	IGISK	4918.09	6230.62	3546.17	4981.69
ADP-ribosylation factor-like protein 1	IGISK	6682.11	8544.92	2065.25	4492.65
ADP-ribosylation factor-like protein 1	IGISK	7142.75	6971.16	3334.23	3816.36
ADP-ribosylation factor-like protein 1	IGISK	983.21	946.4	1601.52	1150.15
ADP-ribosylation factor-like protein 1	IGISK	2985.09	2632.98	3439.12	3614.87
ADP-ribosylation factor-like protein 1	ILILGLDGAGK	307.52	256.34	234.87	191.48
ADP-ribosylation factor-like protein 1	ILILGLDGAGK	799.18	511.19	666.54	683
ADP-ribosylation factor-like protein 1	ILYR	526.12	680.13	650.51	1023.27
ADP-ribosylation factor-like protein 1	ILYR	589.71	354.57	724.47	922.15
ADP-ribosylation factor-like protein 1	LVAML	103.95	48.77	132.24	132.23
ADP-ribosylation factor-like protein 1	LVAML	173.67	75.61	144.31	168.13
ADP-ribosylation factor-like protein 1	QIFK	944.52	832.03	698.7	1348.21
ADP-ribosylation factor-like protein 1	QIFK	812.14	826.51	925.19	1053.71
ADP-ribosylation factor-like protein 1	QIFK	2341.82	4378.22	2760.69	5011.31
ADP-ribosylation factor-like protein 1	WQIFK	637.14	669.41	720.59	1487.08
ADP-ribosylation factor-like protein 1	WQIFK	1182.53	1360.38	1509.25	1966.72
ADP-ribosylation factor-like protein 1	WQIFK	595.96	1013.96	1699.53	1388.7
MAP/microtubule affinity-regulating kinase 3	SSTVPSSNTASGGMTR	157.95	139.29	190.05	228.04
MAP/microtubule affinity-regulating kinase 3	TPVASTHSISSAATPDR	89.92	51.93	293.05	242.41
MAP/microtubule affinity-regulating kinase 3	NSIASCADEQPHIGNYR	487.18	378.44	1196.12	1230.34
MAP/microtubule affinity-regulating kinase 3	ENLLK	999.94	888.79	1889.7	2038.79
MAP/microtubule affinity-regulating kinase 3	ENLLK	1203	720.98	1415.15	2014.67
MAP/microtubule affinity-regulating kinase 3	ENLLK	380.23	731.62	446.44	716.79
MAP/microtubule affinity-regulating kinase 3	EVAIK	610.73	724.77	951.1	1138.43
MAP/microtubule affinity-regulating kinase 3	EVAIK	810.28	797.48	1570.16	2299.78
MAP/microtubule affinity-regulating kinase 3	EVAIK	1872.73	1467.66	2874.52	2381.52
MAP/microtubule affinity-regulating kinase 3	EVAIK	1190.96	1697	3549.49	2900.05
MAP/microtubule affinity-regulating kinase 3	EVAIK	2273.7	3448.11	2957.4	2913.3
MAP/microtubule affinity-regulating kinase 3	EVAIK	2026.61	2753.66	3109.49	3904.91
MAP/microtubule affinity-regulating kinase 3	IAFK	1793.47	1000.15	1876.92	2158.65
MAP/microtubule affinity-regulating kinase 3	IAFK	1711.02	1593.23	1883.12	3627.6

MAP/microtubule affinity-regulating kinase 3	IANELKL	1981.46	1469.88	2047.41	2623.95
MAP/microtubule affinity-regulating kinase 3	IANELKL	1763.96	1043.96	1201.19	1227.92
MAP/microtubule affinity-regulating kinase 3	IANELKL	1774.55	1805.83	2516.73	2219.34
MAP/microtubule affinity-regulating kinase 3	IANELKL	30848.87	35282.73	2264.81	3591.41
MAP/microtubule affinity-regulating kinase 3	IANELKL	21733.49	23202.9	1428.84	2562.22
MAP/microtubule affinity-regulating kinase 3	IANELKL	47661.21	49897.16	786.09	2683.3
MAP/microtubule affinity-regulating kinase 3	IANELKL	2665.49	2718.95	3176.73	2726.96
MAP/microtubule affinity-regulating kinase 3	IANELKL	3322.55	3228.11	953.92	719.21
MAP/microtubule affinity-regulating kinase 3	IANELKL	14520.77	17400.01	1835.78	2128.39
MAP/microtubule affinity-regulating kinase 3	IANELKL	2582.63	2075.33	1276.81	1927.3
MAP/microtubule affinity-regulating kinase 3	IANELKL	19547.32	19298.13	634.25	2656.79
MAP/microtubule affinity-regulating kinase 3	IANELKL	31566.22	33132.98	1671	3414.74
MAP/microtubule affinity-regulating kinase 3	IANELKL	10562.66	12602.16	3522.41	5318.37
MAP/microtubule affinity-regulating kinase 3	IANELKL	1124	972.62	745.74	797.41
MAP/microtubule affinity-regulating kinase 3	IANELKL	23795.87	26252.41	1634.26	3374.62
MAP/microtubule affinity-regulating kinase 3	IANELKL	2331.47	1783.78	1087.49	1328.42
MAP/microtubule affinity-regulating kinase 3	IANELKL	2463.29	1951.25	671.24	987.84
MAP/microtubule affinity-regulating kinase 3	IANELKL	1438.56	1384.45	1454.58	758.28
MAP/microtubule affinity-regulating kinase 3	IIDK	1325.13	782.29	1817.38	2421.19
MAP/microtubule affinity-regulating kinase 3	IIDK	1259.27	1224.83	1231.17	1605.55
MAP/microtubule affinity-regulating kinase 3	IIDK	11494.35	11829.41	19877.02	24045.93
MAP/microtubule affinity-regulating kinase 3	IIDK	8107.16	8072.27	13308.71	15925.9
MAP/microtubule affinity-regulating kinase 3	IIDK	1686.04	2252.31	2137.37	2898.31
MAP/microtubule affinity-regulating kinase 3	IIDK	1799.72	1871.91	3038.68	3494.33
MAP/microtubule affinity-regulating kinase 3	IIDK	2542.42	2325.42	3467.44	4284.44
MAP/microtubule affinity-regulating kinase 3	IIDK	2044.79	2765.98	3071.91	3593.15
MAP/microtubule affinity-regulating kinase 3	IIDK	11419.16	12769	18247.56	23009.46
MAP/microtubule affinity-regulating kinase 3	IIDK	4581.13	5798.65	7107.07	10954.17
MAP/microtubule affinity-regulating kinase 3	IIDK	1817.73	1794.65	2751.75	3695.78
MAP/microtubule affinity-regulating kinase 3	IIDK	6734.04	6925.33	10184.37	13283.95
MAP/microtubule affinity-regulating kinase 3	IIDK	2816.96	2116	3299.59	5206.11
MAP/microtubule affinity-regulating kinase 3	IIDK	6381.53	5263.29	12843.03	15057.89
MAP/microtubule affinity-regulating kinase 3	IIDK	1634.62	1694.21	1902.07	2653.86
MAP/microtubule affinity-regulating kinase 3	IIDK	2610.13	3614.15	4202.4	5576.11
MAP/microtubule affinity-regulating kinase 3	IIDK	1520.78	1558.44	3184.54	2618.89
MAP/microtubule affinity-regulating kinase 3	IIDK	8800.84	9644.57	14104.83	17354.68
MAP/microtubule affinity-regulating kinase 3	IIDK	994.06	1469.88	1517.2	2486.21
MAP/microtubule affinity-regulating kinase 3	IPFYMSTDCENLLK	332.38	353.31	453.35	361.57
MAP/microtubule affinity-regulating kinase 3	ISDQK	2987.47	3036.22	4600.17	3368.25
MAP/microtubule affinity-regulating kinase 3	IVKL	1071.45	1590.3	2510.33	3331.23
MAP/microtubule affinity-regulating kinase 3	IVKL	2944.6	2377.74	4777.68	5411.7
MAP/microtubule affinity-regulating kinase 3	IVKL	2298.42	2602.15	3385.07	5172.57
MAP/microtubule affinity-regulating kinase 3	IVKL	3592.95	2897	5810.44	6800.76
MAP/microtubule affinity-regulating kinase 3	LEQIMK	999.93	1315.06	1228.43	1775.26
MAP/microtubule affinity-regulating kinase 3	LEQIMK	662.14	752.56	398.88	515.18
MAP/microtubule affinity-regulating kinase 3	LFSK	3056.35	3570.66	4399.12	4841.04
MAP/microtubule affinity-regulating kinase 3	LFSK	1389.34	1672.03	2302.68	2188.84
MAP/microtubule affinity-regulating kinase 3	LSLAK	3807.56	4246.84	7664.89	7968.16
MAP/microtubule affinity-regulating kinase 3	NIVK	127.27	191.64	266.02	165.17
MAP/microtubule affinity-regulating kinase 3	NLLK	540.63	204.34	676.13	887.3
MAP/microtubule affinity-regulating kinase 3	NLLK	359.27	249.9	363.8	1049.81
MAP/microtubule affinity-regulating kinase 3	NLLK	202.84	385.47	613.68	578.56
MAP/microtubule affinity-regulating kinase 3	QNLK	1331.98	1275.66	2386.22	2606.35
MAP/microtubule affinity-regulating kinase 3	QNLK	1114.11	1205	1892.83	2006.06
Uncharacterized protein CSNK1G2-AS1	AGPRSTADTRPR	2061.37	1553.88	1588.67	1488.34
Uncharacterized protein CSNK1G2-AS1	VAISK	6212.44	5798.33	2687.24	3461.96
Kelch-like ECH-associated protein 1	IGVGVAVLNR	1896.68	1548.08	1202.49	2139.46
Kelch-like ECH-associated protein 1	QAFGIMNELR	359.53	224.13	361.15	340.66
Kelch-like ECH-associated protein 1	LADLQVPR	426.08	350.89	229.32	167.9
Kelch-like ECH-associated protein 1	SALGITVHQGR	822.43	553.93	1179.92	1367.5
Kelch-like ECH-associated protein 1	YEPER	360.58	120.17	393.55	339.13
Kelch-like ECH-associated protein 1	DLNVR	435.32	395.24	554.73	533.41
Kelch-like ECH-associated protein 1	IGVGVAVLNR	1305.81	1733.83	1147.03	971.75
Kelch-like ECH-associated protein 1	IGVGVAVLNR	3484.76	3798.34	1087.63	1690.32

Kelch-like ECH-associated protein 1	IGVGVAVLNR	3727.72	3943.53	1419.15	1765.11
Kelch-like ECH-associated protein 1	IGVGVAVLNR	2154.69	1908.76	925.15	1089.45
Kelch-like ECH-associated protein 1	IGVGVAVLNR	3761.94	3115.93	1914.45	1892.97
Kelch-like ECH-associated protein 1	IGVGVAVLNR	8734.21	10057.52	2325.42	3302.84
Kelch-like ECH-associated protein 1	IGVGVAVLNR	2869.23	2696.41	991.83	1823.94
Kelch-like ECH-associated protein 1	IGVGVAVLNR	2274.97	2046.4	1175.7	1524.17
Kelch-like ECH-associated protein 1	IGVGVAVLNR	3131.11	1930.06	708.15	1210.49
Kelch-like ECH-associated protein 1	IGVGVAVLNR	1797.49	2412.2	894.22	980.58
Kelch-like ECH-associated protein 1	IGVGVAVLNR	2188.48	3037.78	2078.73	3109.77
Kelch-like ECH-associated protein 1	IGVGVAVLNR	2556.5	2742.27	2210.08	3260.3
Kelch-like ECH-associated protein 1	IGVGVAVLNR	2017.17	1026.66	863.07	1415.5
Kelch-like ECH-associated protein 1	IGVGVAVLNR	3346.84	2637.06	2496.55	3635.45
Kelch-like ECH-associated protein 1	IGVGVAVLNR	6512.01	6626.98	1980.13	2961.28
Kelch-like ECH-associated protein 1	IGVGVAVLNR	4467.3	4810.42	1037.75	2201.62
Kelch-like ECH-associated protein 1	LIEF	730.93	338.56	307.57	135.93
Kelch-like ECH-associated protein 1	LQVK	858.84	680.67	1053.15	1421.2
Kelch-like ECH-associated protein 1	QAFGIMNELR	1374.62	573	766.04	1791.26
Retinoic acid receptor responder protein 2	LVHCPIETQVLR	730.99	546.16	835.68	1127.76
Retinoic acid receptor responder protein 2	LEFK	5980.65	6890.07	700.28	851.97
Retinoic acid receptor responder protein 2	LEFK	2365.31	3014.86	702.85	1203.49
Retinoic acid receptor responder protein 2	LGSEDK	4758.08	4592.46	4583.98	6452.94
Retinoic acid receptor responder protein 2	LVHCPIETQVLR	307.02	305.1	377.92	471.94
Lymphocyte transmembrane adapter 1	DGVTPTLSTIRGR	661.9	776.23	399.07	424.82
Lymphocyte transmembrane adapter 1	RQVPYLR	2918.01	1493.74	519.58	354.91
Lymphocyte transmembrane adapter 1	LLAI	196.22	188.4	226.96	250.42
Lymphocyte transmembrane adapter 1	LLAI	75.78	172.69	115.6	69.95

Table S3. Clinicopathological Characteristics of Clinical Samples and Expression of RNF219 and LGALS3 in HCC

**Characteristics**	**No. patients**	**(%)**
**Age (years)**		
≤ 60	249	0.52
> 60	226	0.48
**Gender**		
Male	410	0.86
Female	65	0.14
**HBsAg**		
positive	367	0.77
negative	108	0.23
**HCV-Ab**		
positive	12	0.03
negative	463	0.97
**AFP(ng/ml)**		
> 20	319	0.67
≤ 20	156	0.33
**Clinical stage**		
I-II	192	0.40
III-IV	283	0.60
**Tumor size**		
> 5 cm	211	0.44
≤ 5 cm	264	0.56
**Intrahepatic control**		
Yes	342	0.72
No	133	0.28
**Bone metastasis**		
Yes	38	0.08
No	437	0.92

Continued

Table S3 (Continued**)**

**Characteristics**	**No. patients**	**(%)**
**Other extrahepatic metastasis**		
Yes	158	0.33
No	317	0.67
**Vital status (at follow-up)**		
Alive	314	0.66
Death due to liver cancer cause	161	0.34
**Expression of RNF219**		
Low expression	292	0.61
High expression	183	0.39
**Expression of LGALS3**		
Low expression	283	0.60
High expression	192	0.40

HBsAg: hepatitis B surface antigen, HCV-Ab: hepatitis C virus antibody, AFP: α-fetoprotein, RNF219: ring finger protein 219

Table S4. Correlation between RNF219 and LGALS3 Expression and Clinicopathologic Charateristics of HCC Patient

**Characteristics**	**RNF219 Protein level**		**Chi-square test**	**LGALS3 Protein level**		**Chi-square test**
	**Low**	**High**	***P* value**	**Low**	**High**	***P* value**
**Age (years)**						
≤ 60	158	91	0.201	149	100	0.903
> 60	134	92		134	92	
**Gender**						
Male	253	157	0.447	241	169	0.373
Female	39	26		42	23	
**HBsAg**						
positive	221	146	0.178	216	151	0.554
negative	71	37		67	41	
**HCV-Ab**						
positive	7	5	0.520	9	3	0.270
negative	285	178		274	189	
**AFP (ng/ml)**						
> 20	202	117	0.139	191	128	0.851
≤ 20	90	66		92	64	
**Clinical stage**						
I-II	78	114	< 0.001	76	116	< 0.001
III-IV	214	69		207	76	
**Tumor size**						
> 5 cm	128	83	0.409	133	78	0.170
≤ 5 cm	164	100		150	114	
**Intrahepatic control**						
Yes	215	127	0.185	201	141	0.565
No	77	56		82	51	
**Bone metastasis**						
Yes	5	33	< 0.001	8	30	< 0.001
No	287	150		275	162	

Continued

Table S4 (Continued**)**

Characteristics	RNF219 Protein level		Chi-square test	LGALS3 Protein level		Chi-square test
	Low	High	*P* value	Low	High	*P* value
**Other extrahepatic metastasis**						
Yes	96	62	0.449	103	55	0.079
No	196	121		180	137	
**Vital status (at follow-up)**						
Alive	244	70	< 0.001	261	53	< 0.001
Death	48	113		22	139	
**Expression of LGALS3**				**Expression of RNF219**		
Low expression	248	35	< 0.001	248	44	< 0.001
High expression	44	148		35	148	

HBsAg: hepatitis B surface antigen, HCV-Ab: hepatitis C virus antibody, AFP: α-fetoprotein,

RNF219: ring finger protein 219

Table S5. Univariate and Multivariate Analyses of Various Prognostic Parameters in HCC Patients by Cox-regression Analysis

	**Univariate analysis**			**Multivariate analysis**		
	C. OR	95% CI	P-value	A. OR	95% CI	P-value
Clinical stage	1.542	1.105-2.152	0.011	2.246	1.526-3.306	< 0.001
Bone metastasis	22.440	14.470-34.800	< 0.001	4.235	2.108-7.449	< 0.001
Exp. of RNF219	1.414	1.334-1.498	< 0.001	1.261	1.181-1.348	< 0.001
Exp. of LGALS3	1.379	1.279-1.485	< 0.001	1.125	1.042-1.215	0.002

Exp.: Expression; Abbreviations: C. OR, crude odds ratio; CI, confidence interval; A. OR, adjusted odds ratio;

Table S6. Bone metastases related events

Cell line	Mouse	Metastasis sites								Fracture
		Skull	Spine	Fore limb	Femur	Knee /tibia	Pelvis	Ribs/ sternum	Scapula	
HCCLM3-Parental (n=8)	1	-	-	+	+	+	-	-	+	-
	2	-	+	-	+	+	-	-	+	-
	3	-	-	-	+	+	-	-	-	-
	4	-	+	+	+	+	-	-	+	-
	5	+	+	+	+	+	-	-	-	+
	6	+	+	+	+	+	-	-	+	+
	7	-	+	-	+	+	+	-	-	-
	8	-	-	-	+	+	-	-	-	-
HCCLM3/RNF219 (n=8)	1	+	+	+	+	+	-	-	+	-
	2	+	+	+	+	+	+	-	+	+
	3	+	+	+	+	+	+	+	+	+
	4	+	+	+	+	+	+	+	+	+
	5	+	+	+	+	+	+	+	+	+
	6	-	+	+	+	+	-	+	+	-
	7	+	+	+	+	+	+	+	+	+
	8	-	-	-	+	-	-	-	-	+
Huh7 (n=8)	1	-	-	-	+	-	-	-	-	-
	2	-	-	+	+	+	-	-	-	-
	3	-	-	+	+	+	-	-	-	-
	4	-	-	-	+	+	-	-	-	-
	5	-	+	+	+	+	-	-	-	-
	6	-	+	+	+	+	-	+	-	-
	7	-	+	-	+	+	+	-	-	-
	8	+	+	+	+	+	-	-	-	-
Huh7/RNF219 (n=8)	1	+	+	+	+	+	-	-	-	+
	2	+	+	+	+	+	+	+	+	+
	3	+	+	+	+	+	+	+	+	+
	4	+	+	+	+	+	+	+	+	+
	5	-	+	+	+	+	+	+	-	+
	6	+	-	+	+	+	+	-	-	-
	7	-	+	+	+	+	-	+	+	+
	8	+	+	+	+	+	+	+	+	+
HCCLM3-BM4 (n=8)	1	-	+	+	+	+	-	-	+	+
	2	-	-	+	+	+	-	-	+	+
	3	-	-	+	+	+	-	-	+	+
	4	-	-	+	+	+	-	-	+	-
	5	-	-	+	+	+	-	-	-	-
	6	-	+	+	+	+	-	-	+	-
	7	-	+	+	+	+	-	-	-	+
	8	-	-	+	+	+	-	-	-	-
HCCLM3-BM4-RNF219 sh#1 (n=8)	1	-	-	-	-	-	-	-	-	-
	2	-	-	-	-	-	-	-	-	-
	3	-	-	-	-	-	-	-	-	-
	4	-	-	-	-	+	-	-	-	-
	5	-	-	-	-	-	-	-	-	-
	6	-	-	-	-	+	-	-	-	-
	7	-	-	-	-	-	-	-	-	-
	8	-	-	-	-	+	-	-	-	-

**HCCLM3-BM4-RNF219 sh#2 (n=8)**	**1**	-	-	-	-	-	-	-	-	-
	**2**	-	-	-	-	-	-	-	-	-
	**3**	-	-	-	-	-	-	-	-	-
	**4**	-	-	-	-	+	-	-	-	-
	**5**	-	-	-	-	+	-	-	-	-
	**6**	-	-	-	-	+	-	-	-	-
	**7**	-	-	-	-	+	-	-	-	-
	**8**	-	-	-	-	+	-	-	-	-
**MHCC97H (n=8)**	**1**	+	-	+	+	+	-	+	+	+
	**2**	+	-	+	+	+	-	+	+	+
	**3**	-	-	-	+	+	-	+	+	-
	**4**	-	-	+	+	+	-	+	+	+
	**5**	+	+	-	+	+	-	+	+	-
	**6**	-	-	-	+	+	-	+	+	-
	**7**	-	-	-	+	+	-	-	+	-
	**8**	+	-	+	+	+	-	+	+	+
**MHCC97H-RNF219 sh#1 (n=8)**	**1**	-	-	-	-	-	-	-	-	-
	**2**	-	-	-	-	-	-	-	-	-
	**3**	-	-	-	-	+	-	-	-	-
	**4**	-	-	-	-	-	-	-	-	-
	**5**	-	-	-	-	-	-	-	-	-
	**6**	-	-	-	-	+	-	-	-	-
	**7**	-	-	-	-	+	-	-	-	-
	**8**	-	-	-	-	-	-	-	-	-
**MHCC97H-RNF219 sh#2 (n=8)**	**1**	-	-	-	-	-	-	-	-	-
	**2**	-	-	-	-	-	-	-	-	-
	**3**	-	-	-	-	-	-	-	-	-
	**4**	-	-	-	-	+	-	-	-	-
	**5**	-	-	-	-	+	-	-	-	-
	**6**	-	-	-	-	-	-	-	-	-
	**7**	-	-	-	-	+	-	-	-	-
	**8**	-	-	-	-	-	-	-	-	-

Table S7. List of quantified protein in HCC/RNF219-CM compared with HCC/vector-CM, and in mature

Protein ID (Uniprot)	Gene name	Protein Name	Log2Foldchange		Pvalue
			Proteins in osteodast OC/ pre-osteodast OP	Secreted proteins in RNF219/Vector cells culture medium	
P00558	PGK1	Phosphoglycerate kinase 1	0.98	2.756	1.90E-08
P62249	RS16	40S ribosomal protein S16	0.58	2.472	4.68E-02
P53602	MVD1	Diphosphomevalonate decarboxylase	1.03	2.011	4.67E-02
P17931	LEG3	Galectin-3	1.79	1.997	6.01E-07
P62888	RL30	60S ribosomal protein L30	0.85	1.956	2.43E-02
P46776	RL27A	Ubiquitin-40S ribosomal protein S27a	0.1	1.924	7.67E-03
P43243	MATR3	Matrin-3	0.17	1.836	2.77E-03
Q12874	SF3A3	Splicing factor 3A subunit 3	0.8	1.791	1.74E-04
Q92688	AN32B	Acidic leucine-rich nuclear phosphoprotein 32 family member B	0.83	1.777	1.39E-03
P61353	RL27	60S ribosomal protein L27	0.88	1.691	1.04E-03
P18124	RL7	60S ribosomal protein L7	0.43	1.672	1.52E-02
Q99613	EIF3C	Eukaryotic translation initiation factor 3 subunit C	0.78	1.613	1.00E-02
P04792	HSPB1	Hsp70-binding protein 1	0.86	1.582	5.77E-04
O14737	PDCD5	Programmed cell death protein 5	0.52	1.472	4.68E-02
P19338	NUCL	Nucleolin	0.32	1.378	1.48E-05
Q07955	SRSF1	Serine/arginine-rich splicing factor 1	1.69	1.346	2.18E-04
P62913	RL11	60S ribosomal protein L11	0.24	1.161	6.59E-04
Q9P258	RCC2	Protein RCC2	0.36	1.141	3.98E-02
P62906	RL10A	60S ribosomal protein L10a	1.1	1.118	6.96E-04
P30050	RL12	60S ribosomal protein L12	1.03	1.035	5.18E-05
Q99733	NP1L4	Nucleosome assembly protein 1-like 4	1.19	0.998	4.80E-02
P84098	RL19	60S ribosomal protein L19	0.25	0.913	9.45E-04
P39019	RS19	40S ribosomal protein S19	0.18	0.877	2.89E-02
P47914	RL29	60S ribosomal protein L29 -around 25k	0.06	0.875	1.26E-04
Q13765	NACA	Nascent polypeptide-associated complex subunit alpha	0.81	0.866	1.84E-04
O60218	AK1BA	Aldose reductase	1.22	0.846	4.93E-03
P62277	RS13	40S ribosomal protein S13	0.89	0.826	1.01E-02
P62244	RS15A	40S ribosomal protein S15a	1.18	0.818	5.73E-04
P05455	LA	Lupus La protein homolog - around 50k	0.35	0.817	1.34E-04
P39687	AN32A	Acidic leucine-rich nuclear phosphoprotein 32 family member A	1.19	0.797	1.48E-04
Q13263	TIF1B	Transcription intermediary factor 1-beta	0.65	0.79	8.50E-03
Q6IBS0	TWF2	Twinfilin-2	1.62	0.786	6.09E-03
Q99497	PARK7	Protein DJ-1	1.29	0.761	1.24E-02
P40121	CAPG	Macrophage-capping protein	1.39	0.721	2.23E-02
P26373	RL13	60S ribosomal protein L13	0.17	0.696	3.50E-02
Q01105	SET	Protein SET	0.89	0.694	2.29E-03
Q86VP6	CAND1	Cullin-associated NEDD8-dissociated protein 1	0.79	0.691	2.70E-04
P61626	LYSC	Lysozyme C-1	0.12	0.689	3.73E-04
P05388	RLA0	60S acidic ribosomal protein P0	0.89	0.675	3.05E-03
P05067	A4	Amyloid beta A4 precursor protein-binding family B member 1-interacting protein	0.85	0.667	3.44E-06
P04406	G3P	Glyceraldehyde-3-phosphate dehydrogenase	0.92	0.634	3.19E-04
P07900	HS90A	Heat shock protein HSP 90-alpha	0.8	0.609	3.54E-04

Q61937	Npm1	Nucleophosmin	0.83	1.279	1.47E-03

Table S8. Potential trans-regulatory factors were enriched on the LGALS3 promoter in HCCLM3 cells

Accession	Description	-10lgP	#Peptides	#Unique
P46937|YAP1_HUMAN	Transcriptional coactivator YAP1 OS=Homo sapiens GN=YAP1 PE=1 SV=2	343.01	57	57
P35222|CTNB1_HUMAN	Catenin beta-1 OS=Homo sapiens GN=CTNNB1 PE=1 SV=1	395.8	46	45
P26358|DNMT1_HUMAN	DNA (cytosine-5)-methyltransferase 1 OS=Homo sapiens GN=DNMT1 PE=1 SV=2	98.66	10	8
Q8NFU7|TET1_HUMAN	Tet methylcytosine dioxygenase 1 OS=Homo sapiens GN=TET PE=1 SV=1	124.14	9	9
Q9Y3Y2|CHTOP_HUMAN	Chromatin target of PRMT1 protein OS=Homo sapiens GN=CHTOP PE=1 SV=2	120.69	8	8
Q99873|ANM1_HUMAN	Protein arginine N-methyltransferase 1 OS=Homo sapiens GN=PRMT1 PE=1 SV=2	49.46	9	7
Q9UMN6|KMT2B_HUMAN	Histone-lysine N-methyltransferase 2B OS=Homo sapiens GN=KMT2B PE=1 SV=1	136.52	10	10
P35221|CTNA1_HUMAN	Catenin alpha-1 OS=Homo sapiens GN=CTNNA1 PE=1 SV=1	16.37	5	4
P25054|APC_HUMAN	Adenomatous polyposis coli protein OS=Homo sapiens GN=APC PE=1 SV=2	13.1	3	3
O60341|KDM1A_HUMAN	Lysine-specific histone demethylase 1A OS=Homo sapiens GN=KDM1A PE=1 SV=1	19.72	5	5

Protein Accession	Peptide	Unique	-10lgP	Mass	Length	Start	End
P46937|YAP1_HUMAN	R.DESTDSGLSMSSYSVPR.T	Y	97.51	1816.7784	17	395	411
P46937|YAP1_HUMAN	K.QPPPLAPQSPQGGVMGGSNSNQQQQMR.L	Y	95.59	2818.324	27	281	307
P46937|YAP1_HUMAN	R.SQLPTLEQDGGTQNPVSSPGMSQELR.T	Y	95.06	2755.3083	26	350	375
P46937|YAP1_HUMAN	R.DESTDSGLSM(+15.99)SSYSVPR.T	Y	95.01	1832.7734	17	395	411
P46937|YAP1_HUMAN	R.QASTDAGTAGALTPQHVR.A	Y	93.27	1779.8864	18	107	124
P46937|YAP1_HUMAN	R.GDSETDLEALFN(+.98)AVMNPK.T	Y	89.86	1950.8881	18	59	76
P46937|YAP1_HUMAN	R.YFLN(+.98)HIDQTTTWQDPR.K	Y	89.82	2034.9435	16	188	203
P46937|YAP1_HUMAN	R.GDSETDLEALFNAVMNPK.T	Y	87.99	1949.9041	18	59	76
P46937|YAP1_HUMAN	R.TPDDFLNSVDEMDTGDTINQSTLPSQQNR.F	Y	87.4	3237.4368	29	412	440
P46937|YAP1_HUMAN	R.Q(-17.03)ASTDAGTAGALTPQHVR.A	Y	86.94	1762.8597	18	107	124
P46937|YAP1_HUMAN	R.QSSFEIPDDVPLPAGWEMAK.T	Y	86.35	2216.0459	20	162	181
P46937|YAP1_HUMAN	R.AHSSPASLQLGAVSPGTLTPTGVVSGPAATPTAQHLR.Q	Y	84.45	3533.8589	37	125	161
P46937|YAP1_HUMAN	R.TMTTN(+.98)SSDPFLNSGTYHSR.D	Y	80.52	2115.9167	19	376	394
P46937|YAP1_HUMAN	H.SSPASLQLGAVSPGTLTPTGVVSGPAATPTAQHLR.Q	Y	80.2	3325.7629	35	127	161
P46937|YAP1_HUMAN	K.QPPPLAPQSPQGGVM(+15.99)GGSNSNQQQQMR.L	Y	80.18	2834.3188	27	281	307
P46937|YAP1_HUMAN	R.YFLNHIDQTTTWQDPR.K	Y	79.86	2033.9595	16	188	203
P46937|YAP1_HUMAN	R.TPDDFLNSVDEM(+15.99)DTGDTINQSTLPSQQNR.F	Y	79.85	3253.4316	29	412	440
P46937|YAP1_HUMAN	R.TMTTNSSDPFLNSGTYHSR.D	Y	79.63	2114.9326	19	376	394
P46937|YAP1_HUMAN	R.KLPDSFFKPPEPK.S	Y	79.07	1528.8289	13	90	102
P46937|YAP1_HUMAN	R.GDSETDLEALFNAVM(+15.99)NPK.T	Y	78.56	1965.8989	18	59	76
P46937|YAP1_HUMAN	A.SLQLGAVSPGTLTPTGVVSGPAATPTAQHLR.Q	Y	75.34	2983.6091	31	131	161
P46937|YAP1_HUMAN	K.QPPPLAPQSPQGGVMGGSNSNQQQQM(+15.99)R.L	Y	73.73	2834.3188	27	281	307
P46937|YAP1_HUMAN	K.LPDSFFKPPEPK.S	Y	72.39	1400.7339	12	91	102
P46937|YAP1_HUMAN	L.RSQLPTLEQDGGTQNPVSSPGMSQELR.T	Y	69.8	2911.4094	27	349	375
P46937|YAP1_HUMAN	R.Q(-17.03)SSFEIPDDVPLPAGWEMAK.T	Y	67.57	2199.0193	20	162	181
P46937|YAP1_HUMAN	R.SQLPTLEQDGGTQNPVSSPGM(+15.99)SQELR.T	Y	66.83	2771.3032	26	350	375
P46937|YAP1_HUMAN	R.TMTTNSSDPFLN(+.98)SGTYHSR.D	Y	64.49	2115.9167	19	376	394
P46937|YAP1_HUMAN	S.SFEIPDDVPLPAGWEMAK.T	Y	63.67	2000.9553	18	164	181
P46937|YAP1_HUMAN	K.QPPPLAPQSPQGGVMGGSN(+.98)SNQQQQMR.L	Y	62.7	2819.3079	27	281	307
P46937|YAP1_HUMAN	R.TPDDFLNSVDEMD.T	Y	61.27	1496.5977	13	412	424
P46937|YAP1_HUMAN	R.QSSFEIPDDVPLPAGWEM(+15.99)AK.T	Y	60.93	2232.0408	20	162	181
P46937|YAP1_HUMAN	L.SSDILNDMESVLAATK.L	Y	60.81	1692.824	16	479	494
P46937|YAP1_HUMAN	R.TPDDFLN(+.98)SVDEMDTGDTINQSTLPSQQNR.F	Y	58.76	3238.4209	29	412	440
P46937|YAP1_HUMAN	H.VRGDSETDLEALFNAVMNPK.T	Y	58.63	2205.0735	20	57	76
P46937|YAP1_HUMAN	K.AMLSQMNVTAPTSPPVQQN.M	Y	58.54	2012.9659	19	205	223

P46937|YAP1_HUMAN	K.TANVPQTVPMR.L	Y	55.47	1212.6285	11	77	87
P46937|YAP1_HUMAN	R.TPDDFLNSVDEM(+15.99)DTGDTINQSTLPSQQNR.F	Y	54.16	3254.4158	29	412	440
P46937|YAP1_HUMAN	K.LDKESFLTWL	Y	53.4	1250.6547	10	495	504
P46937|YAP1_HUMAN	V.RGDSETDLEALFNAVMNPK.T	Y	53.24	2106.0051	19	58	76
P46937|YAP1_HUMAN	N.SSDPFLNSGTYHSR.D	Y	47.23	1566.7063	14	381	394
P46937|YAP1_HUMAN	R.LQQLQMEK.E	Y	47.15	1016.5325	8	308	315
P46937|YAP1_HUMAN	D.SETDLEALFNAVMNPK.T	Y	47.05	1777.8556	16	61	76
P46937|YAP1_HUMAN	R.TPDDFLNSVDEM(+15.99)D.T	Y	43.44	1512.5927	13	412	424
P46937|YAP1_HUMAN	R.GDSETDLEALFNAVMN(+.98)PK.T	Y	41.45	1950.8881	18	59	76
P46937|YAP1_HUMAN	R.LKQQELLR.Q	Y	39.64	1026.6185	8	320	327
P46937|YAP1_HUMAN	R.SQLPTLEQDGGTQN(+.98)PVSSPGM(+15.99)SQELR.T	Y	37.47	2772.2871	26	350	375
P46937|YAP1_HUMAN	K.ESFLTWL	Y	35.1	894.4487	7	498	504
P46937|YAP1_HUMAN	Q.SSFEIPDDVPLPAGWEMAK.T	Y	34.21	2087.9873	19	163	181
P46937|YAP1_HUMAN	R.ISQSAPVK.Q	Y	32.99	828.4705	8	273	280
P46937|YAP1_HUMAN	G.DSETDLEALFNAVMNPK.T	Y	28.98	1892.8826	17	60	76
P46937|YAP1_HUMAN	R.GDSETDLEALFN.A	Y	26.45	1309.5674	12	59	70
P46937|YAP1_HUMAN	R.TPDDFLNSVDEMDTGDTIN(+.98)QSTLPSQQNR.F	Y	25.4	3238.4209	29	412	440
P46937|YAP1_HUMAN	K.QQELLR.Q	Y	23.13	785.4395	6	322	327
P46937|YAP1_HUMAN	Q.QELLR.Q	Y	21.01	657.381	5	323	327
P46937|YAP1_HUMAN	R.YFLNHIDQTTT.W	Y	18.27	1351.6409	11	188	198
P46937|YAP1_HUMAN	R.GDSETDLEALFNAVMN.P	Y	17.64	1724.7563	16	59	74
P46937|YAP1_HUMAN	L.Q(+.98)EALSSDILN(+.98)DM(+15.99)ESVLAATK.L	Y	15.21	2152.0093	20	475	494
P35222|CTNB1_HUMAN	K.AAVSHWQQQSYLDSGIHSGATTTAPSLSGK.G	Y	98.27	3084.49	30	20	49
P35222|CTNB1_HUMAN	K.HAVVNLINYQDDAELATR.A	Y	85.2	2041.0228	18	134	151
P35222|CTNB1_HUMAN	Q.SYLDSGIHSGATTTAPSLSGK.G	Y	84.84	2049.0015	21	29	49
P35222|CTNB1_HUMAN	R.TEPMAWNETADLGLDIGAQGEPLGYR.Q	Y	79.72	2803.3123	26	685	710
P35222|CTNB1_HUMAN	R.TSM(+15.99)GGTQQQFVEGVR.M	Y	79.7	1639.7625	15	551	565
P35222|CTNB1_HUMAN	R.TSMGGTQQQFVEGVR.M	Y	77.84	1623.7675	15	551	565
P35222|CTNB1_HUMAN	R.NEGVATYAAAVLFR.M	Y	77.35	1480.7673	14	648	661
P35222|CTNB1_HUMAN	Y.AITTLHNLLLHQEGAK.M	Y	74.22	1757.9788	16	255	270
P35222|CTNB1_HUMAN	Y.LDSGIHSGATTTAPSLSGK.G	Y	73.79	1798.9061	19	31	49
P35222|CTNB1_HUMAN	W.NETADLGLDIGAQGEPLGYR.Q	Y	73.14	2088.0122	20	691	710
P35222|CTNB1_HUMAN	M.ATQADLM(+15.99)ELDMAMEPDR.K	Y	70.15	1951.8325	17	2	18
P35222|CTNB1_HUMAN	S.YLDSGIHSGATTTAPSLSGK.G	Y	69.03	1961.9694	20	30	49
P35222|CTNB1_HUMAN	R.HQEAEM(+15.99)AQNAVR.L	Y	69.01	1398.631	12	475	486
P35222|CTNB1_HUMAN	K.HAVVNLIN(+.98)YQDDAELATR.A	Y	68.71	2042.0068	18	134	151
P35222|CTNB1_HUMAN	R.MEEIVEGC(+57.02)TGALHILAR.D	Y	68.41	1897.939	17	566	582
P35222|CTNB1_HUMAN	H.AVVNLINYQDDAELATR.A	Y	67.59	1903.9639	17	135	151
P35222|CTNB1_HUMAN	M.ATQADLMELDMAMEPDR.K	Y	67.25	1935.8376	17	2	18
P35222|CTNB1_HUMAN	M.ATQADLM(+15.99)ELDM(+15.99)AMEPDR.K	Y	66.69	1967.8274	17	2	18
P35222|CTNB1_HUMAN	R.AAM(+15.99)FPETLDEGMQIPSTQFDAAHPTNVQR.L	Y	66.62	3217.4807	29	96	124
P35222|CTNB1_HUMAN	R.TMQNTNDVETAR.C	Y	66.38	1378.6147	12	201	212
P35222|CTNB1_HUMAN	R.HQEAEMAQNAVR.L	Y	66.17	1382.6361	12	475	486
P35222|CTNB1_HUMAN	R.MSEDKPQDYK.K	Y	65.62	1239.5441	10	662	671
P35222|CTNB1_HUMAN	K.LIILASGGPQALVNIMR.T	Y	65.4	1765.0283	17	313	329
P35222|CTNB1_HUMAN	K.LIILASGGPQALVNIM(+15.99)R.T	Y	64.96	1781.0233	17	313	329
P35222|CTNB1_HUMAN	R.HQEAEMAQN(+.98)AVR.L	Y	64.86	1383.6201	12	475	486
P35222|CTNB1_HUMAN	T.TLHNLLLHQEGAK.M	Y	64.77	1472.8099	13	258	270
P35222|CTNB1_HUMAN	K.MMVC(+57.02)QVGGIEALVR.T	Y	64.11	1561.7778	14	436	449
P35222|CTNB1_HUMAN	K.EAAEAIEAEGATAPLTELLH.S	Y	63.94	2035.0109	20	626	645
P35222|CTNB1_HUMAN	V.VNLINYQDDAELATR.A	Y	62.14	1733.8584	15	137	151
P35222|CTNB1_HUMAN	L.ASGGPQALVNIMR.T	Y	61.8	1312.6921	13	317	329
P35222|CTNB1_HUMAN	T.LHNLLLHQEGAK.M	Y	61.26	1371.7622	12	259	270
P35222|CTNB1_HUMAN	M.ATQADLM(+15.99)ELDMAM(+15.99)EPDR.K	Y	61.11	1967.8274	17	2	18
P35222|CTNB1_HUMAN	R.C(+57.02)TAGTLHNLSHHR.E	Y	60.31	1502.7161	13	213	225
P35222|CTNB1_HUMAN	R.N(+.98)EGVATYAAAVLFR.M	Y	60.19	1481.7513	14	648	661
P35222|CTNB1_HUMAN	K.LLHPPSHWPLIK.A	Y	59.56	1436.8292	12	497	508
P35222|CTNB1_HUMAN	R.SPQMVSAIVR.T	Y	59.34	1086.5856	10	191	200
P35222|CTNB1_HUMAN	K.AAVMVHQLSK.K	Y	58.8	1082.5906	10	171	180

P35222|CTNB1_HUMAN	K.EAAEAIEAEGATAPLTEL.L	Y	58.64	1784.8679	18	626	643
P35222|CTNB1_HUMAN	R.LHYGLPVVVK.L	Y	58.37	1123.6753	10	487	496
P35222|CTNB1_HUMAN	S.NKPAIVEAGGMQALGLHLTDPSQR.L	Y	57.9	2502.3013	24	353	376
P35222|CTNB1_HUMAN	N.LINYQDDAELATR.A	Y	56.97	1520.7471	13	139	151
P35222|CTNB1_HUMAN	K.LLNDEDQVVVNK.A	Y	56.71	1384.7197	12	159	170
P35222|CTNB1_HUMAN	M.QALGLHLTDPSQR.L	Y	56.41	1434.7579	13	364	376
P35222|CTNB1_HUMAN	F.YAITTLHNLLLHQEGAK.M	Y	56.27	1921.0421	17	254	270
P35222|CTNB1_HUMAN	R.TMQNTN(+.98)DVETAR.C	Y	56.25	1379.5988	12	201	212
P26358|DNMT1_HUMAN	R.VPTLAVPAISLPDDVR.R	Y	53.26	1661.9352	16	10	25
P26358|DNMT1_HUMAN	R.VLEQLEDLDSR.V	Y	44.2	1315.6619	11	910	920
P26358|DNMT1_HUMAN	R.Q(+.98)TIRHSTR.E	Y	24.4	998.5258	8	602	609
P26358|DNMT1_HUMAN	F.TFNIK.L	Y	21.99	621.3486	5	947	951
P26358|DNMT1_HUMAN	P.EIEIK.L	Y	20.02	630.3588	5	1131	1135
P26358|DNMT1_HUMAN	R.FESPPK.T	Y	19.05	703.3541	6	876	881
P26358|DNMT1_HUMAN	R.HIPLAPGSDWR.D	Y	17.38	1247.6411	11	1427	1437
P26358|DNMT1_HUMAN	N.GILYR.V	Y	14.53	620.3646	5	930	934
Q8NFU7|TET1_HUMAN	R.CGHGEEQK.L	Y	56.04	854.7264	8	725	732
Q8NFU7|TET1_HUMAN	K.TLENNSYK.F	Y	55.77	834.0024	8	835	842
Q8NFU7|TET1_HUMAN	K.DGSPVQPSLLSLMK.D	Y	55.69	1459.4944	14	919	932
Q8NFU7|TET1_HUMAN	K.GDFSANVPEAEK.S	Y	54.81	1329.2352	12	774	785
Q8NFU7|TET1_HUMAN	K.PTVVSYQENDR.Q	Y	53.73	1195.4932	11	1213	1223
Q8NFU7|TET1_HUMAN	K.DSELPTCSCLDR.V	Y	53.52	1226.4948	12	1466	1477
Q8NFU7|TET1_HUMAN	K.QYAPVAYQNQVEYENVAR.E	Y	53.31	1817.8812	18	1679	1696
Q8NFU7|TET1_HUMAN	R.DIHNMNNGSTVVCTLTR.E	Y	53.29	1738.9895	17	1724	1740
Q8NFU7|TET1_HUMAN	K.LSDTDEFGSK.E	Y	53.18	1110.194	10	1763	1772
Q9Y3Y2|CHTOP_HUMAN	R.ASMQQQQQLASAR.N	Y	77.5	1445.7045	13	39	51
Q9Y3Y2|CHTOP_HUMAN	R.LAQQMENRPSVQAALK.L	Y	58.7	1782.941	16	55	70
Q9Y3Y2|CHTOP_HUMAN	K.QPTPVNIR.A	Y	51.84	923.5189	8	31	38
Q9Y3Y2|CHTOP_HUMAN	R.ASMQQQQQLASAR.N	Y	61.37	1445.7045	13	39	51
Q9Y3Y2|CHTOP_HUMAN	R.LAQQMENRPSVQAALK.L	Y	55.43	1782.941	16	55	70
Q9Y3Y2|CHTOP_HUMAN	R.LAQQMENR.P	Y	38.77	988.476	8	55	62
Q9Y3Y2|CHTOP_HUMAN	R.FTNMLK.N	Y	36.61	752.3891	6	23	28
Q9Y3Y2|CHTOP_HUMAN	K.QPTPVNIR.A	Y	35.07	923.5189	8	31	38
Q99873|ANM1_HUMAN	K.WLAPDGLIFPDR.A	Y	49.46	1398.7295	12	174	185
Q99873|ANM1_HUMAN	K.EVDIYTVK.V	Y	32.29	965.507	8	244	251
Q99873|ANM1_HUMAN	K.EPLVDVVDPK.Q	Y	20.95	1109.5968	10	224	233
Q99873|ANM1_HUMAN	K.EPLVDVVDPK.Q	Y	40.62	1109.5968	10	224	233
Q99873|ANM1_HUMAN	K.LDHVVTIIK.G	Y	35.64	936.828	9	135	143
Q99873|ANM1_HUMAN	K.WLAPDGLIFPDR.A	Y	43.19	1307.328	12	184	195
Q99873|ANM1_HUMAN	R.NDYVHALVAYFNIEFTR.C	Y	17.64	1867.2324	17	277	293
Q9UMN6|KMT2B_HUMAN	R.GRPPLTPSQR.A	Y	16.37	1107.6149	10	470	479
Q9UMN6|KMT2B_HUMAN	R.GAGGGGGR.G	Y	68.09	879.7152	8	76	83
Q9UMN6|KMT2B_HUMAN	R.LADVAPTPPK.T	Y	66.22	1061.076	10	216	225
Q9UMN6|KMT2B_HUMAN	R.GEEGTER.M	Y	65.62	766.4209	7	232	238
Q9UMN6|KMT2B_HUMAN	K.VEVSPVLR.P	Y	67.82	842.74	8	607	614
Q9UMN6|KMT2B_HUMAN	R.APTPPSTPVPLPEK.R	Y	57.82	1436.5932	14	636	649
Q9UMN6|KMT2B_HUMAN	R.PSGPESPVQGPR.I	Y	55.25	1236.7488	12	906	917
Q9UMN6|KMT2B_HUMAN	R.VQDCGSCVNCLDK.P	Y	58.51	1450.5127	13	1025	1037
Q9UMN6|KMT2B_HUMAN	K.PTLQPVLQLK.A	Y	58.61	1066.569	10	1176	1185
Q9UMN6|KMT2B_HUMAN	K.GNYCPICTR.C	Y	62.22	945.3321	9	1385	1393
P35221|CTNA1_HUMAN	R.VIHVVTSEMDNYEPGVYTEK.V	Y	107.87	2309.0886	20	552	571
P35221|CTNA1_HUMAN	K.NVPILYTASQAC(+57.02)LQHPDVAAYK.A	Y	98.65	2458.2314	22	217	238
P35221|CTNA1_HUMAN	R.FTEQVEAAVEALSSDPAQPMDENEFIDASR.L	Y	96.9	3295.4827	30	587	616
P35221|CTNA1_HUMAN	K.AHVLAASVEQATEN(+.98)FLEK.G	Y	90.4	1956.9792	18	58	75
P25054|APC_HUMAN	K.SPSEGQTATTSPR.G	Y	21.32	1317.6161	13	2260	2272
P25054|APC_HUMAN	K.REVPK.N	Y	17.9	627.3704	5	1450	1454
P25054|APC_HUMAN	M.KEVLK.Q	Y	16.29	615.3955	5	45	49
O60341|KDM1A_HUMAN	K.DEMVEQEFNR.L	Y	33.28	1047.564	10	375	384
O60341|KDM1A_HUMAN	R.STSQTFIYK.C	Y	26.69	988.0776	9	659	667

O60341|KDM1A_HUMAN	R.VHSYLER.H	Y	14.71	772.1651	7	252	258
O60341|KDM1A_HUMAN	R.GELFLFWNLYK.A	Y	14.84	1198.0232	11	749	759
O60341|KDM1A_HUMAN	R.NGYSCVPVALAEGLDIK.L	Y	21.61	1836.1717	17	856	872

Table S9. Primers and Oligonucleotides used in this study

**Primer used for subcloning and plasmid construction:**	
RNF219-up	5’-gccGCTAGCgccATGGAGCAGAAACTCATCTCTGAAGAGGATCTGGCTCAGACCGTGCAGAATGT-3’
RNF219-dn	5’-gccACTAGTTCAACTTTTAGTTGCTTTTG-3’
RNF219- mutated primer up	5’-GACGTACCTTCCCCAAGCTAATGTGGCTCGTGATGGGCAGAGTGAGCGAC-3’
RNF219- mutated primer dn	5’-GTCGCTCACTCTGCCCATCACGAGCCACATTAGCTTGGGGAAGGTACGTC-3’
LGALS3-up	5’-ATGGCAGACAATTTTTCGC-3’
LGALS3-dn	5’-TTATATCATGGTATATGAAGCACTG-3’
CTNNA1-up	5’-gccGCTAGCgccATGGACTACAAGGACGACGATGACAAGACTGCTGTCCATGCAGGC-3’
CTNNA1-dn	5’-gccACTAGTTTAGATGCTGTCCATAGCTTT-3’
CTNNA1 1aa-290aa-up	5’-gccGCTAGCgccATGGACTACAAGGACGACGATGACAAGACTGCTGTCCATGCAGGC-3’
CTNNA1 1aa-290aa-dn	5’-gccACTAGTTTAAATGATTTGTTTGTCAAA-3’
CTNNA1 291aa-650aa-up	5’-gccGCTAGCgccATGGACTACAAGGACGACGATGACAAGGTGGACCCCTTGAGCTTC-3’
CTNNA1 291aa-650aa-dn	5’-gccACTAGTTTAGACATCAAAATCTTCTGT-3’
CTNNA1 651aa-906aa-up	5’-gccGCTAGCgccATGGACTACAAGGACGACGATGACAAGAGAAGCAGGACGAGCGTC-3’
CTNNA1 651aa-906aa-dn	5’-gccACTAGTTTAGATGCTGTCCATAGCTTT-3’
CTNNB1-up	5’-ATGGCTACTCAAGCTGATTT-3’
CTNNB1-dn	5’-TTACAGGTCAGTATCAAACC-3’
TCF4-up	5’-ATGGACTACAAGGACGACGATGACAAGCCGCAGCTGAACGGCGG-3’
TCF4-dn	5’-CTATTCTAAAGACTTGGTGA-3’
YAP1-up	5’-ATGGATCCCGGGCAGCAG-3’
YAP1-dn	5’-CTATAACCATGTAAGAAAGCTTTCTTTAT-3’
TEAD4-up	5’-ATGTTGGAGGGCACGGC-3’
TEAD4-dn	5’-CATTCTTTCACCAGCCTGTAGATG-3’
**Primer used for CAPTURE system**	
LGALS3-sgRNA-1	5’-ggagaaCCACCTTGTTGGTGTGACTTTGGATTGGGTAATAAAGAGCCGTTTATTGCATGTGTA-3’
LGALS3-sgRNA-2	5’-ggagaaCCACCTTGTTGGCCCAGCCACACTGTGACTTCTATGTGCAAGCCTAGAGGGGACGA-3’
LGALS3-sgRNA-3	5’-ggagaaCCACCTTGTTGGGCTTCTGTAACAAAATATCATAAACTGGGTAGCTTATAGGCAATA-3’
LGALS3-sgRNA-4	5’-ggagaaCCACCTTGTTGGGTCAGTTCCCTAGGGAATAGACATTGAAAAGATTTTTGGTTTTGTA-3’
LGALS3-sgRNA-5	5’-ggagaaCCACCTTGTTGGCTGGCGTTCGGGGAAGGTTGGCAGCACCTTACGAGACCCACACA-3’
**Primer used for qPCR**	
c-Myc-up	5’-GGCTCCTGGCAAAAGGTCA-3’
c-Myc-dn	5’-CTGCGTAGTTGTGCTGATGT-3’
Axin2-up	5’-CAACACCAGGCGGAACGAA-3’
Axin2-dn	5’-GCCCAATAAGGAGTGTAAGGACT-3’

DKK1-up	5'-CCTTGAACTCGGTTCTCAATTCC-3'
DKK1-dn	5'-CAATGGTCTGGTACTTATTCCCG-3'
TWIST1-up	5'-GTCCGCAGTCTTACGAGGAG-3'
TWIST1-dn	5'-GCTTGAGGGTCTGAATCTTGCT-3'
SOX4-up	5'-AGCGACAAGATCCCTTTCATTC-3'
SOX4-dn	5'-CGTTGCCGGACTTCACCTT-3'
GBX2-up	5'-CCGCCTTCAGCATAGACTCG-3'
GBX2-dn	5'-GGTAGCCGGTGTAGACGAAAT-3'
CCND1-up	5'-GGTGGGGTCATGTGTGTGG-3'
CCND1-dn	5'-CGGTTCAGGTACTCAGTCATCC-3'
BIRC5-up	5'-AGGACCACCGCATCTCTACAT-3'
BIRC5-dn	5'-AAGTCTGGCTCGTTCTCAGTG-3'
CYR61-up	5'-CTCGCCTTAGTCGTCACCC-3'
CYR61-dn	5'-CGCCGAAGTTGCATTCCAG-3'
SOX2-up	5'-GCCGAGTGGAAACTTTTGTCG-3'
SOX2-dn	5'-GGCAGCGTGTACTTATCCTTCT-3'
LGALS3-up	5'-ATGGCAGACAATTTTTCGCTCC-3'
LGALS3-dn	5'-GCCTGTCCAGGATAAGCCC-3'
LGR5-up	5'-CTCCCAGGTCTGGTGTGTTG-3'
LGR5-dn	5'-GAGGTCTAGGTAGGAGGTGAAG-3'
Cyclin D1-up	5'-GCTGCGAAGTGGAAACCATC-3'
Cyclin D1-dn	5'-CCTCCTTCTGCACACATTTGAA-3'
SNAI2-up	5'-CGAACTGGACACACATACAGTG-3'
SNAI2-dn	5'-CTGAGGATCTCTGGTTGTGGT-3'
JAG1-up	5'-GTCCATGCAGAACGTGAACG-3'
JAG1-dn	5'-GCGGGACTGATACTCCTTGA-3'
CTGF-up	5'-CAGCATGGACGTTCGTCTG-3'
CTGF-dn	5'-AACCACGGTTTGGTCCTTGG-3'
AMOTL2-up	5'-GCTCGTTGAGTGAACGGCT-3'
AMOTL2-dn	5'-CATGAGCTAGTACAACATGAGGG-3'
IGFBP3-up	5'-AGAGCACAGATACCCAGAACT-3'
IGFBP3-dn	5'-GGTGATTCAGTGTGTCTTCCATT-3'
ITGB2-up	5'-TGCGTCCTCTCTCAGGAGTG-3'
ITGB2-dn	5'-GGTCCATGATGTCGTCAGCC-3'
GLI2-up	5'-CTGCCTCCGAGAAGCAAGAAG-3'
GLI2-dn	5'-GCATGGAATGGTGGCAAGAG-3'
AXL-up	5'-GTGGGCAACCCAGGGAATATC-3'
AXL-dn	5'-GTACTGTCCCGTGTCGGAAAG-3'

RNF219-up	5’-GCCACATTTGCTTGGGGAAG-3’
RNF219-dn	5’-GCAAGCTGGACACTGGCTAT-3’
**Primer used for ChIP-qPCR**	
LGALS3 P1 RT primer up	5’-GATGTGACTTTGGATTGGGTAA-3’
LGALS3 P1 RT primer dn	5’-CTGTGGAGGCAAGGGATG-3’
LGALS3 P2 RT primer up	5’-CGTGCCAACCACTAAGAC-3’
LGALS3 P2 RT primer dn	5’-CTGGCTTATGTAAACTATGGAG-3’
LGALS3 P3 RT primer up	5’-GTCGGGAAATCCAAGATC-3’
LGALS3 P3 RT primer dn	5’-CACGGTGATGTATTAGGAAG-3’
LGALS3 P4 RT primer up	5’-AAGATTTTTGGTTTTGTATTCTACA-3’
LGALS3 P4 RT primer dn	5’-GCCACCGTTAGGTTCCGT-3’
LGALS3 P5 RT primer up	5’-CAGCCAACGAGCGGTGAG-3’
LGALS3 P5 RT primer dn	5’-CCTGGAGGGCAGAGCACA-3’
RNF219 RT primer up	5’-AGCGCACCGTGATTAATGGA-3’
RNF219 RT primer dn	5’-TATCCCTGCGATGCGTTTCT-3’
**Primer used for 5-hmC / 5-mC Analysis**	
Control primer 1:	5’-CTGTCATGGTGACAAAGGCATC-3’
Control primer 2:	5’-CAGGATTTCTCTATTATGAAGACCTTG-3’
**SiRNA**	
Si RNF219#1	5’-CACTGGATTTGGATGGGTTAT-3’
Si RNF219#2	5’-ATAACCCATCCAAATCCAGTG-3’
Si LGALS3#1	5’-TGCTGATAACAATTCTGGGCAC-3’
Si LGALS3#2	5’-TGAAGCGTGGGTTAAAGTGGA-3’
Si CTNNB1#1	5’-CGCATGGAAGAAATAGTTGAA-3’
Si CTNNB1#2	5’-GCTTGGAATGAGACTGCTGAT-3’
Si YAP1#1	5’-CAGGTGATACTATCAACCAAA-3’
Si YAP1#2	5’-TTTGGTTGATAGTATCACCTG-3’
Si TET1#1	5’-CCTCCAGTCTTAATAAGGTTA-3’
Si TET1#2	5’-TAACCTTATTAAGACTGGAGG-3’
Si MLL4#1	5’-CGAATGGAAGAACAACGTGTA-3’
Si MLL4#2	5’-TACACGTTGTTCTTCCATTCG-3’
Si PRMT1#1	5’-TGAGCGTTCCTAGGCGGTTTC-3’
Si PRMT1#2	5’-GAAACCGCCTAGGAACGCTCA-3’
Si CHTOP#1	5’-TTTACAGATGTGTTGACTAAC-3’
Si CHTOP#2	5’-ATCCAATTGGTTGTCCAGCTG-3’

Abbreviations list

LGALS3, galectin 3; **ELISA**, enzyme-linked immunosorbent; **IF**, immunofluorescence; **μCT**, micro-CT; **RNF219**, ring finger protein 219; **IB**, immunoblotting; **IHC**, immunohistochemistry; **YAP1**, Yes1 associated transcriptional regulator; **CAPTURE**, CRISPR affinity purification in situ of regulatory elements; **co-IP**, co-immunoprecipitation; **ChIP**, chromatin immunoprecipitation; **BM**, bone metastasis; **HCC-BM**, HCC with bone metastasis; **SREs**, skeletal-related events; **TRAP**, tartrate-resistant acid phosphatase; **MMPs**, matrix metalloproteinases; -mu, mutated; **ShRNAs**, short hairpin RNAs; **TCF/LEF1**, T cell factor / lymphoid enhancer factor 1; **TEAD**, TEA domain; **siRNAs**, short interfering RNAs; **FB-dCas9**, N-terminal Flag and biotin-acceptor-site (FB)-tagged dCas9; **dCas9**, dead Cas9 **sgRNAs**, small guide RNAs; **DNMT1**, DNA methyltransferase 1; **CHTOP**, chromatin target of PRMT1; **PRMT1**, protein arginine methyltransferase 1; **TET1**, tet methylcytosine dioxygenase 1; **MLL4**, lysine methyltransferase 2D; **APC**, APC regulator of WNT signaling pathway; **LSD1**, lysine demethylase 1; **H3K4me3**, Histone H3 (tri methyl K4); **H4R3me1**, Histone H4 (mono methyl R3); **H4R3me2a**, Histone H4R3 Dimethyl Asymmetric; **H3R2me1**, Histone H3 (mono methyl R2); **H3R2me2s**, Histone H3 (symmetric di methyl R2); **H3R8me2s**, Histone H3 (symmetric di methyl R8); **c-SRC**, SRC proto-oncogene, non-receptor tyrosine kinase; **p-SYK**, phosphorylation-spleen associated tyrosine kinase; **p-VAV-3**, phosphorylation-vav guanine nucleotide exchange factor 3; **RAC1**, Rac family small GTPase 1; **CD98**, solute carrier family 7 member 5; **H3**, Histone 3; **CM**, conditioned media; w/v, weight/volume; **αvβ3**, integrin αVβ3; **BSA**, albumin from bovine serum; **DAPI**, 4',6-diamidino-2-phenylindole; **SI**, staining index; **TGF-β**, transforming growth factor beta 1;

RANKL, TNF superfamily member 11; **OPG**, TNF receptor superfamily member 11b; **MTT**, 3-(4, 5-Dimethylthiazol-2-yl)-2, 5- diphenyltetrazolium bromide; **DMSO**, dimethyl sulfoxide; **SEM**, scanning electron microscopy; **H&E stained**, hematoxylin-eosin staining; **ALP**, Alkaline phosphatase; **BLI**, bioluminescence imaging; **BMD**, bone mineral density; **OC**, osteoclast; **OP**, osteoclast precursor; **M-CSF**, macrophage-colony stimulating factor; **C-fos**, fos proto-oncogene; **AP-1**, transcription factor subunit; **Acp5**, acid phosphatase 5, tartrate resistant (TRAP); **Ctsk**, cathepsin K; **Nfat-c1**, nuclear factor of activated T cells, cytoplasmic, calcineurin dependent 1; **Dc-stamp**, dendrocyte expressed seven transmembrane protein; **qPCR**, quantitative Real-time PCR; **VH domain**, vinculin homology domain; **5mC/5hmC**, 5-methylcytosine/5-hydroxymethylcytosine; **WDR5**, WD repeat domain 5; **FDA**, Food and Drug Administration;
